# Prevalence, Genotype Diversity, and Distinct Pathogenicity of 205 Gammacoronavirus Infectious Bronchitis Virus Isolates in China during 2019–2023

**DOI:** 10.3390/v16060930

**Published:** 2024-06-07

**Authors:** Ting Xiong, Hangao Xie, Lin Li, Shijin Liang, Meizhen Huang, Chuanzhao Yu, Tingting Zhuang, Xuejing Liang, Dingxiang Liu, Ruiai Chen

**Affiliations:** 1 College of Veterinary Medicine, South China Agricultural University, Guangzhou 510640, China; 2 Zhaoqing Branch of Guangdong Laboratory of Lingnan Modern Agricultural Science and Technology, Zhaoqing 526238, China; 3 Key Laboratory of Manufacture Technology of Veterinary Bioproducts, Ministry of Agriculture and Rural Affairs, Zhaoqing 526238, China; 4 Integrative Microbiology Research Centre, South China Agricultural University, Guangzhou 510642, China

**Keywords:** IBV, epidemic trend, homologous recombination, pathogenicity

## Abstract

Gammacoronavirus infectious bronchitis virus (IBV) causes a highly contagious disease in chickens and seriously endangers the poultry industry. The emergence and co-circulation of diverse IBV serotypes and genotypes with distinct pathogenicity worldwide pose a serious challenge to the development of effective intervention measures. In this study, we report the epidemic trends of IBV in China from 2019 to 2023 and a comparative analysis on the antigenic characteristics and pathogenicity of isolates among major prevalent lineages. Phylogenetic and recombination analyses based on the nucleotide sequences of the spike (S) 1 gene clustered a total of 205 isolates into twelve distinct lineages, with GI-19 as a predominant lineage (61.77 ± 4.56%) exhibiting an overall increasing trend over the past five years, and demonstrated that a majority of the variants were derived from gene recombination events. Further characterization of the growth and pathogenic properties of six representative isolates from different lineages classified four out of the six isolates as nephropathogenic types with mortality rates in one-day-old SPF chickens varying from 20–60%, one as a respiratory type with weak virulence, and one as a naturally occurring avirulent strain. Taken together, our findings illuminate the epidemic trends, prevalence, recombination, and pathogenicity of current IBV strains in China, providing key information for further strengthening the surveillance and pathogenicity studies of IBV.

## 1. Introduction

Coronaviruses are enveloped, single-stranded and positive-sensed RNA viruses in the order *Nidovirales* [1], and are classified into four genera, alpha-, beta-, gamma-, and deltacoronavirus. Infectious bronchitis virus (IBV), discovered in North Dakota, USA, in 1931, is the prototype coronavirus in the gammacoronavirus genus and mainly infects chickens with a wide range of tissue tropism, causing damage to the respiratory, genitourinary, and digestive systems [2]. Chickens aged from one to four weeks are most susceptible, with a mortality rate as high as 20% in severe cases [3]. Furthermore, IBV infection can also result in a deterioration of the performance in broilers and laying hens, leading to a decrease in the food conversion ratio as well as reductions in both egg production and quality. Some IBV strains are capable of causing intestinal, glandular, and muscular diseases. Currently, vaccination remains the most critical measure for the prevention and control of this disease, with live attenuated vaccines being the most widely utilized. Vaccine strains commonly used in China, including H120, H52, LDT3-A, W93, FNO-E55, HC13-91, and Ma5, are Mass-type and evolutionarily distant from currently predominant isolates [4].

The genome of IBV encodes four major structural proteins: the phosphorylated nucleocapsid (N) protein, the membrane (M) glycoprotein, the envelope (E) protein, and the spike (S) glycoprotein. The S protein can be post-translationally cleaved by host proteases into two polypeptide components, known as S1 and S2. The S1 subunit is responsible for inducing neutralizing antibodies that are specific to serotypes and inhibit hemagglutination, leading to protective immunity [5]. As nucleotide sequences in the S1 region are highly heterogenous, particularly in the three hypervariable regions (HVRs) [6], analysis of the complete or partial S1 gene sequences is commonly used to determine the genetic types of IBV. A comprehensive genetic classification system was proposed by Valastro et al. in 2016, to classify IBV into six genotypes (GI-GVI) and thirty-two lineages (GI-1~GI-27, GII-1, GII-2, GIV-1, GV-1, and GVI-1) [7]. A new genotype, GVII, and two lineages, GI-28 and GI-29, were subsequently added, resulting in the current seven genotypes (GI-GVII) and thirty-five lineages (GI-1~GI-29, GII-1, GII-2, GIV-1, GV-1, GVI-1, and GVII-1) for the classification of IBV [8]. Lineage GI-19 (QX- or LX4-type) has become the most prevalent strain in China in the last decade [9,10,11]. Meanwhile, at least four other main lineages, including GI-1 (Mass-type), GI-7 (TW-type), GI-13 (4/91-type), and GVI-1 (Tc07-2-type), were found to be co-circulating in the same period.

The evolution of IBV typically occurs through genomic replacement, deletion, insertion, and RNA recombination in the S1 gene [12]. Comparative analysis of the phylogenetic evolution of S, E, M, and N protein sequences from different IBV strains revealed that strains with less than 89% similarity belong to distinct serotypes [13,14]. This suggests that even a minor change in the highly mutable region of the S1 gene can result in a serotype switch and ultimately lead to limited cross-protection between different lineages. Therefore, investigation and characterization of prevalent strains of IBV are of significance in developing vaccines with enhanced adaptability.

In this study, we report the epidemic trends of IBV in China from 2019 to 2023. Isolation, sequencing, and phylogenic analysis of 205 isolates revealed a multi-lineage pattern, with GI-19 (QX-like) as the predominant strain responsible for over 60% of cases and six other lineages and variants as minor strains. Further characterization of six representative isolates in different lineages demonstrated distinct growth and pathogenic properties among different isolates. Based on a comprehensive surveillance and analysis of IBV epidemic dynamics in China over the past five years, this study provides key information on the emergence and prevalence of different IBV variants as well as their distinct pathogenicity, guiding the development of new prevention and control strategies as well as novel vaccines.

## 2. Materials and Methods

### 2.1. The Materials, Animals, and Ethics Statement

Specific pathogen-free (SPF) chicken embryos and 1-day-old SPF chickens were purchased from Xinxing Dahuanong Poultry Egg Co., Ltd. (Guangdong, China). The study did not involve endangered or protected species, and was approved by the Animal Experiments Committee of Zhaoqing Dahuanong Biopharmaceutical Co., Ltd. (Zhaoqing, China). 

### 2.2. Viruses and S1 Gene Sequencing

A total of 205 strains were isolated from broiler or layer chickens in 19 provinces and regions in China during 2019–2023. These viruses were isolated from suspected positive specimens submitted for clinical examination, and isolates were aliquoted and stored at −80 °C. The viral allantoic fluids were vortexed briefly and centrifuged at 750× *g* for 5 min at 4 °C; 0.2 mL of supernatants per egg was then inoculated into 9-day-old SPF chicken embryonated eggs, and harvested at indicated times post-inoculation. Total RNA was extracted using the Viral RNA Isolation Kit (Corning life sciences, CO., LTD, Wujiang, China), and reverse-transcribed into cDNA using the FastKing RT SuperMix kit (TIANGEN Biotech CO., LTD, Beijing, China). PCR was performed to identify and screen positive samples with specific primers [15], and the positive PCR products were submitted for sequencing (Sangon Biotech, CO., LTD, Shanghai, China).

### 2.3. Phylogenetic, Recombination, Deletion and Insertion Analysis of S1 Gene

The S1 gene of all isolates was sequenced and the complete S1 sequences used in this study are listed in Supplementary File S1. The nucleotide sequences of the S1 gene from all isolates and nine reference strains were used to construct a phylogenetic tree using the neighbor-joining method in MEGA version 7.0, and bootstrap values were determined based on 1000 replicates of the original data [16]. The phylogenetic tree was further enhanced using the online software iTOL v6 (https://itol.embl.de/itol.cgi). Homology analysis of S1 nucleotide (S1-N) and amino acid (S1-A) sequences between representative strains of different lineages and vaccine strain H120 was carried out as described [17]. Briefly, amino acid sequences of analyzed strains were aligned by the ClustalW method in Mega software 7.0, saved as Fasta files, and were subsequently analyzed by the ClustalW method in Megalign software 7.10. As cleavage of the S protein by furin facilitates viral entry, promotes syncytium formation, and enhances viral infectivity [18], the presence of a furin restriction site in IBV lineages was analyzed by multiple alignment and comparative analysis using the online software Weblogo 3 (http://weblogo.berkeley.edu/logo.cgi).

The complete IBV S1 sequences of 205 isolates and 9 reference strains (HM194680.1 (GI-1), MW560630.1(GI-7), KX107840.1 (GI-7), AF093794.1 (GI-13), KX364300.1 (GI-19), MN548289.1 (GI-19), KX107718.1 (GI-22), MH427448.1 (GI-28), and GQ265948.1 (GVI-1)) were analyzed by Megalign software 7.10 and saved as meg. files. Recombination events were assessed to identify putative parental sequences by Recombination Detection Program 4.0 (RDP 4.0, version 4.100) [16], and the data were analyzed using various methods and default program settings, including RDP Bootscan, GeneCony, Maxchi, Chimaera, SiSscan, LARD, 3Seq, and PhylPro. The potential recombination events and breakpoints of variants were determined by similarity plot analysis (SimPlots, SimPlot version 3.5.1.), using a window of 200 bp and a step size of 20 bp. Deletion and insertion events were analyzed by Megalign software.

### 2.4. Growth Characteristics of Isolates in 9-Day-Old SPF Chicken Embryos

The virus solutions were ten-fold diluted in sterilized PBS, ranging from 10^−1^ to 10^−8^ in five replicates. The mortality rate and the presence of lesions (dwarfism and bleeding) in chicken embryos were observed and recorded, followed by calculation of EID_50_ using the Reed–Muench method [19].

Nine-day-old SPF chicken embryos were inoculated with virus at a dose of ~10^3^ EID_50_ in duplicate. Following inoculation, embryos were cultured at 37 °C, and allantoic fluids (200 µL each) were collected at 0, 12, 24, 36, 48, and 60 h post-inoculation, respectively. Viral RNA copy numbers were determined by real-time qPCR (RT-qPCR) and calculated at each time point, and standard curves were constructed.

### 2.5. Pathogenicity Evaluation of Isolates

A total of 35 one-day-old chickens were randomly divided into seven groups, A, B, C, D, E, F, and G, with the control group designated as Group A and other groups infected and labeled as CZ_GI-7 (Group B), GZ_GI-13 (Group C), KP_GI-19 (Group D), KM-1_GI-22 (Group E), QY_GI-28 (Group F), and YC_GVI-1 (Group G). Five Chickens in each group were infected with ~10^5.5^ EID_50_ each of one isolate by the nasal–ocular route, respectively. The infected chicks were observed daily for clinical symptoms, such as tracheal rales, wheezing, nasal discharge, or death, for 7 days. The surviving chicks in each group were euthanized at 7 days post-challenge, and necropsies were performed immediately postmortem.

Trachea, lung, and kidney tissues were collected and fixed in 10% neutral buffered formalin for further histopathological and immunohistochemical analysis, respectively. The immunohistochemical staining was conducted following previously reported protocols [20], using a monoclonal antibody against IBV N as the primary antibody. Meanwhile, total RNAs were extracted from the trachea, lung, and kidney tissues harvested at 7 days post-infection, and the viral RNA copy numbers were quantified by RT-qPCR.

### 2.6. Statistical Analysis

Data were analyzed using the GraphPad software 9 package, and two-way analysis of variance (ANOVA) was used to analyze significant differences between the indicated samples and the respective control samples. Significance levels are presented by the *p*-value (ns, non-significant; * *p* < 0.05; ** *p* < 0.01; *** *p* < 0.001; and **** *p* < 0.0001).

## 3. Results

### 3.1. Isolation, Identification, and Phylogenetic Analysis of Clinical Isolates

**Figure 1:**
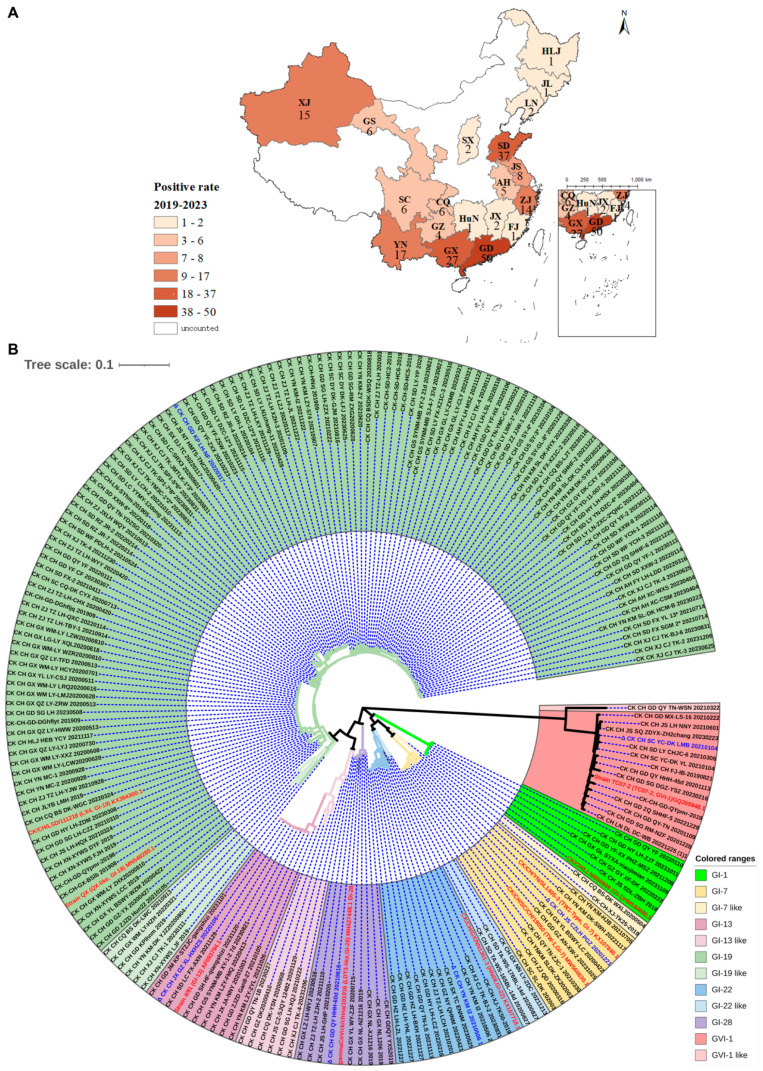
Geographical distribution and evolution analysis of IBV isolates. (**A**) Regional distribution of 205 IBV isolates from 2019 to 2023 in China. (**B**) Evolution analysis of IBV isolates. After conducting a comparative analysis of the S1 sequences between the 205 isolates and reference strains, an evolutionary tree was constructed by the neighbor-joining method using the MEGA7 software, and the resulting evolutionary tree was further beautified utilizing the iTOL online software.

Between 2019 and 2023, we isolated and identified a total of 205 IBV isolates, including 50 from Guangdong (GD), 27 from Guangxi (GX), 17 from Yunnan (YN), 4 from Guizhou (GZ), 1 from Fujian (FJ), 2 from Jiangxi (JX), 1 from Hunan (HuN), 6 from Chongqing (CQ), 6 from Sichuan (SC), 14 from Zhejiang (ZJ), 5 from Anhui (AH), 8 from Jiangsu(JS), 37 from Shandong (SD), 2 from Shanxi (SX), 2 from Liaoning (LN), 1 from Jilin (JL), 1 from Heilongjiang (HLJ), 6 from Gansu (GS), and 15 from Xinjiang (XJ). The numbers and geographic distribution of these isolates are summarized in Figure 1 (Figure 1A).

Based on S1 gene sequences, phylogenetic analysis was carried out using MEGA version 7.0, classifying these 205 isolates and nine reference strains into seven main lineages (GI-1, GI-7, GI-13, GI-19, GI-22, GI-28, and GVI-1) and five variant-lineages (GI-7-like, GI-13-like, GI-19-like, GI-22-like, and GVI-1-like) (Figure 1B). These five variant-lineages exhibit the highest homology with lineages GI - 1, GI - 7, GI - 13, GI-19, GI-22, GI-28, and GVI-1, respectively. However, they clearly belong to distinct clades, and were therefore named as five variant-lineages in this study.

### 3.2. Prevalence of Different Genotypic Isolates and Sequence Variations in the Furin Motifs of S Genes

**Figure 2:**
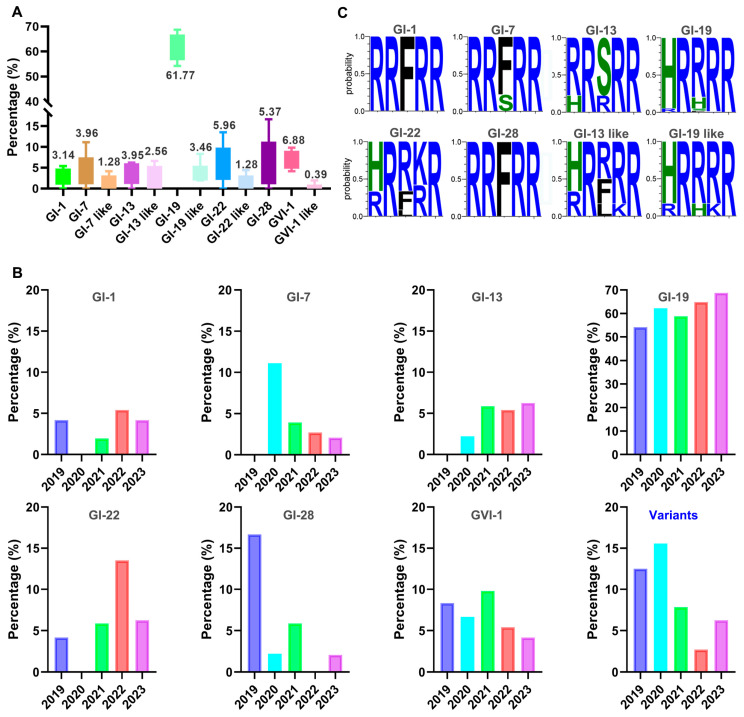
Analysis of proportion and prevalence of different lineages of IBV isolates, as well as the furin motif sequences. (**A**) Percentages of different lineages of 205 isolates from 2019 to 2023 in China. (**B**) The annual proportion of different lineages in 2019–2023. (**C**) Analysis of furin motif sequences in diverse lineages of isolates. The furin motif sequences were aligned using Mega sequencing, saved as Fasta files, and subsequently subjected to analysis using WebLogo 3 online software. The amino acid proportion at a certain position with sequence variation is represented by the relative height of the single amino acid letter.

Statistical analysis of the prevalence of the 205 isolates revealed that GI-19 emerged as the predominant prevalent strain, accounting for an average percentage of 61.77 ± 4.56 over the past five years. GI-1, GI-7, GI-7-like, GI-13, GI-13-like, GI-19-like, GI-22, GI-22-like, GI-28, GVI-1, and GVI-1-like strains constituted 3.14 ± 1.76%, 3.95 ± 3.46%, 1.28 ± 1.53%, 3.95 ± 2.22%, 2.56 ± 2.34%, 3.46 ± 2.34%, 5.96 ± 3.99%, 1.28 ± 1.60%, 5.37 ± 5.44%, 6.88 ± 1.84%, and 0.39 ± 0.72%, respectively, in this period of time (Figure 2A).

Analysis of the prevalent patterns of these isolates in the past five years demonstrated that the prevalence of GI-1 remained relatively stable, prevalence trends of GI-19 and GI-13 isolates were consistently upward, and GI-7 was in a declining trend (Figure 2B). The prevalence of the GVI-1 variant slightly declined in the last two years, but was over 8.97% in the previous year, ranking second only to the predominant GI-19 strain (Figure 2B).

The furin motif sequences in the S protein from isolates with different lineages were then analyzed by Weblogo 3, and the following relatively conserved patterns were observed: RR(F/S)RR for GI-7, (R/H)R(S/R)RR for GI-13, (H/R)R(R/F)(R/K)R for GI-13-like, (H/R)R(R/H/F)RR for GI-19, (H/R)R(R/H)(R/K)R for GI-19-like, (H/R)R(R/F/L)(K/R)R for GI-22, and RRFRR for both GI-1 and GI-28 (Figure 2C). Due to the availability of either a limited number of isolates (fewer than three isolates) or insufficient isolates with the full-length S sequences (fewer than three), the furin motif sequences for the GI-7-like, GI-22-like, GVI-1, and GVI-1-like strains could not be subjected to statistical analysis and were excluded. These results demonstrate that, although there is no significant conservation in the furin motif sequences of different genotypic isolates, they are comparatively more conserved in the GI-1 and GI-28 lineages.

### 3.3. Analysis of Recombination, Deletion, and Insertion on S1 Genes of Different Isolates

Recombination analysis was conducted on the S1 gene sequences of all 214 isolated and reference strains to identify putative parental sequences with statistical significance set at *p*-values <0.05 using RDP 4.0. Table 1 lists the breakpoint positions and specific *p*-values for the analysis of each recombination event in five variant-lineages. The results showed that 80% (4/5) variant-lineages were involved in potential recombination events (Table 1).

**Figure 3:**
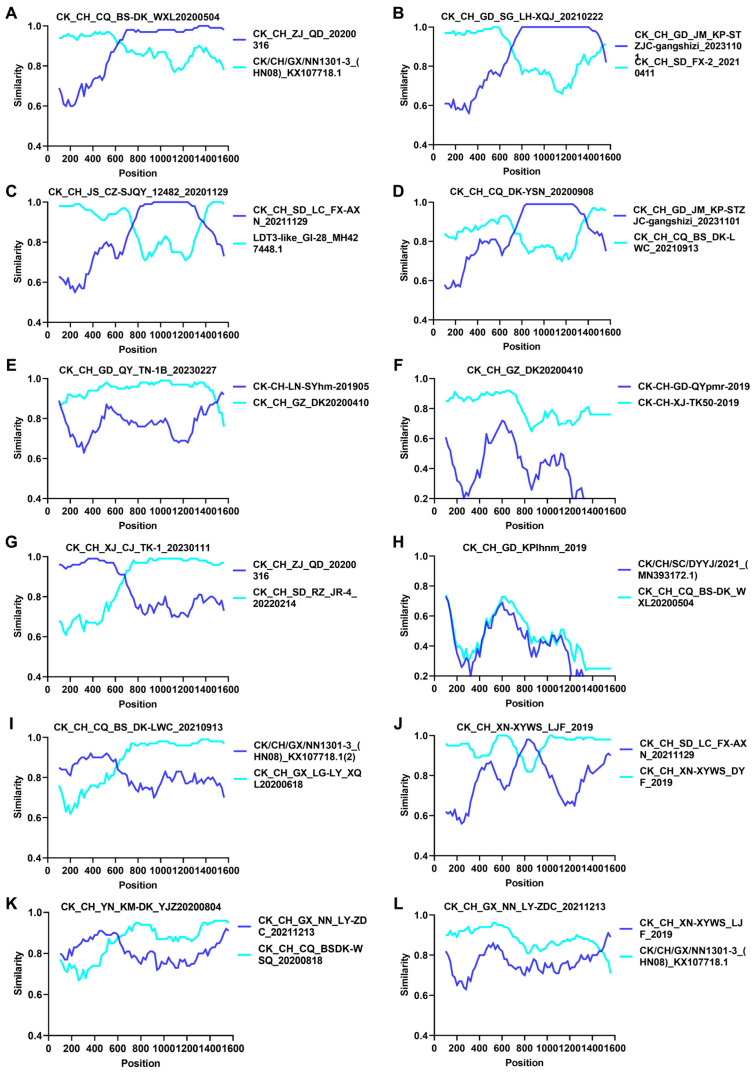
Analysis of the recombination events in the S1 gene of different variants. Determination of the potential recombination events in 12 isolates of four variant-lineages by the SimPlot analysis. These isolates are: (**A**) CK CH CQ BS-DK_WXL20200504, (**B**) CK CH GD SG LH-XQJ_20210222, (**C**) CK CH JS CZ-SJQY 12482-20201129, (**D**) CK CH CQ DK-YSN 20200908, (**E**) CK CH GD QY TN-1B 20230227, (**F**) CKCH GZ DK20200410, (**G**) CK CH XJ CJ TK-1 20230111, (**H**) CK CH GD KPlhnm 2019, (**I**) CK CH CQ BS DK-LWC 20210913, (**J**) CK CH XN-XYWS-LJF 2019, (**K**) CK CH YN KM-DK YJZ20200804, and (**L**) CK CH GX NN LY-ZDC 20211213. The *y*-axis represents the ratio of identity within a 200 bp wide sliding window centered on the position plotted, with a 20 bp step size between plots.

Determination of the potential recombination events and breakpoints in these four variant-lineages by SimPlot analysis showed that the occurrence rates of gene recombination events were 83.33% (10/12), with lineages GI-19, GI-22, GI-28, GI-13-like, and GI-19-like identified as major parental strains, and GI-7, GI-13, GI-19, GI-22, GI-19-like, and GI-22-like as minor parental strains, respectively, in ten events (Figure 3A–E,G,J–L). It suggests that gene recombination may serve as a primary factor contributing to the emergence of these variants.

**Figure 4:**
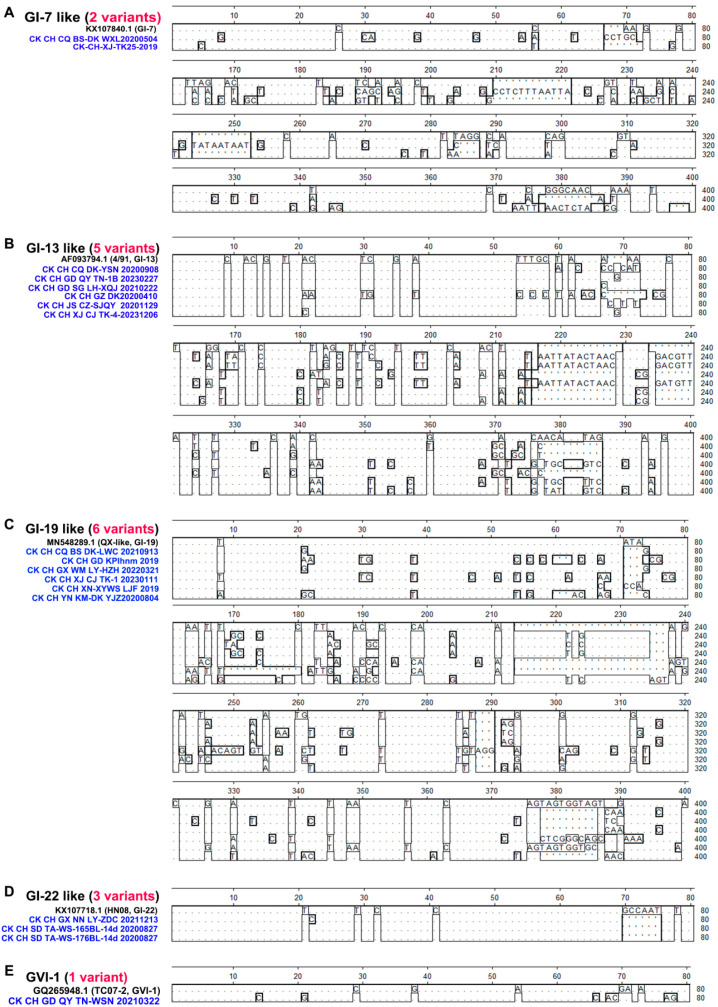
Deletion and insertion events in the S1 gene of 17 variants. Megalign analysis was performed to detect gene deletion and insertion in the S1 from 17 variants in 5 variant-lineages, including GI-7-like (**A**), GI-13-like (**B**), GI-19-like (**C**), GI-22-like (**D**), and GVI-1-like (**E**).

Deletion and insertion events of five variant-lineages were determined by Megalign analysis. Compared to the reference strains GI-7, GI-13, GI-19, GI-22, and GVI-1, variants belonging to the GI-7-like, GI-13-like, and GI-19-like lineages exhibited a significantly higher frequency (ranging from 67 to 400) of deletion and insertion events in the S1 gene (Figure 4). Among variants in the GI-7-like lineage, two distinct variants displayed a characteristic three-base deletion (AGG) at positions 285–287, and variants in the GI-22-like lineage featured a distinctive five-base deletion (GCCATT) at positions 70–75 (Figure 4). No specific mutations or insertions were detected in the S1 gene of variants in the GI-13-like, GI-19-like, and GVI-1-like lineages (Figure 4). These results suggest that deletion and insertion events are also significant factors contributing to the emergence of these variants.

### 3.4. Homology Analysis of Six Representative Isolates with the Vaccine Strain H120 and Their Growth Kinetics and Pathogenicity

**Figure 5:**
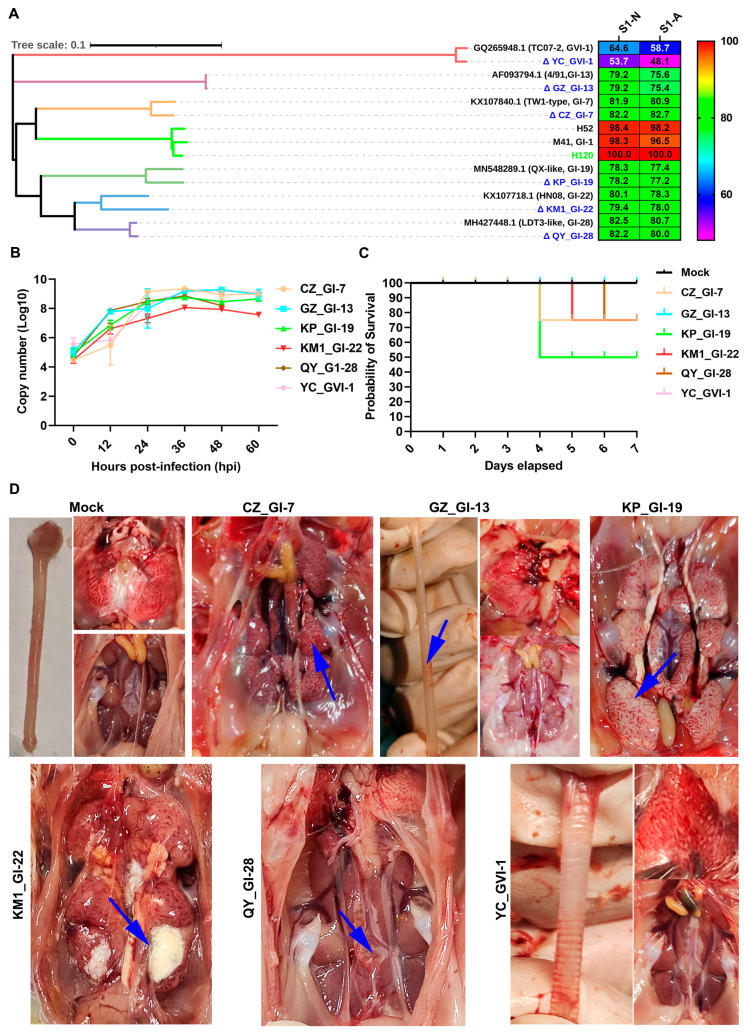
Homology, growth kinetics, and pathogenicity analyses of six representative isolates. (**A**) Two software programs, MEGA 7.0 and Megalign, were successively used to analyze the homology of S1 sequences between the vaccine strain H120 and six representative isolates with different lineages. S1-N and S1-A represent homology of nucleic acid and amino acid sequences, respectively. (**B**) Growth curves of six representative isolates in chicken embryos. Nine-day-old chicken embryos were infected at an EID_50_ ~10^3,^ respectively. The copy numbers of IBV in allantoic fluids were determined and calculated by RT-qPCR. (**C**) Surviving curves of six representative isolates in 1-day-old SPF chickens. Chickens were infected with ~10^5.5^ EID_50_ each of the six representative isolates by the nasal–ocular route, respectively. The number of deaths occurring within 7 days after the challenge was recorded, and a survival curve was generated using the Graphpad Prism 9 program. (**D**) Examination of pathological lesions in the trachea, lung, and kidney autopsies. At 7 days post-challenge, the trachea, lung, and kidney autopsies of dead chickens or chickens randomly selected from chickens infected with the six representative isolates were examined macroscopically. Blue arrows indicate the pathological lesions observed.

Based on the phylogenetic analysis, six representative isolates, CZ_GI-7, GZ_GI-13, KP_GI-19, KM-1_GI-22, QY_GI-28, and YC_GVI-1, from each of the six distinct lineages (GI-7, GI-13, GI-19, GI-22, GI-28, and GVI-1) were randomly selected to investigate their variations in the growth characteristics and pathogenicity among different lineages of IBV strains. Homology analysis of S1 nucleotide (S1-N) sequences between these six isolates and vaccine strain H120 demonstrated 82.2% (CZ_GI-7), 79.2% (GZ_GI-13), 78.2% (KP_GI-19), 79.4% (KM-1_GI-22), 82.2% (QY_GI-28), and 53.7% (YC_GVI-1), respectively, nucleotide sequence homology with H120, and even lower amino acid sequence homology (S1-A) (Figure 5A). It revealed a low homology between the main immunogenic S1 genes from the six prevalent strains of different lineages and H120. This low homology might be a significant factor contributing to the suboptimal immune efficacy or even failure of the current immunization campaign.

The EID_50_ of these isolates was determined in chicken embryos. As shown in Table 2, CZ_GI-7, GZ_GI-13, KP_GI-19, KM-1_GI-22, and QY_GI-28 reached their peak EID_50_ of 10^6.5^, 10^4.67^, 10^5^, 10^4.5^, and 10^5^, respectively, at 72 h post-inoculation. The pathogenicity of YC_GVI-1 to chicken embryos was limited, making it difficult to determine the EID50 value for this isolate (Table 2).

Their growth curves in chicken embryos were then determined by infecting 9-day-old chicken embryos at 10^3^ EID_50_, harvested at 12, 24, 36, 48, and 60 h post-inoculation, respectively. Total RNA was extracted and viral RNA copy numbers were determined by RT-qPCR, showing markedly different growth characteristics for these isolates. Among them, CZ_GI-7 reached the highest peak copy numbers (~10^9^) at 24 h post-inoculation, and KM-1_GI-22 the lowest peak copy numbers (~10^7^) at 36 h post-inoculation (Figure 5B).

The mortality rates of these isolates were evaluated within 7 days post-challenge with ~10^5.5^ EID_50_ in viral allantoic fluid. Chickens in the mock, YC_GVI-1-, and GZ_GI-13-infected groups showed no obvious clinical symptoms and death (Figure 4C). One chicken (20%) in the CZ_GI-7, three (60%) in the KP_GI-19, one (20%) in the KM-1_GI-22, and one (20%) in the QY_GI-28 group displayed pronounced symptoms and illness (Figure 5C).

The autopsy results of the dead chickens in the CZ_GI-7, KP_GI-19, KM-1_GI-22, and QY_GI-28 groups showed no visible pathological changes in the trachea and lungs, but with urate deposition in the kidneys of all infected birds. Among them, severe urate deposition and typical nephritis of swollen and pale kidneys were observed in chickens infected with CZ_GI-7, KP_GI-19, and KM-1_GI-22 (Figure 5D). As all chickens in the YC_GVI-1- and GZ_GI-13 groups showed no obvious clinical symptoms and death within 7 days after challenge, three chickens each in these two groups were randomly euthanized and necropsied at 7 days post-challenge, showing no obvious macroscopic lesions in lungs and kidneys, but mild tracheal hemorrhages in chickens infected with GI-13 (Figure 5D).

### 3.5. Histopathological Characterization of Chickens Infected with the Six Representative Isolates

**Figure 6:**
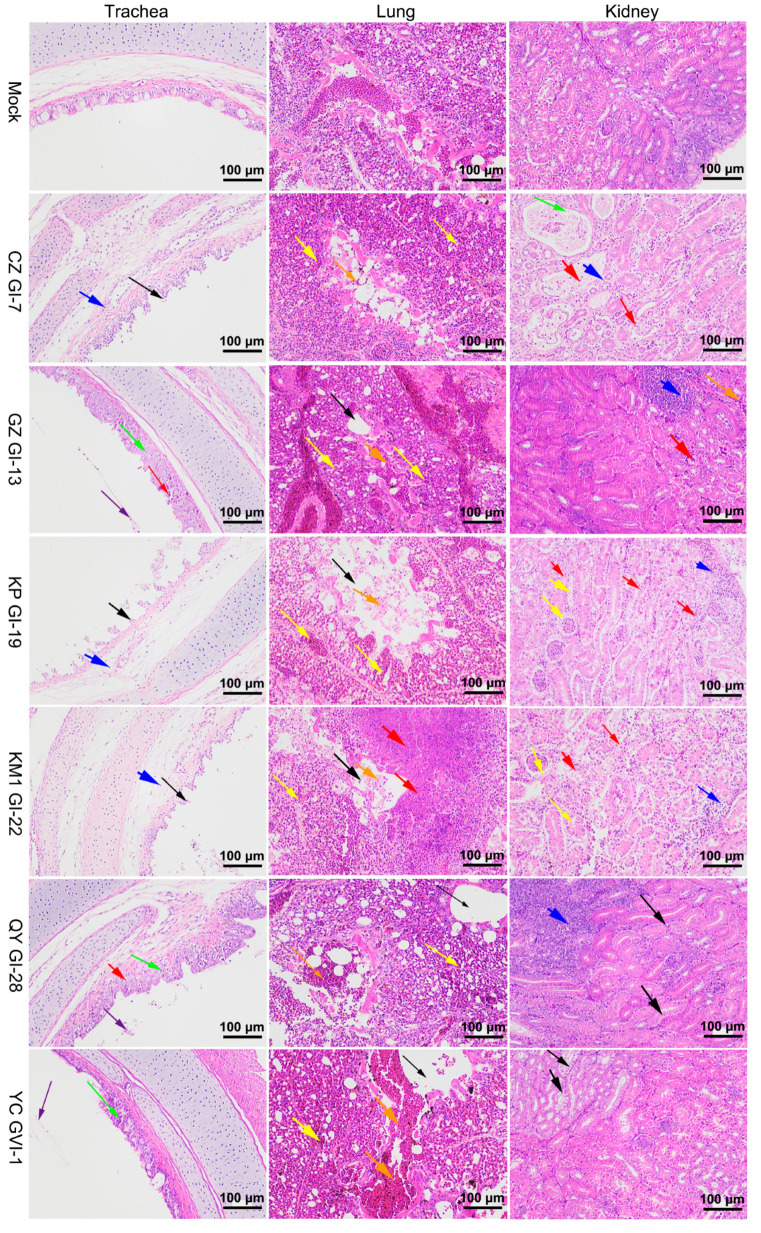
Histopathological examination of the trachea, lungs, and kidneys in chickens infected with each of the six representative isolates. At 7 days post-challenge, the trachea, lung, and kidney autopsies of dead chickens or chickens randomly selected from each experimental group were conducted and examined by microscopy.

Further evaluation of tissue damage by routine HE sections was conducted to investigate the pathological features of these isolates and to confirm the macroscopic lesions observed. Compared to the control group, chickens in the CZ_GI-7, KP_GI-19, and KM1_GI-22 groups displayed extensive necrosis and exfoliation of mucosal epithelial cells in the trachea (black arrows), along with moderate lamina propria edema, loose connective tissue arrangement, and a small amount of lymphocyte infiltration (blue arrows) (Figure 6). Chickens in the GZ_GI-13, QY_GVI-28, and YC_GVI-1 groups showed irregularly arranged tracheal cilia, slightly thickened tracheal mucosa (green arrows), and the presence of cell debris in the tracheal cavity (purple arrows) (Figure 6). Some necrosis of mucosal epithelial cells was also found in chickens in the GZ_GI-13 and QY_GVI-28 groups (red arrows) (Figure 6).

Chickens in the CZ_GI-7, GZ_GI-13, KP_GI-19, and KM1_GI-22 groups exhibited irregular arrangement of glomeruli and renal tubules in the kidneys, with a significant number of renal tubular epithelial cells that were necrotic and exfoliated (red arrows), and, to a lesser extent, localized lymphocyte infiltration (blue arrows) (Figure 6). Renal tubular expansion was also specifically detected in chickens infected with CZ_GI-7 (green arrows) (Figure 6). In contrast, chickens in the QY_GVI-28 and YC_GVI-1 groups showed minimal renal tubular epithelium degeneration (black arrows), along with focal lymphocyte infiltration within the interstitium only in the QY_GVI-28 group (blue arrows) (Figure 6).

Chickens in the GZ_GI-13, KP_GI-19, and KM1_GI-22 groups displayed widespread severe bronchiole and lung chamber dilation (black arrows), numerous necrotic-cell-fragment-containing bronchial cavities (orange arrows), interstitial vessels and capillary congestion (yellow arrows), and necrotic foci were detected in the lung tissues of the KM1_GI-22 group (red arrows) (Figure 6). In addition, mild bronchiectasis (black arrows), bronchial hemorrhage (orange arrows), and congestion of interstitial vessels and capillaries (yellow arrows) were observed infrequently in the lung tissues of chickens in the CZ_GI-7, QY_GI-28, and YC_GVI-1 groups (Figure 6).

### 3.6. Determination of Viral Loads in Different Organs in Chickens Infected with the Six Representative Isolates

**Figure 7:**
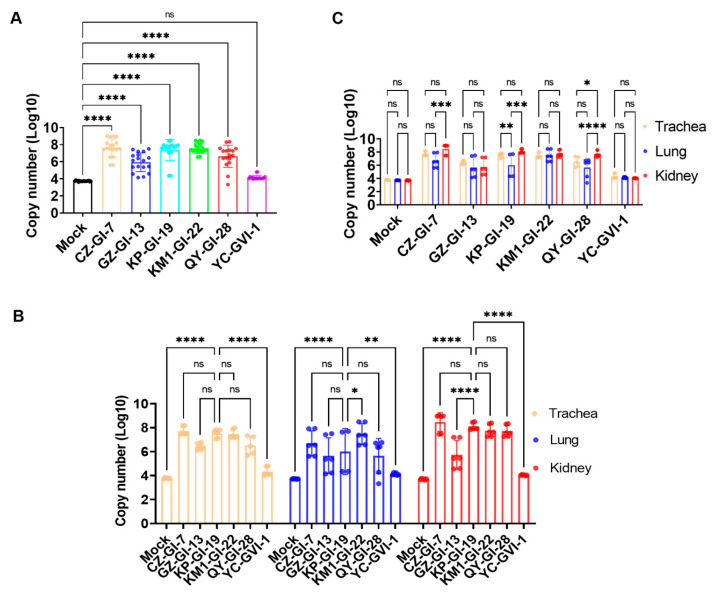
Viral loads in the trachea, lung, and kidney tissues from chickens infected with six representative isolates. At 7 days post-challenge, three chickens from each experimental group were randomly selected, the trachea, lung, and kidney tissues were collected, and the viral RNA copy numbers were determined and calculated by RT-qPCR. Significance levels are presented by the *p*-value (ns, non-significant; * *p* < 0.05; ** *p* < 0.01; *** *p* < 0.001; and **** *p* < 0.0001). (**A**) Statistical analysis of the total viral loads in the three tissues within each challenging group and the control group. (**B**) Statistical analysis of viral load variations in different tissues from chickens challenged with six representative isolates. (**C**) Statistical analysis of viral load variations in the same organs from chickens challenged with the other five isolates and the predominant lineage strain KP_GI-19, respectively.

Viral loads in the trachea, lungs, and kidneys from three randomly selected chickens in each group were assessed at 7 days post-challenge (Figure 7A). The average viral loads in all three organs were first determined by RT-qPCR. With the exception of YC_GV-1, significant higher viral loads (*p* < 0.0001) were detected in chickens infected with the other five isolates than in the control group. The average viral loads in all three organs as well as in individual organs from chickens infected with YC_GVI-1 were at similar levels as in the control group, with no statistical difference between the infected and control groups, indicating that this isolate is a naturally occurring avirulent strain, and it was excluded in the following analysis.

The viral loads in the kidneys of the infected chickens were determined and calculated (Figure 7B). Very similar levels of average viral loads in the CZ_GI-7, KM1_GI-22, and QY_GI-28 groups as those in chickens infected with the predominant lineage representative strain KP_GI-19 were detected, suggesting that these three strains were nephropathogenic. The average viral loads in the GZ_GI-13 group were significantly lower than those in the KP_GI-19 group (**** *p* < 0.0001). KM1_GI-22 displayed a significantly higher viral load in the lungs (* *p* < 0.05), but no significant difference was observed in the trachea from chickens infected with the five isolates.

Comparison of the viral loads in the three organs infected with individual isolates was then conducted (Figure 7C), showing that viral loads in the kidneys of the QY_GI-28 group were significantly higher than those in both trachea (* *p* < 0.05) and lungs (**** *p* < 0.0001). Viral loads in both kidneys (*** *p* < 0.001) and trachea (** *p* < 0.01) of the KP_GI-19 group were significantly higher than in the lungs. No significant differences were observed in chickens infected with GZ_GI-13, KM1_GI-22, and QY_GVI-1, respectively, in all three organs. These findings further support the strong renal tropism of CZ_GI-7, QY_GI-28, and KP_GI-19.

**Figure 8:**
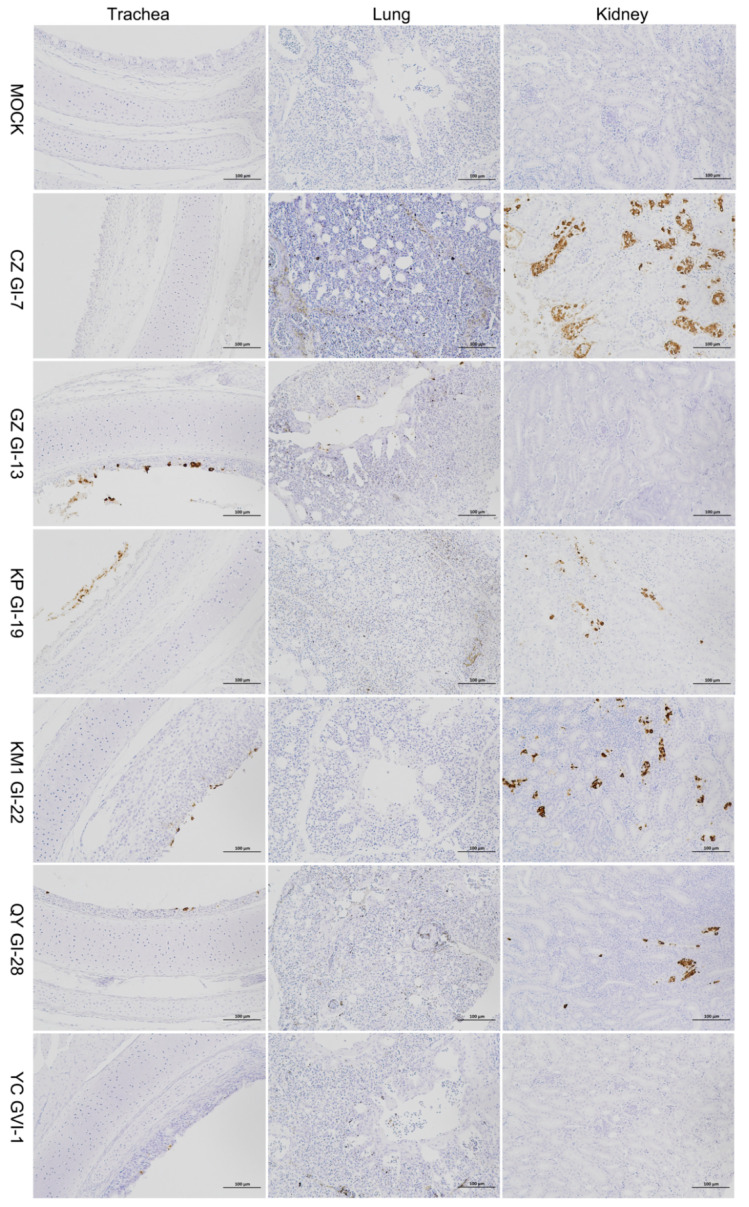
Immunohistochemical examination of IBV replication in the trachea, lungs, and kidneys from chickens infected with six representative isolates. At 7 days post-challenge, the trachea, lung, and kidney autopsies of dead chickens or chickens randomly selected were conducted, and lung and kidney sections were immunohistochemically stained with a monoclonal antibody against IBV N protein.

Immunohistochemistry results (Figure 8) demonstrated that isolates CZ_GI-7, KM1_GI-22, and QY_GI-28 had a similar significant renal tropism as KP_GI-19, and also exhibited a broad tissue tropism to the trachea and lung tissues. GZ_GI-13 displayed a limited tropism to the trachea and lung tissues, with no associated virions detected in the kidneys. Careful observation also revealed that the main cell types primarily infected by these isolates were the tracheal mucosal epithelial cells, the bronchial and parabronchial epithelial cells in the lungs, and the renal tubular epithelial cells. These findings further support the classification of CZ_GI-7, KM1_GI-22, QY_GI-28, and KP_GI-19 as nephropathogenic strains, GZ_GI-13 a respiratory type, and YC_GVI-1 an avirulent strain.

## 4. Discussion

The co-existence of multiple genotypes of IBV strains with varying antigenicity and pathogenicity in many countries challenges the current surveillance and prevention strategies. The current classification scheme classifies IBV into seven genotypes (GI-GVII) and a total of 35 distinct lineages [8]. QX-type (GI-19) strains are the most prevalent in six Asian countries, including Japan, Korea, India, Indonesia, Thailand, and China, and 793B-type (GI-13) strains are the second most prevalent lineage in these countries except Korea [21]. However, during 2013–2015, 89.8% isolates were the QX-type (GI-19), TW I-type (GI-7), and 4/91-type (GI-13) [21], with the QX-type (GI-19) as the most common isolates (46.7%) in China [22]. In 2016–2018, an epidemiological survey conducted in Henan, Hubei, and Hunan provinces showed that the prevalence of GI-19 (QX) was 48.2% in 56 isolates [23]. The GVI-1 (TC07-2) lineage was first detected in China in 2007 [24], and has significantly increased in recent years. This lineage is currently prevalent in more than five countries, including China, Colombia, Japan, Korea, and Vietnam [25]. In this study, we report the isolation and identification of seven predominant lineages and five variant-lineages circulating in China over the past five years. The GI-19 (QX or LX4) lineage has emerged as the most prevalent strain, accounting for 61.77 ± 4.56%, and the GVI-1 lineage as the second, accounting for 6.88 ± 1.84%, supporting that the GI-19 (QX-type) lineage has become the dominant strain in China. These findings are largely consistent with previous reports. It is worth noting that GVI-1 genotype (TC07-2) strains have been isolated and identified, in this study, in Sichuan in Southwest China, Liaoning in Northeast China, Jiangsu in East China, and Shandong in North China, apart from isolates in South China. This reveals a nationwide epidemic trend of GVI-1 in China. As the existing vaccines may provide poor immune protection against GVI-1 [26], these findings highlight the pressing needs to update IBV vaccine strains and develop novel broad-spectrum vaccines.

The coronavirus replicase gene contains an ExoN domain in non-structural protein (nsp) 14, playing a crucial role in proofreading and repair activities. Despite this unique feature, the estimated substitution rate of IBV genome at 10−4–10−5 substitutions/site/year remains remarkably high [27], ensuring the introduction of mutations and the emergence of multiple variants. A more important force that may drive the continuous evolution of IBV and emergence of variants would be through recombination [28]. In recent years, isolation and identification of variants have been increasingly reported [16,29,30]. In this study, potential recombination events showed an incidence rate of 66.67% (12/18). A novel recombinant strain of IBV was reported to emerge from three attenuated live vaccine strains (H120, 4/91, and LDT3-A) [31], but no such recombination events were found from any of the 205 isolates in this study. Variants of GI-7-like, GI-13-like, G-19-like, and GI-22-like are most likely generated via genetic recombination, deletion, and insertion. On the other hand, the GI-1 and GI-28 lineages exhibited remarkable stability, with no associated mutations detected across all isolates. Isolates in the two lineages also share highly conserved furin motif sequences (RRFRR). The sustained stability of the GI-1 lineage under the pressure of widespread use of vaccines based on the same GI-1 lineage (H120 and H52 strains) highlights the continuous usefulness of these vaccines for preventing and controlling outbreaks caused by IBV strains of the GI-1 lineage. Meanwhile, the relative stability of GI-28 and the emergence of multiple other lineages and variants would call for the development of novel bi- and multivalent vaccines.

Furin, a proprotein convertase, is located in the trans-Golgi network and activated by acidic PH. Furin can cleave precursor proteins with specific motifs to produce mature proteins with biological activity [32]. Cleavage of the S protein by furin is thought to play an important role in promoting productive IBV infection in cultured cells [33]. In this study, the furin motif sequences of each genotype were analyzed systematically, revealing some relatively conserved patterns: RR(F/S)RR for GI-7, (R/H)R(S/R)RR for GI-13, (H/R)R(R/F)(R/K)R for GI-13-like, (H/R)R(R/H/F)RR for GI-19, (H/R)R(R/H)(R/K)R for GI-19-like, (H/R)R(R/F/L)(K/R)R for GI-22, and RRFRR for both GI-1 and GI-28. The furin motif, R-X-X-R↓X (X: any amino acid; ↓: cleavage site), displays a significant level of conservation. Interestingly, we find that the furin cleavage site in the S protein of three out of four nephropathogenic isolates (CZ_GI-7, KM1_GI-22, and QY_GI-28) is RRFRR, exhibiting renal tropism with urate deposition. The same motif in GZ_GI-13, an isolate with strong tracheal tropism, is RRSRR. As this motif contains physical properties critical for furin cleavage and S protein-mediated fusion efficiency [34], this sequence difference may play a role in determining the tissue tropism and pathogenicity of IBV. In addition, the relative abundance of furin proteases in different tissues and cells may also determine the IBV tissue and cell tropism. Cellular furin abundance has been strongly associated with the susceptibility of cells to IBV infection [33,35]. Subtle variations in furin expression across tissues and cell types may account for differential susceptibility to IBV infection, despite its ubiquitous expression [36,37]. Further studies are warranted to clarify these issues.

The virulence and mortality rates of prevalent IBV isolates in different lineages were reported to vary markedly. Some QX-like strains recently isolated in China exhibited a mortality rate ranging from 10% to 50% among infected chickens [38,39]. As observed in this study, the up to 60% fatality rate in chickens infected with the KP_GI-19 strain is significantly higher than the approximate 20% fatality rate in chickens infected by other prevalent strains of different lineages (CZ_GI-7, KM1_GI-22, and QY_GI-28). Chickens infected with the two attenuated strains (GZ_GI-13 and YC_GVI-1) showed no mortality. However, it was recently reported that the pathogenicity of an isolate of the GVI-1 lineage (SX/2204) was significantly increased, with a mortality rate up to 60%, compared to previously isolated GVI-1 strains [25]. It appears that the pathogenicity of isolates of the GI-19 and GVI-1 lineages exhibit an upward trend, as evidenced by this and several other recent studies.

Although the clinical and pathological outcomes of IBV infection are heavily influenced by the infecting strains, it is noteworthy that multiple physiological systems can be concurrently infected. The target organs susceptible to IBV infection encompass mainly the trachea, lung, kidney, oviduct, and gut [3,40]. In this study, immunohistochemistry results were consistent with their viral loads in the three organs, and we showed that four out of six representative isolates (CZ_GI-7, KP_GI-19, KM1_GI-22, and QY_GI-28) could infect the tracheal, lung, and renal tissues of SPF chickens, with typical ‘macular nephropathy’ pathological changes in the kidney autopsy. However, the other two isolates (GZ_GI-13 and YC_GVI-1) exhibited no to mild clinical symptoms and pathological alterations. Our results support that GI-19 is more pathogenic than other genotypes, but variation in virulence was also noted in the same lineage.

In summary, this study has demonstrated that the GI-19 lineage is currently the predominant IBV strain, with high pathogenicity and an escalating mortality rate. The prevalence of this lineage and the second most prevalent strains of the GVI-1 lineage, along with their related variants, are steadily increasing, and the primary driving force for the emergence of variants is by genetic recombination. These observations urge the development of novel bi- and multivalent vaccines targeting the prevalent strains of the GI-19 and GVI-1 lineages. In addition, the possibility of developing the naturally occurring avirulent YC_GVI-1 strain as a live attenuated vaccine warrants further investigation to assess its immunogenicity and protection efficacy.

## Figures and Tables

**Table 1:** RDP v.4.100 recombination analysis of S1 of variant isolates.

Recombinant Isolates	Breakpoint Positions		Minor Parent ^3^	Major Parent ^4^	RDP ^6^
	End	Begin			
CK CH CQ BS-DK WXL20200504	1239 *	605	CK CH ZJ QD 20200316	CK/CH/GX/NN1301-3 (HN08) KX107718.1	6.38 × 10^−14^
CK CH GD SG LH-XQJ 20210222	1516	666	CK CH GD JM KP-STZJC-gangshizi 20231101	CK CH SD FX-2 20210411	2.91 × 10^−46^
CK CH JS CZ-SJQY12482 20201129	1358	723	CK CH SD LC FX-AXN 20211129	LDT3-like_GI-28 MH427448.1	4.69 × 10^−43^
^CK CH CQ DK-YSN 202009082	1370	714	CK CH GD JM KP-STZJC-gangshizi 20231101	CK CH CQ BS DK-LWC 20210913	1.22 × 10^−32^
^CK CH GD QY TN-1B 202302272	94	1475 *	CK-CH-LN-SYhm-201905	CK CH GZ DK20200410	4.65 × 10^−13^
CK CH GZ DK20200410	76	18 *	Unknown (CK-CH-GD-QYpmr-2019) ^5^	CK-CH-XJ-TK50-2019	4.11 × 10^−5^
CK CH XJ CJ TK-1 20230111	666	28	CK CH ZJ QD 20200316	CK CH SD RZ JR-4 20220214	1.33 × 10^−32^
CK CH GD_KPlhnm_2019	1653	1040	CK/CH/SC/DYYJ/2021 (MN393172.1)	CK CH CQ BS-DK WXL20200504	5.03 × 10^−26^
CK CH CQ BS DK-LWC 20210913	646	46	CK/CH/GX/NN1301-3 (HN08) KX107718.1	CK CH GX LG-LY XQL20200618	2.94 × 10^−12^
CK CH XN-XYWS LJF_ 019	463 *	355	CK CH SD LC FX-AXN 20211129	CK CH XN-XYWS DYF 2019	3.25 × 10^−12^
CK CH YN KM-DK YJZ20200804	666	159 *	CK CH GX NN LY-ZDC 20211213	CK CH CQ BSDK-WSQ 20200818	5.28 × 10^−4^
CK CH GX NN LY-ZDC 20211213	36	1494	CK CH XN-XYWS LJF 2019	CK CH GX NN1301-3 (HN08) KX107718.1	6.05 × 10^−11^

* The actual breakpoint position is undetermined (most likely overprinted by a subsequent recombination event). ^ The recombinant sequence may have been misidentified (one of the identified parents might be the recombinant). ^3^ Minor parent: parent contributing to a small fraction of sequences. ^4^ Major parent: parent contributing to a large fraction of sequences. ^5^ Unknown: only one parent and a recombinant need to be in the alignment for a recombination event to be detectable, and the sequences listed as unknown were used to infer the existence of a missing parental sequence. ^6^ *p*-value: *p*-value of RDP method.

**Table 2:** EID_50_ of the six representative isolates.

Isolates	Name	Genotype	EID_50_
CK CH CZLH PGZ 20201221	CZ_CI-7	GI-7	10^6.5^
CK CH GD KP LH-NF 20220317	KP_GI-19	GI-19	10^5^
CK CH YN KM I2 20210506 1	KM1_GI-22	GI-22	10^4.5^
CK CH GD QY HHHH-60d 20210616	QY_GI-28	GI-28	10^5^
CK CH SC YC-DK LMB 20210104	YC_GVI-1	GVI-1	-

## Data Availability

The data presented in this study are available on request from the corresponding author.

## Supplementary Materials

The following supporting information can be downloaded at: https://www.mdpi.com/article/10.3390/v16060930/s1, Supplementary File: Sequences of 205 isolates.

## References

[1] Wang Y., Grunewald M., Perlman S. (2020). Coronaviruses: An Updated Overview of Their Replication and Pathogenesis. Methods Mol. Biol..

[2] Fung T.S., Liu D.X. (2019). Human Coronavirus: Host-Pathogen Interaction. Annu. Rev. Microbiol..

[3] Hoerr F.J. (2021). The Pathology of Infectious Bronchitis. Avian Dis..

[4] Feng K., Xue Y., Wang F., Chen F., Shu D., Xie Q. (2014). Analysis of S1 gene of avian infectious bronchitis virus isolated in southern China during 2011–2012. Virus Genes.

[5] Liu S., Kong X. (2004). A new genotype of nephropathogenic infectious bronchitis virus circulating in vaccinated and non-vaccinated flocks in China. Avian Pathol..

[6] Cavanagh D., Davis P.J., Mockett A.P. (1988). Amino acids within hypervariable region 1 of avian coronavirus IBV (Massachusetts serotype) spike glycoprotein are associated with neutralization epitopes. Virus Res..

[7] Valastro V., Holmes E.C., Britton P., Fusaro A., Jackwood M.W., Cattoli G., Monne I. (2016). S1 gene-based phylogeny of infectious bronchitis virus: An attempt to harmonize virus classification. Infect. Genet. Evol..

[8] Houta M.H., Hassan K.E., El-Sawah A.A., Elkady M.F., Kilany W.H., Ali A., Abdel-Moneim A.S. (2021). The emergence, evolution and spread of infectious bronchitis virus genotype GI-23. Arch. Virol..

[9] Feng K., Xue Y., Wang J., Chen W., Chen F., Bi Y., Xie Q. (2015). Development and efficacy of a novel live-attenuated QX-like nephropathogenic infectious bronchitis virus vaccine in China. Vaccine.

[10] Yan S., Sun Y., Huang X., Jia W., Xie D., Zhang G. (2019). Molecular characteristics and pathogenicity analysis of QX-like avian infectious bronchitis virus isolated in China in 2017 and 2018. Poult. Sci..

[11] Ren G., Liu F., Huang M., Li L., Shang H., Liang M., Luo Q., Chen R. (2020). Pathogenicity of a QX-like avian infectious bronchitis virus isolated in China. Poult. Sci..

[12] Cavanagh D. (2003). Severe acute respiratory syndrome vaccine development: Experiences of vaccination against avian infectious bronchitis coronavirus. Avian Pathol..

[13] Alvarado I.R., Villegas P., Mossos N., Jackwood M.W. (2005). Molecular characterization of avian infectious bronchitis virus strains isolated in Colombia during 2003. Avian Dis..

[14] Jackwood M.W., Hall D., Handel A. (2012). Molecular evolution and emergence of avian gammacoronaviruses. Infect. Genet. Evol..

[15] Ting X., Xiang C., Liu D.X., Chen R. (2022). Establishment and Cross-Protection Efficacy of a Recombinant Avian Gammacoronavirus Infectious Bronchitis Virus Harboring a Chimeric S1 Subunit. Front. Microbiol..

[16] Xia J., He X., Yao K., Du L., Liu P., Yan Q., Wen Y., Cao S., Han X., Huang Y. (2016). Phylogenetic and antigenic analysis of avian infectious bronchitis virus in southwestern China, 2012–2016. Infect. Genet. Evol..

[17] Ali A., Ojkic D., Elshafiee E.A., Shany S., El-Safty M.M., Shalaby A.A., Abdul-Careem M.F. (2022). Genotyping and In Silico Analysis of Delmarva (DMV/1639) Infectious Bronchitis Virus (IBV) Spike 1 (S1) Glycoprotein. Genes.

[18] Yamada Y., Liu D.X. (2009). Proteolytic activation of the spike protein at a novel RRRR/S motif is implicated in furin-dependent entry, syncytium formation, and infectivity of coronavirus infectious bronchitis virus in cultured cells. J. Virol..

[19] Ramakrishnan M.A. (2016). Determination of 50% endpoint titer using a simple formula. World J. Virol..

[20] Oumarou H.H., Aboudharam G., Barbieri R., Lepidi H., Drancourt M. (2022). Immunohistochemical diagnosis of human infectious diseases: A review. Diagn. Pathol..

[21] Zhao J., Zhao Y., Zhang G. (2023). Key Aspects of Coronavirus Avian Infectious Bronchitis Virus. Pathogens.

[22] Feng K., Wang F., Xue Y., Zhou Q., Chen F., Bi Y., Xie Q. (2017). Epidemiology and characterization of avian infectious bronchitis virus strains circulating in southern China during the period from 2013–2015. Sci. Rep..

[23] Ji J., Gao Y., Chen Q., Wu Q., Xu X., Kan Y., Yao L., Bi Y., Xie Q. (2020). Epidemiological investigation of avian infectious bronchitis and locally determined genotype diversity in central China: A 2016–2018 study. Poult. Sci..

[24] Li L., Xue C., Chen F., Qin J., Xie Q., Bi Y., Cao Y. (2010). Isolation and genetic analysis revealed no predominant new strains of avian infectious bronchitis virus circulating in South China during 2004–2008. Vet. Microbiol..

[25] Yang Y., Wang D., Bai Y., Huang W., Gao S., Wu X., Wang Y., Ren J., He J., Jin L. (2023). Genetic and pathogenic characterization of new infectious bronchitis virus strains in the GVI-1 and GI-19 lineages isolated in central China. J. Integr. Agric..

[26] Chen L., Xiang B., Hong Y., Li Q., Du H., Lin Q., Liao M., Ren T., Xu C. (2021). Phylogenetic analysis of infectious bronchitis virus circulating in southern China in 2016-2017 and evaluation of an attenuated strain as a vaccine candidate. Arch. Virol..

[27] Franzo G., Massi P., Tucciarone C.M., Barbieri I., Tosi G., Fiorentini L., Ciccozzi M., Lavazza A., Cecchinato M., Moreno A. (2017). Think globally, act locally: Phylodynamic reconstruction of infectious bronchitis virus (IBV) QX genotype (GI-19 lineage) reveals different population dynamics and spreading patterns when evaluated on different epidemiological scales. PLoS ONE.

[28] Yuan S., Cheng Q., Guo J., Li Z., Yang J., Wang C., Liang Z., Zhang X., Yu H., Li Y. (2022). Detection and genetic characterization of novel infectious bronchitis viruses from recent outbreaks in broiler and layer chicken flocks in southern China, 2021. Poult. Sci..

[29] Sjaak D.W.J., Cook J.K., van der Heijden H.M. (2011). Infectious bronchitis virus variants: A review of the history, current situation and control measures. Avian Pathol..

[30] Lin S., Chen H. (2017). Infectious Bronchitis Virus Variants: Molecular Analysis and Pathogenicity Investigation. Int. J. Mol. Sci..

[31] Gong H., Ni R., Qiu R., Wang F., Yan W., Wang K., Li H., Fu X., Chen L., Lei C. (2022). Evaluation of a novel recombinant strain of infectious bronchitis virus emerged from three attenuated live vaccine strains. Microb. Pathog..

[32] Wu C., Zheng M., Yang Y., Gu X., Yang K., Li M., Liu Y., Zhang Q., Zhang P., Wang Y. (2020). Furin: A Potential Therapeutic Target for COVID-19. iScience.

[33] Tay F.P., Huang M., Wang L., Yamada Y., Liu D.X. (2012). Characterization of cellular furin content as a potential factor determining the susceptibility of cultured human and animal cells to coronavirus infectious bronchitis virus infection. Virology.

[34] Tian S. (2009). A 20 Residues Motif Delineates the Furin Cleavage Site and its Physical Properties May Influence Viral Fusion. Biochem. Insights.

[35] Chen H.Y., Guo A.Z., Peng B., Zhang M.F., Guo H.Y., Chen H.C. (2007). Infection of HeLa cells by avian infectious bronchitis virus is dependent on cell status. Avian Pathol..

[36] Molloy S.S., Thomas L., Van Slyke J.K., Stenberg P.E., Thomas G. (1994). Intracellular trafficking and activation of the furin proprotein convertase: Localization to the TGN and recycling from the cell surface. Embo J..

[37] Bosshart H., Humphrey J., Deignan E., Davidson J., Drazba J., Yuan L., Oorschot V., Peters P.J., Bonifacino J.S. (1994). The cytoplasmic domain mediates localization of furin to the trans-Golgi network en route to the endosomal/lysosomal system. J. Cell Biol..

[38] Zhao W., Zhao Y., Qin F. (2017). Holocene fire, vegetation, and climate dynamics inferred from charcoal and pollen record in the eastern Tibetan Plateau. J. Asian Earth Sci..

[39] Yan W., Yang Q., Huang S., Liu S., Wang K., Tang Y., Lei C., Wang H., Yang X. (2023). Insights on genetic characterization and pathogenesis of a GI-19 (QX-like) infectious bronchitis virus isolated in China. Poult. Sci..

[40] Khataby K., Kasmi Y., Souiri A., Loutfi C., Ennaji M.M., Ennaji M.M. (2020). Chapter 33—Avian Coronavirus: Case of Infectious Bronchitis Virus Pathogenesis, Diagnostic Approaches, and Phylogenetic Relationship Among Emerging Strains in Middle East and North Africa Regions. Emerging and Reemerging Viral Pathogens.

