# Supplementary material for: Prevalence, Genotype Diversity, and Distinct Pathogenicity of 205 Gammacoronavirus Infectious Bronchitis Virus Isolates in China during 2019–2023
Source: Viruses. 2024 Jun 7;16(6):930. doi: 10.3390/v16060930 (PMC11209364; doi:10.3390/v16060930)
Supplement: Supplementary file 1 [file viruses-16-00930-s001.zip › viruses-2989697-supplementary.pdf]

>CK-CH-XJ-TK25-2019.seq

ATGTCGGTGAAGTCACTGTTTTTAGTGACTCTTTTGTTTGCACTATCTAGTGCTATTTTGTATGATAATAGTTAT
GTTTATTATTATCAGAGTGCTTTTAGGCCTTCAACTGGTTGGCATTTACATGGTGGTGCTTATGCAGTAGTTAA
TGTTACTACTCACTATGCCAATGCAGGCAAAGCGTCTGTCTGTATTGGTGGTAGTATTCAAGGCGGCTATGTA
TTTAATGCTTCTTCTGTAGCTATGACCGCTCCGAATAATGGTATGACTTGGTCTACAACACAATTTTGTACTGC
ACACTGCAATTTCTCGGAAGTTACAGTGTTTGTTACACATTGTTATAATTCAACTCTAGGCGCTTGTCCTACGG
GCTTTGTTCCAAGGAATCATATACGTATTTCTGCTATGAAAAATGGTTCTCTGTTTTATAATTCAACAATTAGTG
TATCTAAATACCCTAGATTTATGTCATTTCAGTGTGTTAAAAATTTAACATCTGTCTATCTGAATGGTGACCTTG
TTTACACTTCTAATGAGACCACAGACGTTACATCTGCAGGTGTCTATTTTAAAGCCGGTGGACCTATAACTTAT
AAAATTATGAGAGAAGTTAAAGCCTTAGCTTATTTTATTAATGGTACTGCACAAGATGTTATTTTGTGTGATGA
CACACCTAGAGGTTTGCTAGCATGTCAATATAATACTGGCAATTTTTCAGATGGTTTCTATCCTTTTACTAATAG
TAGTCTAGTTAAGCAAAGGTTTGTTGTTTATCGTGAGAATAGTGTTAACACTACTCTTACTTTAACCAACTACA
CTTTTCATAATGAGACTAATGCCTCGCCTAACTCAGGTGGTGTTCATTCTATCCCAATTTATCAAACACAAACA
GCTCAGAGTGGTTATTATAATTTTAATTTATCATTTCTGAGTAGTTTTGTGTATAAAGAGTCTAATTTCATGTATG
GGTCTTACCACCCAGCATGTAATTTTAGACTAGAAACTATTAATAATGGCTTGTGGTTTAATTCACTGTCAGTC
TCATTAACTTATGGCCCCCTTCAAGGTGGGTGTAAGCAATCAGTTTTTAGCGGCAGAGCTACTTGTTGTTATG
CGTATTCCTATAACGGACCACGTAAGTGTAAAGGTGTTTATAGTGGTGAGTTATCACAAGATTTTGAATGTGG
ACTGTTGGTTTATGTTACTAAGAGTGATGGCTCTCGTATACAAACAGCCACAGAACCACCAGTTATAACTCAA
CACAATTATAATAATATTACTTTAAACACTTGTGTTGATTACAATATATATGGCAGAGTTGGCCAAGGTTTTATT
ACTAATATAACTGACTTAGCAGCTAGCCATAATTATTTAGCAGATGCTGGATTAGCAATTTTAGATACTTCAGG
TGCCATAGACACTTTCGTTGTACAAGGTGAATATGGTCTCAATTATTATAAGGTTAACCCTTGTGAAGATGTTA
ATCAGCAGTTTGTAGTGTCAGGCGGTAAGTTAGTAGGCATTCTGACTTCTCGTAATGAAACTGGTTCTCAGC
CTCTTGAAAATCAGTTTTATATTAAGTTAACTAATGGAAGCCGCCGTTTTAGACGT

>CK_CH_FJ-IB-20190821.seq

ATGTTGGAGAAGTTACTGTTTTTAGTGATCATTTTGTGTGCACTATGTAGTGCAAATTTGTTTGATGCTGATAA
TAGTTATGTGTACTACTACCAGAGTGGATTTAGACCTCCTTTAGGTTGGCACCTTTATGGTGGTGCATATGCAG
TAGAACGGTTTTTTAATGAAACCAGCAATGCAGGCTCTAGTGACTGTACTGCTGGGGCCATTGTACATAGTTT
AAATGTTACTGCAAGTGCAGTTGCGATTACTACACCTGTTAATGGCATGCATTGGTCATCTAGTAAAGGAGTG
TGTTCAATACATTGCAATTTTAGTACAATTGTTGTTTTTGTTACACATTGTTTTAAAAATAGACAAGGAATATGT
CCCTTGACAGGTAAATTAAGGGAGGGTGACATTCGTATTGGTGTTCTAGATAGTAGTGGTAATTCTATTTTTA
ATAAAACAGTTACCACTTCTAGTTATAGTAAATTTAAATCATTACATTGCGTTAACAATTTCACTTCTGTATATTT
AAATGGTGATCTTGTTTACACGTCTAATGAAACTTCAGATATTACTGGTTTTGGTGTACATTTTAAGACAGGA
GGACCTGTTACTTATAAAATTATGAAAGAACATAAGGTTCTAGCATATTTTGAAAATGGTACTGCACACGACA
TTATTTTATGTGATGACAGTCCCCGTGGTAGGTTAGCTTGTCAGTATAATACAGGCAATTTTTCTGACGGTTTG
TACCCTTTTAGCGTAAGCAGTGAAGTTAATGAAACTTTTATAGTTTTTGAAAAGAATACAGAAACTACTATGC
TTACATTAAATAATTTCACTTTTTTTAATCAGAGTGGGGCTCAACCTAATCAAAAGGAACCTTCACCTGGTGTT
TCAAATTTTGTGTATTATCAACAGATTAGTGCTGTTCCTGGTTATAATAATTTTAATTTTTCCTTTTTGAGTTCTT
TTACTTATTTAAGTAGTGATTATACGAAAGGTTCTTTTCACCCAAGTTGTACTTTTAGGCCTGAAGATATTAATA
AAAATCGCAGGTTTAATCATTTGTCTATATCCTTATCTTATGGTCCTCGTAATGGAGGCTGTAAGCAAGCATGC
TTTAATACTAGGAGTTCATGTTGTTGTTTCATGTACTCTTATAATGGTCAACCTCTTTGTAAAGGTGTGTATAGT
GGTGATTTAAATCAAGATTTTGAGTGCGTATTGCTTGTGTTTATTAATCATAGCCCAGGCAGTCGTATATTTAC
TTCTGAAACAGTACCTACTGTCACTGCTAATTTTGCAAATAATGTGGTTTTAGATAGGTGTGTTGATTATAATAT
CTATG

>CK-CH-GD-QYpmr-2019.seq

ATGTTGGAGAAGTTACTGTTTTTAGTGACCATTTTGTGTGCACTATGTAGTGCAAATTTGTTTGATGCTGATAA
TAGTTATGTGTACTACTACCAGAGTGGATTTAGACCTCCTTCAGGTTGGCACCTTTATGGTGGTGCGTATGCA
GTAGAACGGTTTTTTAATGAAACCAGCAATGCAGGCTCTGGTGACTGTACTGCTGGAGCCATTGTACATAGT
TTAAATGTTAGTGCAAGTGCAGTTGCGATTACTACACCTGTTAATGGCATGCGTTGGTCATCTAAAGGAGTAT
GTTCAATACATTGCAATTTTAGTACAATTGTTGTTTTTGTTACACATTGTTTTAAAAATGGACAAGGAATATGT
CCCTTGACGGGTAAATTAAGGGAGGGTGATATTCGTATTGGTGTTCTAGATAGTAGTGGTAATTCTATTTTTAA
TAAAACAGTTACCACTTCTAGTTATAGTAAATTTAAATCATTACATTGCGTTAACAATTTCACTTCTGTGTATTTA
AATGGTGATCTTGTTTACACGTCTAGTGAAACTTCTGATATTACTGGTTTTGGTGTACATTTTAAGACAGGAG
GACCTGTTACTTATAAAATTATGAAAGAACATAAGGTTTTAGCATATTTTGAAAATGGTACTGCACATGACATT
ATTTTATGTGATGACAGTCCCCGTGGTAGGTTAGCTTGTCAGTATAATACAGGCAATTTTTCTGACGGTTTGTA
CCCTTTTAGCATAAGCAGTGAAGTTAATGAAACTTTTGTAGTTTTTGAAAAGAATACAGAAACTACTATGCTT
ACATTAAATAATTTCACTTTTCTTAATCAGAGTGGGGCTCAACCTAATCAAAGGGAACTTTCACCTGGTGTTT
CAAATTTTGTATATTATCAACAGATTAGTGCTGTTCCTGGTTATAATAATTTTAATTTTTCTTTTTTGCGTTCTTTT
ACTTATTTAAGTAGTGATTATACGAGGGGTTCTTTTCACCCAAGTTGTACTTTTAGGCCTGAAGATATTAATAA
AAATCGCAGGTTTAATCATTTGTCTATATCCTTATCTTACGGTCCTCGTAATGGCGGCTGTAAGCAAGCATGCT
TTAATACTAAGAGTTCATGTTGTTGTTTCATGTACTCTTATAATGGTCAACCTCTTTGTAAGGGTGTGTATAGTG
GTGATTTAAATCAAGATTTTGAGTGCGTATTGCTTGTGTTTATTAAACATAGCCCAGGCAGTCGTATATTTACT
TCTGAAACAGTACCTACTGTCACTGCTAATTTTGTAAATAATGTGGTTTTAGATAGGTGTGTTGATTATAATATC
TATGTTAGTTATGGCAGGTTTTGA
>CK_CH_JS_SDL-ZBH_2019.seq
ATGTTGGTAACACCTCTTTTACTAGTGACTCTTTTGTGTGCACTATGTAGTGCTGCTTTGTATGACAGTAGTTC
TTACGTGTACTACTACCAAAGTGCCTTCAGACCACCTGATGGTTGGCATTTACATGGGGGTGCGTATGCGGTT
GTTAATATTTCTAGTGAATCTAATAATGCAGGCTCTTCATCTGTGTGTACTGTTGGTATTATTCATGGTGGTCGT
GTTGTTAATGCTTCTTCTATAGCTATGACGGCACCGTCATCAGGTATGGCTTGGTCTAGCAGTCAGTTTTGTAC
TGCATACTGTAACTTTTCAGATACTACAGTGTTTGTTACACATTGTTATAAACATGGTGGGTGTCCTATAACTG
GCATGCTTCAACAGCATTCTATACGTGTTTCTGCTATGAAAAATGGCCAGCTTTTCTATAATTTAACAGTTAGT
GTAGCTAAGTACCCTACTTTTAAATCATTTCAGTGTGTTAATAATTTAACATCCGTATATTTAAATGGTGATCTTG
TTTACACCTCTAATGCGACCACAGATGTTACATCTGCAGGTGTTTATTTTAAAGCTGGTGGACCTATAACTTAT
AAAGTTATGAGAGAAGTTAGAGCCCTGGCTTATTTTGTTAATGGTACTGCACAAGATGTTATTTTGTGTGATG
GGTCACCTAGAGGCTTGTTAGCATGCCAGTATAATACTGGCAATTTTTCAGATGGCTTTTATCCTTTTACTAAT
AGTAGTTTAGTTAAGCAGAAGTTTATTGTCTATCGTGAAAATAGTGTTAATACTACTTTTACGTTACACAATTTC
ACTTTTCATAATGAGACTGGCGCCAACCCAAATCCTAGTGGTGTCCAGAATATTCAAACTTACCAAACACAAA
CAGCTCAGAGTGGTTATTATAATTTTAATTTTTCCTTTCTGAGTAGTTTTGTTTATAAGGAGTCTAATTTTATGTA
TGGATCTTATCACCCAAGTTGTAATTTTAGACTAGAAACTATTAATAATGGTTTGTGGTTTAATTCACTTTCAGT
TTCAATTGCTTACGGTCCTCTTCAAGGTGGTTGCAAGCAATCTGTCTTTAGTGGTAGAGCAACCTGTTGTTAT
GCTTACTCATATGGAGGTCCTTTGCTGTGTAAAGGTGTTTATTCAGGTGAGTTAGATCATAATTTTGAATGTG
GACTGTTAGTTTATGTTACTAAGAGCGGTGGCTCTCGTATACAAACAGCCACTGAACCGCCAGTTATAACTCA
ACACAATTATAATAATATTACTTTAAATACTTGTGTTGATTATAATATATATGGCAGAACTGGCCAAGGTTTTATT
ACTAATGTAACCGACTCAGCTGTTAGTTATAATTATCTAGCAGACGCAGGTTTGGCTATTTTAGATACATCTGG
TTCCATAGACATCTTTGTCGTACAAAGTGAATATGGTCTTAATTATTATAAGGTTAACCCTTGCGAAGATGTCA
ACCAGCAGTTTGTAGTTTCTGGTGGTAAATTAGTAGGTATTCTTACTTCACGTAATGAGACTGGTTCCCAGCT
TCTTGAGAATCAGTTTTACATCAAAATCACTAATGGAACACGTCGTTTTAGACGT
>CK-CH-XJ-TK50-2019.seq
ATGTTGGGGAAGTCACTGTTTTTAGTGACCATTTTGTGTGCACTATGTAGTGCAAATTTGTTTGATTCTGGCA

ATAATTATGTGTACTACTACCAAAGTTCCTATAGACCACCAAATGGATGGCATTTGCAGGGGGGTGCTTATGC
AGTAGTGAATTCTACTATTAAATATAACAATGCAGGCGACGCTCACGAGTGTTCTGTAGGTGTTCTTTTTAATT
ATTCTAACGGAAATGATGTTGGTTATAATAATAGTGCTTCTTCCGTAGCCATGACAGCACCGTTGCCTGGTATG
TCTTGGTCTAAAAACGAATTTTGTACTGCCCACTGTAACTTTTCGGATGTTACAGTGTTTGTTACACATTGTTA
TGCAAACTCTTGTCCTTTAACAGGTCAGATAGAGAAGGGCCGTGTTCGTATTTCTGCTATGAAAAATGGTGCT
CTATTTTATAATTTAACAGTTAGTGTATCTCAATACCCTAAATTTAAATCGCTTCAATGTGTTAACAACTTCACAT
CTGTTTATTTAAATGGTGATCTTGTTTTTACTTCCAACAAAACTACTGACGTTACAGGTGCTGGTGTGTACTTT
AAATCAGGTGGGCCTATAACCTACAAAATTATGAGAGAATTTAAGGTTTTGGCTTATTTTGTAAATGGCACTG
TGCAAGATGTAATTTTGTGTGATGACACACCGAGAGGCTTGCTTGCATGTCAATATAATACTGGTAATTTTTCA
GATGGGTTTTATCCTTTTACTAATTCTAGTTTAGTTAAGGAAAAATTTATTGTTTATCGTGAGAATAGTGTTAAT
ACCACTCTTACTTTAACTAACTATACTTTTTTAAATGAGACTGGTGCCTCTCCTAATTTAGGTGATGTTTATACTA
TTCCAACTTATCAAACACAAACAACTCAGAGTGGTTATTATAATTTTAATTTATCATTTCTGAGTAGTTTTGTGT
ATGTAGAGTCTAATTACATGTATGGGTCTTATCACCCTGCATGTAATTTTAGATTAGAAACTATTAACAATGGCT
TGTGGTTTAATTCACTGTCAGTTTCGTTAGCTTACGGACCACTTCAAGGTGGTTGTAAGCAGTCCGTCTTTAG
TGGTAGGACCACTTGTTGTTATGCTTATTCATACGGGGGCCCTCGCCTCTGTAAGGGTGTTTACAGTGGCGA
GTTACAACAAAGTTTTGAATGTGGACTGTTGGTTTATGTTACTAAGAGTGATGGCTCTCGTATACAAACAGCC
ATAAAACCACCGGTCATAACTCAACACAATTATAATAATATTACTTTAAATACTTGTGTTGAGTACAATATATATG
GCAGAGTTGGCCAAGGTTTTATTACTAATGTAACTGACCCCGCAGCTAGCTATAATTACTTAGCAGATGCTGG
ATTGGCAATTTTAGATACTTCAGGTGCCATAGACACTTTCGTTTTACAAGGTGAACATGGTCTCAATTATTATA
AGGTTAACCCTTGTGAAGATGTTAACCAGCAGTTTGTAGTGTCAGGCGGTAAGTTAGTAGGCGTTCTGACTT
CTCGTAATGAAACTGGTTCTCAGCCTCTTGAAAACCAGTTTTATATTAAGTTAACTAATGGAAGCCGTCGTTTT
AGACGT
>CK_CH_GDQY_YXS2019.seq
ATGTTGGGGAAGTCACTGTTTTTAGTGACCATTTTGTGTGCACTATGTAGTGCAAATTTGTTTGATTCTGCTAA
TAATTATGTGTACTACTACCAAAGTGCCTTTAGGCCTCCAAATGGATGGCATCTGCAAGGTGGTGCTTATGCA
GTAGTGAATTCTACTAATTATACTAATAATGCCGGTTCTGCAAGTGAGTGCACTGTTGGTGTTATTAAGGACGT
CTATAATCAAAGTGCGGCTTCCATAGCTATGACAGCACCTCCTCAGGGTATGGCTTGGTCTAAGTTACAATTTT
GTAGTGCACACTGTAAATTTTCTGAAATTACAGTTTTTGTCACACATTGTTATAGTAGTGGTGCAGGTTCTTGT
CCTATAACAGGCATGATTGCACGTGATCATATTCGTATTTCTGCAATGAAAAATGGTTCTTTATTTTATAATTTA
ACAGTTAGCGTATCTAAATACCCTAAGTTTAAATCTTTTCAATGTGTTAACAATTTCACATCTGTTTATCTAAAT
GGTAATCTTGTTTTTACTTCTAACAAAACTGCTGATGTTACATCTGCAGGTGTGTATTTTAAAGCAGGTGGAC
CTGTAAATTATAATATTATGAAAGAATTTAAGGTTCTGGCTTATTTTGTCAATGGTACTGTGCGAGATGTAATTC
TGTGTGATGACACACCGAGAGGCTTGCTTGCATGTCAATATAATAATGGTAATTTTTCAGATGGGTTTTACCCT
TTTACTAATTCTAGTTTAGTTAAGGAAAAGTTTATTGTTTATCGTGAGAATAGTGTTAATACTACTCTTACTTTA
ACTAACTATACTTTTTATAATGTGACTAATGCCTCGCCTAATCGAGGTGGTGTTCAGTCTATTCCAACTTATCAA
ACACAAACAGCTCAGAGTGGTTATTATAATTTTAATTTATCATTTCTGAGTAGTTTTGTGTATAAAGAGTCTAAT
TACATGTATGGGTCTTACCACCCTGCATGTAATTTTAGATTAGAAACTATTAATAATGGCTTGTGGTTTAATTCA
TTGTCAGTTTCGCTTGCTTATGGACCACTTCAAGGTGGGTGTAAGCAGTCGGTTTTTAGTAGTAGAGCCACT
TGTTGTTATGCTTATTCATATAATGGTCCTCGCGCATGTAAGGGTGTTTACGCAGGCGAGTTACTACAAAATTT
TGAATGTGGACTGTTGGTTTATGTTACTAAGAGCGATGGCTCTCGTATACAAACAGCCACCGTTCCACCAGTT
GTAACTCAACACAATTATAATAATATTACTTTAAATACTTGTGTTGATTATAATATATATGGCAGAGTTGGTCGA
GGTTTTATTACTAATGTAACTGACTCATCATCTAGTTATAATTATTTAGCAGATGCAGGGTTGGCTATTTTAGAT
ACATCAGGTGCCATAGACATCTTTGTTGTACAAGGTGAACATGGTCTTAATTATTACAAGGTTAATCCCTGTGA
AGATGTAAACCAGCAGTTTGTAGTTTCTGGTGGTAAATTAGTAGGTATTCTTACCTCACGTAATGCAACAGGT

TCTCAGCCTCTTGAGAATCAATTCTACATTAAACTCACTAAAGAGACACGTCGTTTTAGACGT
>CK_CH_GX_NL1206_2019.seq
ATGTTGGGGAAGTCACTGTTTTTAGTGACCATTTTGTGTGCACTATGTAGTGCAAATTTGTTTGATTCTGCTAA
TAATTATGTGTACTACTACCAAAGTGCCTTTAGGCCTCCAAATGGATGGCATCTGCAAGGGGGTGCTTATGCA
GTAGTGAATTCTACTAATTATACTAATAATGCCGGTTCTGCAAGTGAGTGCACTGTTGGTGTTATTAAGGACGT
CTATAATCAAAGTGCGGCTTCCATAGCTATGACAGCACCTCCTCAGGGTATGGCTTGGTCTAAGTTACAATTTT
GTAGTGCACACTGTAACTTTTCTGAAATTACAGTTTTTGTCACACATTGTTATAGTAGTGGTGCAGGTTCTTGT
CCTATAACAGGCATGATTGCACGTGATCATATTCGTATTTCTGCAATGAAAAATGGTTCTTTATTTTATAATTTA
ACAGTTAGCGTATCTAAATACCCTAAGTTTAAATCTTTTCAATGTGTTAACAATTTCACATCTGTTTATCTAAAT
GGTGATCTTGTTTTTACTTCTAACAAAACTGCTGATGTTACATCTGCAGGTGTGTATTTTAAAGCAGGTGGAC
CTGTAAATTATAATATTATGAAAGAATTTAAGGTTCTGGCTTATTTTGTCAATGGTACTGTGCGAGATGTAATTC
TGTGTGATGACACACCGAGAGGCTTGCTTGCATGTCAATATAATAATGGTAATTTTTCAGATGGGTTTTACCCT
TTTACTAATTCTAGTTTAGTTAAGGAAAAGTTTATTGTTTATCGTGAGAATAGTGTTAACACTACTCTTACTTTA
ACTAACTATACTTTTTATAATGTGACTAATGCCTCGCCTAATCGAGGTGGTGTTCAGTCTATTCCAACTTATCAA
ACACAAACAGCTCAGAGTGGTTATTATAATTTTAATTTATCATTTCTGAGTAGTTTTGTGTATAAAGAGTCTAAT
TACATGTATGGGTCTTACCACCCTGCATGTAATTTTAGATTAGAAACTATTAATAATGGCTTGTGGTTTAATTCA
TTGTCAGTTTCGCTTGCTTATGGACCACTTCAAGGCGGGTGTAAGCAGTCGGTTTTTAGTAGTAGAGCCACT
TGTTGTTATGCTTATTCATATAATGGTCCTCGCGCATGTAAGGGTGTTTACGCAGGCGAGTTACTACAAAATTT
TGAATGTGGACTGTTGGTTTATGTTACTAAGAGCGATGGCTCTCGTATACAAACAGCCACCGTTCCACCAGTT
GTAACTCAACACAATTATAATAATATTACTTTAAATACTTGTGTTGATTATAATATATATGGCAGAGTTGGTCGA
GGTTTTATTACTAATGTAACTGACTCATCATCTAGTTATAATTATTTAGCAGATGCAGGGTTGGCTATTTTAGAT
ACATCAGGTGCCATAGACATCTTTGTTGTACAAGGTGAACATGGTCTTAATTATTACAAGGTTAATCCCTGTGA
AGATGTAAACCAGCAGTTTGTAGTTTCTGGTGGTAAATTAGTAGGTATTCTTACCTCACGTAATGCAACAGGT
TCTCAGCCTCTTGAGAATCAATTCTACATTAAACTCACTAAAGAGACACGTCGTTTTAGACGT
>CK_CH_GX_NL-XJ1216_2019.seq
ATGTTGGGGAAGTCACTGTTTTTAGTGACCATTTTGTGTGCACTATGTAGTGCAAATTTGTTTGATTCTGCTAA
TAATTATGTGTACTACTACCAAAGTGCCTTTAGGCCTCCAAATGGATGGCATCTGCAAGGGGGTGCTTATGCA
GTAGTGAATTCTACTAATTATACTAATAATGCCGGTTCTGCAAGTGAGTGCACTGTTGGTGTTATTAAGGACGT
CTATAATCAAAGTGCGGCTTCCATAGCTATGACAGCACCTCCTCAGGGTATGGCTTGGTCTAAGTTACAATTTT
GTAGTGCACACTGTAACTTTTCTGAAATTACAGTTTTTGTCACACATTGTTATAGTAGTGGTGGAGGTTCTTGT
CCTATAACAGGCATGATTGCACGTGATCATATTCGTATTTCTGCAATGAAAAATGGTTCTTTATTTTATAATTTA
ACAGTTAGCGTATCTAAATACCCTAAGTTTAAATCTTTTCAATGTGTTAACAATTTCACATCTGTTTATCTAAAT
GGTGATCTTGTTTTTACTTCTAACAAAACTGCTGATGTTACATCTGCAGGTGTGTATTTTAAAGCAGGTGGAC
CTGTAAATTATAATATTATGAAAGAATTTAAGGTTCTGGCTTATTTTGTCAATGGTACTGTGCGAGATGTAATTC
TGTGTGATGACACACCGAGAGGCTTGCTTGCATGTCAATATAATAATGGTAATTTTTCAGATGGGTTTTACCCT
TTTACTAATTCTAGTTTAGTTAAGGAAAAGTTTATTGTTTATCGTGAGAATAGTGTTAACACTACTCTTACTTTA
ACTAACTATACTTTTTATAATGTGACTAATGCCTCGCCTAATCGAGGTGGTGTTCAGTCTATTCCAACTTATCAA
ACACAAACAGCTCAGAGTGGTTATTATAATTTTAATTTATCATTTCTGAGTAGTTTTGTGTATAAAGAGTCTAAT
TACATGTATGGGTCTTACCACCCTGCATGTAATTTTAGATTAGAAACTATTAATAATGGCTTGTGGTTTAATTCA
TTGTCAGTTTCGCTTGCTTATGGACCACTTCAAGGCGGGTGTAAGCAGTCGGTTTTTAGTAGTAGAGCCACT
TGTTGTTATGCTTATTCATATAATGGTCCTCGCGCATGTAAGGGTGTTTACGCAGGCGAGTTACTACAAAATTT
TGAATGTGGACTGTTGGTTTATGTTACTAAGAGCGATGGCTCTCGTATACAAACAGCCACCGTTCCACCAGTT
GTAACTCAACACAATTATAATAATATTACTTTAAATACTTGTGTTGATTATAATATATATGGCAGAGTTGGTCGA
GGTTTTATTACTAATGTAACTGACTCATCATCTAGTTATAATTATTTAGCAGATGCAGGGTTGGCTATTTTAGAT

ACATCAGGTGCCATAGACATCTTTGTTGTACAAGGTGAACATGGTCTTAATTATTACAAGGTTAATCCCTGTGA
AGATGTAAACCAGCAGTTTGTAGTTTCTGGTGGTAAATTAGTAGGTATTCTTACCTCACGTAATGCAACAGGT
TCTCATCCTCTTGAGAATCAATTCTACATTAAACTCACTAAAGAGACACGTCGTTTTAGACGT
>CK_CH_GX_NL-NZ1216_2019.seq
ATGTTGGGGAAGTCACTGTTTTTAGTGACCATTTTGTGTGCACTATGTAGTGCAAATTTGTTTGATTCTGCTAA
TAATTATGTGTACTACTACCAAAGTGCCTTTAGGCCTCCAAATGGATGGCATCTGCAAGGGGGTGCTTATGCA
GTAGTGAATTCTACTAATTATACTAATAATGCCGGTTCTGCAAGTGAGTGCACTGTTGGTGTTATTAAGGACGT
CTATAATCAAAGTGCGGCTTCCATAGCTATGACAGCACCTCCTCAGGGTATGGCTTGGTCTAAGTTACAATTTT
GTAGTGCACACTGTAACTTTTCTGAAATTACAGTTTTTGTCACACATTGTTATAGTAGTGGTGCAGGTTCTTGT
CCTATAACAGGCATGATTGCACGTGATCATATTCGTATTTCTGCAATGAAAAATGGTTCTTTATTTTATAATTTA
ACAGTTAGCGTATCTAAATACCCTAAGTTTAAATCTTTTCAATGTGTTAACAATTTCACATCTGTTTATCTAAAT
GGTGATCTTGTTTTTACTTCTAACAAAACTGCTGATGTTACATCTGCAGGTGTGTATTTTAAAGCAGGTGGAC
CTGTAAATTATAATATTATGAAAGAATTTAAGGTTCTGGCTTATTTTGTCAATGGTACTGTGCGAGATGTAATTC
TGTGTGATGACACACCGAGAGGCTTGCTTGCATGTCAATATAATAATGGTAATTTTTCAGATGGGTTTTACCCT
TTTACTAATTCTAGTTTAGTTAAGGAAAAGTTTATTGTTTATCGTGAGAATAGTGTTAACACTACTCTTACTTTA
ACTAACTATACTTTTTATAATGTGACTAATGCCTCGCCTAATCGAGGTGGTGTTCAGTCTATTCCAACTTATCAA
ACACAAACAGCTCAGAGTGGTTATTATAATTTTAATTTATCATTTCTGAGTAGTTTTGTGTATAAAGAGTCTAAT
TACATGTATGGGTCTTACCACCCTGCATGTAATTTTAGATTAGAAACTATTAATAATGGCTTGTGGTTTAATTCA
TTGTCAGTTTCGCTTGCTTATGGACCACTTCAAGGCGGGTGTAAGCAGTCGGTTTTTAGTAGTAGAGCCACT
TGTTGTTATGCTTATTCATATAATGGTCCTCGCGCATGTAAGGGTGTTTACGCAGGCGAGTTACTACAAAATTT
TGAATGTGGACTGTTGGTTTATGTTACTAAGAGCGATGGCTCTCGTATACAAACAGCCACCGTTCCACCAGTT
GTAACTCAACACAATTATAATAATATTACTTTAAATACTTGTGTTGATTATAATATATATGGCAGAGTTGGTCGA
GGTTTTATTACTAATGTAACTGACTCATCATCTAGTTATAATTATTTAGCAGATGCAGGGTTGGCTATTTTAGAT
ACATCAGGTGCCATAGACATCTTTGTTGTACAAGGTGAACATGGTCTTAATTATTACAAGGTTAATCCCTGTGA
AGATGTAAACCAGCAGTTTGTAGTTTCTGGTGGTAAATTAGTAGGTATTCTTACCTCACGTAATGCAACAGGT
TCTCATCCTCTTGAGAATCAATTCTACATTAAACTCACTAAAGAGACACGTCGTTTTAGACGT
>CK_CH_XN-XYWS_LJF_2019.seq
ATGTTGGGGAAGTCACTGTTTTTAGTGACCATTTTGTGTGCACTATGTAGTGCAAATTTGTTTGATCCTGCCA
ATAATTATGTGTACTACTACCAAAGTGCCTATAGGCCTCCAAATGGATGGCATCTGCAAGGGGGTGCTTATGC
AGTAGTGAATTCTACTAATTATGATTGTGCAAGTGAGTGCACTGTTGGTGTTATTAAGGACGTCTATAATCAAA
GTGCGGCTTCCATAGCTATGACAGCACCTCCTCAGGGTATGACTTGGTCTAAGTTACAATTTTGTAGTGCACA
CTGTAACTTTTCTGAAATTACAGTTTTTGTCACACATTGTTATAGTAGTGGTGCAGGTTCTTGTCCATTGACAG
GTATGATTCCTCAGAATCATATTCGTATTTCTGCTATGAGATCTGGATCTTTATTTTATAATTTAACAGTTAGTGT
ATCTAAATACCCTAATTTTAAGTCTTTTCAATGTGTTAACAACTTCACATCTGTTTATTTAAATGGTGATCTTGTT
TTTACTTCCAACAAAACTACTGATGTTACGTCAGCAGGTGTGTATTTTAAAGCAGGTGGACCTGTAAATTATA
GTATTATGAAAGAATTTAAGGTTCTTGCTTACTTTGTTAATGGTACAGCACAAGATGTAATTTTGTGCGACAA
GTCCCCCAAGGGTTTGCTAGCTTGTCAATATAACACTGGTAATTTTTCAGATGGATTCTACCCTTTTACTAATT
CTTCTTTAGTTAAGGATAGGTTTATTGTATATCGAGAAAGTAGCACTAACACTACTTTAGAGTTAACTAATTTC
ACTTTTACTAATGTAAGTAATGCTTCTCCTAATTCAGGTGGCGTTGATACTTTTCATTTATATCAAACACAAACA
GCTCAGAGTGGTTATTATAATTTTAATTTGTCATTTCTGAGTCAGTTTGTGTATAAGGCAAGTGATTTTATGTAT
GGGTCTTACCACCCTAAGTGTTCTTTTAGGCCAGAAACCATTAATAGTGGTTTATGGTTTAATTCCTTGTCAGT
TTCTCTTACTTATGGACCCCTACAGGGAGGGTGTAAGCAATCTGTTTTTAAAGGTAGGGCAACGTGTTGTTAT
GCCTACTCTTATAATGGCCCAAGGGCATGTAAAGGTGTTTATTCAGGTGAATTAAGCACTAATTTTGAATGTG
GATTGTTGATTTATGTTACTAAGAGTGATGGCTCTCGTATACAGACTAGAACAGAGCCCTTAGTATTAACGCA

ACACAATTATAATAATATTACTTTAGATAAGTGTGTTGACTATAATATATATGGCAGAGTAGGCCAAGGTTTTAT
TACTAATGTGACTAATTCTGCTGCTAATTTTAGTTATTTAGCAGATGGTGGGTTAGCTATTTTAGATACTTCCGG
TGCCATAGATGTCTTTGTTGTACAGGGCAGCTATGGTCTTAACTATTACAAGGTCAATCCTTGTGAAGATGTTA
ATCAACAGTTTGTAGTGTCTGGTGGCAATATAGTTGGCATTCTTACTTCTAGAAATGAAACAGGTTCTGAACA
GGTTGAGAACCAGTTTTATGTTAAGTTAACCAATAGCTCACATCGTCATAGGCGT
>CK_CH_GD_KPlhnm_2019.seq
ATGTTGGGGAAGTCACTGTTAATAGTGACTGTTTTGTTTGCACTATGTAGTGCCACTCTTCATACTGACGATTA
TGTTTACTACTACCAAAGTGCCTATAGACCACCAAATGGATGGCATTTGCAAGGGGGTGCTTATGCAGTAGT
GAATTCTACTTCTAAATATAATAATGCAGGCAACGCTAGCGAGTGTTCTGTAGGTGTTCTTTTTAATTACACTA
ACGGAAATGACGTTGGTTATAATAATAAAGCTTCTTCTGTAGCCATGACAGCACCGACTTCTGGTATGTCTTG
GTCTAAAGCACAATTTTGCACTGCCCATTGTAACTTCTCGGATTTACAGTGTTTGTTACACATTGTTTTGCAT
CTTCTTGTCCTTTAACAGGTAGGATAGAGCAAAACCATATCCGTATTTCTGCCATGAGAAATGGTTCTCTATTT
TATAATTTAACAGTTAGTGTATCCAAATACCCTAAATTTAAATCGCTTCAATGTGTTAACAATTTCACTTCTGTCT
ATTTAAATGGTGACCTTGTTTTTACTTCTAACGAAACCACTGATGTTATAGGTGCTGGTGTGTATTTTAAAGCA
GGTGGGCCTATAACCTATAAAATTATGAAAGAGTTTAAGGTTTTGGCTTATTTTGTTAATGGTACTGTGCAAG
ATGTAATTCTGTGTGATGACACACCGAGAGGCTTGCTAGCTTGTCAATATAACATTGGCAATTTTTCAGATGG
CTTTTATCCTTTTACTAATACTACTTTAGTTAGGGAAAAGTTCACCGTATATCGCGAAAGTAGTGTTAATACTAC
TCTGGCGTTAATTAATTTCACTTTTGCTAATGTAAGTAATGCACAGCCTAATAGTGGTGGTGTTAATACTTTTCA
TCTATATCAAACACAAACAGCTCAGAGTGGTTATTATAATTTTAATTTATCATTTCTGAGTGGTTTTGTGTATAA
AGCTTCTGATTATATGTATGGGTCTTACCACCCACGATGTAGTTTTAGACCAGAAACTATTAATAATGGCTTGT
GGTTTAATTCCTTGTCAGTTTCTCTAGCTTATGGACCACTTCAAGGTGGGTGTAAGCAGTCAGTTTTTAGTGG
TAGGGCAACGTGTTGCTATGCCTACTCTTACAATGGCCCGGTAGCCTGTAAAGGTGTTTATTCAGGCGAATTA
CGGACTAATTTTGAATGTGGATTGCTGATTTATGTTACTAAGAGTGATGGCTCTCGTATACAGACTAGAACAG
AGCCCTTAGTATTAACGCAACACAATTATAATAATATTACTTTAGATAAGTGTGTTGACTATAATATATATGGCAG
AGTAGGCCAAGGTTTTATTACTAATGTGACTGATTCTGCTGCTAATTTTAGTTATTTAGCAGATGGTGGGTTAG
CTATTTTAGATACTTCTGGTGCCATAGATGTCTTTGTTGTACAGGGCAGCTATGGTCTTAATTATTACAAGGTC
AATCCTTGTGAAGATGTTAACCAACAGTTTGTAGTGTCTGGTGGCAATATAGTTGGTATTCTTACTTCTAGAAA
TGAAACAGGTTCTGAACAGGTTGAGAACCAGTTTTATGTTAAGTTAACCAATAGCTCACATCGTCGCAAGCG
T
>CK_CH_XN-XYWS_LCC_2019.
seqatgttggggaagtcactgttttagtgaccattttgtgtgcactatgtagtgcaaatttgtttgattctgccaataattatgtgtactactacc
aaagtgcctttaggcctccaaatggatggcatctgcaagggggtgcttatgcagtagtgaattctactaattatactaataatgccggttctgc
aagtgggtgcactgttggtgttattaaggacgtctataatcaaagtgcggcttccatagctatgacagcacctcctcagggtatggcttggtct
aagtcacaattttgtagtgcacactgtaacttttctgaaattacagtttttgtcacacattgttatagtagtggtgcaggttcttgtcctataacag
gcatgattgcacgtgattatattcgtatttctgcaatgaaaaatggttctttattttataatttaacagttagcgtatttaaataccctacgtttaa
atcttttcaatgtgttaacaattccacatctgtttatctaaatggtgatcttgtttttacttctaacaaaaCTGCTGATGTTACGTCAGCA
GGTGTGTATTTTAAAGCAGGTGGACCCGTAAATTATAGTGTTATGAAAGAATTTAAGGTTCTTGCTTACTTTG
TTAATGGTACAGTACAAGATGTAATTTTGTGCGACAATTCCCCCAAGGGTTTGCTGGCTTGTCAATATAATACT
GGCAATTTTTCAGATGGCTTTTATCCTTTTACTAATAGTATTTTAGTTAGGGACAAGTTCATTGTCTATCGTGAA
AGTAGTGTTAATACTACTTTGACGTTAACTAATTTCACTTTTACTAATGTAAGTACTGCACAGCCTAATAGTGGT
GGTGTTAGTACTTTTCATTTATATCAAACACAAACAGCTCAGAGTGGTTATTATAATTTTAATTTGTCATTTCTG
AGTCAGTTTGTGTATAAGGCAAGTGATTTTATGTATGGGTCTTACCACCCTAGGTGTTCTTTTAGACCAGAAA
CCATTAATAGTGGTTTATGGTTTAATTCCTTGTCAGTTTCTCTTACTTATGGACCCCTACAGGGAGGGTGTAAG
CAATCTGTTTTTAATGGTAAGGCAACGTGTTGTTATGCCTACTCTTATAATGGCCCAAGGGCATGTAAAGGTG

TTTATTCAGGTGAATTAAGCAAGACTTTTGAATGTGGATTGCTGGTTTATGTTACTAAGAGTGATGGCTCTCG
TATACAGACTAGAACGGAGCCCTTAGTATTAATGCAACACAATTATAATAATATTACTTTAGATAAGTGTGTTGA
CTATAATATATATGGCAGAGTAGGCCAAGGTTTTATTACTAATGTGACTGATTCTGCTGCTAATTTTAGTTATTT
AGCAGATGGTGGGTTAGCTATTTTAGATACTTCGGGTGCCATAGATGTCTTTGTTGTACAGGGCAGCTATGGT
CTTAATTATTACAAGGTCAATCCTTGTGAAGATGTTAACCAACAGTTTGTAGTGTCTGGTGGCAATATAGTTG
GCATTCTTACTTCTAGAAATGAAACAGGTTCTGAACAGGTTGAGAACCAGTTTTATGTTAAGTTAACCAATAG
CTCACATCGTCGCAGGCGT
>CK-CH-GX-BSlb_201908.seq
ATGTTGGTGAAGTCACTGTTTTTAGTGACCATTTTGTGTGCACTATGTAGTGCAAATTTGTTTGATCCTGATAA
TAATTATGTGTACTACTACCAAAGTGCTTTTAGACCGCCAAATGGGTGGCACCTACAAGGAGGTGCTTATGCA
GTAGTCAATTCTACTAATTATACTAATAATGCCGGTTCTGCACATGAGTGCACTGTTGGTGTTATTAAGGATGT
TTATAATCAAAGTGTGGCTTCCATAGCTATGAAAGCACCTCTTCAGGGTATGGCTTGGTCTAAGTCACAATTCT
GTAGTGCACACTGTAATTTTTCTGAAATTACAGTTTTTGTCACACATTGTTATAGTAGTGGTAGCGGGTCTTGT
CCTATAACAGGCATGATTCCACGTTATCATATTCGTATTTCTGCAATGAAAAATGGTTCTTTATTTTATAATTTAA
CAGTTAGCGTATCTAAATACCCTAATTTTAAATCTTTTCAATGTGTTAACAACTTCACATCTGTTTATTTAAATGG
TGATCTTGTTTTTACTTCCAATAAAACTACTGATGTTACGTCAGCAGGTGTGTATTTTAAAGCAGGTGGACCT
GTAAATTATAGTATTATGAAAGAATTTAAGGTTCTTGCTTACTTTGTTAATGGTACAGCACAAGATGGTAGTTT
TGTGCGACAATTCCCCCAAGGGTTTGCTAGCTTGTCAATATAACACTGGCAATTTTTCAGATGGCTTTTATCCT
TTTACTAATAGTACTTTGGTTAAGGAAAAGTTCATCGTCTATCGCGAAAGTAGTGTTAATACTACTCTGGCGTT
AACTAATTTCACTTTTACTAATGTAAGTAATGCACAGCCTAATAGTGGTGGTGTTAATACTTTTCATTTATACCA
AACACAAACAGCTCAGAGTGGTTATTATAATTTTAATTTGTCATTTCTGAGTCAGTTTGTGTATAAGGCAAGT
GATTTTATGTATGGGTCCTACCACCCCTAGTTGTTCTTTTAGACCAGAAACCATTAATAGTGGTTTATGGTTTA
ATTCCTTGTCAGTTTCTCTTACTTATGGACCCCTACAGGGAGGGTGTAAGCAATCTGTTTTTAGTGGTAAGGC
AACGTGTTGTTACGCCTACTCTTATAAAGGCCCAATGGCATGTAAAGGTGTTTATTCATTTGAATTAAGCACG
AATTTTGAATGTGGATTGCTGGTTTATGTTACTAAGAGTGATGGCTCTCGTATACAGACTAGAACAGAGCCCT
TAGTATTAACGCAATACAATTATAATAATATTACTTTAGATAAGTGTGTTGCCTATAATATATATGGCAGAGTAGG
CCAAGGTTTTATTACTAATGTGACTGATTCTGCTGCTAATTTTAGTTATTTAGCAGATGGTGGGTTAGCTATTTT
AGATACGTCGGGTGCCATAGATGTTTTTGTTGTACAGGGCATCTATGGTCTTAATTATTACAAGGTTAATCCTT
GTGAAGATGTTAATCAACAATTTGTAGTGTCTGGTGGCAATATAGTTGGCATTCTTACTTCTAGAAATGAAAC
AGGTTCTGAACAGGTTGAGAACCAGTTTTATGTTAAGTTAACCAATAGCTCACATCGTCGTAGGCGT
>CK-CH-GD-QYpmr-20198.seq
ATGTTGGTGAAGTCACTGTTTTTAGTGACCATTTTGTGTGCACTATGTAGTGCAAATTTGTTTGATYCTGATAA
TAATTATGTGTACTACTACCAAAGTGCTTTTAGACCGCCAAATGGGTGGCACCTACAAGGAGGTGCTTATGCA
GTAGTCAATTCTACTAATTATACTAATAATGCCGGTTCTGCACATGAGTGCACTGTTGGTGTTATTAAGGATGT
TTATAATCAAAGTGTGGCTTCCATAGCTATGAAAGCACCTCTTCAGGGTATGGCTTGGTCTAAGTCACAATTCT
GTAGTGCACACTGTAATTTTTCTGAAATTACAGTTTTTGTCACACATTGTTATAGTAGTGGTAGCGGGTCTTGT
CCTATAACAGGCATGATTCCACGTGATCATATTCGTATTTCTGCAATGAAAAATGGTTCTTTATTTTATAATTTAA
CAGTTAGCGTATCTAAATACCCTAATTTTAAATCTTTTCAATGTGTTAACAACTTCACATCTGTTTATTTAAATGG
TGATCTTGTTTTTACTTCCAATAAAACTACTGATGTTACGTCAGCAGGTGTGTATTTTAAAGCAGGTGGACCT
GTAAATTATAGTATTATGAAAGAATTTAAGGTTCTTGCTTACTTTGTTAATGGTACAGCACAAGATGGTAGTTT
TGTGCGACAATTCCCCCAAGGGTTTGCTAGCTTGTCAATATAACACTGGCAATTTTTCAGATGGCTTTTATCCT
TTTACTAATAGTACTTTGGTTAAGGAAAAGTTCATCGTCTATCGCGAAAGTAGTGTTAATACTACTCTGGCGTT
AACTAATTTCACTTTTACTAATGTAAGTAATGCACAGCCTAATAGTGGTGGTGTTAATACTTTTCATTTATACCA
AACACAAACAGCTCAGAGTGGTTATTATAATTTTAATTTGTCATTTCTGAGTCAGTTTGTGTATAAGGCAAGT

GATTTTATGTATGGGTCCTACCACCCTAGTTGTTCTTTTAGACCAGAAACCATTAATAGTGGTTTATGGTTTAAT
TCCTTGTCAGTTTCTCTTACTTATGGACCCCTACAGGGAGGGTGTAAGCAATCTGTTTTTAGTGGTAAGGCAA
CGTGTTGTTACGCCTACTCTTATAAAGGCCCAATGGCATGTAAAGGTGTTTATTCATTTGAATTAAGCACGAAT
TTTGAATGTGGATTGCTGGTTTATGTTACTAAGAGTGATGGCTCTCGTATACAGACTAGAACAGAGCCCTTAG
TATTAACGCAATACAATTATAATAATATTACTTTAGATAAGTGTGTTGCCTATAATATATATGGCAGAGTAGGCCA
AGGTTTTATTACTAATGTGACTGATTCTGCTGCTAATTTTAGTTATTTAGCAGATGGTGGGTTAGCTATTTTAGA
TACGTCGGGTGCCATAGATGTTTTTGTTGTACAGGGCATCTATGGTCTTAATTATTACAAGGTTAATCCTTGTG
AAGATGTTAATCAACAATTTGTAGTGTCTGGTGGCAATATAGTTGGCATTCTTACTTCTAGAAATGAAACAGG
TTCTGAACAGGTTGAGAACCAGTTTTATGTTAAGTTAACCAATAGCTCACATCGTCGTAGGCG
>CK_CH_XN-XYWS_FJH_2019.seq
ATGTTGGGGAAGTCACTGTTTTTAGTGACCATTTTGTGTGCACTATGTAGTGCAAATTTCTTTGATTCTGCCAA
TTATGTGTACTACTACCAAAGTGCCTTTAGGCCTCCAAATGGATGGCATTTGCAAGGGGGTGCTTATGCAGTA
GTGAATTCTACTAATTATACTAATAATGCCGGTTCTGCAAGTGAGTGCACTGTTGGTATTATTAAGGACGTTTAT
AATCAAAGTGCGGCTTCCATAGCTATGACAGCACCTCCTCAGGGTATGGCTTGGTCTAAGTCACAATTTTGTA
GTGCACACTGTAACTTTTCTGAAATTACAGTTTTTGTCACACATTGTTATAGAAGTGGTGCAGGTTCTTGTCC
TTTAACAGGCATGATTGCACGTGATCATATTCGTATTTCCGCAATGAAAAATGGTTTTTTATTTTATAATTTAAC
AGTTAGTGTATCTAAATACCCTAATTTTAAGTCTTTTCAATGTGTTAACAACTTCACATCTGTTTATTTAAATGGT
AATCTTGTTTTTACTTCCAACAAAACTACTGATGTTACGTCAGCAGGTGTGTATTTTAAAGCAGGTGGACCTG
TAAATTATAGTATTATGAAAGAATTTAAGGTTCTTGCTTACTTTGTTAATGGTACAGCACAAGATGTAATTTTGT
GCGACAAGTCCCCCAAGGGTTTGCTAGCTTGTCAATATAACACTGGCAATTTTTCAGATGGCTTTTATCCTTTT
ACTAATACTACTTTAGTTAGGGAAAAGTTCACTGTATATCGCGAAAGTAGTGTTAATACTACTCTGGTGTTAAC
TAATTTCACTTTTACTAATGTAAGTAATGCACAGCCCAATAGTGGTGGTGTTAGTACTTTTCATTTATATCAAAC
ACAAACAGCTCAGAGTGGTTATTATAATTTTAATTTGTCATTTCTGAGTCAGTTTGTGTATAAGGCAAGTGATT
TTATGTATGGGTCTTACCACCCTAAGTGTTCTTTTAGGCCAGAAACCATTAATAGTGGTTTATGGTTTAATTCCT
TGTCAGTTTCTCTTACTTATGGACCCCTACAGGGAGGGTGTAAGCAATCTGTTTTTAATGGTAGGGCAACGT
GTTGTTATGCCTACTCTTATAATGGCCCAAGGGCATGTAAAGGTGTTTATTCAGGTGAATTAAGCACTAATTTT
GAATGTGGATTGCTGATTTATGTTACTAAGAGTGATGGCTCTCGTATACAGACTAGAACAGAGCCCTTAGTAT
TAACGCAACACAATTATAATAATATTACTTTAGATAAGTGTGTTGACTATAATATATATGGCAGAGTAGGCCAAG
GTTTTATTACTAATGTGACTGATTCTGCTGCTAATTTTAGTTATTTAGCAGATGGTGGGTTAGCTATTTTAGATA
CTTCCGGTGCCATAGATGTCTTTGTTGTACAGGGCAGCTATGGTCTTAACTATTACAAGGTCAATCCTTGTGA
AGATGTTAATCAACAGTTTGTAGTGTCTGGTGGCAATATAGTTGGCATTCTTACTTCTAGAAATGAAACAGGT
TCTGAACAGGTTGAGAACCAGTTTTATGTTAAGTTAACCAATAGCTCACATCGTCATAGGCGT
>CK_CH_XN-XYWS_DYF_2019.seq
ATGTTGGGGAAGTCACTGTTTTTAGTGACCATTTTGTGTGCACTATGTAGTGCAAATTTGTTTGATTCTGCCA
ATAATTATGTGTACTACTACCAAAGTGCCTTTAGGCCTCCAAATGGATGGCATTTGCAAGGGGGTGCTTATGC
AGTAGTGAATTCTACTAATTATACTAATAATGCCGGTTCTGCAAGTCATTGCACTGTTGGTATTATTAAGGACG
TCTATAATCAAAGTGCGGCTTCCATAGCTATGACAGCACCTCCTCAGGGTATGACTTGGTCTAAGTCACAATTT
TGTAGTGCACACTGTAACTTTTCTGAAATTACAGTTTTTGTCACACATTGTTATAGTAGTGGTGCAGGGTCTT
GCCCTATAACAGGCATGATTGAACGTGATCATATTCGTATTTCTGCAATGAAAAATGGTTTTTTATTTTATAATT
TAACAGTTAGTGTATCTAAATACCCTAATTTTAAGTCTTTTCAATGTGTTAACAACTTCACATCTGTTTATTTAAA
TGGTGATCTTGTTTTTACTTCCAACAAAACTACTGATGTTACGTCAGCAGGTGTGTATTTTAAAGCAGGTGGA
CCTGTAAATTATAGTATTATGAAAGAATTTAAGGTTCTTGCTTACTTTGTTAATGGTACAGCACAAGATGTAAT
TTTGTGCGACAAGTCCCCCAAGGGTTTGCTAGCTTGTCAATATAACACTGGCAATTTTTCAGATGGCTTTTAT
CCTTTTACTAATACTACTTTAGTTAGGGAAAAGTTCACTGTATATCGCGAAAGTAGTGTTAATACTACTCTGGT

GTTAACTAATTTCACTTTTACTAATGTAAGTAATGCACAGCCCAATAGTGGTGGTGTTAGTACTTTTCATTTATA
TCAAACACAAACAGCTCAGAGTGGTTATTATAATTTTAATTTGTCATTTCTGAGTCAGTTTGTGTATAAGGCAA
GTGATTTTATGTATGGGTCTTACCACCCTAAGTGTTCTTTTAGGCCAGAAACCATTAATAGTGGTTTATGGTTT
AATTCCTTGTCAGTTTCTCTTACTTATGGACCCCTACAGGGAGGGTGTAAGCAATCTGTTTTTAATGGTAGGG
CAACGTGTTGTTATGCCTACTCTTATAATGGCCCAAGGGCATGTAAAGGTGTTTATTCAGGTGAATTAAGCGC
TAATTTTGAATGTGGATTGCTGATTTATGTTACTAAGAGTGATGGCTCTCGTATACAGACTAGAACAGAGCCC
TTAGTATTAACGCAACACAATTATAATAATATTACTTTAGATAAGTGTGTTGACTATAATATATATGGCAGAGTAG
GCCAAGGTTTTATTACTAATGTGACTGATTCTGCTGCTAATTTTAGTTATTTAGCAGATGGTGGGTTAGCTATT
TTAGATACTTCCGGTGCCATAGATGTCTTTGTTGTACAGGGTAGCTATGGTCTTAATTATTACAAGGTCAATCC
TTGTGAAGATGTTAATCAACAGTTTGTAGTGTCTGGTGGTAATATAGTTGGCATTCTTACTTCTAGAAATGAAA
CAGGTTCTGAACAGGTTGAGAACCAGTTTTATGTTAAGTTAACCAATAGCTCACATCGTCATAGGCGT
>CK_CH_JLYB_LMH_2019.seq
ATGTTGGGGAAGTCACTGTTTTTAGTGACCATTTTGTGTGCACTATGTAGTGCAAATTTATTCGATCCTGCTAA
TACTTATGTGTACTACTACCAAAGTGCCTTTAGGCCTCCAAATGGATGGCACCTACAAGGGGGTGCTTATGCA
GTAGTCAATTCCACTAATTATACTAATAATGCCGGTTCTGCAGAACATTGCACTGTTGGTGTTATTAAGGACGT
CTATAATCAAAGTGCGGCTTCCATAGCTATGACAGCACCTCTTCAGGGTATGGCTTGGTCTAAGTCACAATTT
TGTAGTGCACACTGTAACTTTTCTGAAATTACAGTTTTTGTCACACATTGTTATAGTAGTGGTAGCGGGTCTTG
TCCTATAACAGGCATGATTGCACGTGATCATATTCGTATTTCTGCAATGAAAAATGGTACTTTATTTTATAATTTA
ACAGTTAGCGTATCTAAATACCCTAATTTTAAATCTTTTCAATGCGTTAATAATCTCACATCTGTTTATCTAAATG
GTAATCTTGTTTTTACTTCCAACAAAACTACTGATGTTACGTCAGCAGGTGTGTATTTTAAAGCAGGTGGACC
TGTAAATTATAGTATTATGAAAGAATTTAAGGTTCTTGCTTACTTTGTTAATGGTACAGCACAAGATGTAATTTT
GTGCGACAATTCCCCCAAGGGTTTGCTAGCTTGTCAATATAACACTGGCAATTTTTCAGATGGCTTTTATCCTT
TTACTAATAGTACTTTAGTTAGGGAAAAGTTCATCGTATATCGCGAAAGTAGTGTTAATACTACTCTGGCGTTA
ACTAATTTCACTTTTACTAATGTAAGTAATGCACAGCCTAATAGTGGTGGTGTTAATACTTTTCATCTATATCAA
ACACAAACAGCTCAGAGTGGTTATTATAATTTTAATTTGTCATTTCTGAGTCAGTTTGTGTATAAGGCAAGTGA
TTTTATGTATGGGTCCTACCACCCTAGTTGCTCTTTTAGACCAGAAACCATTAATAGTGGTTTGTGGTTTAATT
CTTTGTCAGTTTCTCTAGCTTACGGACCACTTCAAGGTGGGTGTAAGCAGTCAGTTTTTAGTGGTAGGGCAA
CGTGTTGCTATGCCTACTCTTACAATGGCCCGATAGCCTGTAAAGGTGTTTATTCAGGCGAATTACGGACTAA
TTTTGAATGTGGATTGCTGATTTATGTTACTAAGAGTGATGGCTCTCGTATACAGACTAGAACAGAGCCCTTA
GTATTAACGCAACACAATTATAATAATATTACTTTAGATAAGTGTGTTGACTATAATATATATGGCAGAGTAGGC
CAAGGTTTTATTACTAATGTGACTGATTCTGCTGCTAATTTTAGTTATTTAGCAGATGGTGGGTTAGCTATTTTA
GATACTTCGGGTGCCATAGATGTCTTTGTTGTACAGGGCAGCTATGGTCTTAATTATTACAAGGTCAATCCTTG
TGAAGATGTTAACCAACAGTTTGTAGTGTCTGGTGGCAATATAGTTGGCATTCTTACTTCTAGAAATGAAACA
GGTTCTGAACAGGTTGAGAACCAGTTTTATGTTAAGTTAACCAATAGCTCACATCGTCGCAGGCGT
>CK-CH-GD-DGhflyt_201909.seq
ATGTTGGGGAAGTCACTGTTTTTAGTGACCATTTTGTGTGCACTATGTAGTGCAAATTTATTCGATCCTGCTAA
TACTTATGTGTACTACTACCAAAGTGCCTTTAGGCCTCCAAATGGATGGCACCTACAAGGGGGTGCTTATGCA
GTAGTCAATTCCACTAATTATACTAATAATGCCGGTTCTGCACAACATTGCACTGTTGGTGTTATTAAGGATGT
CTATAATCAAAGTGCGGCTTCCATAGCTATGACAGCACCTCTTCAGGGTATGGCTTGGTCTAAGTCACAATTT
TGTAGTGCACACTGTAACTTTTCTGAAATTACAGTTTTTGTCACACATTGTTATAGTAGTGGTAGCGGGTCTTG
TCCTATAACAGGCATGATTGCACGTGATCATATTCGTATTTCTGCAATGAAAAATGGTACTTTATTTTATAATTTA
ACAGTTAGCGTATCTAAATACCCTAATTTTAAATCTTTTCAATGCGTTAATAATCTCACATCTGTTTATCTAAATG
GTGATCTTGTTTTTACTTCCAACAAAACTACTGATGTTACGTCAGCAGGTGTGTATTTTAAAGCAGGTGGACC
TGTAAATTATAGTATTATGAAAGAATTTAAGGTTCTTGCTTACTTTGTTAATGGTACAGCACAAGATGTAATTTT

GTGCGACAATTCCCCCAAGGGTTTGCTAGCTTGTCAATATAACACTGGCAATTTTTCAGATGGCTTTTATCCTT
TTACTAATAGTACTTTAGTTAGGGAAAAGTTCATCGTATATCGCGAAAGTAGTGTTAATACTACTCTGGCGTTA
ACTAATTTCACTTTTACTAATGTAAGTAATGCACAGCCTAATAGTGGTGGTGTTAATACTTTTCATCTATATCAA
ACACAAACAGCTCAGAGTGGTTATTATAATTTTAATTTGTCATTTCTGAGTCAGTTTGTGTATAAGGCAAGTGA
TTTTATGTATGGGTCCTACCACCCCTAGTTGTTCTTTTAGACCAGACACCATTAATAGTGGTTTGTGGTTTAATT
CTTTGTCAGTTTCTCTAGCTTACGGACCACTTCAAGGTGGGTGTAAGCAGTCAGTTTTTAGTGGTAGGGCAA
CGTGTTGCTATGCCTACTCTTACAATGGCCCGATAGCCTGTAAAGGTGTTTATTCAGGCGAATTACGGACTAA
TTTTGAATGTGGATTGCTGATTTATGTTACTAAGAGTGATGGTTCTCGTATACAGACTAGAACAGAGCCCTTA
GTATTAACGCAACACAATTATAATAATATTACTTTAGATAAGTGTGTTGACTATAATATATATGGCAGAGTAGGC
CAAGGTTTTATTACTAATGTGACTGATTCTGCTGCTAGTTATTTAGCAGATGGTGGGTTAGCTATTTTAGATAC
TTCGGGTGCCATAGATGTCTTTGTTGTACAGGGCAGCTATGGTCTTAATTATTACAAGGTCAATCCTTGTGAA
GATGTTAACAAACAGTTTGTAGTGTCTGGTGGCAATATAGTTGGCATTCTTACTTCTAGAAATGAAACAGGTT
CTGAACAGGTTGAGAACCAGTTTTATGTTAAGTTAACCAATAGCTCACATCGTCGCAGGCGT
>CK-CH-GD-DGhfljq_201909.seq
ATGTTGGGGAAGTCACTGTTTTTAGTGACCATTTTGTGTGCACTATGTAGTGCAAATTTATTCGATCCTGCTAA
TACTTATGTGTACTACTACCAAAGTGCCTTTAGGCCTCCAAATGGATGGCACCTACAAGGGGGTGCTTATGCA
GTAGTCAATTCCACTAATTATACTAATAATGCCGGTTCTGCACAACATTGCACTGTTGGTGTTATTAAGGACGT
CTATAATCAAAGTGCGGCTTCCATAGCTATGACAGCACCTCTTCAGGGTATGGCTTGGTCTAAGTCACAATTT
TGTAGTGCACACTGTAACTTTTCTGAAATTACAGTTTTTGTCACACATTGTTATAGTAGTGGTAGCGGGTCTTG
TCCTATAACAGGCATGATTGCACGTGATCATATTCGTATTTCTGCAATGAAAAATGGTACTTTATTTTATAATTTA
ACAGTTAGCGTATCTAAATACCCTAATTTTAAATCTTTTCAATGCGTTAATAATCTCACATCTGTTTATCTAAATG
GTGATCTTGTTTTTACTTCCAACAAAACTACTGATGTTACGTCAGCAGGTGTGTATTTTAAAGCAGGTGGACC
TGTAAATTATAGTATTATGAAAGAATTTAAGGTTCTTGCTTACTTTGTTAATGGTACAGCACAAGATGTAATTTT
GTGCGACAATTCCCCCAAGGGTTTGCTAGCTTGTCAATATAACACTGGCAATTTTTCAGATGGCTTTTATCCTT
TTACTAATAGTACTTTAGTTAGGGAAAAGTTCATCGTATATCGCGAAAGTAGTGTTCATACTACTCTGGCGTTA
ACTAATTTCACTTTTACTAATGTAAGTAATGCACAGCCTAATAGTGGTGGTGTTAATACTTTTCATCTATATCAA
ACACAAACAGCTCAGAGTGGTTATTATAATTTTAATTTGTCATTTCTGAGTCAGTTTGTGTATAAGGCAAGTGA
TTTTATGTATGGGTCCTACCACCCCTAGTTGTTCTTTTAGACCAGACACCATTAATAGTGGTTTGTGGTTTAATTT
TTTGTCAGTTTCTCTAGCTTACGGACCACTTCAAGGTGGGTGTAAGCAGTCAGTTTTTAGTGGTAGGGCAAC
GTGTTGCTATGCCTACTCTTACAATGGCCCGATAGCCTGTAAAGGTGTTTATTCAGGCGAATTACGGACTAAT
TTTGAATGTGGATTGCTGATTTATGTTACTAAGAGTGATGGTTCTCGTATACAGACTAGAACAGAGCCCTTAG
TATTAACGCAACACAATTATAATAATATTACTTTAGATAAGTGTGTTGACTATAATATATATGGCAGAGTAGGCC
AAGGTTTTATTACTAATGTGACTGATTCTGCTGCTAATTTTAGTTATTTAGCAGATGGTGGGTTAGCTATTTTA
GATACTTCGGGTGCCATAGATGTCTTTGTTGTACAGGGCAGCTATGGTCTTAATTATTACAAGGTCAATCCTTG
TGAAGATGTTAACAAACAGTTTGTAGTGTCTGGTGGCAATATAGTTGGCATTCTTACTTCTAGAAATGAAACA
GGTTCTGAACAGGTTGAGAACCAGTTTTATGTTAAGTTAACCAATAGCTCACATCGTCGCAGGCGT
>CK-CH-LN-SYhm-201905.seq
ATGTTGGGGAAGTCACTGTTTTTAGTGACCATTTTGTGTGCACTATGTAGTGCAAATTTGTTTGATTCTGCCA
ATAATTATGTGTACTACTACCAAAGTGCCTTTAGGCCTCCTACTGGATGGCATTTGCAAGGGGGTGCTTATGC
AGTAGTGAATTCTACTAATTACACTAGTAATGCTGGTTCTGCAAGTGAGTGCACTATTGGTGTCATTAAGGAC
GTTTATAATCAAAGTGCGGCTTCCATAGCTATGACAGCACCTCCTCAGGGTATGGCTTGGTCTAAGTCACAAT
TTTGTAGTGCACACTGTAACTTTTCTGAAATTACAGTTTTTGTCACACATTGTTATAGTAGTGGTACAGGGTCT
TGTCCTCTAACAGGCCTGATTGCACGTGATCATATTCGTATTTCAGCAATGAAAAATGGTTCTTTATTTTATAAT
TTAACAGTTAGCGTATCTAAATACCCTAGATTTAAGTCTTTTCAATGTGTTAACAACTTCACATCTGTTTATTTA

AATGGTGATCTTGTTTTTACTTCTAATAAAACTACTGATGTTACGTCAGCAGGTGTGTATTTTAAAGCAGGTG
GACCTGTAAACTATAGTGTTATGAAAGAATTTAAGGTTCTTGCTTACTTTGTTAATGGTACAGCACAAGATGTA
ATTTTGTGTGACAATTCCCCTAAGGGTTTGCTAGCCTGTCAATATAATACTGGCAATTTTTCAGACGGCTTTTA
TCCTTTTACTAATAGTACTTTAGTTAGGGACAAGTTCATTGTCTATCGTGAAAGTAGTGTTAATACTACTTTGAC
GTTAACTAATTTCACTTTTACTAATGTAAGTACTGCACAGCCTAATAGTGGTGGTGTTAGTACTTTTCATCTATA
TCAAACACAAACAGCTCAGAGTGGTTATTATAATTTTAATTTGTCATTTCTGAGTCAGTTTGTGTATAAGGCAA
GTGATTTTATGTATGGGTCTTATCATCCTAGGTGTTCTTTTAGACCAGAAACCATTAATAGTGGTTTATGGTTTA
ATTCTTTGTCAGTTTCTCTTACTTATGGACCCCTACAGGGAGGGTGTAAGCAATCTGTTTTTAGTGGTAGGGC
AACGTGTTGTTATGCCTACTCTTATAATGGCCCTAGGGCATGTAAAGGTGTTTATCCAGGTGAATTAAGCAAG
ACTTTTGAATGTGGATTGCTGGTTTATGTTACTAAGAGTGATGGCTCTCGTATACAGACTAGAACAGAGCCCT
TAGTATTAACGCAACACAATTATAATAATATTACTTTAGATAAGTGTGTTGACTATAATATATATGGCAGAGTAG
GCCAAGGTTTTATTACTAATGTGACTGATTCTGCTGCTAATTCTAGTTATTTAGCAGATGGTGGGTTAGCTATT
TTAGATACTTCGGGTGCCATAGATGTCTTTGTTGTACAGGGCAGCTATGGTCTTAATTATTACAAGGTCAATCC
TTGTGAAGATGTTAATCAACAGTTTGTAGTGTCTGGTGGCAATATAGTTGGCATGCTTACTTCTAGAAATGAA
ACAGGTTCTGAACAGGTTGAGAACCAGTTTTATGTTAAGTTAACCAATAGCTCACATCGTCGTAGGCGT
>CK-CH-HNxj_201909.seq
ATGTTGGGGAAGTCACTGTTTTTAGTGACCATTTTGTGTGCACTATGTAGTGCAAATTTGTTTGATTCTGCCA
ATAATTATGTGTACTACTACCAAAGTGCCTTTAGGCCTCCAAATGGATGGCATTTGCAAGGGGGTGCTTATGC
AGTAGTGAATTCTACTAATTATACTAGTAATGCCGGTTCTGCAAGTCAGTGCACTGTTGGTATTATTAAGGACG
TCTATAATCAAAGTGCGGCTTCCATAGCTATGACAGCACCTCCTCAGGGTATGGCTTGGTCTAAGTCACAATT
TTGTAGTGCACACTGTAACTTTTCTGAAATTACAGTTTTTGTCACACATTGTTTTAGTAGTGGTGCAGGGTCTT
GCCCTATAACAGGCATGATTGCACGTGATCATATTCGTATTTCTGCAATGAAAAATGGTTCTTTATTTTATAACT
TAACAGTTAGCGTATCTAAATACTCTAGGTTTAAGTCTTTTCAATGTGTTAACAACCTCACATCTGTTTATTTAA
ATGGTGATCTTGTTTTTACTTCCAATAAAACTACTGATGTTACGTCAGCAGGTGTGTATTTTAAAGCAGGTGG
ACCTGTAAATTATAGTGTTATGAAAGAATTTAAGGTTCTTGCTTACTTTGTTAATGGTACAGCACAAGATGTAA
TTTTGTGTGACAAGTCCCCCAAGGGTTTGCTAGCTTGTCAATATAACACTGGCAATTTTTCAGATGGCTTTTAT
CCTTTTACTAATACTACTTTAGTTAGGGAAAAGTTCATCGTATATCGTGAAAGTAGTGTTAATACTACTCTGGC
GTTAACTAATTTCACTTTTACTAATGTAAGTAATGCACAGCCTAATAGTGGTGGTGTTAATACTTTTCATTTATAT
CAAACACAAACAGCTCAGAGTGGTTATTATAATTTTAATTTCTCATTTCTGAGTCAGTTTGTGTATAAGGCAAG
TGATTTTATGTATGGGTCTTATCATCCTAGGTGTTCTTTTAGACCAGAAACCATTAATAATGGTTTGTGGTTTAA
TTCCTTGTCAGTTTCTCTTACTTATGGACCCCTACAGGGAGGGTGTAAGCAATCTGTTTTTAGTGGTAAGGCA
ACGTGTTGTTATGCCTACTCTTATAATGGCCCTAGAGTATGTAAAGGTGTTTATTCAGGTGAATTAAGCAAGAC
TTTTGAATGTGGATTGCTGGTTTATGTTACTAAGAGTGATGGCTCTCGTATACAGACTAGAACAGAGCCCTTA
GTATTAACGCAACACAATTATAATAATATTACTTTAGATAAGTGTGTTGAATATAATATATATGGCAGAGTAGGC
CAAGGTTTTATTACTAATGTGACTGATTCTGCTGCTAATTCTAGTTATTTAGCAGATGGTGGGTTAGCTATTTTA
GATACTTCGGGTGCCATAGATGTCTTTGTTGTACAGGGCAGCTATGGTCTTAATTATTACAAGGTCGATCCTTG
TGAAGATGTTAATCAACAGTTTGTAGTGTCTGGTGGTAATATAGTTGGCATTCTTACTTCTAGAAATGAAACA
GGTTCTGAACAGGTTGAGAACCAGTTTTATGTTAAGTTAACCAATAGCTCACATCGTCGTAGGCGT
>CK-CH-SD-HC6-2019.seq
ATGTTGGGGAAGTCACTGTTTTTAGTGACCATTTTGTGTGCACTATGTAGTGCAAATTTGTTTGATTTTGCCA
ATAATTATGTGTACTACTACCAAAGTGCCTTTAGGCCTCCAAACGGATGGCATTTGCAAGGGGGTGCTTATGC
AGTAGTGAATTCTACTAATTATACTAGTAATGCCGGTTCTGCAAGTGGGTGCACTGTTGGTATTATTAAGGAC
GTCTATAATCAAAGTGCGGCTTCCATAGCTATGACAGCACCTTCTCAGGGTATGGCTTGGTCTAAGTCACAAT
TTTGTAGTGCACACTGTAACTTTTCTGAAATTACAGTTTTCGTCACACATTGTTATAGTAGTGGTACAGGGTCT

TGCCCTATAACAGGCATGATTGCACGTGATCATATTCGTATTTCTGCAATGAAAAATGGTTCTTTATTTTATAAC
TTAACAGTTAGCGTATCTAAATACTCTAGGTTTAAGTCTTTTCAATGTGTTAACAACCTCACATCTGTTTATCTA
AATGGTGATCTTGTTTTTACTTCCAACAAAACTACTGATGTTACGTCAGCAGGTGTGTATTTTAAAGCAGGTG
GACCTGTAAATTATAGTGTTATGAAAGAATTTAAGGTTCTTGCTTATTTTGTTAATGGTACAGCACAAGATGTA
ATTTTGTGTGACAAGTCCCCCAAGGGTTTGCTAGCTTGTCAATATAACACTGGCAATTTTTCAGATGGCTTTT
ATCCTTTTACTAATACTACTTTAGTTAGGGAAAAGTTCATCGTATATCGTGAAAGTAGTGTTAATACTACTCTGG
CGTTAACTAATTTCACTTTTACTAATGTAAGTAATGCACAGCCTAATAGTGGTGGTGTTAATACTTTTCATTTAT
ATCAAACACAAACAGCTCAGAGTGGTTATTATAATTTTAATTTGTCATTTCTGAGTCAGTTTGTGTATAAGGCA
AGTGATTTTATGTATGGGTCTTATTATCCTAGGTGTTCTTTTAGACCAGAAACCATTAATAATGGTTTGTGGTTT
AATTCCTTGTCAGTTTCTCTTACTTATGGACCCCTACAGGGAGGGTGTAAGCAATCTGTTTTTAGTGGTAAGG
CAACGTGTTGTTATGCCTACTCTTATAATGGCCCTAGAGCATGTAAAGGTGTTTATTCAGGTGAATTAAGCAA
GACTTTTGAATGTGGATTGCTGGTTTATGTTACTAAGAGTGATGGCTCTCGTATACAAACTAGAACGGAGCCC
TTAGTATTAATGCAACACAATTATAATAATATTACTTTAGATAAGTGTGTTAACTATAATATATATGGCAGAGTGG
GCCAAGGTTTTATTACTAATGTGACTGATTCTGCTGCTAATTTTAGTTATTTAGCAGATGGTGGGTTAGCTATT
TTAGACACTTCGGGTGCCATAGATGTTTTTGTTGCACAGGGCAGCTATGGTCCTAATTATTACAAGGTCAATC
CTTGTGAAGATGTTAACCAACAGTTTGTAGTGTCTGGTGGCAATATAGTTGGCATTCTCACTTCTAGAAATGA
AACAGGTTCTGAACAGGTTGAGAACCAGTTTTATGTTAAGTTAACCAATAGCTCACATCGTCGTAGGCGT
>CK-CH-SD-HC5-2019.seq
ATGTTGGGGAAGTCACTGTTTTTAGTGACCATTTTGTGTGCACTATGTAGTGCAAATTTGTTTGATTTTGCCA
ATAATTATGTGTACTACTACCAAAGTGCCTTTAGGCCTCCAAACGGATGGCATTTGCAAGGGGGTGCTTATGC
AGTAGTGAATTCTACTAATTATACTAGTAATGCCGGTTCTGCAAGTGAGTGCACTGTTGGTATTATTAAGGACG
TCTATAATCAAAGTGCGGCTTCCATAGCTATGACAGCACCTTCTCAGGGTATGGCTTGGTCTAAGTCACAATT
TTGTAGTGCACACTGTAACTTTTCTGAAATTACAGTTTTCGTCACACATTGTTATAGGAGTGGTACAGGGTCT
TGCCCTATAACAGGCATGATTGCACGTGATCATATTCGTATTTCTGCAATGAAAAATGGTTCTTTATTTTATAAC
TTAACAGTTAGCGTATCTAAATACTCTAGGTTTAAGTCTTTTCAATGTGTTAACAACCTCACATCTGTTTATCTA
AATGGTGATCTTGTTTTTACTTCCAACAAAACTACTGATGTTACGTCAGCAGGTGTGTATTTTAAAGCAGGTG
GACCTGTAAATTATAGTGTTATGAAAGAATTTAAGGTTCTTGCTTATTTTGTTAATGGTACAGCACAAGATGTA
ATTTTGTGTGACAAGTCCCCCAAGGGTTTGCTAGCTTGTCAATATAACACTGGCAATTTTTCAGATGGCTTTT
ATCCTTTTACTAATACTACTTTAGTTAGGGAAAAGTTCATCGTATATCGTGAAAGTAGTGTTAATACTACTCTGG
CGTTAACTAATTTCACTTTTACTAATGTAAGTAATGCACAGCCTAATAGTGGTGGTGTTAATACTTTTCATTTAT
ATCAAACACAAACAGCTCAGAGTGGTTATTATAATTTTAATTTGTCATTTCTGAGTCAGTTTGTGTATAAGGCA
AGTGATTTTATGTATGGGTCTTATTATCCTAGGTGTTCTTTTAGACCAGAAACCATTAATAATGGTTTGTGGTTT
AATTCCTTGTCAGTTTCTCTTACTTATGGACCCCTACAGGGAGGGTGTAAGCAATCTGTTTTTAGTGGTAAGG
CAACGTGTTGTTATGCCTACTCTTATAATGGCCCTAGAGCATGTAAAGGTGTTTATTCAGGTGAATTAAGCAA
GACTTTTGAATGTGGATTGCTGGTTTATGTTACTAAGAGTGATGGCTCTCGTATACAAACTAGAACGGAGCCC
TTAGTATTAATGCAACACAATTATAATAATATTACTTTAGATAAGTGTGTTAACTATAATATATATGGCAGAGTGG
GCCAAGGTTTTATTACTAATGTGACTGATTCTGCTGCTAATTTTAGTTATTTAGCAGATGGTGGGTTAGCTATT
TTAGACACTTCGGGTGCCATAGATGTTTTTGTTGCACAGGGCAGCTATGGTCCTAATTATTACAAGGTCAATC
CTTGTGAAGATGTTAACCAACAGTTTGTAGTGTCTGGTGGCAATATAGTTGGCATTCTCACTTCTAGAAATGA
AACAGGTTCTGAACAGGTTGAGAACCAGTTTTATGTTAAGTTAACCAATAGCTCACATCGTCGTAGGCGT
>CK-CH-SD-HC2-2019.seq
ATGTTGGGGAAGTCACTGTTTTTAGTGACCATTTTGTGTGCACTATGTAGTGCAAATTTGTTTGATTTTGCCA
ATAATTATGTGTACTACTACCAAAGTGCCTTTAGGCCTCCAAACGGATGGCATTTGCAAGGGGGTGCTTATGC
AGTAGTGAATTCTACTAATTATACTAGTAATGCCGGTTCTGCAAGTGGGTGCACTGTTGGTATTATTAAGGAC

GTCTATAATCAAAGTGCGGCTTCCATAGCTATGACAGCACCTTCTCAGGGTATGGCTTGGTCTAAGTCACAAT
TTTGTAGTGCACACTGTAACTTTTCTGAAATTACAGTTTTCGTCACACATTGTTATAGTAGTGGTACAGGGTCT
TGCCCTATAACAGGCATGATTGCACGTGATCATATTCGTATTTCTGCAATGAAAAATGGTTCTTTATTTTATAAC
TTTACAGTTAGCGTATCTAAATACTCTAGGTTTAAGTCTTTTCAATGTGTTAACAACCTCACATCTGTTTATCTA
AATGGTGATCTTGTTTTTACTTCCAACAAAACTACTGATGTTACGTCAGCAGGTGTGTATTTTAAAGCAGGTG
GACCTGTAAATTATAGTGTTATGAAAGAATTTAAGGTTCTTGCTTATTTTGTTAATGGTACAGCACAAGATGTA
ATTTTGTGTGACAAGTCCCCCAAGGGTTTGCTAGCTTGTCAATATAATACTGGCAATTTTTCAGATGGCTTTTA
TCCTTTTACTAATACTACTTTAGTTAGGGAAAAGTTCATCGTATATCGTGAAAGTAGTGTTAATACTACTCTGGC
GTTAACTAATTTCACTTTTACTAATGTAAGTAATGCACAGCCTAATAGTGGTGGTGTTAATACTTTTCATTTATAT
CAAACACAAACAGCTCAGAGTGGTTATTATAATTTTAATTTGTCATTTCTGAGTCAGTTTGTGTATAAGGCAA
GTGATTTTATGTATGGGTCTTATTATCCTAGGTGTTCTTTTAGACCAGAAACCATTAATAATGGTTTGTGGTTTA
ATTCCTTGTCAGTTTCTCTTACTTATGGACCCCTACAGGGAGGGTGTAAGCAATCTGTTTTTAGTGGTAAGGC
AACGTGTTGTTATGCCTACTCTTATAATGGCCCTAGAGCATGTAAAGGTGTTTATTCAGGTGAATTAAGCAAG
ACTTTTGAATGTGGATTGCTGGTTTATGTTACTAAGAGTGATGGCTCTCGTATACAAACTAGAACGGAGCCCT
TAGTATTAATGCAACACAATTATAATAATATTACTTTAGATAAGTGTGTTAACTATAATATATATGGCAGAGTGGG
CCAAGGTTTTATTACTAATGTGACTGATTCTGCTGCTAATTTTAGTTATTTAGCAGATGGTGGGTTAGCTATTTT
AGACACTTCGGGTGCCATAGATGTTTTTGTTGCACAGGGCAGCTATGGTCCTAATTATTACAAGGTCAATCCT
TGTGAAGATGTTAACCAACAGTTTGTAGTGTCTGGTGGCAATATAGTTGGCATTCTCACTTCTAGAAATGAAA
CAGGTTCTGAACAGGTTGAGAACCAGTTTTATGTTAAGTTAACCAATAGCTCACATCGTCGTAGGCGT
>CK_CH_GX_YL_LY-CSJ_20200511.seq
ATGTTGGGGAAGTCACTGTTTTTAGTGACCATTTTGTGTGCACTATGTAGTGCAAATTTATTCGATCCTGCTAA
TACTTATGTGTACTACTACCAAAGTGCCTTTAGGCCTCCAAATGGATGGCACCTACAAGGGGGTGCTTATGCA
GTAGTCAATTCCACTAATTATACTAATAATGCCGGTTCTGCACAACATTGCACTGTTGGTGTTATTAAGGACGT
CTATAATCAAAGTGCGGCTTCCATAGCTATGACAGCACCTCTTCAGGGTATGGCTTGGTCTAAGTCACAATTT
TGTAGTGCACACTGTAACTTTTCTGAAATTACAGTTTTTGTCACACATTGTTATAGTAGTGGTAGCGGGTCTTG
TCCTATAACAGGCATGATTGCACGTGATCATATTCGTATTTCTGCAATGAAAAATGGTACTTTATTTTATAATTTA
ACAGTTAGCGTATCTAAATACCCTAATTTTAAATCTTTTCAATGCGTTAATAATCTCACATCTGTTTATCTAAATG
GTGATCTTGTTTTTACTTCCAACAAAACTACTGATGTTACGTCAGCAGGTGTGTATTTTAAAGCAGGTGGACC
TGTAAATTATAGTATTATGAAAGAATTTAAGGTTCTTGCTTACTTTGTTAATGGTACAGCACAAGATGTAATTTT
GTGCGACAATTCCCCCAAGGGTTTGCTAGCTTGTCAATATAACACTGGCAATTTTTCAGATGGCTTTTATCCTT
TTACTAATAGTACTTTAGTTAGGGAAAAGTTCATCGTATATCGCGAAAGTAGTGTTAATACTACTCTGGCGTTA
ACTAATTTCACTTTTACTAATGTAAGTAATGCACAGCCTAATAGTGGTGGTGTTAATACTTTTCATCTATATCAA
ACACAAACAGCTCAGAGTGGTTATTATAATTTTAATTTGTCATTTCTGAGTCAGTTTGTGTATAAGGCAAGTGA
TTTTATGTATGGGTCCTACCACCCTAGTTGTTTTTTTAGACCAGACACCATTAATAGTGGTTTGTGGTTTAATTC
TTTGTCAGTTTCTCTAGCTTACGGACCACTTCAAGGTGGGTGTAAGCAGTCAGTTTTTAGTGGTAGGGCAAC
GTGTTGCTATGCCTACTCTTACAATGGCCCGATAGCCTGTAAAGGTGTTTATTCAGGCGAATTACGGACTAAT
TTTGAATGTGGATTGCTGATTTATGTTACTAAGAGTGATGGTTCTCGTATACAGACTAGAACAGAGCCCTTAG
TATTAACGCAACACAATTATAATAATATTACTTTAGATAAGTGTGTTGACTATAATATATATGGCAGAGTAGGCC
AAGGTTTTATTACTAATGTGACTGATTCTGCTGCTAATTTTAGTTATTTAGCAGATGGTGGGTTAGCTATTTTA
GATACTTCGGGTGCCATAGATGTCTTTGTTGTACAGGGCAGCTATGGTCTTAATTATTACAAGGTCAATCCTTG
TGAAGATGTTAACAAACAGTTTGTAGTGTCTGGTGGCAATATAGTTGGCATTCTTACTTCTAGAAATGAAACA
GGTTCTGAACAGGTTGAGAACCAGTTTTATGTTAAGTTAACCAATAGCTCACATCGTCGCAGGCGT
>CK_CH_GX_WM-LY_HCY20200701.seq
ATGTTGGGGAAGTCACTGTTTTTAGTGACCATTTTGTGTGCACTATGTAGTGCAAATTTATTCGATCCTGCTAA

TACTTATGTGTACTACTACCAAAGTGCCTTTAGGCCTCCAAATGGATGGCACCTACAAGGGGGTGCTTATGCA
GTAGTCAATTCCACTAATTATACTAATAATGCCGGTTCTGCACAACATTGCACTGTTGGTGTTATTAAGGACGT
CTATAATCAAAGTGCGGCTTCCATAGCTATGACAGCACCTCTTCAGGGTATGGCTTGGTCTAAGTCACAATTT
TGTAGTGCACACTGTAACTTTTCTGAAATTACAGTTTTTGTCACACATTGTTATAGTAGTGGTAGCGGGTCTTG
TCCTATAACAGGCATGATTGCACGTGATCATATTCGTATTTCTGCAATGAAAAATGGTACTTTATTTTATAATTTA
ACAGTTAGCGTATCTAAATACCCTAATTTTAAATCTTTTCAATGCGTTAATAATCTCACATCTGTTTATCTAAATG
GTGATCTTGTTTTTACTTCCAACAAAACTACTGATGTTACGTCAGCAGGTGTGTATTTTAAAGCAGGTGGACC
TGTAAATTATAGTATTATGAAAGAATTTAAGGTTCTTGCTTACTTTGTTAATGGTACAGCACAAGATGTAATTTT
GTGCGACAATTCCCCCAAGGGTTTGCTAGCTTGTCAATATAACACTGGCAATTTTTCAGATGGCTTTTATCCTT
TTACTAATAGTACTTTAGTTAGGGAAAAGTTCATCGTATATCGCGAAAGTAGTGTTAATACTACTCTGGCGTTA
ACTAATTTCACTTTTACTAATGTAAGTAATGCACAGCCTAATAGTGGTGGTGTTAATACTTTTCATCTATATCAA
ACACAAACAGCTCAGAGTGGTTATTATAATTTTAATTTGTCATTTCTGAGTCAGTTTGTGTATAAGGCAAGTGA
TTTTATGTATGGGTCCTACCACCCTAGTTGTTTTTTTAGACCAGACACCATTAATAGTGGTTTGTGGTTTAATTC
TTTGTCAGTTTCTCTAGCTTACGGACCACTTCAAGGTGGGTGTAAGCAGTCAGTTTTTAGTGGTAGGGCAAC
GTGTTGCTATGCCTACTCTTACAATGGCCCGATAGCCTGTAAAGGTGTTTATTCAGGCGAATTACGGACTAAT
TTTGAATGTGGATTGCTGATTTATGTTACTAAGAGTGATGGTTCTCGTATACAGACTAGAACAGAGCCCTTAG
TATTAACGCAACACAATTATAATAATATTACTTTAGATAAGTGTGTTGACTATAATATATATGGCAGAGTAGGCC
AAGGTTTTATTACTAATGTGACTGATTCTGCTGCTAATTTTAGTTATTTAGCAGATGGTGGGTTAGCTATTTTA
GATACTTCGGGTGCCATAGATGTCTTTGTTGTACAGGGCAGCTATGGTCTTAATTATTACAAGGTCAATCCTTG
TGAAGATGTTAACAAACAGTTTGTAGTGTCTGGTGGCAATATAGTTGGCATTCTTACTTCTAGAAATGAAACA
GGTTCTGAACAGGTTGAGAACCAGTTTTATGTTAAGTTAACCAATAGCTCACATCGTCGCAGGCGT
>CK_CH_GX_QZ_LY-TFD_20200513.seq
ATGTTGGGGAAGTCACTGTTTTTAGTGACCATTTTGTGTGCACTATGTAGTGCAAATTTATTCGATCCTGCTAA
TACTTATGTGTACTACTACCAAAGTGCCTTTAGGCCTCCAAATGGATGGCACCTACAAGGGGGTGCTTATGCA
GTAGTCAATTCCACTTATTATACTAATAATGCCGGTTCTGCACAACATTGCACTGTTGGTGTTATTAAGGACGT
CTATAATCAAAGTGCGGCTTCCATAGCTATGACAGCACCTCTTCAGGGTATGGCTTGGTCTAAGTCACAATTT
TGTAGTGCACACTGTAACTTTTCTGAAATTACAGTTTTTGTCACACATTGTTATAGTAGTGGTAGCGGGTCTTG
TCCTATAACAGGCATGATTGCACGTGATCATATTCGTATTTCTGCAATGAAAAATGGTACTTTATTTTATAATTTA
ACAGTTAGCGTATCTAAATACCCTAATTTTAAATCTTTTCAATGCGTTAATAATCTCACATCTGTTTATCTAAATG
GTGATCTTGTTTTTACTTCCAACAAAACTACTGATGTTACGTCAGCAGGTGTGTATTTTAAAGCAGGTGGACC
TGTAAATTATAGTATTATGAAAGAATTTAAGGTTCTTGCTTACTTTGTTAATGGTACAGCACAAGATGTAATTTT
GTGCGACAATTCCCCCAAGGGTTTGCTAGCTTGTCAATATAACACTGGCAATTTTTCAGATGGCTTTTATCCTT
TTACTAATAGTACTTTAGTTAGGGAAAAGTTCATCGTATATCGCGAAAGTAGTGTTAATACTACTCTGGCGTTA
ACTAATTTCACTTTTACTAATGTAAGTAATGCACAGCCTAATAGTGGTGGTGTTAATACTTTTCATCTATATCAA
ACACAAACAGCTCAGAGTGGTTATTATAATTTTAATTTGTCATTTCTGAGTCAGTTTGTGTATAAGGCAAGTGA
TTTTATGTATGGGTCCTACCACCCTAGTTGTTCTTTTAGACCAGACACCATTAATAGTGGTTTGTGGTTTAATTC
TTTGTCAGTTTCTCTAGCTTACGGACCACTTCAAGGTGGGTGTAAGCAGTCAGTTTTTAGTGGTAGGGCAAC
GTGTTGCTATGCCTACTCTTACAATGGCCCGATAGCCTGTAAAGGTGTTTATTCAGGCGAATTACGGACTAAT
TTTGAATGTGGATTGCTGATTTATGTTACTAAGAGTGATGGTTCTCGTATACAGACTAGAACAGAGCCCTTAG
TATTAACGCAACACAATTATAATAATATTACTTTAGATAAGTGTGTTGACTATAATATATATGGCAGAGTAGGCC
AAGGTTTTATTACTAATGTGACTGATTCTGCTGCTAATTTTAGTTATTTAGCAGATGGTGGGTTAGCTATTTTA
GATACTTCGGGTGCCATAGATGTCTTTGTTGTACAGGGCAGCTATGGTCTTAATTATTACAAGGTCAATCCTTG
TGAAGATGTTAACAAACAGTTTGTAGTGTCTGGTGGCAATATAGTTGGCATTCTTACTTCTAGAAATGAAACA
GGTTCTGAACAGGTTGAGAACCAGTTTTATGTTAAGTTAACCAATAGCTCACATCGTCGCAGGCGT

>CK_CH_GX_WM-LY_WZR20200810.seq
ATGTTGGGGAAGTCACTGTTTTTAGTGACCATTTTGTGTGCACTATGTAGTGCAAATTTATTCGATCCTGCTAA
TACTTATGTGTACTACTACCAAAGTGCCTTTAGGCCTCCAAATGGATGGTACCTACAAGGGGGTGCTTATGCA
GTAGTCAATTCCACTAATTATACTAATAATGCCGGTTCTGCACAACATTGCACTGTTGGTGTTATTAAGGACGT
CTATAATCAAAGTGCGGCTTCCATAGCTATGACAGCACCTCTTCAGGGTATGGCTTGGTCTAAGTCACAATTT
TGTAGTGCACACTGTAACTTTTCTGAAATTACAGTTTTTGTCACACATTGTTATAGTAGTGGTAGCGGGTCTTG
TCCTATAACAGGCATGATTGCACGTGATCATATTCGTATTTCTGCAATGAAAAATGGTACTTTATTTTATAATTTA
ACAGTTAGCGTATCTAAATACCCTAATTTTAAATCTTTTCAATGCGTTAATAATCTCACATCTGTTTATCTAAATG
GTGATCTTGTTTTTACTTCCAACAAAACTACTGATGTTACGTCAGCAGGTGTGTATTTTAAAGCAGGTGGACC
TGTAAATTATAGTATTATGAAAGAATTTAAGGTTCTTGCTTACTTTGTTAATGGTACAGCACAAGATGTAATTTT
GTGCGACAATTCCCCCAAGGGTTTGCTAGCTTGTCAATATAACACTGGCAATTTTTCAGATGGCTTTTATCCTT
TTACTAATAGTACTTTAGTTAGGGAAAAGTTCATCGTATATCGCGAAAGTAGTGTTAATACTACTCTGGCGTTA
ACTAATTTCACTTTTACTAATGTAAGTAATGCACAGCCTAATAGTGGTGGTGTTAATACTTTTCATCTATATCAA
ACACAAACAGCTCAGAGTGGTTATTATAATTTTAATTTGTCATTTCTGAGTCAGTTTGTGTATAAGGCAAGTGA
TTTTATGTATGGGTCCTACCACCCTAGTTGTTCTTTTAGACCAGACACCATTAATAGTGGTTTGTGGTTTAATTC
TTTGTCAGTTTCTCTAGCTTACGGACCACTTCAAGGTGGGTGTAAGCAGTCAGTTTTTAGTGGTAGGGCAAC
GTGTTGCTATGCCTACTCTTACAATGGCCCGATAGCCTGTAAAGGTGTTTATTCAGGCGAATTACGGACTAAT
TTTGAATGTGGATTGCTGATCTATGTTACTAAGAGTGATGGTTCTCGTATACAGACTAGAACAGAGCCCTTAG
TATTAACGCAACACAATTATAATAATATTACTTTAGATAAGTGTGTTGACTATAATATATATGGCAGAGTAGGCC
AAGGTTTTATTACTAATGTGACTGATTCTGCTGCTAATTTTAGTTATTTAGCAGATGGTGGGTTAGCTATTTTA
GATACTTCGGGTGCCATAGATGTCTTTGTTGTACAGGGCAGCTATGGTCTTAATTATTACAAGGTCAATCCTTG
TGAAGATGTTAACAAACAGTTTGTAGTGTCTGGTGGCAATATAGTTGGCATTCTTACTTCTAGAAATGAAACA
GGTTCTGAACAGGTTGAGAACCAGTTTTATGTTAAGTTAACCAATAGCTCACATCGTCGCAGGCGT
>CK_CH_GX_LG-LY_XQL20200618.seq
ATGTTGGGGAAGTCACTGTTTTTAGTGACCATTTTGTGTGCACTATGTAGTGCAAATTTATTCGATCCTGCTAA
TACTTATGTGTACTACTACCAAAGTGCCTTTAGGCCTCCAAATGGATGGCACCTACAAGGGGGTGCTTATGCA
GTAGTCAATTCCACTAATTATACTAATAATGCCGGTTCTGCACAACATTGCACTGTTGGTGTTATTAAGGACGT
CTATAATCAAAGTGCGGCTTCCATAGCTATGACAGCACCTCTTCAGGGTATGGCTTGGTCTAAGTCACAATTT
TGTAGTGCACACTGTAACTTTTCTGAAATTACAGTTTTTGTCACACATTGTTATAGTAGTGGTAGCGGGTCTTG
TCCTACAACAGGCATGATTGCACGTGATCATATTCGTATTTCTGCAATGAAAAATGGTACTTTATTTTATAATTT
AACAGTTAGCGTATCTAAATACCCTAATTTTAAATCTTTTCAATGCGTTAATAATCTCACATCTGTTTATCTAAAT
GGTGATCTTGTTTTTACTTCCAACAAAACTACTGATGTTACGTCAGCAGGTGTGTATTTTAAAGCAGGTGGAC
CTGTAAATTATAGTATTATGAAAGAATTTAAGGTTCTTGCTTACTTTGTTAATGGTACAGCACAAGATGTAATTT
TGTGCGACAATTCCCCCAAGGGTTTGCTAGCTTGTCAATATAACACTGGCAATTTTTCAGATGGCTTTTATCCT
TTTACTAATAGTACTTTAGTTAGGGAAAAGTTCATCGTATATCGCGAAAGTAGTGTTAATACTACTCTGGCGTT
AACTAATTTCACTTTTACTAATGTAAGTAATGCACAGCCTAATAGTGGTGGTGTTAATACTTTTCATCTATATCA
AACACAAACAGCTCAGAGTGGTTATTATAATTTTAATTTGTCATTTCTGAGTCAGTTTGTGTATAAGGCAAGT
GATTTTAGGTATGGGTCCTACCACCCTAGTTGTTCTTTTAGACCAGACACCATTAATAGTGGTTTGTGGTTTAA
TTCTTTGTCAGTTTCTCTAGCTTACGGACCACTTCAAGGTGGGTGTAAGCAGTCAGTTTTTAGTGGTAGGGC
AACGTGTTGCTATGCCTACTCTTACAATGGCCCGATAGCCTGTAAAGGTGTTTATTCAGGCGAATTACGGACT
AATTTTGAATGTGGATTGCTGATCTATGTTACTAAGAGTGATGGTTCTCGTATACAGACTAGAACAGAGCCCT
TAGTATTAACGCAACACAATTATAATAATATTACTTTAGATAAGTGTGTTGACTATAATATATATGGCAGAGTAG
GCCAAGGTTTTATTACTAATGTGACTGATTCTGCTGCTAATTTTAGTTATTTAGCAGATGGTGGGTTAGCTATT
TTAGATACTTCGGGTGCCATAGATGTCTTTGTTGTACAGGGCAGCTATGGTCTTAATTATTACAAGGTCAATCC

TTGTGAAGATGTTAACAAACAGTTTGTAGTGTCTGGTGGCAATATAGTTGGCATTCTTACTTCTAGAAATGAA
ACAGGTTCTGAACAGGTTGAGAACCAGTTTTATGTTAAGTTAACCAATAGCTCACATCGTCGCAGGCGT
>CK_CH_GX_WM-LY_LZW20200810.seq
ATGTTGGGGAAGTCACTGTTTTTAGTGACCATTTTGTGTGCACTATGTAGTGCAAATTTATTCGATCCTGCTAA
TACTTATGTGTACTACTACCAAAGTGCCTTTAGGCCTCCAAATGGATGGCACCTACAAGGGGGTGCTTATGCA
GTAGTCAATTCCACTAATTATACTAATAATGCCGGTTCTGCACAACATTGCACTGTTGGTGTTATTAAGGACGT
CTATAATCAAAGTGCGGCTTCCATAGCTATGACAGCACCTCTTCAGGGTATGGCTTGGTCTAAGTCACAATTT
TGTAGTGCACACTGTAACTTTTCTGAAATTACAGTTTTTGTCACACATTGTTATAGTAGTGGTAGCGGGTCTTG
TCCTATAACAGGCATGATTGCACGTGATCATATTCGTATTTCTGCAATGAAAAATGGTACTTTATTTTATAATTTA
ACAGTTAGCGTATCTAAATACCCTAATTTTAAATCTTTTCAATGCGTTAATAATCTCACATCTGTTTATCTAAATG
GTGATCTTGTTTTTACTTCCAACAAAACTACTGATGTTACGTCAGCAGGTGTGTATTTTAAAGCAGGTGGACC
TGTAAATTATAGTATTATGAAAGAATTTAAGGTTCTTGCTTACTTTGTTAATGGTACAGCACAAGATGTAATTTT
GTGCGACAATTCCCCCAAGGGTTTGCTAGCTTGTCAATATAACACTGGCAATTTTTCAGATGGCTTTTATCCTT
TTACTAATAGTACTTTAGTTAGGGAAAAGTTCATCGTATATCGCGAAAGTAGTGTTAATACTACTCTGGCGTTA
ACTAATTTCACTTTTACTAATGTAAGTAATGCACAGCCTAATAGTGGTGGTGTTAATACTTTTCATCTATATCAA
ACACAAACAGCTCAGAGTGGTTATTATAATTTTAATTTGTCATTTCTGAGTCAGTTTGTGTATAAGGCAAGTGA
TTTTATGTATGGGTCCTACCACCCTAGTTGTTCTTTTAGACCAGACACCATTAATAGTGGTTTGTGGTTTAATTT
TTTGTCAGTTTCTCTAGCTTACGGACCACTTCAAGGTGGGTGTAAGCAGTCAGTTTTTAGTGGTAGGGCAAC
GTGTTGCTATGCCTACTCTTACAATGGCCCGATAGCCTGTAAAGGTGTTTATTCAGGCGAATTACGGACTAAT
TTTGAATGTGGATTGCTGATTTATGTTACTAAGAGTGATGGTTCTCGTATACAGACTAGAACAGAGCCCTTAG
TATTAACGCAACACAATTATAATAATATTACTTTAGATAAGTGTGTTGACTATAATATATATGGCAGAGTAGGCC
AAGGTTTTATTACTAATGTGACTGATTCTGCTGCTAATTTTAGTTATTTAGCAGATGGTGGGTTAGCTATTTTA
GATACTTCGGGTGCCATAGATGTCTTTGTTGTACAGGGCAGCTATGGTCTTAATTATTACAAGGTCAATCCTTG
TGAAGATGTTAACAAACAGTTTGTAGTGTCTGGTGGCAATATAGTTGGCATTCTTACTTCTAGAAATGAAACA
GGTTCTGAACAGGTTGAGAACCAGTTTTATGTTAAGTTAACCAATAGCTCACATCGTCGCAGGCGT
>CK_CH_ZJ_TZ-LH-CHX_20200420.seqA
TGTTGGGGAAGTCACTGTTTTTAGTGACCATTTTGTGTGCACTATGTAGTGCAAATTTATTCGATCCTGCTAAT
ACTTATGTGTACTACTACCAAAGTGCCTTTAGGCCTCCAAATGGATGGCACCTACAAGGGGGTGCTTATGCA
GTAGTCAATTCCACTAATTATACTAATAATGCCGGTTCTGCACAACATTGCACTGTTGGTGTTATTAAGGACGT
CTATAATCAAAGTGCGGCTTCCATAGCTATGACAGCACCTCTTCAGGGTATGGCTTGGTCTAAGTCACAATTT
TGTAGTGCACACTGTAACTTTTCTGAAATTACAGTTTTTGTCACACATTGTTATAGTAGTGGTAGCGGGTCTTG
TCCTATAACAGGCATGATTGCACGTGATCATATTCGTATTTCTGCAATGAAAAATGGTACTTTATTTTATAATTTA
ACAGTTAGCGTATCTAAATACCCTAATTTTAAATCTTTTCAATGCGTTAATAATCTCACATCTGTTTATCTAAATG
GTGATCTTGTTTTTACTTCCAACAAAACTACTGATGTTACGTCAGCAGGTGTGTATTTTAAAGCAGGTGGACC
TGTAAATTATAGTATTATGAAAGAATTTAAGGTTCTTGCTTACTTTGTTAATGGTACAGCACAAGATGTAATTTT
GTGCGACAATTCCCCCAAGGGTTTGCTAGCTTGTCAATATAACACTGGCAATTTTTCAGATGGCTTTTATCCTT
TTACTAATAGTACTTTAGTTAGGGAAAAGTTCATCGTACATCGCGAAAGTAGTGTTAATACTACTCTGGCGTTA
ACTAATTTCACTTTTACTAATGTAAGTAATGCACAGCCTAATAGTGGTGGTGTTAATACTTTTCATCTATATCAA
ACACAAACAGCTCAGAGTGGTTATTATAATTTTAATTTGTCATTTCTGAGTCAGTTTGTGTATAAGGCAAGTGA
TTTTATGTATGGGTCCTACCACCCTAGTTGTTCTTTTAGACCAGACACCATTAATAGTGGTTTGTGGTTTAATTT
TTTGTCAGTTTCTCTAGCTTACGGACCACTTCAAGGTGGGTGTAAGCAGTCAGTTTTTAGTGGTAGGGCAAC
GTGTTGCTATGCCTACTCTTACAATGGCCCGATAGCCTGTAAAGGTGTTTATTCAGGCGAATTACGGACTAAT
TTTGAATGTGGATTGCTGATTTATGTTACTAAGAGTGATGGTTCTCGTATACAGACTAGAACAGAGCCCTTAG
TATTAACGCAACACAATTATAATAATATTACTTTAGATAAGTGTGTTGACTATAATATATATGGCAGAGTAGGCC

AAGGTTTTATTACTAATGTGACTGATTCTGCTGCTAATTTTAGTTATTTAGCAGATGGTGGGTTAGCTATTTTA
GATACTTCGGGTGCCATAGATGTCTTTGTTGTACAGGGCAGCTATGGTCTTAATTATTACAAGGTCAATCCTTG
TGAAGATGTTAACAAACAGTTTGTAGTGTCTGGTGGCAATATAGTTGGCATTCTTACTTCTAGAAATGAAACA
GGTTCTGAACAGGTTGAGAACCAGTTTTATGTTAAGTTAACCAATAGCTCACATCGTCGCAGGCGT
>CK_CH_SC_CQ-DK_CYX_20200713.seq
ATGTTGGGGAAGTCACTGTTTTTAGTGACCATTTTGTGTGCACTATGTAGTGCAAATTTATTCGATCCTGCTAA
TACTTATGTGTACTACTACCAAAGTGCCTTTAGGCCTTCAAATGGATGGCACCTACAAGGGGGTGCTTATGCA
GTAGTCAATTCCACTAATTATACTAATAATGCCGGTTCTGCACAACATTGCACTGTTGGTGTTATTAAGGACGT
CTATAATCAAAGTGCGGCTTCCATAGCTATGACAGCACCTCTTCAGGGTATGGCTTGGTCTAAGTCACAATTT
TGTAGTGCACACTGTAACTTTTCTGAAATTACAGTTTTTGTCACACATTGTTATAGTAGTGGTAGCGGGTCTTG
TCCTATAACAGGCATGATTGCACGTGATCATATTCGTATTTCTGCAATGAAAAATGGTACTTTATTTTATAATTTA
ACAGTTAGCGTATCTAAATACCCTAATTTTAAATCTTTTCAATGCGTTAATAATCTCACATCTGTTTATCTAAATG
GTGATCTTGTTTTTACTTCCAACAAAACTACTGATGTTACGTCAGCAGGTGTGTATTTTAAAGCAGGTGGACC
TGTAAATTATAGTATTATGAAAGAATTTAAGGTTCTTGCTTACTTTGTTAATGGTACAGCACAAGATGTAATTTT
GTGCGACAATTCCCCCAAGGGTTTGCTAGCTTGTCAATATAACACTGGCAATTTTTCAGATGGCTTTTATCCTT
TTACTAATAGTACTTTAGTTAGGGAAAAGTTCATCGTACATCGCGAAAGTAGTGTTAATACTACTCTGGCGTTA
ACTAATTTCACTTTTACTAATGTAAGTAATGCACAGCCTAATAGTGGTGGTGTTAATACTTTTCATCTATATCAA
ACACAAACAGCTCAGAGTGGTTATTATAATTTTAATTTGTCATTTCTGAGTCAGTTTGTGTATAAGGCAAGTGA
TTTTATGTATGGGTCCTACCACCCTAGTTGTTCTTTTAGACCAGACACCATTAATAGTGGTTTGTGGTTTAATTT
TTTGTCAGTTTCTCTAGCTTACGGACCACTTCAAGGTGGGTGTAAGCAGTCAGTTTTTAGTGGTAGGGCAAC
GTGTTGCTATGCCTACTCTTACAATGGCCCGATAGCCTGTAAAGGTGTTTATTCAGGCGAATTACGGACTAAT
TTTGAATGTGGATTGCTGATTTATGTTACTAAGAGTGATGGTTCTCGTATACAGACTAGAACAGAGCCCTTAG
TATTAACGCAACACAATTATAATAATATTACTTTAGATAAGTGTGTTGACTATAATATATATGGCAGAGTAGGCC
AAGGTTTTATTACTAATGTGACTGATTCTGCTGCTAATTTTAGTTATTTAGCAGATGGTGGGTTAGCTATTTTA
GATACTTCGGGTGCCATAGATGTCTTTGTTGTACAGGGCAGCTATGGTCTTAATTATTACAAGGTCAATCCTTG
TGAAGATGTTAACAAACAGTTTGTAGTGTCTGGTGGCAATATAGTTGGCATTCTTACTTCTAGAAATGAAACA
GGTTCTGAACAGGTTGAGAACCAGTTTTATGTTAAGTTAACCAATAGCTCACATCGTCGCAGGCGT
>CK_CH_SD_LC-SRX3_20200601.seq
ATGTTGGGGAAGTCACTGTTTTTAGTGACCATTTTGTGTGCACTATGTAGTGCAAATTTGTTTGATTCTGCCA
ATAATTATGTGTACTACTACCAAAGTGCCTTTAGGCCTCCAAATGGATGGCATTTGCAAGGGGGTGCTTATGC
AGTAGTGAATTCTACTAATTATACTAGTAATGCCGGTTCTGCAAGTGGGTGCACTGTTGGTATTATTAAGGAC
GTCTATAATCAAAGTGCGGCTTCTATAGCTATGACAGCACCTCCTCAGGGTATGGCTTGGTCTAAGTCACAAT
TTTGTAGTGCACACTGTAACTTTTCTGAAATTACAGTTTTTGTCACACATTGTTATAGTAGTGGTGCAGGGTCT
TGCCCTATAACAGGCATGATTGCACGTGACCATATTCGTATTTCTGCAATGAAAAATGGTTCTTTATTTTATAAC
TTAACAGTTAGCGTATCTAAATACTCTAGGTTTAAGTCTTTTCAATGTGTTAACAACCTCACATCTGTTTATTTA
AATGGTGATCTTGTTTTTACTTCCAATAAAACTACTGATGTTACGTCAGCAGGTGTGTATTTTAAAGCAGGTG
GACCTGTAAATTATAGTGTTATGAAAGAATTTAAGGTTCTTGCTTACTTTGTTAATGGTACAGCACAAGACGT
AATTTTGTGTGACAATTCCCCTAAGGGTTTGCTAGCCTGTCAATATAGTACTGGCAATTTTTCAGATGGCTTCT
ATCCTTTTACTAATAGCACTTTGGTTAGGGACAAGTTCATTGTCTATCGTGAAAGTAGTGTTAATACTACTTTG
ACGTTAACTAATTTCACTTTTACTAATGTAAGTACTGCACAGCCTAATAGTGGTGGTGTTAGTACTTTTCATCTA
TATCAAACACAAACAGCTCAGAGTGGTTATTATAATTTTAATTTGTCATTTCTGAGTCAGTTTGTGTATAAGGC
AAGTGATTTTATGTATGGGTCTTATCATCCTAGGTGTTCTTTTAGACCAGAAACCATTAATAGTGGTTTATGGT
TTAATTCCTTGTCAGTTTCTCTTACTTATGGACCCCTACAGGGAGGGTGTAAGCAATCTGTTTTTAGTGGTAA
GGCAACGTGTTGTTATGCCTACTCTTATAATGGCCCTAGGGCATGTAAAGGTGTCTATTCAGGTGAATTAAGC

AAGACTTTTGAATGTGGATTGCTGGTTTATGTTACTAAGAGTGATGGCTCTCGTATACAAACTAGAACAGAGC
CCTTAGTATTAACGCAACACAATTATAATAATGTTACTTTAGATAAGTGTGTTGACTATAATATATATGGCAGAGT
AGGCCAAGGTTTTATTACTAATGTGACTGATTCTGCTGCTAATTTTAGTTATTTAGCAGACGGTGGGTTAGCTA
TTTTAGATACTTCGGGTGCCATAGATGTCTTCGTTGTACAGGGCAGCTATGGTTTTAATTATTACAAGGTCAAT
CCTTGTGAAGATGTTAACCAACAGTTTGTAGTGTCTGGTGGTAATATAGTTGGCATTCTTACTTCTAGAAATG
AAACAGGTTCTGAACAGGTTGAGAACCAGTTTTATGTTAAGTTAACCAATAGCTCACATCGTCGTAGGCGT
>CK_CH_ZJ_TZ-LH_XZH-3_20201109.seq
ATGTTGGGGAAGTCACTGTTTTTAGTGACCATTTTGTGTGCACTATGTAGTGCAAATTTGTTTGATTTTGCCA
ATAATTATGTGTACTACTACCAAAGTGCCTTTAGGCCTCCAAATGGATGGCATTTGCAAGGGGGTGCTTATGC
AGTAGTGAATTCTACTAATTATACTAGTAATGCCGGTTGCACTGTTGGTATTATTAAGGACGTCTATAATCAAA
GTGCGGCTTCCATAGCTATGACAGCACCTCCTCAGGGTATGGCTTGGTCTAAGTCACAATTTTGTAGTGCACA
CTGTAACTTTTCTGAAATTACAGTCTTTGTCACACATTGTTATAGTAGTGGTGCAGGGTCTTGCCCTATAACAG
GCATGATTGCACGTGATCATATTCGTATTTCTGCAATGAAAAATGGTTCTTTATTTTATAACTTAACAGTTAGCG
TATCTAAATACTCTAGGTTTAAGTCTTTTCAATGTGTTAATAACCTCACATCTGTTTATTTAAATGGTGATCTTGT
TTTTACTTCCAATAAAACTACTGATGTTACGTCAGCAGGTGTGTATTTTAAAGCAGGTGGACCTGTAAATTATA
GTGTTATGAAAGAATTTAAGGTTCTTGCTTACTTTGTTAATGGTACAGCACAAGACGTAATTTTGTGTGACAA
TTCCCCTAAGGGTTTGCTAGCCTGTCAATATAGTACTGGCAATTTTTCAGATGGCTTCTATCCTTTTACTAATAG
TACTCTAGTTAGGGACAAGTTTATTGTCTATCGTGAAAGTAGTGTTAATACTACTTTGACGTTAACTAATTTCA
CTTTTACTAATGTAAGTACTGCACAGCCTAATAGTGGTGGTGTTAGTACTTTTCATCTATATCAAACACAAACA
GCTCAGAGTGGTTATTATAATTTTAATTTGTCATTTCTGAGTCAGTTTGTGTATAAGGCAAGTGATTTTATGTAT
GGGTCTTATCATCCTAGGTGTTCTTTTAGACCAGAAACCATTAATAGTGGTTTATGGTTTAATTCCTTGTCAGT
TTCTCTTACTTATGGACCCCTACAGGGAGGGTGTAAGCAATCTGTTTTTAGTGGTAAGGCAACGTGTTGTTAT
GCCTACTCTTATAATGGCCCTAGGGCATGTAAAGGTGTTTATTCAGGTGAATTAAGCAAGACTTTTGAATGTG
GATTGCTGGTTTATGTTACTAAGAGTGATGGCTCTCGTATACAAACTAGAACAGAGCCCTTAGTATTAACGCA
ACACAATTATAATAATGTTACTTTAGATAAGTGTGTTGACTATAATATATATGGCAGAGTAGGCCAAGGTTTTAT
TACTAATGTGACTGATTCTGCTGCTAATTTTAGTTATTTAGCAGATGGTGGGTTAGCTATTTTAGATACTTCGG
GTGCCATAGATGTCTTTGTTGTACAGGGCATCTATGGTTCTAATTATTACAAGGTCAATCCTTGTGAAGATGTT
AACCAACAGTTTGTAGTGTCTGGTGGTAATATAGTTGGCATTCTTACTTCTAGAAATGAAACAGGTTCTGAAC
AGGTTGAGAACCAGTTTTATGTTAAGTTAACCAATAGCTCACATCGTCATAGGCGT
>CK_CH_GD_SG-RM_ZXG20200628.seq
ATGTTGGGGAAGTCACTGTTTTTAGTGACCATTTTGTGTGCACTATGTAGTGCAAATTTGTTTGATTCTGCCA
ATAATTATGTGTACTACTACCAAAGTGCCTTTAGGCCTCCAGATGGATGGCATTTGCAAGGGGGTGCTTATGC
AGTAGTGAATTCTACTAATTATACTAATAATGCCGGTTCTGCAAGTGAGTGCACTGTTGGTATTATTAAGGATG
TCTATAATCAAAGTGCGGCTTCCATAGCTATGACAGCACCTTCTCAGGGTATGGCTTGGTCTAAGTCACAATT
TTGTAGTGCACACTGTAACTTTTCTGAAATTACAGTTTTCGTTACACATTGTTATAGTAGTGGTACAGGGTCTT
GCCCTATAACAGGCATGATTGCACGTGATCATATTCGTATTTCTGCAATGAAAAATGGTTCTTTATTTTATAACT
TAACAGTTAGCGTATCTAAATACTCTAGGTTTAAGTCTTTTCAATGTGTTAACAACTTCACATCTGTTTATCTAA
ATGGTGATCTTGTTTTTACTTCCAACAAAACTACTGATGTTATGTCAGCAGGTGTGTATTTTAAAGCAGGTGG
ACCTGTAAATTATAGTGTTATGAAAGAATTTAAGGTTCTTGCTTATTTTGTTAATGGTACAGCACAAGATGTAA
TTTTGTGTGACAAGTCCCCCAAGGGTTTGCTAGCTTGTCAATATAACACTGGCAATTTTTCAGATGGCTTTTAT
CCTTTTACTAATACTACTTTAGTTAGGGAAAAGTTCATCGTATATCGTGAAAGTAGTGTTAATACTACTCTGGC
GTTAACTAATTTCACTTTTACTAATGTAAGTAATGCACAGCCTAATAGTGGTGGTGTTAATACTTTTCAGTTATA
TCAAACACAAACAGCTCAGAGTGGTTATTATAATTTTAATTTGTCATTTCTGAGTCAGTTTGTGTATAAGGCAA
GTGATTTTATGTATGGGTCTTATCATCCTAGGTGTTCTTTTAGACCAGAAACCATTAATAATGATTTGTGGTTTA

ATTCCTTGTCAGTTTCTCTTACTTATGGACCCCTACAGGGAGGGTGTAAGCAATCTGTTTTTAGTGGTAAGGC
AACGTGTTGTTATGCCTACTCTTATAATGGCCCTAGAGTATGTAAAGGTGTTTATTCAGGTGAATTAAGCAAG
ACTTTTGAATGTGGATTGCTGGTTTATGTTACTAAGAGTGATGGCTCTCGTATACAAACTAGAAGTGAACCAC
TGGTGTTAACTCAATATAATTATAACAACATTACTTTAAATAAGTGTGTTGAGTATAATATATATGGTAGAGTCG
GTCAAGGTTTTATTACTAATGTAACTGAAGCAACTGCTAATTATAGTTATCTAGCAGATGGTGGTTTAGCTATT
TTAGATACTTCAGGAGCCATAGACATCTTTGTTGTACAAGGTGAACATGGTCTTAATTATTACAAGGTTAATCC
CTGTGAAGATGTAAACCAGCAGTTTGTAGTTTCTGGTGGTAAATTAGTAGGTATTCTTACCTCACGTAATGCA
ACAGGTTCTCAGCCTCTTGAGAATCAATTCTACATTAAACTCACTAAAGAGACACGTCGTTTTAGACGT
>CK_CH_ZJ_TZ-LH_202003.seq
ATGTTGGGGAAGTCACTGTTTTTAGTGACCATTTTGTGTGCACTATGTAGTGCAAATTTGTTTGATTTTGCCA
ATAATTATGTGTACTACTACCAAAGTGCCTTTAGGCCTCCAAATGGATGGCATTTGCAAGGGGGTGCTTATGC
AGTAGTGAATTCTACTAATTATACTAGTAATGCCGGTTCTGCAAGTGAGTGCACTGTTGGTATTATTAAGGACG
TCTATAATCAAAGTGCGGCTTCCATAGCTATGACAGCACCTTCTCAGGGTATGGCTTGGTCTAAGTCACAATT
TTGTAGTGCACACTGTAACTTTTCTGAAATTACAGTTTTCGTCACACATTGTTATAGTAGTGGTACAGGGTCTT
GCCCTATAACAGGCATGATTGCACGTGATCATATTCGTATTTCTGCAATGAAAAATGGTTCTTTATTTTATAACT
TAACAGTTAGCGTATCTAAATACTCTAGGTTTAAGTCTTTTCAATGTGTTAACAACCTCACATCTGTTTATCTAA
ATGGTGATCTTGTTTTTACTTCCAACAAAACTACTGATGTTACGTCAGCAGGTGTGTATTTTAAAGCAGGTGG
ACCTGTAAATTATAGTGTTATGAAAGAATTTAAGGTTCTTGCTTATTTTGTTAATGGTACAGCACAAGATGTAA
TTTTGTGTGACAAGTCCCCCAAGGGTTTGCTTGCTTGTCAATATAACACTGGCAATTTTTCAGATGGCTTTTAT
CCTTTTACTAATACTACTTTAGTTAGGGAAAAGTTCATCGTATATCGTGAAAGTAGTGTTAATACTACTCTGGC
GTTAACTAATTTCACTTTTACTAATGTAAGTAATGCACAGCCTAATAGTGGTGGTGTTAATACTTTTCATTTATAT
CAAACACAAACAGCTCAGAGTGGTTATTATAATTTTAATTTGTCATTTCTGAGTCAGTTTGTGTATAAGGCAA
GTGATTTTATGTATGGGTCTTATCATCCTAGGTGCTCTTTTAGACCAGAAACCATTAATAATGGTTTGTGGTTTA
ATTCCTTGTCAGTTTCTCTTACTTATGGACCCCTACAGGGAGGGTGTAAGCAATCTGTCTTTAGTGGTAAGGC
AACGTGTTGTTATGCCTACTCTTATAATGGCCCTAGAGCATGTAAAGGTGTTTATTCAGGTGAATTAAGCAAG
ACTTTTGAATGTGGATTGCTGGTTTATGTTACTAAGAGTGATGGCTCTCGTATACAAACTAGAACGGAGCCCT
TAGTATTAATGCAACACAATTATAATAATATTACTTTAGATAAGTGTGTTAACTATAATATATATGGCAGAGTGGG
CCAAGGTTTTATTACTAATGTGACTGATTCTGCTGCTAATTTAGTTATTTAGCAGATGGTGGGTTAGCTATTTT
AGACACTTCGGGTGCCATAGATGTTTTTGTTGTACAGGGCAGCTATGGTACTAATTATTACAAGGTCAATCCT
TGTGAAGATGTTAACCAACAGTTTGTAGTGTCTGGTGGCAATATAGTTGGCATTCTTACTTCTAGAAATGAAA
CAGGTTCTGAACAGGTTGAGAACCAGTTTTATGTTAAGTTAACCAATAGCTCACATCGTCGTAGGCGT
>CK_CH_GS_SY-GL-8#_20201229.seq
ATGTTGGGGAAGTCACTGTTTTTAGTGACCATTTTGTGTGCACTATGTAGTGCAAATTTGTTTGATTTTGCCA
ATAATTATGTGTACTACTACCAAAGTGCCTTTAGGCCTCCAAATGGATGGCATTTGCAAGGGGGTGCTTATGC
AGTAGTGAATTCTACTAATTATACTAATAATGCCGGTTCTGCAAGTGAGTGCACTGTTGGTATTATTAAGGACG
TCTATAATCAAAGTGCGGCTTCCATAGCTATGACAGCACCTTCTCAGGGTATGGCTTGGTCTAAGTCACAATT
TTGTAGTGCACACTGTAACTTTTCTGAAATTACAGTTTTCGTTACACATTGTTATAGTAGTGGTACAGGGTCTT
GCCCTATAACAGGCATGATTGCACGTGATCATATTCGTATTTCTGCAATGAAAAATGGTTCTTTATTTTATAACT
TAACAGTTAGCGTATCTAAATACTCTAGGTTTAAGTCTTTTCAATGTGTTAACAACCTCACATCTGTTTATCTAA
ATGGTGATCTTGTTTTTACTTCCAACAAAACTACTGATGTTACGTCAGCAGGTGTGTATTTTAAAGCAGGTGG
ACCTGTAAATTATAGTGTTATGAAAGAATTTAAGGTTCTTGCTTATTTTGTTAATGGTACAGCACAAGATGTAA
TTTTGTGTGACAAGTCCCCCAAGGGTTTGCTAGCTTGTCAATATAACACTGGCAATTTTTCAGATGGCTTTTAT
CCCTTTACTAATACTACTTTAGTTAGGGAAAAGTTCATTGTATATCGTGAAAGTAGTGTTAATACTACTCTGGC
GTTAACTAATTTCACTTTTACTAATGTAAGTAATGCACAGCCTAATAGTGGTGGTGTTAATACTTTTCATTTATAT

CAAACACAAACAGCTCAGAGTGGTTATTATAATTTTAATTTGTCATTTCTGAGTCAGTTTGTGTATAAGGCAA
GTGATTTTATGTATGGGTCTTATTATCCTAGGTGTTCTTTTAGACCAGAAACCATTAATAATGGTTTGTGGTTTA
ATTCCTTGTCAGTTTCACTTACTTATGGACCCCTACAGGGAGGGTGTAAGCAATCTGTTTTTAGTGGTAAGGC
AACGTGTTGTTATGCCTACTCTTATAATGGCCCTAGAGCATGCAAAGGTGTTTATTCAGGCGAATTAAGCAAG
ACTTTTGAATGTGGATTGCTGGTTTATGTTACTAAGAGTGATGGCTCTCGTATACAAACTAGAACGGAGCCCT
TAGTATTAACGCAACACAATTATAATAATATTACTTTAGATAAGTGTGTTAACTATAATATATATGGCAGAGTGG
GCCAAGGTTTTATTACTAATGTGACTGATTCTGCTGCTAATTTTAGTTATTTAGCAGATGGTGGGTTAGCTATT
TTAGACACTTCGGGTGCCATAGATGTTTTTGTTGCACAGGGCAGCTATGGTCCTAATTATTACAAGGTCAATC
CTTGTGAAGATGTTAATCAACAGTTTGTAGTGTCTGGTGGCAATATAGTTGGCATTCTTACTTCTAGAAATGA
AACAGGTTCTGAACAGGTTGAGAACCAGTTTTATGTTAAGTTAACCAATAGCTCACATCGTCGTAGGCGT
>CK_CH_JX_JA-NNY_20201003.seq
ATGTTGGGCAAACCGCTTTTACTAGTGACTCTTTGGTATGCACTATGTAGTGCTTTGCTTTATGATAAAAATAC
TTACGTTTACTACTACCAAAGTGCCTTTAGGCCTGGTCGAGGTTGGCATCTACATGGGGGTGCTTATGCAGTA
GATAAGGTTTTTAATGGAACCAACAATGCAGTCAGTGTATCTGATTGCACTGCTGGTACTTTTTATGAAAGCT
ATAATACTTCTGCTGCTTCTGTAGCCATGACAGTACCACCTGATGGTATGTCTTGGTCAACTTCACAGTTTTGT
ACAGCTCATTGTAACTTCTCAGACTTTACAGTGTTTGTTACGCATTGTTTTAAAAGTCAACAAGGTAGTTGTC
CATTGACAGGTATGATTCCTCAGCATCATATTCGTATTTCTGCTATGAGATCTGGATTTTTGTTTTATAATTTAAC
AGTTAGCGTATCTAAATACCCTAAATTTAAATCGCTTCAATGTGTTGGCAATTCTACATCTGTCTATTTAAATGG
TGATCTTGTTTTCACTTCTAATGAAACAACTCACGTTACGGGTGCAGGCGTTTATTTTAAAAGTGGTGGGCCT
GTAACTTATAAAGTTATGAAAGAAGTTAAAGCCCTAGCCTACTTTATTAATGGTACCGCACAAGAGGTTATTTT
ATGTGATAACTCACCTAGAGGTTTGCTTGCATGTCAGTATAACACTGGTAATTTTTCAGATGGATTCTACCCTT
TTACTAATTCTTCTTTAGTTAAGGATAGGTTTATTGTATATCGAGAAAGTAGCACTAACACTACTTTAGAGTTAA
CTAATTTCACTTTTACTAATGTAAGTAATGCTTCTCCTAATTCAGGTGGCGTTGATACTTTCCAATTATATCAAA
CACATACTGCTCAGGATGGTTATTATAATTTTAATTTATCATTTCTGAGTAGTTTTGTGTATAAACCATCTGATTT
TATGTATGGGTCATACCACCCAAATTGTAATTTTAGACCAGAGAATATTAATAATGGCTTATGGTTTAATTCATT
ATCTGTGTCACTTACTTACGGACCCATTCAAGGTGGTTGTAAGCAATCTGTTTTTAGTAATAAAGCAACTTGTT
GCTATGCTTATTCTTACCGAGGTCCTACTAGATGTAAGGGTGTTTATAGAGGGGAGCTAACGCAATACTTTGA
ATGTGGACTTCTAGTTTATGTAACTAAGAGTGATGGCTCTCGTATACAAACTAGAAGTGAACCACTGGTGTTA
ACTCAATATAATTATAACAACATTACTTTAAATAAGTGTGTTGAGTATAATATATATGGTAGAGTTGGTCAAGGT
TTTATTACTAATGTAACTGAAGCAACTGCTAATTATAGTTATTTAGCAGATGGTGGGTTAGCTATTTTAGATACT
TCGGGTGCCATAGATGTCTTTGTTGTACAGGGCAGCTATGGTCTTAATTATTACAAGGTCAATCCTTGTGAAG
ATGTTAACCAACAGTTTGTAGTGTCTGGTGGCAATATAGTTGGCATTCTTACTTCTAGAAATGAAACAGGTTC
TGAACAGGTTGAGAACCAGTTTTATGTTAAGTTAACCAATAGCTCACATCGTCGCAGGCGT
>CK_CH_JS_NT_HMTS-TNC20200420.seq
ATGTTGGGGAAGTCACTGTTTTTAGTGACCATTTTGTGTGCACTATGTAGTGCAAATTTGTTTGATTCTGCCA
ATAATTATGTGTACTACTACCAAAGTGCCCTTAGGCCTCCAGATGGATGGCATCTGCAAGGGGGTGCTTATGC
AGTAGTGAATTCTACTAATTATACTAGTAATGCCGGTTCTGCAAGTGAGTGCACTGTTGGTATTATTAAGGACG
TCTATAATCAAAGTGCGGCTTCCATAGCTATGACAGCACCTCTTCAGGGTATGGCTTGGTCTAAGTCACAATT
TTGTAGTGCACACTGTAACTTTTCTGAAATTACAGTCTTTGTCACACATTGTTATAGTAGTGGTGCAGGGTCTT
GCCCTATAACAGGCATGATTGCACGTGATCATATTCGTATTTCTGCAATGAAAAATGGTTCTTTATTTTATAACT
TAACAGTTAGCGTATCTAAATACTCTAGGTTTAAGTCTTTTCAATGTGTTAATAACCTCACATCTGTTTATTTAAA
TGGTGATCTTGTTTTTACTTCCAATAAAACTACTGATGTTACGTCAGCAGGTGTGTATTTTAAAGCAGGTGGA
CCTGTAAATTATAGTGTTATGAAAGAATTTAAGGTTCTTGCTTACTTTGTTAATGGTACAGCACAGGACGTAAT
TTTGTGTGACAATTCCCCTAAGGGTTTGCTAGCCTGTCAATATAGTACTGGCAATTTTTCAGATGGCTTCTATC

CTTTTACTAATAGTACTTTAGTTAGGGACAAGTTCATTGTCTATCGTGAAAGTAGTGTTAATACTACTTTGACG
TTAACTAATTTCACTTTTACTAATGTAAGTACTGCACAGCTAATAGTGGTGGTGTTAGTACTTTTCATCTATATC
AAACACAAACAGCTCAGAGTGGTTATTATAATTTTAATTTGTCATTTCTGAGTCAGTTTGTGTATAAGGCAAGT
GATTTTATGTATGGGTCTTATCATCCTAGGTGTTCTTTTAGACCAGAAACCATTAATAGTGGTTTATGGTTTAAT
TCCTTGTCAGTTTCTCTTACTTATGGACCCCTACAGGGAGGGTGTAAGCAATCTGTTTTTAGTGGTAAGGCAA
CGTGTTGTTATGCCTACTCTTATAATGGCCCTAGGGCATGTAAAGGTGTTTATTCAGGTGAATTAAGCAAGAC
TTTTGAATGTGGATTGCTGGTTTATGTTACTAAGAGTGATGGCTCTCGTATACAAACTAGAACAGAGCCCTTA
GTATTAATGCAACACAATTATAATAATATTACTTTAGATAAGTGTGTTGACTATAATATATATGGCAGAGTAGGC
CAAGGTTTTATTACTAATGTGACTGATTCTGCTGCTAATTTTGGTTATTTAGCAGATGGTGGGTTAGCTATTTT
AGATACTTCGGGTGCCATAGATGTCTTTGTTGTACAGGGCAGCTATGGTCTTAATTATTACAAGGTCAATCCTT
GTGAAGATGTTAACCAACAGTTTGTAGTGTCTGGTGGCAATATAGTTGGCATTCTTACTTCTAGAAATGAAAC
AGGTTCTGAACAGGTTGAGAACCAGTTTTATGTTAAGTTAACCAATAGCTCACATCGTCGTAGGCGT
>CK_CH_SD_LY-YP_2020.seq
ATGTTGGGGAAGTCACTGTTTTTAGTGACCATTTTGTGTGCACTATGTAGTGCAAATTTGTTTGATTTTGCCA
ATAATTATGTGTACTACTACCAAAGTGCCTTTAGGCCTCCAAATGGATGGCATTTGCAAGGGGGTGCTTATGC
AGTAGTGAATTCTACTAATTATACTAGTAATGCCGGTTCTGCAAGGGAGTGCACTGTTGGTATTATTAAGGAC
GTCTATAATCAAAGTGCGGCTTCCATAGCTATGACAGCACCCTCTCAGGGTATGGCTTGGTCTAAGTCACAAT
TTTGTAGTGCACACTGTAACTTTTCTGAAATTACAGTTTTCGTCACACATTGTTTTAGTAGTGGTACAGGGTCT
TGCCCTATAACAGGCATGATTGCACGTGATCATATTCGTATTTCTGCAATGAAAAATGGTTCTTTATTTTATAAC
TTAACAGTTAGCGTATCTAAATACTCTAGGTTTAAGTCTTTTCAATGTGTTAACAACCTCACATCTGTTTATCTA
AATGGTGATCTTGTTTTTACTTCCAACAAAACTACTGATGTTACGTCAGCAGGTGTGTATTTTAAAGCAGGTG
GACCTGTAAATTATAGTGTTATGAAAGAATTTAAGGTTCTTGCTTATTTTGTTAATGGTACAGCACAAGATGTA
ATTTTGTGTGACAAGTCCCCCAAGGGTTTGCTAGCTTGTCAATATAACACTGGCAATTTTTCAGATGGCTTTT
ATCCTTTTACTAATACCACTTTAGTTAGGGAAAAGTTCATCGTATATCGTGAAAGTAGTGTTAATACTACTCTG
GCGTTAACTAATTTCACTTTTACTAATGTAAGTAATGCACAGCCTAATAGTGGTGGTGTTAATAATTTTCATTTA
TATCAAACACAAACAGCTCAGAGTGGTTATTATAATTTTAATTTGTCATTTCTGAGTCAGTTTGTGTATAAGGC
AAGTGATTTTATGTATGGGTCTTATCATCCTAGGTGTTCTTTTAGACCAGAAACCATTAATAATGGTTTGTGGT
TTAATTCCTTGTCAGTTTCTCTTACTTATGGACCCCTACAGGGAGGGTGTAAGCAATCTGTTTTTAGTGGTAA
GGCAACGTGTTGTTATGCCTACTCTTATAATGGCCCTAGAGTATGCAAAGGTGTTTATTCAGGTGAATTAAGC
AAGACTTTTGAATGTGGATTGCTGGTTTATGTTACTAAGAGTGATGGCTCTCGTATACAAACTAGAACGGAG
CCCTTAGTATTAACGCAACACAATTATAATAATATTACTTTAGATAAGTGTGTTAACTATAATATATATGGCAGAG
TGGGCCAAGGTTTTATTACTAATGTGACTGATTCTGCTGCTAATTTTAGTTATTTAGCAGATGGTGGGTTAGCT
ATTTTAGACACTTCGGGTGCCATAGATGTTTTTGTTGCACAGGGCAGCTATGGTCCTAATTATTACAAGGTCA
ATCCTTGTGAAGATGTTAACCAACAGTTTGTAGTGTCTGGTGGCAATATAGTTGGCATTCTTACTTCTAGAAAT
GAAACAGGTTCTGAACAGGTTGAGAACCAGTTTTATGTTAAGTTAACCAATAGCTCACATCGTCGTAGGCGT
>CK_CH_CQ_BSDK-WSQ_20200818.seq
ATGTTGGGGAAGTCACTGTTTTTAGTGACCATTTTGTGTGCACTATGTAGTGCAAATTTGTTTGATTTTGCCA
ATAATTATGTGTACTACTACCAAAGTGCCTTTAGGCCTCCAACTGGATGGCATTTGCAAGGGGGTGCTTATGC
AGTAGTGAATTCTACTAATTATACTAATAATGCCGATTCTGCAAGTGGGTGCACTGTTGGTATTATTAAGGACG
TCTATAATCAAAGTGCGGCTTCCATAGCTATGACAGCACCTTCTCAGGGTATGGCTTGGTCTAAGTCAGAATT
TTGTAGTGCACACTGTAACTTTTCTGAAATTACAGTTTTCGTCACACATTGTTATAATAGTGGTCCAGGGTCTT
GCCCTATAACAGGCATGATTCCAAGTGGTCATATTCGTATTTCTGCAATGAAAAATGGTTCTTTATTTTATAACT
TAACAGTTAGCGTATCTAAATACTCTAGGTTTAAGTCTTTTCAATGTGTTAACAACCTCACATCTGTTTATCTAA
ATGGTGATCTTGTTTTTACTTCCAACAAAACTACTGATGTTACGTCAGCAGGTGTGTATTTTAAAGCAGGTGG

ACCTGTAAATTATAGTGTTATGAAAGAATTTAAGGTTCTTGCTTATTTTGTTAATGGTACAGCACAAGATGTAA
TTTTGTGTGACAAGTCCCCCAAGGGTTTGCTAGCTTGTCAATATAACACTGGCAATTTTTCAGATGGCTTTTAT
CCTTTTACTAATACCACTTTAGTTAGGGAAAAGTTCATCGTATATCGTGAAAGTAGTGTTAATACTACTCTGGC
GTTAACTAATTTCACTTTTACTAATGTAAGTAATGCACAGCCTAATAGTGGTGGTGTTAATACTTTTCATTTATAT
CAAACAAAAACAGCTCAGAGTGGTTATTATAATTTTAATTTGTCATTTCTGAGTCAGTTTGTGTATAAGGCAA
GTGATTTTATGTATGGGTCTTATCATCCTAGGTGTTCTTTTAGACCAGAAACCATTAATAATGGTTTGTGGTTTA
ATTCCTTGTCAGTTTCTCTTACTTATGGACCCCTACAGGGAGGGTGTAAGCAATCTGTTTTTAGTGGTAAGGC
AACGTGTTGTTATGCCTACTCTTATAATGGCCCTAGAGCATGTAAAGGTGTTTATTCAGGTGAATTAAGCAATA
CTTTTGAATGTGGATTGCTGGTTTATGTTACTAAGAGTGATGGCTCTCGTATACAAACTAGAATGGAGCCCTT
AGTATTAATGCAACACAATTATAATAATATTACTTTAGATAAGTGTGTTGACTATAATATATATGGCAGAGTGGG
CCAAGGTTTTATTACTAATGTGACTGATTCTGCTGCTAATTTTAGTTATTTAGCAGATGGTGGGTTAGCTATTTT
AGACACTTCGGGTGCCATAGATGTTTTTGTTGTACAGGGCAGCTATGGTCTTAATTATTACAAGGTCAATCCT
TGTGAAGATGTTAATCAACAGTTTGTAGTGTCTGGTGGCAATATAGTTGGCATTCTTACTTCTAGAAATGAAA
CAGGTTCTGAACAGGTTGAGAACCAGTTTTATGTTAAGTTAACCAATAGCTCACATCGTCGTAGGCGT
>CK_CH_GD_QY_HHH-45d_20201113.seq
ATGTTGGAGAAGTTACTGTTTTTAGTGACCATTTTGTGTGCACTATGTAGTGCAAATTTGTTTGATGCTGATAA
TAGTTATGTGTACTACTACCAGAGTGGATTTAGACCTCCTTTAGGTTGGCACCTTTATGGTGGTGCGTATGCA
GTAGAACGGTTTTTTAATGAAACCAGCAATGCAGGCTCTGGTGACTGTACTGCTGGAGCCATTGTACATAGT
TTAAATGTTACTGCAAGTGCAGTTGCGATTACTACACCTGTTAATGGCATGCATTGGTCATCTAGTAGAGGAG
TGTGTTCAATACATTGCAATTTTAGTACAATTGTTGTTTTTGTTACACATTGTTTTAAAAATGGACAAGGAATAT
GTCCCTTGACAGGTAAATTAAAGGAGGGTGATATTCGTATTGGTGTTCTAGATAGTAGTGGTAATTCTATTTTT
AATAAAACAGTTACCACTTCTAGTTATAGTAAATTTAAATCATTACATTGCGTTAACAATTTCACTTCTGTATATT
TAAATGGTGATCTTGTTTACACGTCTAATGAAACTTTAGATATTATTGGTTTTGGTGTACATTTTAAGACAGGA
GGACCTGTTACTTATAAAATTATGAAAGAACATAAGGTTCTAGCATATTTTGAAAATGGTACTGCACATGACAT
TATTTTATGTGATGACAGTCCCCGTGGTAGGTTAGCTTGTCAGTATAATACAGGCAATTTTTCTGACGGTTTGT
ACCCTTTTAGCGTAAGCAGTGAAGTTAATGAAACTTTTATAGTTTTTGAAAAGAATACAGAAACTACTATGCT
TACATTAAATAATTTCACTTTTTTTAATCAGAGTGGGGCTCAACCTAATCAAAAGGAACCTTCACCTGGTGTTT
CAAATTTTGTGTATTATCAACAGATTAGTGCTGTTCCTGGTTATAATAATTTTAATTTTTCCTTTTTGAGTTCTTT
TACTTATTTAAGTAGTGATTATACGAGAGGTTCTTTTCACCCAAGTTGTACTTTTAGGCCTGAAGATATTAATA
AAAATCGCAGGTTTAATCATTTGTCTATATCTTTATCTTATGGTCCTCGTAATGGAGGCTGTAAGCAAGCATGC
TTTAATACTAGGAGTTCATGTTGTTGTTTCATGTACTCTTATAATGGTCAACCTTTTTGTAAAGGTGTGTATAGT
GGTGATTTAAATCAAGATTTTGAGTGCGTATTGCTTGTGTTTATTAATCATAGCCCAGGCAGTCGTATATTTAC
TTCTGAAACAGTACCTACTGTCACTGCTAATTTTGTAAATAATGTGGTTTTAGATAGGTGTGTTGATTATAATAT
CTATGTTAGATA
>CK_CH_GD_QY-TN_20201109.seq
ATGTTGGAGAAGTTACTGTTTTTAGTGACCATTTTGTGTGCACTATGTAGTGCAAATTTGTTTGATGCTGATAA
TAGTTATGTGTACTACTACCAGAGTGGATTTAGACCTCCTACTGGTTGGCACCTTTATGGTGGTGCGTATGCA
GTAGAACAGTTTTTTAATGAAACCAGCAATGCAGGCTCTAGTGAATGTACTGCTGGAGCTATTGTACATAGTT
TAAATGTTAGTGCAAGTGCAGTTGCGATTACTACACCTGTTAATGGCATGCATTGGTCATCTATTAATGGAGTG
TGTTCAACGCATTGCAATTTTAGTACAATTGTTGTTTTTGTTACACATTGTTTTAAAAATGGACAAGGAATATG
TCCCTTGACAGGTAGATTAAGGGAGGGTGAGATTCGTATTGGTGTTCTAGATAGTAGTGGTAATTCTATTTTT
AATAAAACAGTTACCACTTCTAGTTATAGTAAATTCAAATCATTACATTGTGTTAACAATTTCACTTCTGTGTAT
TTAAACGGTGATCTTGTTTACACGTCTAATGAAACTTCTGATATTACCGGTTTTGGTGTACATTTTAAGACAGG
AGGTCCTGTTACTTATAAAATTATGAAAGAACATAAGGTTCTAGCATATTTTGAAAATGGTACTGCACATGACA

TTATTTTATGTGATGACAGTCCCCGTGGTAGGTTAGCTTGTCAGTATAATACAGGCAATTTTTCTGACGGTTTG
TACCCTTTTAGCGTAAGCAGTGAAGTTAATGAAACTTTTATAGTTTTTGAAAAGAATACAGAAACTACTATGC
TTACATTAAATAATTTCACTTTTTTTAATCAGAGTGGGGCTCAACCTAACCAAAGGGAACCTTCACCTGGTGT
TTCAAATTTTGTATATTATCAACAGATTAGTGCTGTTCCTGGTTATAATAATTTTAATTTTTCCTTTTTGCGTTCT
TTTACTTATTTAAGTAGTGATTATATGAGGGGTTCTTTTCACCCAAGTTGTACTTTTAGGCCTGAAGATATTAAT
AAAAATCGCAGGTTTAATCATTTGTCTATATCCTTATCTTACGGTCCTCGTAATGGAGGCTGTAAGCAAGCATG
CTTTAATACTAGGAGTTCATGTTGTTGTTTCATGTACTCTTATAATGGTCAACCTCTTTGTAAAGGTGTGTATAG
TGGTGATTTAAATCAAGATTTTGAGTGCGTATTGCTTGTGTTTATTAAACATAGCCCAGGCAGTCGTATATTTA
CTTCTGAAACAGTACCTACTTTCACTGCTAATTTTGTAAATAATGTGGTTTTAGATAGGTGTGTTGATTATAATA
TCTATGTTAGTTTAGGCAGGTTTTTTATTTCTAATATAACTGATTCTGTGAAGGATGCAAATTATTTGGATTCTT
CTGGTTTAGCTATATTGGATCAATCGGGTGCTGTTGACACTTTCATGATAAAAGGTCAATTAGGACCTAATTAT
TATAAAGTTAATCCATGTAAAGATGTTAATCAACAATATGTTGTTTCGGGCGGCAATATAGTCGGCCTTCTGAC
ATCTATAAATAGTAGTGGTTCCCAGTTACTAGAGGACCAGTACTATGTTAGATTAACCAATACCTCACATAGGC
GTAAGCGT
>CK_CH_GD_SG_RM-NZF_20201228.seq
ATGTTGGAGAAGTTACTGTTTTTAGTGACCATTTTGTGTGCACTATGTAGTGCAAATTTGTTTGATGCCGATAA
TAGTTATGTGTACTACTACCAGAGTGGATTTAGACCTTCTACAGGTTGGCACCTTTATGGTGGTGCGTATGCA
GTAGAACGGTTTTTTAATGAAACCAGCAATGCAGGCTCTCATGAATGTACTGCTGGAGCTATTGTACATAGTT
TGAATGTTAGTGCAAGTGCAGTTGCGATTACTACACCTGTTAATGGCATGCGTTGGTCATCTAGTAATGGAGT
GTGTTCAATACATTGCAATTTTAGTACAATTGTTGTTTTTGTTACACATTGTTTTAAAAATGGACAAGGAATAT
GTCCCTTGACAGGTAAATTAAGGGAGGGTGATATTCGTATTGGTGTTCTAGATAGTAGTGGTAATTCTATTTTT
AATAAAACAGTTACCACTTCTAATTATAGTAAATTTAAATCATTACATTGTGTTAACAATTTCACTTCTGTGTATT
TAAACGGTGATCTTGTTTACACGTCTAATGAAACTTCTGATATTATTGGTTTTGGTGCACATTTTAAGACAGGA
GGTCCTGTTACTTATAAAATTATGAAAGAACATAAGGTTCTAGCATATTTTGAAAATGGTACTGCACATGACAT
TATTTTATGTGATGACAGTCCCCGTGGTAGGTTAGCTTGTCAGTATAATACAGGCAATTTTTCTGACGGTTTGT
ACCCTTTTAGCGTAAGCAGTGAAGTTAATGAAACTTTTATAGTTTTTGAAAAGAATACAGAAACTACTATGCT
TACATTAAATAATTTCACTTTTTTTAATCAGAGTGGGGCTCAACCTAACCAAAGGGAACCTTCACCTGGTGTT
TCAAATCTTGTATATTATCAACAGATTAGTGCTGTTCCTGGTTATAATAATTTTAATTTTTCCTTTTTGCGTTCTT
TTACTTATTTAAGTAGTGATTATATGAGGGGTTCTTTTCACCCAAGTTGTACTTTTAGGCCTGAAGATATTAATA
AAAATCGCAGGTTTAATCATTTGTCTATATCTTTATCTTACGGTCCTCGTAATGGAGGCTGTAAGCAAGCATGC
TTTAATACTAGGAGTTCATGTTGTTGTTTCATGTACTCTTATAATGGTCAACCTCTTTGTAAAGGTGTGTATAGT
GGTGATTTAAATCAAGATTTTGAGTGCGTATTGCTTGTGTTTATTAAACATAGCCCAGGCAGTCGTATATTTAC
TTCTGAAACAGTACCTACTTTCACTGCTAATTTTGTAAATAATGTGGTTTTAGATAGGTGTGTTGATTATAATAT
CTATGTTAGATATGGCAAGTGTTGTATT
>CK_CH_CQ_BS-DK_WXL20200504.seq
ATGTTGGGGAAGTCACTGTTTTTAGTGACCATTTTGTGTGCACTATGTAGTGCAAATTTGTTTGATCCTGCTAA
TACTTATGTGTACTACTACCAAAGCGCCTATAGACCACCAAATGGATGGCATTTGCAAGGGGGTGCTTATGCA
GTAGTGAATTCTACTAATAAATTTAATAATGCAGGCTCCGCCAGCGAGTGTTCTGTAGGTGTCCTCTTTAATTA
TACTAACGGAAATGACGTTGGTTATAATAATAGTGCTTCTTCTGTAGCCATGACAGCACCGGCTTCTGGTATGT
CTTGGTCTAAAAAACAATTTTGTACTGCCCATTGTAATTTTTCAGATTTTACAGTGTTTGTTACACATTGTTTTG
CAAATTCTTGTCCTTTAACAGGTAGGATAGAGGAAAACCATATTCGTATTTCTGCTATGAGAAATGGTTCTCTA
TTTTATAATTTAACAGTTAGTGTATCCAAATACCCTAAATTTAAATCGCTTCAATGTGTCAATAATTTCACTTCTG
TTTACTTAAATGGTGACCTTGTTTTTACTTCTAACGAAACCACTGATGTTATAGGTGCTGGTGTGTATTTTAAA
GCAGGTGGGCCTATAACCTATAAAGTTATGAAGGAATTTAAGGTTTTGGCTTATTTTGTTAATGGTACTGCAC

AAGATGTCATTTTGTGTGATGACACACCTAAAGGTTTGCTAGCATGTCAATATAATACTGGCAATTTCTCAGAT
GGTTTTTATCCTTTTACTAATAGTAGCTTAGTTAAGCAAAGGTTTATTGTTTATCGTGAGAATAGTGTTAATACT
ACTCTTACTTTAACCAATTACACCTTTCATAATGAGACTAATGCTCAGCCTAATTCAGGTGATGTTCATACTATC
TCAACTTATCAAACACAAACTGCTCAGAGTGGTTATTATAATTTTAATTTATCATTTCTGAGTGGTTTTGTGTAT
AAAGCTTCTGATTATATGTATGGGTCCTACCACCCACGATGTAGTTTTAGACCAGAAACTATTAATAATGGCTT
GTGGTTTAATTCACTGTCAGTCTCATTAGCCTATGGCCCCCTTCAAGGTGGGTGTAAGCAATCAGTTTTTCAA
GGCAGAGCTACTTGTTGTTATGCGTATTCCTATAACGGACCACATATTTGTAAAGGTGTTTATAGTGGTCAGTT
ATTACAAAATTTTGAATGTGGACTGTTGGTTTATGTTACTAAGAGTGATGGCTCTCGTATACAAACAGCCACA
AAACCACCGGTCATAACTCAACACAATTATAATAATATTACTTTAAATACTTGTGTTGAGTACAATATATATGGC
AGAGTTGGCCAAGGCTTTATTACTAATGTAACTGACTCCGCAGCTAGCTATAATTACTTAGCAGATGCTGGAT
TGGCAATTTTAGATACTTCAGGTGCCATAGACACTTTCGTTGTACAAGGTGAATATGGTCCCAATTATTATAAG
GTTAACCCTTGTGAAGATGTTAACCAGCAGTTTGTAGTGTCAGGCGGTAAGTTAGTAGGCATCCTGACTTCT
CGTAATGAAACTGGTTCTCAGCCTCTTGAAAATCAGTTTTATATTAAGTTAACTAATGGAAGCAGTCGTTTTA
GACGT
>CK_CH_JS_CZLH_PGZ_20201221.seq
ATGTTGGTGGAGTCAACGTTTTTAGTGACTCTTTTGTTTGTACTATCTAGTGCTATTTTGTATGATAATGATACG
TACGTTTACTACTACCAGAGCGCCTTCAGACCGTTTGACGGTTGGCATTTACATGGTGGCGCTTATGCAGTAG
TAAATGTTTCTTCACAAACTAACAATGCAGGTGCAGCTTCACAATGCACTGTTGGTATTATTAGTGGTGATAA
AGTTGTTAATGCCTCTTCTATAGCTATGACAGCACCTGTAGGTCAAGGTATGCAGTGGTCTAAGTTACAATTTT
GTACTGCACACTGCAATTTTTCTGATTTTACAGTGTTTGTTACACATTGCTATGCCTCGGGCACCGGTAAATGT
CCTTTAACGGGCCTTATTCCACAAGGTCATATTCGTATTTCTGCTATGCGGAATCATACTTTATTCTATAATTTAA
CAGTTAGTGTATCTAAGTACCCTACTTTTAAATCTTTGCAATGCGTTGATAATTTCACATCTGTTTACTTAAATG
GTGACCTTGTCTTCACTTCTAATCAGACGACAGATGTTATAAGTGCAGGTGTGTACTTTAAAGCAGGTGGGC
CTATAACCTATAAAGTTATGAAGGAATTTAAGGTTTTGGCTTATTTTGTTAATGGTACTGCACAAGATGTTATTT
TGTGTGATGACACACCTAGAGGTTTGCTAGCATGTCAATATAATACTGGCAATTTCTCAGATGGTTTTTATCCT
TTTACTAATAGTAGCTTAGTTAAGCAAAGGTTTGTTGTTTATCGTGAGAATAGTGTTAATACTACTCTTACTTTA
ACCAATTACACCTTTCATAATGAGACTAATGCCCAGCCTAATTCAGGTGGTGTCCATAGTATCTCAACTTATCA
AACACAAACTGCTCAGAGTGGTTATTATAATTTTAATTTATCATTTCTGAGTAGTTTTGTGTATAAAGATTCTGA
TTATATGTATGGGTCCTACCACCCACTATGTAGTTTTAGACCAGAAACTATTAATAATGGCTTGTGGTTTAATTC
ACTGTCAGTCTCATTAGCTTATGGCCCCCTTCAAGGTGGGTGTAAGCAATCAGTTTTTCAAGGCAGAGCTAC
TTGTTGTTATGCGTATTCCTATAACGGACCACGTATGTGTAAAGGTGTTTATAGTGGTGAGTTATTAAAAGATT
TTGAATGTGGACTGTTGGTTTATGTTACTAAGAGTGATGGCTCTCGTATACAAACAGCCACAAAACCACCGGT
CATAACTCAACACAATTATAATAACATTACTTTAAATACTTGTGTTGAGTACAATATATATGGCAGAGTTGGCCA
AGGTTTTATTACTAATGTAACTGACTCCGCAGCTAGCTATAATTACTTAGCAGATGCTGGATTGGCAATTTTAG
ATACTTCAGGTGCCATAGACACTTTCGTTGTACAAGGTGAATATGGTCCCAATTATTATAAGGTTAACCCTTGT
GAAGATGTTAATCAGCAGTTTGTAGTGTCGGGCGGTAAGTTAGTAGGCATTCTGACTTCTCGTAATGAAACT
GGTTCTCAGCCTCTTGAAAATCAGTTTTATATTAAGTTAACTAATGGAAGTCGTCGTTTTAGACGT
>CK_CH_GX_YL_BSWY-LC_20200422.seq
ATGTTGGTGGAGTCAACGTTTTTAGTGACTCTTTTGTTTGTACTATCTAGTGCTACTTTGTATGATAATGATACG
TACGTTTACTACTACCAGAGCGCCTTCAGACCGTTTGACGGTTGGCATTTACATGGTGGCGCTTATGCAGTAG
TAAATGTTTCTTCACAAACTAACAATGCAGGTGCAGCTTCAGAATGCACTGTTGGTATTATTAGTGGTGATAA
AGTTGTTAATGCCTCTTCTATAGCTATGACAGCACCTGTAGGTCAAGGTATGCAGTGGTCTAAGTTACAATTTT
GTACTGCACACTGCAATTTTTCTGATTTTACAGTGTTTGTTACACATTGCTATGCCTCGGGCACCGGTAAATGT
CCTTTAACGGGCCTTATTCCACAAGGTCATATTCGTATTTCTGCTATGCGGAATCATACTTTATTCTATAATTTAA

CAGTTAGTGTATCTAAGTACCCTACTTTTAAATCTTTGCAATGCGTTGATAATTTCACATCTGTTTACTTAAATG
GTGACCTTGTCTTCACTTCTAATCAGACGACAGATGTTATAAGTGCAGGTGTGTACTTTAAAGCAGGTGGGC
CTATAACCTATAAAGTTATGAAGGAATTTAAGGTTTTGGCTTATTTTGTTAATGGTACTGCACAAGATGTTATTT
TGTGTGATGACACACCTAGAGGTTTGCTAGCATGTCAATATAATACTGGCAATTTCTCAGATGGTTTTTATCCT
TTTACTAATAGTAGCTTAGTTAAGCAAAGGTTTGTTGTTTATCGTGAGAATAGTGTTAATACTACTCTTACTTTA
ACCAATTACACCTTTCATAATGAGACTAATGCCCAGCCTAATTCAGGTGGTGTCCATACTATCTCAACTTATCA
AACACAAACTGCTCAGAGTGGTTATTATAATTTTAATTTATCATTTCTGAGTAGTTTTGTGTATAAAGATTCTGA
TTATATGTATGGGTCCTACCACCCACTATGTAGTTTTAGACCAGAAACTATTAATAATGGCTTGTGGTTTAATTC
ACTGTCAGTCTCATTAGCTTATGGCCCCCTTCAAGGTGGGTGTAAGCAATCAGTTTTTCAAGGCAGAGCTAC
TTGTTGTTATGCGTATTCCTATAACGGACCACGTATGTGTAAAGGTGTTTATAGTGGTGAGTTATTAAAAGATT
TTGAATGTGGACTGTTGGTTTATGTTACTAAGAGTGATGGCTCTCGTATACAAACAGCCACAAAACCACCGGT
CATAACTCAACACAATTATAATAACATTACTTTAAATACTTGTGTTGAGTACAATATATATGGCAGAGTTGGCCA
AGGTTTTATTACTAATGTAACTGACTCCGCAGCTAGCTATAATTACTTAGCAGATGCTGGATTGGCAATTTTAG
ATACTTCAGGTGCCATAGACACTTTCGTTGTACAAGGTGAATATGGTCCCAATTATTATAAGGTTAACCCTTGT
GAAGATGTTAATCAGCAGTTTGTAGTGTCGGGCGGTAAGTTAGTAGGCATTCTGACTTCTCGTAATGAAACT
GGTTCTCAGCCTCTTGAAAATCAGTTTTATATTAAGTTAACTAATGGAAGTCGTCGTTTTAGACGT
>CK_CH_YNKM_SLDK-ZK202003.seq
ATGTTGGTGAAGTCACTGTTTTTAGCGACTCTTTTGTTTGCACTATCTAGTGCTACTTTGTATGATAATGATACG
TACGTTTACTACTACCAGAGTGCCTTCAGACCGTCTAATGGTTGGCATTTACATGGTGGCGCTTATGCAGTAG
TAAATGTTTCTTCACAAACTAACAATGCAGGTACAGCTTCAGAATGCACTGTTGGTATTATTAGTGGTGATACA
GTTGTTAATGCCTCTTCTATAGCTATGACAGCACCTGTAGGTCAAGGTATGCAGTGGTCTAAGTTACAATTTTG
TACTGCACACTGCAATTTTTCTGATTTTACAGTGTTTGTTACACATTGCTATGCCTCGGGCAGCGGTAAATGTC
CTTTAACGGGCCTTATTCCACAAGGTCATATTCGTATTTCTGCTATGCGGAATCATACTTTATTCTATAATTTAAC
AGTTAGTGTATCTAAGTACCCTACTTTTAAATCTTTGCAATGCGTTGATAATTTCACATCTGTTTACTTAAATGG
TGACCTTGTCTTCACTTCTAATCAGACGACAGACGTTATAAGTGCAGGTGTGTACTTTAAATCAGGTGGGCCT
ATAACCTATAAAGTTATGAAGGAATTTAAGGTTTTGGCTTATTTTGTTAATGGTACTGCACAAGATGTTATTTT
GTGTGATGACACACCTAGAGGTTTGCTAGCATGTCAATATAATACTGGCAATTTCTCAGATGGTTTTTATCCTT
TTACTAATAGTAGCTTAGTTAAGCAAAGGTTTGTTGTTTATCGTGAGAATAGTGTTAATACTGCTCTTACTTTA
ACCAATTACACCTTTCATAATGAGACTAATGCCCAGCCTAATTCAGGTGGTGTCCATACTATCTCAACTTATCA
AACACAAACTGCTCAGAGTGGTTATTATAATTTTAATTTATCATTTCTGAGTAGTTTTGTGTATAAAGATTCTGA
TTATATGTATGGGTCCTACCACCCACGATGTAGTTTTAGACCAGAAACTATTAATAATGGCTTGTGGTTTAATTC
ACTGTCAGTCTCATTAGCTTATGGCCCCCTTCAAGGTGGGTGTAAGCAATCAGTTTTTCAAGGCAGAGCTAC
TTGTTGTTATGCGTATTCCTATAACGGACCACGTATGTGTAAAGGTGTTTATAGTGGTCAGTTATCACAAGATT
TTGAATGTGGACTGTTGGTTTATGTTACTAAGAGTGATGGCTCTCGTATACAAACAGCCACAAAACCACCGGT
CATAACTCAACACAATTATAATAATATTACTTTAAATACTTGTGTTGAGTACAATATATATGGCAGAGTTGGCCA
AGGTTTTATTACTAATGTAACTGACTCCGCAGCTAGCTATAATTACTTAGCAGATGCTGGATTGGCAATTTTAG
ATACTTCAGGTGCCATAGACACTTTCGTTGTACAAGGTGAATATGGTCCCAATTATTATAAGGTTAACCCTTGT
GAAGATGTTAATCAGCAGTTTGTAGTGTCAGGCGGTAAGTTAGTAGGCATTCTGACTTCTCGTAATGAAACT
GGTTCTCAGCCTCTTGAAAATCAGTTTTATATTAAGTTAACTAATGGAAGCCGTCGTTTTAGACGT
>CK_CH_ZJ_QD_20200316.seq
ATGTTGGTGAAGTCACTGTTTTTAGCGACTCTTTTGTTTGCACTATCTAGTGCTACTTTGTATGATAATGATACG
TACGTTTACTACTACCAGAGCGCCTTCAGACCGTCTAATGGTTGGCATTTACATGGTGGCGCTTATGCAGTAG
TAAATGTTTCTTCACAAACTAACAATGCAGGTACAGCTTCAGAATGCACTGTTGGTATTATTAGTGGTGATACA
GTTGTTAATGCCTCTTCTATAGCTATGACAGCACCTGTAGGTCAAGGTATGCAGTGGTCTAAGTTACAATTTTG

TACTGCACACTGCAATTTTTCTGATTTTACAGTGTTTGTTACACATTGCTATGCCTCGGGCAGCGGTAAATGTC
CTTTAACGGGCCTTATTCCACAAGGTCATATTCGTATTTCTGCTATGCGGAATCATACTTTATTCTATAATTTAAC
AGTTAGTGTATCTAAGTACCCTACTTTTAAATCTTTGCAATGCGTTGATAATTTCACATCTGTTTACTTAAATGG
TGACCTTGTCTTCACTTCTAATCAGACGACAGACGTTATAAGTGCAGGTGTGTACTTTAAATCAGGTGGGCCT
ATAACCTATAAAGTTATGAAGGAATTTAAGGTTTTGGCTTATTTTGTTAATGGTACTGCACAAGATGTTATTTT
GTGTGATGACACACCTAGAGGTTTGCTAGCATGTCAATATAATACTGGCAATTTCTCAGATGGTTTTTATCCTT
TTACTAATAGTAGCTTAGTTAAGCAAAGGTTTGTTGTTTATCGTGAGAATAGTGTTAGTACTACTCTTACTTTA
ACCAATTACACCTTTCATAATGAGACTAATGCCCAGCCTAATTCAGGTGGTGTCCATACTATCTCAACTTATCA
AACACAAACTGCTCAGAGTGGTTATTATAATTTTAATTTATCATTTCTGAGTAGTTTTGTGTATAAAGATTCTGA
TTATATGTATGGGTCCTACCACCCACGATGTAGTTTTAGACCAGAAACTATTAATAATGGCTTGTGGTTTAATTC
ACTGTCAGTCTCATTAGCTTATGGCCCCCTTCAAGGTGGGTGTAAGCAATCAGTTTTTCAAGGCAGAGCTAC
TTGTTGTTATGCGTATTCCTATAACGGACCACGTATGTGTAAAGGTGTTTATAGTGGTCAGTTATCACAAGATT
TTGAATGTGGACTGTTGGTTTATGTTACTAAGAGTGATGGCTCTCGTATACAAACAGCCACAAAACCACCGGT
CATAACTCAACACAATTATAATAATATTACTTTAAATACTTGTGTTGAGTACAATATATATGGCAGAGTTGGCCA
AGGCTTTATTACTAATGTAACTGACTCCGCAGCTAGCTATAATTACTTAGCAGATGCTGGATTGGCAATTTTAG
ATACTTCAGGTGCCATAGACACTTTCGTTGTACAAGGTGAATATGGTCCCAATTATTATAAGGTTAACCCTTGT
GAAGATGTTAATCAGCAGTTTGTAGTGTCAGGCGGTAAGTTAGTAGGCATTCTGACTTCTCGTAATGAAACT
GGTTCTCAGCCTCTTGAAAATCAGTTTTATATTAAGTTAACTAATGGAAGCCGTCGTTTTAGACGT
>CK_CH_SC_SL-DK_202003_.seq
ATGTTGGTGAAGTCACTGTTTTTAGCGACTCTTTTGTTTGCACTATCTAGTGCTACTTTGTATGATAATGATACG
TACGTTTACTACTACCAGAGCGCCTTCAGACCGTCTAATGGTTGGCATTTACATGGTGGCGCTTATGCAGTAG
TAAATGTTTCTTCACAAACTAACAATGCAGGTACAGCTTCAGAATGCACTGTTGGTATTATTAGTGGTGATACA
GTTGTTAATGCCTCTTCTATAGCTATGACAGCACCTGTAGGTCAAGGTATGCAGTGGTCTAAGTTACAATTTTG
TACTGCACACTGCAATTTTTCTGATTTTACAGTGTTTGTTACACATTGCTATGCCTCGGGCAGCGGTAAATGTC
CTTTAACGGGCCTTATTCCACAAGGTCATATTCGTATTTCTGCTATGCGGAATCATACTTTATTCTATAATTTAAC
AGTTAGTGTATCTAAGTACCCTACTTTTAAATCTTTGCAATGCGTTGATAATTTCACATCTGTTTACTTAAATGG
TGACCTTGTCTTCACTTCTAATCAGACGACAGACGTTATAAGTGCAGGTGTGTACTTTAAATCAGGTGGGCCT
ATAACCTATAAAGTTATGAAGGAATTTAAGGTTTTGGCTTATTTTGTTAATGGTACTGCACAAGATGTTATTTT
GTGTGATGACACACCTAGAGGTTTGCTAGCATGTCAATATAATACTGGCAATTTCTCAGATGGTTTTTATCCTT
TTACTAATAGTAGCTTAGTTAAGCAAAGGTTTGTTGTTTATCGTGAGAATAGTGTTAATACTACTCTTACTTTAA
CCAATTACACCTTTCATAATGAGACTAATGCCCAGCCTAATTCAGGTGGTGTCCATACTATCTCAACTTATCAA
ACACAAACTGCTCAGAGTGGTTATTATAATTTTAATTTATCATTTCTGAGTAGTTTTGTGTATAAAGATTCTGAT
TATATGTATGGGTCCTACCACCCACGATGTAGTTTTAGACCAGAAACTATTAATAATGGCTTGTGGTTTAATTC
ACTGTCAGTCTCATTAGCTTATGGCCCCCTTCAAGGTGGGTGTAAGCAATCAGTTTTTCAAGGCAGAGCTAC
TTGTTGTTATGCGTATTCCTATAACGGACCACGTATGTGTAAAGGTGTTTATAGTGGTCAGTTATCACAAGATT
TTGAATGTGGACTGTTGGTTTATGTTACTAAGAGTGATGGCTCTCGTATACAAACAGCCACAAAACCACCGGT
CATAACTCAACACAATTATAATAATATTACTTTAAATACTTGTGTTGAGTACAATATATATGGCAGAGTTGGCCA
AGGCTTTATTACTAATGTAACTGACTCCGCAGCTAGCTATAATTACTTAGCAGATGCTGGATTGGCAATTTTAG
ATACTTCAGGTGCCATAGACACTTTCGTTGTACAAGGTGAATATGGTCCCAATTATTATAAGGTTAACCCTTGT
GAAGATGTTAATCAGCAGTTTGTAGTGTCAGGCGGTAAGTTAGTAGGCATTCTGACTTCTCGTAATGAAACT
GGTTCTCAGCCTCTTGAAAATCAGTTTTATATTAAGTTAACTAATGGAAGCCGTCGTTTTAGACGT
>CK_CH_SD_TA-WS-176BL-14d_20200827.seq
ATGTTGGGGAAGTCACTGTTGTTAGTGACCATTTTGTGTGTACTATGTAGTGCAAATTTGTTTGATTCTAAGTA
TGTTTACTACTATCAAAGTGCCTTTAGACCATCAGGTGGATGGCATTTGCACGGAGGTGCTTATGCAGTAGTG

AATGCTACTAATAAAACTAATAATGCAGGCGCCGCTACAGAGTGTTCTGTAGGTGTTCTTTTTAATTATACTAA
CGGAAATGACGTTGGTTATAAATATAATGCTTCTTCTATAGCCATGACAGCACCGTTGCCGGGTATGTCTTGGT
CTAAAACACAATTTTGTACCGCCCACTGTAACTTTTCGGATTTTACAGTGTTTGTTACACATTGTTTTGCAAAC
TCTTGTCCTTTAACAGGTAGGATACAAGAGAACCATATTCGTATTTCTGCTATGAGAAATGGTTCTCTCTTTTAT
AATTTAACAGTTAGTGTATCTAACTACCCTAAATTTAAATCGCTTCAATGTGTTAACAATTTCACTTCTGTTTATT
TAAATGGTGATCTTGTTTTTACTTCTAACGAAACCACTGATGTTATAGGTGCTGGTGTGTATTTTAAATCAGGT
GGGCCTATAACCTATAAAATTATGAAAGAATTTAAGGTTTTGGCTTATTTTGTAAATGGCACTGCGCAAGATG
TAATTCTGTGTGATGACACACCTAGAGGCTTGCTTGCGTGTCAATATAACACCGGTAATTTTACGGATGGTTT
TTATCCATTTACTAATAGCAGTTTAGTTAAGGAAAGATTTATTGTTTACCGTGAAAGTAGTGTTAATACTACTTT
GGCGTTAACTAATTTTACTTTTTATAATGAGACTAATGCACAACCTAATTCTGTAGGTGGTGTTAATAGTATTCA
AACTTATCAAACACAAACAGCTCAGAGTGGTTATTATAATTTTAATTTATCATTTCTGAGTAGTTTTTTGTATGT
AGATTCCAATTATATGTATGGTTCTTATCACCGTAGCTGTAATTTTAGACCAGAAAACATTAATAATGGCTTATG
GTTTAATTCACTCTCAGTTTCAGTTGCATATGGACCTCTTCAAGGTGGGTGTAAGCAGTCTGTTTTTAGTGGT
AGAGCGACTTGTTGTTATGCTTATTCTTATAACGGTCCTTACGCTTGTAAAGGTGTTTACCCAGGTGAGTTAA
CAAAGAGTTTTGAATGTGGTTTATTGGTTTATGTTACTAAGAGCGATGGCTCTCGTATACAAACAGCCAATGA
AGCACCAGTTATAACTCAACACAATTATAATAATATTACTTTAAATACTTGTGTTGATTATAATATATATGGCAGA
GTAGGCCAAGGGTTTATTACTAATGTAACTGAGTCAGCTTCTAGTTATAATTATCTAGCAGATGCAGGTTTAGC
TATTTTAGATACTTCAGGAGCCATAGACATATTTGTTGTTCGAGGTGCATATGGTCCTAATTATTATAAGGTTAA
TCCGTGTGAAGATGTTAATCAACAGTTTGTAGTGTCTGGTGGCAATATAGTTGGCATTCTTACTTCTAGAAATG
AAACAGGTTCTGAACAGGTTGAGAACCAGTTTTATGTTAAGTTAACCAATAGCCCACATCGTCGCAGGCGT
>CK_CH_SD_TA-WS-165BL-14d_20200827.seq
ATGTTGGGGAAGTCACTGTTGTTAGTGACCATTTTGTGTGTACTATGTAGTGCAAATTTGTTTGATTCTAAGTA
TGTTTACTACTATCAAAGTGCCTTTAGACCATCAGGTGGATGGCATTTGCACGGAGGTGCTTATGCAGTAGTG
AATGCTACTAATAAAACTAATAATGCAGGCGCCGCTACAGAGTGTTCTGTAGGTGTTCTTTTTAATTATACTAA
CGGAAATGACGTTGGTTATAAATATAATGCTTCTTCTATAGCCATGACAGCACCGTTGCCGGGTATGTCTTGGT
CTAAAACACAATTTTGTACCGCCCACTGTAACTTTTCGGATTTTACAGTGTTTGTTACACATTGTTTTGCAAAC
TCTTGTCCTTTAACAGGTAGGATACAAGAGAACCATATTCGTATTTCTGCTATGAGAAATGGTTCTCTCTTTTAT
AATTTAACAGTTAGTGTATCTAACTACCCTAAATTTAAATCGCTTCAATGTGTTAACAATTTCACTTCTGTTTATT
TAAATGGTGATCTTGTTTTTACTTCTAACGAAACCACTGATGTTATAGGTGCTGGTGTGTATTTTAAATCAGGT
GGGCCTATAACCTATAAAATTATGAAAGAATTTAAGGTTTTGGCTTATTTTGTAAATGGCACTGCGCAAGATG
TAATTCTGTGTGATGACACACCTAGAGGCTTGCTTGCGTGTCAATATAACACCGGTAATTTTACGGATGGTTT
TTATCCATTTACTAATAGCAGTTTAGTTAAGGAAAGATTTATTGTTTACCGTGAAAGTAGTGTTAATACTACTTT
GGCGTTAACTAATTTTACTTTTTATAATGAGACTAATGCACAACCTAATTCTGTAGGTGGTGTTAATAGTATTCA
AACTTATCAAACACAAACAGCTCAGAGTGGTTATTATAATTTTAATTTATCATTTCTGAGTAGTTTTTTGTATGT
AGATTCCAATTATATGTATGGTTCTTATCACCGTAGCTGTAATTTTAGACCAGAAAACATTAATAATGGCTTATG
GTTTAATTCACTCTCAGTTTCAGTTGCATATGGACCTCTTCAAGGTGGGTGTAAGCAGTCTGTTTTTAGTGGT
AGAGCGACTTGTTGTTATGCTTATTCTTATAACGGTCCTTACGCTTGTAAAGGTGTTTACCCAGGTGAGTTAA
CAAAGAGTTTTGAATGTGGTTTATTGGTTTATGTTACTAAGAGCGATGGCTCTCGTATACAAACAGCCAATGA
AGCACCAGTTATAACTCAACACAATTATAATAATATTACTTTAAATACTTGTGTTGATTATAATATATATGGCAGA
GTAGGCCAAGGGTTTATTACTAATGTAACTGAGTCAGCTTCTAGTTATAATTATCTAGCAGATGCAGGTTTAGC
TATTTTAGATACTTCAGGAGCCATAGACATATTTGTTGTTCGAGGTGCATATGGTCCTAATTATTATAAGGTTAA
TCCGTGTGAAGATGTTAATCAACAGTTTGTAGTGTCTGGTGGCAATATAGTTGGCATTCTTACTTCTAGAAATG
AAACAGGTTCTGAACAGGTTGAGAACCAGTTTTATGTTAAGTTAACCAATAGCCCACATCGTCGCAGGCGT
>CK_CH_GX_YL_WY-ZJF_20200715.seq

ATGTTGGAGAAGTTACTGTTTTTAGTGACCATTTTGTGTGCACTATGTAGTGCAAATTTGTTTGATGCTGATAA
TAATTATGTGTACTACTACCAAAGTGCCTTTAGGCCTCCAACGGGATGGCATTTGCAAGGGGGTGCTTATGCT
GTAGTGAATTCTACTAATTATACTAATAATGCCGGTTCTGCAAGTGAGTGCACTGTTGGTATTATTAAGGACGT
CTATAATCAAAGTGCGGCTTCCATAGCTATGACAGCACCTCTTCAGGGTATGGCTTGGTCTAAGTCACAATTT
TGTAGTGCACACTGTAACTTTTCTGAAATTACAGTTTTTGTCACACATTGTTATAGTAGTGGTGCAGGGTCTT
GTCCTATAACAGGCATGATTCCACGGGGTCATATTCGCGTTTCTGCAATGAAAAATGGCTCTTTATTTTATAAT
TTAACAGTTAGCGTGTCTAAATACCCTAATTTTAAATCGTTTCAATGTGTTAACAACTTCACATCTGTTTATTTA
AATGGTGATCTTGTTTTTACTTCTAACACAACTACTGATGTTAAGTCAGCAGGTGTGTATTTTAAAGCAGGTG
GACCTGTAAATTATAATATTATGAAAGAATTTAAGGTTCTGGCTTATTTTGTCAATGGTACTGTGCAAGATGTA
ATTCTGTGTGATGACACACCGAGAGGCTTGCTTGCATGTCAATATAATAATGGTAATTTTTCAGATGGGTTTTA
CCCTTTTACTAATTCTAGTTTAGTTAAGGAAAAGTTTATTGTTTATCGTGAGAATAGTGTTAATACTATTCTTAC
TTTAACTAACTATACTTTTTATAATGTGACTAATGCCTCGCCTAATCGAGGTGGTGTTCAGTCTATTCCAACTTA
TCAAACACAAACAGCTCAGAGTGGTTATTATAATTTTAATTTATCATTTCTGAGTAGTTTTGTGTATAAAGAGT
CTAATTACATGTATGGGTCTTACCCACCCTGCATGTAATTTTAGATTAGAAACTATTAATAATGGCTTGTGGTTTA
ATTCATTGTCAGTTTCGCTTGCTTATGGACCACTTCAAGGTGGGTGTAAGCAGTCGGTTTTTAGTAGTAGAGC
CACTTGTTGTTATGCTTATTCATATAATGGTCCTCGCGCATGTAAGGGTGTTTATGCAGGCGAGTTACTACAAA
ATTTTGAATGTGGACTGTTGGTTTATGTTACTAAGAGCGATGGCTCTCGTATACAAACAGCCACCGTTCCACC
AGTTGTAACTCAACACAATTATAATAATATTACTTTAAATACTTGTGTTGATTATAATATATATGGCAGAGTTGGT
CGAGGTTTTATTACTAATGTAACTGACTCATCATCTAGTTATAATTATTTAGCAGATGCAGGGTTGGCTATTTTA
GATACATCAGGTGCCATAGACATCTTTGTTGTACAAGGTGAACATGGTCTTAATTATTACAAGGTTAATCCCTG
TGAAGATGTAAACCAGCAGTTTGTAGTTTCTGGTGGTAAATTAGTAGGTATTCTTACCTCACGTAATGCAACA
GGTTCTCAGCCTCTTGAGAATCAATTCTACATTAAACTCACTAAAGAGACACGTCGTTTTAGACGT
>CK_CH_JS_CZ-SJQY_12482_20201129.seq
ATGTTGGGGAAGTCACTGTTTTTAGTGACCATTTTGTGTGCACTATGTAGTGCAAATTTGTTTGATTCTTCTAA
TAATTATGTGTACTACTACCAAAGTGCCTTTAGGCCTCCAAATGGATGGCATCTGCAAGGGGGTGCTTATGCA
GTAGTGAATTCTACTAATTATACTAATAATGCCGGTTCTGCAAGTGAGTGCACTGTTGGTGTTATTAAGGACGT
CTATAATCAAAGTGCGGCTTCCATAGCTATGACAGCACCTCCTCAGGGTATGGCTTGGTCTAAGTCACAATTT
TGTAGTGCACACTGTAACTTTTCTGAAATTACAGTTTTTGTCACACATTGTTATAGTAGTGGTGCAGGTTCTTG
TCCTATAACAGGCATGATTGCACGTGATCATATTCGTATTTCTGCAATGAAAAATGGTTCTTTATTTTATAATTTA
ACAGTTAGCGTATCTAAATACCCTAAGTTTAAATCTTTTCAATGTGTTAACAATCTCACATCTGTTTATCTAAAT
GGTGATCTTGTTTTTACTTCTAACAAAACTGCTGATGTTATATCTGCAGGTGTGTATTTTAAAGCAGGTGGAC
CTGTAAATTATAATATTATGAAAGAATTTAAGGTTCTGGCTTATTTTGTCAATGGTACTGTGCAAGATGTAATTC
TGTGTGATGACACACCGAGAGGCTTGCTTGCATGTCAATATAATAATGGTAATTTTTCAGATGGATTCTACCCT
TTTACTAATTCTTCTTTAGTTAAGGATAGGTTTATTGTATATCGAGAAAGTAGCACTAACACTATTTTAGAGTTA
ACTAATTTCACTTTTACTAATGTAAGTAATGCTTCTCCTAATTCAGGTGGCGTTGATACTTTCCAATTATATCAA
ACACATACTGCTCAGGATGGTTATTATAATTTTAATTTATCATTTCTGAGTAGTTTTGTGTATAAACCATCTGATT
TTATGTATGGGTCATACCACCCAAATTGTAATTTTAGACCAGAGAATATTAATAATGGCTTATGGTTTAATTCAT
TATCTGTGTCACTTACTTACGGACCCATTCAAGGTGGTTGTAAGCAATCTGTTTTTAGTAATAAAGCAACTTGT
TGCTATGCTTATTCTTACCGAGGTCCTACTAGATGTAAGGGTGTTTATAGAGGGGAGCTAACGCAATACTTTG
AATGTGGACTTCTAGTTTATGTAACTAAGAGTGATGGCTCTCGTATACAAACTAGAAGTGAACCACTGGTGTT
AACTCAATATAATTATAACAACATTACTTTAAATAAGTGTGTTGATTATAATATATATGGCAGAGTTGGTCGAGG
TTTTATTACTAATGTAACTGACTCATCATCTAGTTATAATTATTTAGCAGATGCAGGGTTGGCTATTTTAGATACA
TCAGGTGCCATAGACATCTTTGTTGTACAAGGTGAACATGGTCTTAATTATTACAAGGTTAATCCCTGTGAAG
ATGTAAACCAGCAGTTTGTAGTTTCTGGTGGTAAATTAGTAGGTATTCTTACCTCACGTAATGCAACAGGTTCT

CAGCCTCTTGAGAATCAATTCTACATTAAACTCACTAAAGAGACACGTCGTTTTAGACGT
>CK_CH_CQ_DK-YSN_20200908.seq
ATGTTGGGGAAGTCACTGTTTTTAGTGACCATTTTGTGTGCACTATGTAGTGCAAATTTATTCGATCCTCATAA
TAATTATGTGTACTACTACCAAAGTGCCTATAGACCACCAAATGGGTGGCATTTGCAAGGGGGTGCTTATGCA
GTAGTGAATTCTACTATTAAATATAACAATGCAGGCACCGCTACTGAGTGTTCTGTAGGTGTTCTTTTTAATTAT
ACTAACGGAAATGACGTTGGTTATAATAATAGTGCATCTTCCGTAGCCATGACAGCACCGTCTTCTGGTATGTC
TTGGTCTAAAAGTCAATTTTGCACTGCTCACTGTAATTTTTCAGATTTTACAGTGTTTGTTACTCATTGTTTTGC
AAATTCTTGTCCTTTAACAGGTAGGATAGAGGAGGGCCATATTCGTATTTCTGCTATGAGAAATGGTTCTCTAT
TTTATAATTCAACAGTTAGTGTATCTACATACCCTAAATTTAAATCCTTTCAATGTGTTAACAATTACACTTCTGT
TTATTTAAATGGTGACCTTGTTTTTACTTCTAATGAAACTACTGATGTTAAAGGTGCTGGCGTGTATTTTAAAG
CAGGTGGGCCTATAACCTATAAAATTATGAAAGAATTTAAGGTTTTGGCTTATTTTGTAAATGGCACAGCACA
AGATGTAATTTTGTGCGACAATTCCCCCAAGGGTTTGCTAGCTTGTCAGTATAACACTGGTAATTTTTCAGAT
GGATTCTACCCTTTTACTAATTCTTCTTTAGTTAAGGATAGGTTTATTGTATATCGAGAAAGTAGCACTAACACT
ACTTTAGAGTTAACTAATTTTACTTTTACTAATGTAAGTAATGCTTCTCCTAATTCAGGTGGCGTTGATACTTTC
CAATTATATCAAACACATACTGCTCAGGATGGTTATTATAATTTTAATTTATCATTTCTGAGTAGTTTTGTGTATA
AACCATCTGATTTTATGTATGGGTCATACCACCCAAAGTGTAATTTTAGACCAGAGAATATTAATAATGGCTTAT
GGTTTAATTCATTATCTGTGTCACTTACTTACGGACCCATTCAAGGTGGTTGTAAGCAATCTGTTTTTAGTAAT
AAAGCAACTTGTTGCTATGCTTATTCTTACCGAGGTCCTACTAGATGTAAGGGTGTTTATAGAGGGGAGCTAA
TGCAATACTTTGAATGTGGACTTCTAGTTTATGTAACTAAGAGTGATGGCTCTCGTATACAAACTAGAAGTGA
ACCACTGGTGTTAACTCAATATAATTATAACAACATTACTTTAAATAAGTGTGTTGAGTATAATATATATGGCAG
AGTAGGCCAAGGTTTTATTACTAATGTGACTGATTCTGCTGCTAATTTTAGTTATTTAGCAGATGGTGGGTTAG
CTATTTTAGATACGTCGGGTGCCATAGATGTTTTTGTTGTACAGGGCATCTATGGTCTTAATTATTACAAGGTT
AATCCTTGTGAAGATGTTAATCAACAATTTGTAGTGTCTGGTGGCAATATAGTTGGCATTCTTACTTCTAGAAA
TGAAACAGGTTCTGAACAGGTTGAGAACCAGTTTTATGTTAAGTTAACCAATAGCTCACATCGTCTTAGGCG
T
>CK_CH_GZ_DK20200410.seq
ATGTTGGGGAAGTCACTGTTAATAGTGACTGTTTTGTTTGCACTATGTAGTGCCACTCTTTATACTCACGATTA
TGTTTACTACTACCAAAGTGCCTATAGACCACCAAATGGATGGCATTTGCAAGGGGGTGCTTATGCAGTAGT
GAATTCTACTACTAAAACTAATAATGCAGGCACCGCTAGCGAGTGTTCTGTAGGTGTTCTTTTTAATTATACTA
ACGGAAATGATGTTGGTTATAATAGTGTTGTTTCTTCTGTAGCCATGACAGCACCGCCTTCTGGTATGTCTTG
GTCTAAAGCAGAATTTTGTACTGCCCATTGTAACTATTCCGATTTTACAGTGTTTGTTACACATTGTTTTGCAA
ACTCTTGTCCTTTAACAGGTAAGATAGAGAAAAACCATATCCGTATTTCTGCTATGAGAAATGGTTCTCTATTT
TACAATTTAACAGTTAGTGTATCCAAATACCCTAAATTTAAATCGCTTCAATGTGTTAACAATTTCACTTCTGTT
TATTTAAATGGTGACCTTGTTTTTACTTCTAACAAAACCACTGCTGTTATAGGTGCTGGTGTGTATTTTAAAGC
AGGTGGGCCTATAACCTATAAAATTATGAAACAGTTTAAGGTTTTGGCTTATTTTGTCAATGGTACTGTGCAA
GATGTAATTCTGTGTGATGACACACCGAGAGGCTTGCTTGCATGTCAGTATAACACTGGTAATTTTTCAGATG
GATTCTACCCTTTTACTAATTCTTCTTTAGTTAAGGATAGGTTTATTGTATATCGACAAAGTAGCACTAACACTA
CTTTAGACTTAACTAATTTCACTTTTACTAATGTAAGTAATGCTTCTCCTAATTCGGGTGGCGTTGATACTTTCC
AATTATATCAAACACATACTGCTCAGGATGGTTATTATAATTTTAATTTATCATTTCTGAGTAGTTTTGTGTATATA
CCATCTGATTTTATGTATGGGTCATACCACCCAAATTGTAATTTTAGACCAGAGAATATTAATAATGGCTTATGG
TTTAATTCATTATCTGTGTCACTTACTTACGGACCCATTCAAGGTGGTTGTAAGCAATCTGTTTTTAGTAATAAA
GCAACTTGTTGCTATGCTTATTCTTACCAAGGTCCTACTAGATGTAAGGGTGTTTATAGAGGGGAGCTAACGC
AATACTTTGAATGTGGACTTCTAGTTTATGTAACTAAGAGTGATGGCTCTCGTATACAAACTAGAAGTGAACC
ACTGGTGTTAACACAATATAATTATAACAACATTACTTTAAATAAGTGTGTTGAGTATAATATATATGGTAGAGT

TGGTCAAGGTTCTATTACTAATGTAACTGAAGCAACTGCCAATTATAGTTATCTAGCAGATGGTGGTTTAGCTA
TTTTAGATACTTCAGGAGCCATAGACATATTTGTTGTACAAGGTGAATATGGTCTTAATTATTACAAGGTTAAC
CCCTGTGAAGATGTAAACCAGCAGTTTGTAGTTTCTGGTGGTAAATTAGTAGGTATTCTTACCTCACGTAATG
CAACTGGTTCTCAGTCTCTTGAAAATCAGTTTTATATTAAGTTAACTAATGGAAGCCGTCGTTTTAGACGT
>CK_CH_YN_KM-DK_YJZ20200804.seq
ATGTTGGAGAAGTCACTGTTGCTAGTGACCATTTTGTTTGCACTATGTAGTGCTAGTTTACATAGTGCTAATTA
TGTGTACTATTATCAAAGTGCTTTTAGGCCTCCAAATGGGTGGCATTTACAAGGGGGTGCGTATGCAGTAGTT
AATTCTACTAGTAAGACTAATAACGCAGGCACAGCCCCCGAGTGTTCTGTGGGTGTTCTTTTTAATTATACTAA
CGGAAATAGTGACGTTGGTTATAATCAAAGTGCTTCTTCCATAGCCATGACAGCACCGTCTGAAGGTATGGCT
TGGTCTAAATCACAATTTTGTACTGCTCACTGTAATTACTCGGATTTTACAGTGTTTATTACACATTGTTTTGCA
AACTCTTGTCCTTTAACAGGTAGGATAGAGCAGGGCCATATTCGTATTTCTGCTATGAGAAATGGTTCTTTATT
TTATAATCTAACAGTTAGTGTAACTAAATACCCTAATTTTAAATCTCTTCAATGTGTTAACAATTTCACTTCTGTT
TATCTAAATGGTGATCTTGTTTTTACATCCAATGAAACCACTGATGTTAAATCTGCCGGTGTGTATTTTAAAGC
AGGTGGGCCTATAACCTATAATGTTATGAAAGAATTTAAGGTTTTGGCTTACTTTGTTAATGGTACAGCACAA
GATGTAATTTTGTGCGACAATTCACCCAAGGGTTTGTTAGCTTGTCAATATAACACTGGCAATTTTTCAGATG
GATTTTATCCTTTTACTAATACCACTTTAGTTAGGGAAAAATTCATCGTACATCGCGAAAGTAGTGTTAATACTA
CTCTGGAGTTAACTAATTTCACTTTTACTAATGTAAGTAATGCACAGCCTAATGCTGGTGGTGTTGGTACTTTT
CATTTATATCAAACACAAACAGCTCAGAGTGGCTATTATAATTTTAATTTGTCATTTCTGAGTAGTTTTGTGTAT
AAACCAACTGATTTTATGTATGGTTCCTACCATAAAGCATGTTCTTTTAGACCAGAAAACATTAATAATGGTTT
GTGGTTTAATTCGTTGTCAGTATCTCTTGCCTATGGACCCCTACAAGGAGGGTGTAAGCAATCTGTTTTTAGT
GGTAGGGCAACGTGTTGCTATGCTTACTCTTATAATGGCCCAAGAGCCTGTAAAGGTGTCTATAGCGGCGAG
TTACAGACTAATTTTGAATGTGGATTGTTGGTTTATGTTACTAAGAGTGATGGCTCTCGTATACAAACTAGATC
AGAACCCTTAGTATTAACTCAACACAATTATAATAATATTACTTTAAATAAGTGTGTTGACTATAATATATATGGC
AGAGTAGGCCAAGGTTTTATTACTAATGTGACTGATTCTGCTGCTAATTTTAGTTACTTAGCAGATGGTGGGT
TAGCTATTTTAGATACTTCGGGTGCCATAGATGTCTTTGTTGTACAAGGCAACTATGGTCCTAATTATTACAAG
GTTAATCCTTGTGAAGATGTTAATCAACAGTTTGTAGTGTCTGGTGGCAATATAGTTGGCATTCTTACTTCTAG
AAATGAAACAGGTTCTGAACAGGTTGAGAACCAGTTTTATTTCAAGTTAACCAATAGCTCACGTCGTCGCAG
GCGT
>CK_CH_GD_GZ-YX_20200427.seq
ATGTTGGGGAAGTCACTGTTTTTAGTGACCATTTTGTGTGCACTATGTAGTGCAAATTTGTTTGATTCTGCCA
ATAATTATGTGTACTACTACCAAAGTGCCTTTAGGCCTCCAAATGGATGGCATCTGCAAGGGGGTGCTTATGC
AGTAGTGAATTCTACTAATTATACTAATAATGCCGGTTCTGCAAGTGGGTGCACTGTTGGTGTTATTAAGGAC
GTCTATAATCAAAGTGCGGCTTCCATAGCTATGACAGCACCTCCTCAGGGTATGGCTTGGTCTAAGTCACAAT
TTTGTAGTGCACACTGTAACTTTTCTGAAATTACAGTTTTTGTCACACATTGTTATAGTAGTGGTGCAGGTTCT
TGTCCTATAACAGGCATGATTGCACGTGATTATATTCGTATTTCTGCAATGAAAAATGGTTCTTTATTTTATAATT
TAACAGTTAGCGTATCTAAATACCCTACGTTTAAATCTTTTCAATGTGTTAACAATTCCACATCTGTTTATCTAAA
TGGTGATCTTGTTTTTACTTCTAACAAAACTGCTGATGTTACGTCAGCAGGTGTGTATTTTAAAGCAGGTGGA
CCCGTAAATTATAGTGTTATGAAAGAATTTAAGGTTCTTGCTTACTTTGTTAATGGTACAGCACAAGATGTAAT
TTTGTGCGACAATTCCCCCAAGGGTTTGCTGGCTTGTCAATATAATACTGGCAATTTTTCAGATGGCTTTTATC
CTTTTACTAATAGTATTTTAGTTAGGGACAAGTTCATTGTCTATCGTGAAAGTAGTGTCAATACTACTTTGACG
TTAACTAATTTCACTTTTACTAATGTAAGTACTGCACAGCCTAATAGTGGTGGTGTTAGTACTTTTCATTTATAT
CAAACACAAACAGCTCAGAGTGGTTATTATAATTTTAATTTGTCATTTCTGAGTCAGTTTGTGTATAAGGCAA
GTGATTTTATGTATGGGTCTTACCACCCTAGGTGTTCTTTTAGACCAGAAACCATTAATAGTGGTTTATGGTTT
AATTCCTTGTCAGTTTCTCTTACTTATGGACCCCTACAGGGAGGGTGTAAGCAATCTGTTTTTGATGGTAAGG

CAACGTGTTGTTATGCCTACTCTTATAATGGCCCAAGGGCATGTAAAGGTGTTTATTCAGGTGAATTAAGCAA
GACTTTTGAATGTGGATTGCTGGTTTATGTTACTAAGAGTGATGGCTCTCGTATACAGACTAGAACGGAGCCC
TTAGTATTAATGCAACACAATTATAATAATATTACTTTAGATAAGTGTGTTGACTATAATATATATGGCAGAGTAG
GCCAAGGTTTTATTACTAATGTGACTGATTCTGCTGCTAATTTTAGTTATTTAGCAGATGGTGGGTTAGCTATT
TTAGATACTTCGGGTGCCATAGATGTCTTTGTTGTACAGGGCAGCTATGGTCTTAATTATTACAAGGTCAATCC
TTGTGAAGATGTTAACCAACAGTTTGTAGTGTCTGGTGGCAATATAGTTGGCATTCTTACTTCTAGAAATGAA
ACAGGTTCTGAACAGGTTGAGAACCAGTTTTATGTTAAGTTAACCAATAGCTCACATCGTCGTAGGCGT
>CK_CH_GX_YL_BSWY-WZM_20200422.seq
ATGTTGGGGAAGTCACTGTTTTTAGTGACCATTTTGTGTGCACTATGTAGTGCAAATTTGTTTGATTCTGCCA
ATAATTATGTGTACTACTACCAAAGTGCCTTTAGGCCTCCAAATGGATGGCATCTGCAAGGGGGTGCTTATGC
AGTAGTGAATTCTACTAATTATACTAATAATGCCGGTTCTGCAAGTGGGTGCACTGTTGGTGTTATTAAGGAC
GTCTATAATCAAAGTGCGGCTTCCATAGCTATGACAGCACCTCCTCAGGGTATGGCTTGGTCTAAGTCACAAT
TTTGTAGTGCACACTGTAACTTTTCTGAAATTACAGTTTTTGTCACACATTGTTATAGTAGTGGTGCAGGTTCT
TGTCCTATAACAGGCATGATTGCACGTGATTATATTCGTATTTCTGCAATGAAAAATGGTTCTTTATTTTATAATT
TAACAGTTAGCGTATCTAAATACCCTACGTTTAAATCTTTTCAATGTGTTAACAATTCCACATCTGTTTATCTAAA
TGGTGATCTTGTTTTTACTTCTAACAAAACTGCTGATGTTACGTCAGCAGGTGTGTATTTTAAAGCAGGTGGA
CCCGTAAATTATAGTGTTATGAAAGAATTTAAGGTTCTTGCTTACTTTGTTAATGGTACAGTACAAGATGTAAT
TTTGTGCGACAATTCCCCCAAGGGTTTGCTGGCTTGTCAATATAATACTGGCAATTTTTCAGATGGCTTTTATC
CTTTTACTAATAGTATTTTAGTTAGGGACAAGTTCATTGTCTATCGTGAAAGTAGTGTCAATACTACTTTGACG
TTAACTAATTTCACTTTTACTAATGTAAGTACTGCACAGCCTAATAGTGGTGGTGTTAGTACTTTTCATTTATAT
CAAACACAAACAGCTCAGAGTGGTTATTATAATTTTAATTTGTCATTTCTGAGTCAGTTTGTGTATAAGGCAA
GTGATTTTATGTATGGGTCTTACCACCCTAGGTGTTCTTTTAGACCAGAAACCATTAATAGTGGTTTATGGTTT
AATTCCTTGTCAGTTTCTCTTACTTATGGACCCCTACAGGGAGGGTGTAAGCAATCTGTTTTTAATGGTAAGG
CAACGTGTTGTTATGCCTACTCTTATAATGGCCCAAGGGCATGTAAAGGTGTTTATTCAGGTGAATTAAGCAA
GACTTTTGAATGTGGATTGCTGGTTTATGTTACTAAGAGTGATGGCTCTCGTATACAGACTAGAACGGAGCCC
TTAGTATTAATGCAACACAATTATAATAATATTACTTTAGATAAGTGTGTTGACTATAATATATATGGCAGAGTAG
GCCAAGGTTTTATTACTAATGTGACTGATTCTGCTGCTAATTTTAGTTATTTAGCAGATGGTGGGTTAGCTATT
TTAGATACTTCGGGTGCCATAGATGTCTTTGTTGTACAGGGCAGCTATGGTCTTAATTATTACAAGGTCAATCC
TTGTGAAGATGTTAACCAACAGTTTGTAGTGTCTGGTGGCAATATAGTTGGCATTCTTACTTCTAGAAATGAA
ACAGGTTCTGAACAGGTTGAGAACCAGTTTTATGTTAAGTTAACCAATAGCTCACATCGTCGCAGGCGT
>CK_CH_GX_WM-LY_ZHX20200810.seq
ATGTTGGGGAAGTCACTGTTTTTAGTGACCATTTTGTGTGCACTATGTAGTGCAAATTTATTCGATCCTGCTAA
TACTTATGTGTACTACTACCAAAGTGCCTTTAGGCCTCCAAATGGATGGCACCTACAAGGGGGTGCTTATGCA
GTAGTCAATTCCACTAATTATACTAATAATGCCGGTTCTGCACAACATTGCACTGTTGGTGTTATTAAGGACGT
CTATAATCAAAGTGCGGCTTCCATAGCTATGACAGCACCTCTTCAGGGTATGGCTTGGTCTAAGTCACAATTT
TGTAGTGCACACTGTAACTTTTCTGAAATTACAGTTTTTGTCACACATTGTTATAGTAGTGGTAGCGGGTCTTG
TCCTATAACAGGCATGATTGCACGTGATCATATTCGTATTTCTGCAATGAAAAATGGTACTTTATTTTATAATTTA
ACAGTTAGCGTATCTAAATACCCTAATTTTAAATCTTTTCAATGCGTTAATAATCTCACATCTGTTTATCTAAATG
GTGATCTTGTTTTTACTTCCAACAAAACTACTGATGTTACGTCAGCAGGTGTGTATTTTAAAGCAGGTGGACC
TGTAAATTATAGTATTATGAAAGAATTTAAGGTTCTTGCTTACTTTGTTAATGGTACAGCACAAGATGTAATTTT
GTGCGACAATTCCCCCAAGGGTTTGCTAGCTTGTCAATATAACACTGGCAATTTTTCAGATGGCTTTTATCCTT
TTACTAATAGTACTTTAGTTAGGGAAAAGTTCATCGTATATCGCGAAAGTAGTGTTAATACTACTCTGGCGTTA
ACTAATTTCACTTTTACTAATGTAAGTAATGCACAGCCTAATAGTGGTGGTGTTAATACTTTTCATTTATATCAA
ACACAAACAGCTCAGAGTGGTTATTATAATTTTAATTTGTCATTTCTGAGTCAGTTTGTGTATAAGGCAAGTGA

TTTTATGTATGGGTCTTACCACCCTAGGTGTTCTTTTAGACCAGAAACCATTAATAGTGGTTTATGGTTTAATTC
CTTGTCAGTTTCTCTTACTTATGGACCCCTACAGGGAGGGTGTAAGCAATCTGTTTTTAATGGTAAGGCAACG
TGTTGTTATGCCTACTCTTATAATGGCCCAAGGGCATGTAAAGGTGTTTATTCAGGTGAATTAAGCAAGACTT
TTGAATGTGGATTGCTGATTTATGTTACTAAGAGTGATGGTTCTCGTATACAGACTAGAACAGAGCCCTTAGT
ATTAACGCAACACAATTATAATAATATTACTTTAGATAAGTGTGTTGACTATAATATATATGGCAGAGTAGGCCA
AGGTTTTATTACTAATGTGACTGATTCTGCTGCTAATTTTAGTTATTTAGCAGATGGTGGGTTAGCTATTTTAGA
TACTTCGGGTGCCATAGATGTCTTTGTTGTACAGGGCAGCTATGGTCTTAATTATTACAAGGTCAATCCTTGTG
AAGATGTTAACAAACAGTTTGTAGTGTCTGGTGGCAATATAGTTGGCATTCTTACTTCTAGAAATGAAACAGG
TTCTGAACAGGTTGAGAACCAGTTTTATGTTAAGTTAACCAATAGCTCACATCGTCGCAGGCGT
>CK_CH_YN_MC-2_20200928.seq
ATGTTGGGGAAGTCACTGTTTTTAGTGACCATTTTGTGTGCACTATGTAGTGCAAATTTATTCGATCCTGCTAA
TACTTATGTGTACTACTACCAAAGTGCCTTTAGGCCTCCAAATGGATGGCACCTACAAGGGGGTGCTTATGCA
GTAGTCAATTCCACTAATTATACTAATAATGCCGGTTCTGCACAACATTGCACTGTTGGTGTTATTAAGGACGT
CTATAATCAAAGTGCGGCTTCCATAGCTATGACAGCACCTCTTCAGGGTATGGCTTGGTCTAAGTCACAATTT
TGTAGTGCACACTGTAACTTTTCTGAAATTACAGTTTTTGTCACACATTGTTATAGTAGTGGTAGCGGGTCTTG
TCCTATAACAGGCATGATTGCACGTGATCATATTCGTATTTCTGCAATGAAAAATGGTACTTTATTTTATAATTTA
ACAGTTAGCGTATCTAAATACCCTAATTTTAAATCTTTTCAATGCGTTAATAATCTCACATCTGTTTATCTAAATG
GTGATCTTGTTTTTACTTCCAACAAAACTACTGATGTTACGTCAGCAGGTGTGTATTTTAAAGCAGGTGGACC
TGTAAATTATAGTATTATGAAAGAATTTAAGGTTCTTGCTTACTTTGTTAATGTACAGCACAAGATGTAATTTTG
TGCGACAATTCCCCCAAGGGTTTGCTAGCTTGTCAATATAACACTGGCAATTTTTCAGATGGCTTTTATCCTTT
TACTAATAGTACTTTAGTTAGGGAAAAGTTCATCGTATATCGCGAAAGTAGTGTTAATACTACTCTGGCGTTAA
CTAATTTCACTTTTACTAATGTAAGTAATGCACAGCCTAATAGTGGTGGTGTTAATACTTTTCATCTATATCAAA
CACAAACAGCTCAGAGTGGTTATTATAATTTTAATTTGTCATTTCTGAGTCAGTTTGTGTATAAGGCAAGTGAT
TTTATGTATGGGTCCTACCACCCTAGTTGTTCTTTTAGACCAGAAACCATTAATAGTGGTTTGTGGTTTAATTC
TTTGTCAGTTTCTCTAGCTTACGGACCACTTCAAGGTGGGTGTAAGCAGTCAGTTTTTAGTGGTAGGGCAAC
GTGTTGCTATGCCTACTCTTACAATGGCCCGATAGCCTGTAAAGGTGTTTATTCAGGCGAATTACGGACTAAT
TTTGAATGTGGATTGCTGATTTATGTTACTAAGAGTGATGGTTCTCGTATACAGACTAGAACAGAGCCCTTAG
TATTAACGCAACACAATTATAATAATATTACTTTAGATAAGTGTGTTGACTATAATATATATGGCAGAGTAGGCC
AAGGTTTTATTACTAATGTGACTGATTCTGCTGCTAATTTTAGTTATTTAGCAGATGGTGGGTTAGCTATTTTA
GATACTTCGGGTGCCATAGATGTCTTTGTTGTACAGGGCAGCTATGGTCTTAATTATTACAAGGTCAATCCTTG
TGAAGATGTTAACAAACAGTTTGTAGTGTCTGGTGGCAATATAGTTGGCATTCTTACTTCTAGAAATGAAACA
GGTTCTGAACAGGTTGAGAACCAGTTTTATGTTAAGTTAACCAATAGCTCACATCGTCGCAGGCGTT
>CK_CH_YN_MC-1_20200928.seq
ATGTTGGGGAAGTCACTGTTTTTAGTGACCATTTTGTGTGCACTATGTAGTGCAAATTTATTCGATCCTGCTAA
TACTTATGTGTACTACTACCAAAGTGCCTTTAGGCCTCCAAATGGATGGCACCTACAAGGGGGTGCTTATGCA
GTAGTCAATTCCACTAATTATACTAATAATGCCGGTTCTGCACAACATTGCACTGTTGGTGTTATTAAGGACGT
CTATAATCAAAGTGCGGCTTCCATAGCTATGACAGCACCTCTTCAGGGTATGGCTTGGTCTAAGTCACAATTT
TGTAGTGCACACTGTAACTTTTCTGAAATTACAGTTTTTGTCACACATTGTTATAGTAGTGGTAGCGGGTCTTG
TCCTATAACAGGCATGATTGCACGTGATCATATTCGTATTTCTGCAATGAAAAATGGTACTTTATTTTATAATTTA
ACAGTTAGCGTATCTAAATACCCTAATTTTAAATCTTTTCAATGCGTTAATAATCTCACATCTGTTTATCTAAATG
GTGATCTTGTTTTTACTTCCAACAAAACTACTGATGTTACGTCAGCAGGTGTGTATTTTAAAGCAGGTGGACC
TGTAAATTATAGTATTATGAAAGAATTTAAGGTTCTTGCTTACTTTGTTAATGGTACAGCACAAGATGTAATTTT
GTGCGACAATTCCCCCAAGGGTTTGCTAGCTTGTCAATATAACACTGGCAATTTTTCAGATGGCTTTTATCCTT
TTACTAATAGTACTTTAGTTAGGGAAAAGTTCATCGTATATCGCGAAAGTAGTGTTAATACTACTCTGGCGTTA

ACTAATTTCACTTTTACTAATGTAAGTAATGCACAGCCTAATAGTGGTGGTGTTAATACTTTTCATCTATATCAA
ACACAAACAGCTCAGAGTGGTTATTATAATTTTAATTTGTCATTTCTGAGTCAGTTTGTGTATAAGGCAAGTGA
TTTTATGTATGGGTCCTACCACCCTAGTTGTTCTTTTAGACCAGAAACCATTAATAGTGGTTTGTGGTTTAATT
CTTTGTCAGTTTCTCTAGCTTACGGACCACTTCAAGGTGGGTGTAAGCAGTCAGTTTTTAGTGGTAGGGCAA
CGTGTTGCTATGCCTACTCTTACAATGGCCCGATAGCCTGTAAAGGTGTTTATTCAGGCGAATTACGGACTAA
TTTTGAATGTGGATTGCTGATTTATGTTACTAAGAGTGATGGTTCTCGTATACAGACTAGAACAGAGCCCTTA
GTATTAACGCAACACAATTATAATAATATTACTTTAGATAAGTGTGTTGACTATAATATATATGGCAGAGTAGGC
CAAGGTTTTATTACTAATGTGACTGATTCTGCTGCTAATTTTAGTTATTTAGCAGATGGTGGGTTAGCTATTTTA
GATACTTCGGGTGCCATAGATGTCTTTGTTGTACAGGGCAGCTATGGTCTTAATTATTACAAGGTCAATCCTTG
TGAAGATGTTAACAAACAGTTTGTAGTGTCTGGTGGCAATATAGTTGGCATTCTTACTTCTAGAAATGAAACA
GGTTCTGAACAGGTTGAGAACCAGTTTTATGTTAAGTTAACCAATAGCTCACATCGTCGCAGGCGT
>CK_CH_GX_WM_LY-LCW20200628.seq
ATGTTGGGGAAGTCACTGTTTTTAGTGACCATTTTGTGTGCACTATGTAGTGCAAATTTATTCGATCCTGCTAA
TACTTATGTGTACTACTACCAAAGTGCCTTTAGGCCTCCAAATGGATGGCACCTACAAGGGGGTGCTTATGCA
GTAGTCAATTCCACTAATTATACTAATAATGCCGGTTCTGCACAACATTGCACTGTTGGTGTTATTAAGGACGT
CTATAATCAAAGTGCGGCTTCCATAGCTATGACAGCACCTCTTCAGGGTATGGCTTGGTCTAAGTCACAATTT
TGTAGTGCACACTGTAACTTTTCTGAAATTACAGTTTTTGTCACACATTGTTATAGTAGTGGTAGCGGGTCTTG
TCCTATAACAGGCATGATTGCACGTGATCATATTCGTATTTCTGCAATGAAAAATGGTACTTTATTTTATAATTTA
ACAGTTAGCGTATCTAAATACCCTAATTTTAAATCTTTTCAATGCGTTAATAATTTCACATCTGTTTATCTAAATG
GTGATCTTGTTTTTACTTCCAACAAAACTACTGATGTTACGTCAGCAGGTGTGTATTTTAAAGCAGGTGGACC
TGTAAATTATAGTATTATGAAAGAATTTAAGGTTCTTGCTTACTTTGTTAATGGTACAGCACAAGATGTAATTTT
GTGCGACAATTCCCCCAAGGGTTTGCTAGCTTGTCAATATAACACTGGCAATTTTTCAGATGGCTTTTATCCTT
TTACTAATAGTACTTTAGTTAGGGAAAAGTTCATCGTATATCGCGAAAGTAGTGTTAATACTACTCTGGCGTTA
ACTAATTTCACTTTTACTAATGTAAGTAATGCACAGCCTAATAGTGGTGGTGTTAATACTTTTCATCTATATCAA
ACACAAACAGCTCAGAGTGGTTATTATAATTTTAATTTTTCATTTCTGAGTCAGTTTGTGTATAAGGCAAGTGA
TTTTATGTATGGGTCCTACCACCCTAGTTGTTCTTTTAGACCAGACACCATTAATAGTGGTTTGTGGTTTAATTC
TTTGTCAGTTTCTCTAGCTTACGGACCACTTCAAGGTGGGTGTAAGCAGTCAGTTTTTAGTGGTAGGGCAAC
GTGTTGCTATGCCTACTCTTACAATGGCCCGATAGCCTGTAAAGGTGTTTATTCAGGCGAATTACGGACTAAT
TTTGAATGTGGATTGCTGATTTATGTTACTAAGAGTGATGGTTCTCGTATACAGACTAGAACAGAGCCCTTAG
TATTAACGCAACACAATTATAATAATATTACTTTAGATAAGTGTGTTGACTATAATATATATGGCAGAGTAGGCC
AAGGTTTTATTACTAATGTGACTGATTCTGCTGCTAATTTTAGTTATTTAGCAGATGGTGGGTTAGCTATTTTA
GATACTTCGGGTGCCATAGATGTCTTTGTTGTACAGGGCAGCTATGGTCTTAATTATTACAAGGTCAATCCTTG
TGAAGATGTTAACAAACAGTTTGTAGTGTCTGGTGGCAATATAGTTGGCATTCTTACTTCTAGAAATGAAACA
GGTTCTGAACAGGTTGAGAACCAGTTTTATGTTAAGTTAACCAATAGCTCACATCGTCGCAGGCGT
>CK_CH_GX_WM_LY-XXZ_20200608.seq
ATGTTGGGGAAGTCACTGTTTTTAGTGACCATTTTGTGTGCACTATGTAGTGCAAATTTATTCGATCCTGCTAA
TACTTATGTGTACTACTACCAAAGTGCCTTTAGGCCTCCAAATGGATGGCACCTACAAGGGGGTGCTTATGCA
GTAGTCAATTCCACTAATTATACTAATAATGCCGGTTCTGCACAACATTGCACTGTTGGTGTTATTAAGGACGT
CTATAATCAAAGTGCGGCTTCCATAGCTATGACAGCACCTCTTCAGGGTATGGCTTGGTCTAAGTCACAATTT
TGTAGTGCACACTGTAACTTTTCTGAAATTACAGTTTTTGTCACACATTGTTATAGTAGTGGTAGCGGGTCTTG
TCCTATAACAGGCATGATTGCACGTGATCATATTCGTATTTCTGCAATGAAAAATGGTACTTTATTTTATAATTTA
ACAGTTAGCGTATCTAAATACCCTAATTTTAAATCTTTTCAATGCGTTAATAATTTCACATCTGTTTATCTAAATG
GTGATCTTGTTTTTACTTCCAACAAAACTACTGATGTTACGTCAGCAGGTGTGTATTTTAAAGCAGGTGGACC
TGTAAATTATAGTATTATGAAAGAATTTAAGGTTCTTGCTTACTTTGTTAATGGTACAGCACAAGATGTAATTTT

GTGCGACAATTCCCCCAAGGGTTTGCTAGCTTGTCAATATAACACTGGCAATTTTTCAGATGGCTTTTATCCTT
TTACTAATAGTACTTTAGTTAGGGAAAAGTTCATCGTATATCGCGAAAGTAGTGTTAATACTACTCTGGCGTTA
ACTAATTTCACTTTTACTAATGTAAGTAATGCACAGCCTAATAGTGGTGGTGTTAATACTTTTCATCTATATCAA
ACACAAACAGCTCAGAGTGGTTATTATAATTTTAATTTGTCATTTCTGAGTCAGTTTGTGTATAAGGCAAGTGA
TTTTATGTATGGGTCCTACCACCCTAGTTGTTCTTTTAGACCAGACACCATTAATAGTGGTTTGTGGTTTAATTC
TTTGTCAGTTTCTCTAGCTTACGGACCACTTCAAGGTGGGTGTAAGCAGTCAGTTTTTAGTGGTAGGGCAAC
GTGTTGCTATGCCTACTCTTACAATGGCCCGATAGCCTGTAAAGGTGTTTATTCAGGCGAATTACGGACTAAT
TTTGAATGTGGATTGCTGATTTATGTTACTAAGAGTGATGGTTCTCGTATACAGACTAGAACAGAGCCCTTAG
TATTAACGCAACACAATTATAATAATATTACTTTAGATAAGTGTGTTGACTATAATATATATGGCAGAGTAGGCC
AAGGTTTTATTACTAATGTGACTGATTCTGCTGCTAATTTAGTTATTTAGCAGATGGTGGGTTAGCTATTTTA
GATACTTCGGGTGCCATAGATGTCTTTGTTGTACAGGGCAGCTATGGTCTTAATTATTACAAGGTCAATCCTTG
TGAAGATGTTAACAAACAGTTTGTAGTGTCTGGTGGCAATATAGTTGGCATTCTTACTTCTAGAAATGAAACA
GGTTCTGAACAGGTTGAGAACCAGTTTTATGTTAAGTTAACCAATAGCTCACATCGTCGCAGGCGT
>CK_CH_GX_QZ_LY-LYJ_20200730.seq
ATGTTGGGGAAGTCACTGTTTTTAGTGACCATTTTGTGTGCACTATGTAGTGCAAATTTATTCGATCCTGCTAA
TACTTATGTGTACTACTACCAAAGTGCCTTTAGGCCTCCAAATGGATGGCACCTACAAGGGGGTGCTTATGCA
GTAGTCAATTCCACTAATTATACTAATAATGCCGGTTCTGCACAACATTGCACTGTTGGTGTTATTAAGGACGT
CTATAATCAAAGTGCGGCTTCCATAGCTATGACAGCACCTCTTCAGGGTATGGCTTGGTCTAAGTCACAATTT
TGTAGTGCACACTGTAACTTTTCTGAAATTACAGTTTTTGTCACACATTGTTATAGTAGTGGTAGCGGGTCTTG
TCCTATAACAGGCATGATTGCACGTGATCATATTCGTATTTCTGCAATGAAAAATGGTACTTTATTTTATAATTTA
ACAGTTAGCGTATCTAAATACCCTAATTTTAAATCTTTTCAATGCGTTAATAATTTCACATCTGTTTATCTAAATG
GTGATCTTGTTTTTACTTCCAACAAAACTACTGATGTTACGTCAGCAGGTGTGTATTTTAAAGCAGGTGGACC
TGTAAATTATAGTATTATGAAAGAATTTAAGGTTCTTGCTTACTTTGTTAATGGTACAGCACAAGATGTAATTTT
GTGCGACAATTCCCCCAAGGGTTTGCTAGCTTGTCAATATAACACTGGCAATTTTTCAGATGGCTTTTATCCTT
TTACTAATAGTACTTTAGTTAGGGAAAAGTTCATCGTATATCGCGAAAGTAGTGTTAATACTACTCTGGCGTTA
ACTAATTTCACTTTTACTAATGTAAGTAATGCACAGCCTAATAGTGGTGGTGTTAATACTTTTCATCTATATCAA
ACACAAACAGCTCAGAGTGGTTATTATAATTTTAATTTGTCATTTCTGAGTCAGTTTGTGTATAAGGCAAGTGA
TTTTATGTATGGGTCCTACCACCCTAGTTGTTCTTTTAGACCAGACACCATTAATAGTGGTTTGTGGTTTAATTC
TTTGTCAGTTTCTCTAGCTTACGGACCACTTCAAGGTGGGTGTAAGCAGTCAGTTTTTAGTGGTAGGGCAAC
GTGTTGCTATGCCTACTCTTACAATGGCCCGATAGCCTGTAAAGGTGTTTATTCAGGCGAATTACGGACTAAT
TTTGAATGTGGATTGCTGATTTATGTTACTAAGAGTGATGGTTCTCGTATACAGACTAGAACAGAGCCCTTAG
TATTAACGCAACACAATTATAATAATATTACTTTAGATAAGTGTGTTGACTATAATATATATGGCAGAGTAGGCC
AAGGTTTTATTACTAATGTGACTGATTCTGCTGCTAATTTAGTTATTTAGCAGATGGTGGGTTAGCTATTTTA
GATACTTCGGGTGCCATAGATGTCTTTGTTGTACAGGGCAGCTATGGTCTTAATTATTACAAGGTCAATCCTTG
TGAAGATGTTAACAAACAGTTTGTAGTGTCTGGTGGCAATATAGTTGGCATTCTTACTTCTAGAAATGAAACA
GGTTCTGAACAGGTTGAGAACCAGTTTTATGTTAAGTTAACCAATAGCTCACATCGTCGCAGGCGT
>CK_CH_GX_QZ_LY-HWW_20200513.seq
ATGTTGGGGAAGTCACTGTTTTTAGTGACCATTTTGTGTGCACTATGTAGTGCAAATTTATTCGATCCTGCTAA
TACTTATGTGTACTACTACCAAAGTGCCTTTAGGCCTCCAAATGGATGGCACCTACAAGGGGGTGCTTATGCA
GTAGTCAATTCCACTAATTATACTAATAATGCCGGTTCTGCACAACATTGCACTGTTGGTGTTATTAAGGACGT
CTATAATCAAAGTGCGGCTTCCATAGCTATGACAGCACCTCTTCAGGGTATGGCTTGGTCTAAGTCACAATTT
TGTAGTGCACACTGTAACTTTTCTGAAATTACAGTTTTTGTCACACATTGTTATAGTAGTGGTAGCGGGTCTTG
TCCTATAACAGGCATGATTGCACGTGATCATATTCGTATTTCTGCAATGAAAAATGGTACTTTATTTTATAATTTA
ACAGTTAGCGTATCTAAATACCCTAATTTTAAATCTTTTCAATGCGTTAATAATCTCACATCTGTTTATCTAAATG

GTGATCTTGTTTTTACTTCCAATAAAACTACTGATGTTACGTCAGCAGGTGTGTATTTTAAAGCAGGTGGACC
TGTAAATTATAGTATTATGAAAGAATTTAAGGTTCTTGCTTACTTTGTTAATGGTACAGCACAAGATGTAATTTT
GTGCGACAATTCCCCCAAGGGTTTGCTAGCTTGTCAATATAACACTGGCAATTTTTCAGATGGCTTTTATCCTT
TTACTAATAGTACTTTAGTTAGGGAAAAGTTCATCGTATATCGCGAAAGTAGTGTTAATACTACTCTGGCGTTA
ACTAATTTCACTTTTACTAATGTAAGTAATGCACAGCCTAATAGTGGTGGTGTTAATACTTTTCATCTATATCAA
ACACAAACAGCTCAGAGTGGTTATTATAATTTTTAATTTGTCATTTCTGAGTCAGTTTGTGTATAAGGCAAGTG
ATTTTATGTATGGGTCCTACCACCCTAGTTGTTCTTTTAGACCAGACACCATTAATAGTGGTTTGTGGTTTAATT
CTTTGTCAGTTTCTCTAGCTTACGGACCACTTCAAGGTGGGTGTAAGCAGTCAGTTTTTAGTGGTAGGGCAA
CGTGTTGCTATGCCTACTCTTACAATGGCCCGATAGCCTGTAAAGGTGTTTATTCAGGCGAATTACGGACTAA
TTTTGAATGTGGATTGTTGATTTATGTTACTAAGAGTGATGGTTCTCGTATACAGACTAGAACAGAGCCCTTA
GTATTAACGCAACACAATTATAATAATATTACTTTAGATAAGTGTGTTGACTATAATATATATGGCAGAGTAGGC
CAAGGTTTTATTACTAATGTGACTGATTCTGCTGCTAATTTTAGTTATTTAGCAGATGGTGGGTTAGCTATTTTA
GATACTTCGGGTGCCATAGATGTCTTTGTTGTACAGGGCAGCTATGGTCTTAATTATTACAAGGTCAATCCTTG
TGAAGATGTTAATAAACAGTTTGTAGTGTCTGGTGGCAATATAGTTGGCATTCTTACTTCTAGAAATGAAACA
GGTTCTGAACAGGTTGAGAACCAGTTTTATGTTAAGTTAACCAATAGCTCACATCGTCGCAGGCGT
>CK_CH_GX_QZ_LY-ZRW_20200513.seq
ATGTTGGGGAAGTCACTGTTTTTAGTGACCATTTTGTGTGCACTATGTAGTGCAAATTTATTCGATCCTGCTAA
TACTTATGTGTACTACTACCAAAGTGCCTTTAGGCCTCCAAATGGATGGCACCTACAAGGGGGTGCTTATGCA
GTAGTCAATTCCACTAATTATACTAATAATGCCGGTTCTGCACAACATTGCACTGTTGGTGTTATTAAGGACGT
CTATAATCAAAGTGCGGCTTCCATAGCTATGACAGCACCTCTTCAGGGTATGGCTTGGTCTAAGTCACAATTT
TGTAGTGCACACTGTAACTTTTCTGAAATTACAGTTTTTGTCACACATTGTTATAGTAGTGGTAGCGGGTCTTG
TCCTATAACAGGCATGATTGCACGTGATCATATTCGTATTTCTGCAATGAAAAATGGTACTTTATTTTATAATTTA
ACAGTTAGCGTATCTAAATACCCTAATTTTAAATCTTTTCAATGCGTTAATAATCTCACATCTGTTTATCTAAATG
GTGATCTTGTTTTTACTTCCAACAAAACTACTGATGTTACGTCAGCAGGTGTGTATTTTAAAGCAGGTGGACC
TGTAAATTATAGTATTATGAAAGAATTTAAGGTTCTTGCTTACTTTGTTAATGGTACAGCACAAGATGTAATTTT
GTGCGACAATTCCCCCAAGGGTTTGCTAGCTTGTCAATATAACACTGGCAATTTTTCAGATGGCTTTTATCCTT
TTACTAATAGTACTTTAGTTAGGGAAAAGTTCATCGTATATCGCGAAAGTAGTGTTAATACTACTCTGGCGTTA
ACTAATTTCACTTTTACTAATGTAAGTAATGCACAGCCTAATAGTGGTGGTGTTAATACTTTTCATCTATATCAA
ACACAAACAGCTCAGAGTGGTTATTATAATTTTAATTTGTCATTTCTGAGTCAGTTTGTGTATAAGGCAAGTGA
TTTTATGTATGGGTCCTACCACCCTAGTTGTTCTTTTAGACCAGACACCATTAATAGTGGTTTGTGGTTTAATTC
TTTGTCAGTTTCTCTAGCTTACGGACCACTTCAAGGTGGGTGTAAGCAGTCAGTTTTTAGTGGTAGGGCAAC
GTGTTGCTATGCCTACTCTTACAATGGCCCGATAGCCTGTAAAGGTGTTTATTCAGGCGAATTACGGACTAAT
TTTGAATGTGGATTGCTGATTTATGTTACTAAGAGTGATGGTTCTCGTATACAGACTAGAACAGAGCCCTTAG
TATTAACGCAACACAATTATAATAATATTACTTTAGATAAGTGTGTTGACTATAATATATATGGCAGAGTAGGCC
AAGGTTTTATTACTAATGTGACTGATTCTGCTGCTAATTTTAGTTATTTAGCAGATGGTGGGTTAGCTATTTTA
GATACTTCGGGTGCCATAGATGTCTTTGTTGTACAGGGCAGCTATGGTCTTAATTATTACAAGGTCAATCCTTG
TGAAGATGTTAACAAACAGTTTGTAGTGTCTGGTGGCAATATAGTTGGCATTCTTACTTCTAGAAATGAAACA
GGTTCTGAACAGGTTGAGAACCAGTTTTATGTTAAGTTAACCAATAGCTCACATCGTCGCAGGCGT
>CK_CH_GX_WM_LY-LMJ20200628.seq
ATGTTGGGGAAGTCACTGTTTTTAGTGACCATTTTGTGTGCACTATGTAGTGCAAATTTATTCGATCCTGCTAA
TACTTATGTGTACTACTACCAAAGTGCCTTTAGGCCTCCAAATGGATGGCACCTACAAGGGGGTGCTTATGCA
GTAGTCAATTCCACTAATTATACTAATAATGCCGGTTCTGCACAACATTGCACTGTTGGTGTTATTAAGGACGT
CTATAATCAAAGTGCGGCTTCCATAGCTATGACAGCACCTCTTCAGGGTATGGCTTGGTCTAAGTCACAATTT
TGTAGTGCACACTGTAACTTTTCTGAAATTACAGTTTTTGTCACACATTGTTATAGTAGTGGTAGCGGGTCTTG

TCCTATAACAGGCATGATTGCACGTGATCATATTCGTATTTCTGCAATGAAAAATGGTACTTTATTTTATAATTTA
ACAGTTAGCGTATCTAAATACCCTAATTTTAAATCTTTTCAATGCGTTAATAATCTCACATCTGTTTATCTAAATG
GTGATCTTGTTTTTACTTCCAACAAAACTACTGATGTTACGTCAGCAGGTGTGTATTTTAAAGCAGGTGGACC
TGTAAATTATAGTATTATGAAAGAATTTAAGGTTCTTGCTTACTTTGTTAATGGTACAGCACAAGATGTAATTTT
GTGCGACAATTCCCCCAAGGGTTTGCTAGCTTGTCAATATAACACTGGCAATTTTTCAGATGGCTTTTATCCTT
TTACTAATAGTACTTTAGTTAGGGAAAAGTTCATCGTATATCGCGAAAGTAGTGTTAATACTACTCTGGCGTTA
ACTAATTTCACTTTTACTAATGTAAGTAATGCACAGCCTAATAGTGGTGGTGTTAATACTTTTCATCTATATCAA
ACACAAACAGCTCAGAGTGGTTATTATAATTTTAATTTGTCATTTCTGAGTCAGTTTGTGTATAAGGCAAGTGA
TTTTATGTATGGGTCCTACCACCCTAGTTGTTCTTTTAGACCAGACACCATTAATAGTGGTTTGTGGTTTAATTC
TTTGTCAGTTTCTCTAGCTTACGGACCACTTCAAGGTGGGTGTAAGCAGTCAGTTTTTAGTGGTAGGGCAAC
GTGTTGCTATGCCTACTCTTACAATGGCCCGATAGCCTGTAAAGGTGTTTATTCAGGCGAATTACGGACTAAT
TTTGAATGTGGATTGCTGATTTATGTTACTAAGAGTGATGGTTCTCGTATACAGACTAGAACAGAGCCCTTAG
TATTAACGCAACACAATTATAATAATATTACTTTAGATAAGTGTGTTGACTATAATATATATGGCAGAGTAGGCC
AAGGTTTTATTACTAATGTGACTGATTCTGCTGCTAATTTTAGTTATTTAGCAGATGGTGGGTTAGCTATTTTA
GATACTTCGGGTGCCATAGATGTCTTTGTTGTACAGGGCAGCTATGGTCTTAATTATTACAAGGTCAATCCTTG
TGAAGATGTTAACAAACAGTTTGTAGTGTCTGGTGGCAATATAGTTGGCATTCTTACTTCTAGAAATGAAACA
GGTTCTGAACAGGTTGAGAACCAGTTTTATGTTAAGTTAACCAATAGCTCACATCGTCGCAGGCGT
>CK_CH_GX_WM-LY_LRQ20200616.seq
ATGTTGGGGAAGTCACTGTTTTTAGTGACCATTTTGTGTGCACTATGTAGTGCAAATTTATTCGATCCTGCTAA
TACTTATGTGTACTACTACCAAAGTGCCTTTAGGCCTCCAAATGGATGGCACCTACAAGGGGGTGCTTATGCA
GTAGTCAATTCCACTAATTATACTAATAATGCCGGTTCTGCACAACATTGCACTGTTGGTGTTATTAAGGACGT
CTATAATCAAAGTGCGGCTTCCATAGCTATGACAGCACCTCTTCAGGGTATGGCTTGGTCTAAGTCACAATTT
TGTAGTGCACACTGTAACTTTTCTGAAATTACAGTTTTTGTCACACATTGTTATAGTAGTGGTAGCGGGTCTTG
TCCTATAACAGGCATGATTGCACGTGATCATATTCGTATTTCTGCAATGAAAAATGGTACTTTATTTTATAATTTA
ACAGTTAGCGTATCTAAATACCCTAATTTTAAATCTTTTCAATGCGTTAATAATCTCACATCTGTTTATCTAAATG
GTGATCTTGTTTTTACTTCCAACAAAACTACTGATGTTACGTCAGCAGGTGTGTATTTTAAAGCAGGTGGACC
TGTAAATTATAGTATTATGAAAGAATTTAAGGTTCTTGCTTACTTTGTTAATGGTACAGCACAAGATGTAATTTT
GTGCGACAATTCCCCCAAGGGTTTGCTAGCTTGTCAATATAACACTGGCAATTTTTCAGATGGCTTTTATCCTT
TTACTAATAGTACTTTAGTTAGGGAAAAGTTCATCGTATATCGCGAAAGTAGTGTTAATACTACTCTGGCGTTA
ACTAATTTCACTTTTACTAATGTAAGTAATGCACAGCCTAATAGTGGTGGTGTTAATACTTTTCATCTATATCAA
ACACAAACAGCTCAGAGTGGTTATTATAATTTTAATTTGTCATTTCTGAGTCAGTTTGTGTATAAGGCAAGTGA
TTTTATGTATGGGTCCTACCACCCTAGTTGTTCTTTTAGACCAGACACCATTAATAGTGGTTTGTGGTTTAATTC
TTTGTCAGTTTCTCTAGCTTACGGACCACTTCAAGGTGGGTGTAAGCAGTCAGTTTTTAGTGGTAGGGCAAC
GTGTTGCTATGCCTACTCTTACAATGGCCCGATAGCCTGTAAAGGTGTTTATTCAGGCGAATTACGGACTAAT
TTTGAATGTGGATTGCTGATTTATGTTACTAAGAGTGATGGTTCTCGTATACAGACTAGAACAGAGCCCTTAG
TATTAACGCAACACAATTATAATAATATTACTTTAGATAAGTGTGTTGAATATAATATATATGGCAGAGTAGGCC
AAGGTTTTATTACTAATGTGACTGATTCTGCTGCTAATTTTAGTTATTTAGCAGATGGTGGGTTAGCTATTTTA
GATACTTCGGGTGCCATAGATGTCTTTGTTGTACAGGGCAGCTATGGTCTTAATTATTACAAGGTCAATCCTTG
TGAAGATGTTAACAAACAGTTTGTAGTGTCTGGTGGCAATATAGTTGGCATTCTTACTTCTAGAAATGAAACA
GGTTCTGAACAGGTTGAGAACCAGTTTTATGTTAAGTTAACCAATAGCTCACATCGTCGCAGGCGT
>CK_CH_HLJ_HEB_YCY_20211117.seq
ATGTTGGGGAAGTCACTGTTTTTAGTGACCATTTTGTGTGCACTATGTAGTGCAAATTTATTCGATCCTGCTAA
TACTTATGTGTACTACTACCAAAGTGCCTTTAGGCCTCCAAATGGATGGCACCTACAAGGGGGTGCTTATGCA
GTAGTCAATTCCACTAATTATACTAATAATGCCGGTTCTGCACAACATTGCACTGTTGGTGTTATTAAGGACGT

CTATAATCAAAGTGCGGCTTCCATAGCTATGACAGCACCTCTTCAGGGTATGGCTTGGTCTAAGTCACAATTT
TGTAGTGCACACTGTAACTTTTCTGAAATTACAGTTTTTGTCACACATTGTTATAGTAGTGGTAGCGGGTCTTG
TCCTATAACAGGCATGATTGCACGTGATCATATTCGTATTTCTGCAATGAAAAATGGTACTTTATTTTATAATTTA
ACAGTTAGCGTATCTAAATACCCTAATTTTAAATCTTTTCAATGCGTTAATAATCTCACATCTGTTTATCTAAATG
GTGATCTTGTTTTTACTTCCAACAAAACTACTGATGTTACGTCAGCAGGTGTGTATTTTAAAGCAGGTGGACC
TGTAAATTATAGTATTATGAAAGAATTTAAGGTTCTTGCTTACTTTGTTAATGGTACAGCACAAGATGTAATTTT
GTGCGACAATTCCCCCAAGGGTTTGCTAGCTTGTCAATATAACACTGGCAATTTTTCAGATGGCTTTTATCCTT
TTACTAATAGTACTTTAGTTAGGGAAAAGTTCATCGTATATCGCGAAAGTAGTGTTAATACTACTCTGGCGTTA
ACTAATTTCACTTTTACTAATGTAAGTAATGCACAGCCTAATAGTGGTGGTGTTAATACTTTTCATCTATATCAA
ACACAAACAGCTCAGAGTGGTTATTATAATTTTAATTTGTCATTTCTGAGTCAGTTTGTGTATAAGGCAAGTGA
TTTTATGTATGGGTCCTACCACCCTAGTTGTTCTTTTAGACCAGACACCATTAATAGTGGTTTGTGGTTTAATTC
TTTGTCAGTTTCTCTAGCTTACGGACCACTTCAAGGTGGGTGTAAGCAGTCAGTTTTTAGTGGTAGGGCAAC
GTGTTGCTATGCCTACTCTTACAATGGCCCGATAGCCTGTAAAGGTGTTTATTCAGGCGAATTATGGACTAATT
TTGAATGTGGATTGCTGATTTATGTTACTAAGAGTGATGGTTCTCGTATACAGACTAGAACAGAGCCCTTAGT
ATTAACGCAACACAATTATAATAATATTACTTTAGATAAGTGTGTTGACTATAATATATATGGCAGAGTAGGCCA
AGGTTTTATTACTAATGTGACTGATTCTGCTGCTAATTTTAGTTATTTAGCAGATGGTGGGTTAGCTATTTTAGA
TACTTCGGGTGCCATAGATGTCTTTGTTGTACAGGGCAGCTATGGTCTTAATTATTACAAGGTCAATCCTTGTG
AAGATGTTAACAAACAGTTTGTAGTGTCTGGTGGCAATATAGTTGGCATTCTTACTTCTAGAAATGAAACAGG
TTCTGAACAGGTTGAGAACCAGTTTTATGTTAAGTTAACCAATAGCTCACATCGTCGCAGGCGT
>CK_CH_ZJ_TZ_LH-TBY-1_20210914.seq
ATGTTGGGGAAGTCACTGTTTTTAGTGACCATTTTGTGTGCACTATGTAGTGCAAATTTATTCGATCCTGCTAA
TACTTATGTGTACTACTACCAAAGTGCCTTTAGGCCTCCAAATGGATGGCACCTACAAGGGGGTGCTTATGCA
GTAGTCAATTCCACTAATTATACTAATAATGCCGGTTCTGCACAACATTGCACTGTTGGTGTTATTAAGGACGT
CTATAATCAAAGTGCGGCTTCCATAGCTATGACAGCACCTCTTCAGGGTATGGCTTGGTCTAAGTCACAATTT
TGTAGTGCACACTGTAACTTTTCTGAAATTACAGTTTTTGTCACACATTGTTATAGTAGTGGTAGCGGGTCTTG
TCCTATAACAGGCATGATTGCACGTGATCATATTCGTATTTCTGCAATGAAAAATGGTACTTTATTTTATAATTTA
ACAGTTAGCGTATCTAAATACCCTAATTTTAAATCTTTTCAATGCGTTAATAATCTCACATCTGTTTATCTAAATG
GTGATCTTGTTTTTACTTCCAACAAAACTACTGATGTTACGTCAGCAGGTGTGTATTTTAAAGCAGGTGGACC
TGTAAATTATAGTATTATGAAAGAATTTAAGGTTCTTGCTTACTTTGTTAATGGTACAGCACAAGATGTAATTTT
GTGCGACAATTCCCCCAAGGGTTTGCTAGCTTGTCAATATAACACTGGCAATTTTTCAGATGGCTTTTATCCTT
TTACTAATAGTACTTTAGTTAGGGAAAAGTTCATCGTATATCGCGAAAGTAGTGTTAATACTACTCTGGCGTTA
ACTAATTTCACTTTTACTAATGTAAGTAATGCACAGCCTAATAGTGGTGGTGTTAATACTTTTCATCTATATCAA
ACACAAACAGCTCAGAGTGGTTATTATAATTTTAATTTGTCATTTCTGAGTCAGTTTGTGTATAAGGCAAGTGA
TTTTATGTATGGGTCCTACCACCCTAGTTGTTCTTTTAGACCAGACACCATTAATAGTGGTTTGTGGTTTAATTT
TTTGTCAGTTTCTCTAGCTTACGGACCACTTCAAGGTGGGTGTAAGCAGTCAGTTTTTAGTGGTAGGGCAAC
GTGTTGCTATGCCTACTCTTACAATGGCCCGATAGCCTGTAAAGGTGTTTATTCAGGCGAATTACGGACTAAT
TTTGAATGTGGATTGCTGATTTATGTTACTAAGAGTGATGGTTCTCGTATACAGACTAGAACAGAGCCCTTAG
TATTAACGCAACACAATTATAATAATATTACTTTAGATAAGTGTGTTGACTATAATATATATGGCAGAGTAGGCC
AAGGTTTTATTACTAATGTGACTGATTCTGCTGCTAATTTTAGTTATTTAGCAGATGGTGGGTTAGCTATTTTA
GATACTTCGGGTGCCATAGATGTCTTTGTTGTACAGGGCAGCTATGGTCTTAATTATTACAAGGTCAATCCTTG
TGAAGATGTTAACAAACAGTTTGTAGTGTCTGGTGGCAATATAGTTGGCATTCTTACTTCTAGAAATGAAACA
GGTTCTGAACAGGTTGAGAACCAGTTTTATGTTAAGTTAACCAATAGCTCACATCGTCGCAGGCGT
>CK_CH_SD_FX-2_20210411.seq
ATGTTGGGGAAGTCACTGTTTTTAGTGACCATTTTGTGTGCACTATGTAGTGCAAATTTGTTTGATTCTGCCA

ATAATTATGTGTACTACTACCAAAGTGCCTTTAGGCCTCCAAATGGATGGCATTTGCAAGGGGGTGCTTATGC
AGTAGTGAATTCTACTAATTATACTAGTAATGCCGGTTCTGCAAGTGAGTGCACTGTTGGTATTATTAAGGACG
TCTATAATCAAAGTGCGGCTTCTATAGCTATGACAGCACCTCCTCGGGGTATGGCTTGGTCTAAGTCACAATTT
TGTAGTGCACACTGTAACTTTTCTGAAATTACAGTTTTTGTCACACATTGTTATAGTAGTGGTGCAGGGTCTT
GCCCTATAACAGGCATGATTGCACGTGACCATATTCGTATTTCTGCAATGAAAAATGGTTCTTTATTTTATAACT
TAACAGTTAGCGTATCTAAATACTCTAGGTTTAAGTCTTTTCAATGTGTTAACAACTTCACATCTGTTTATCTAA
ATGGTGATCTTGTTTTTACTTCCAACAAAACTACTGATGTTACGTCAGCAGGTGTGTATTTTAAAGCAGGTGG
ACCTGTAAATTATAGTGTTATGAAAGAATTTAAGGTTCTTGCTTATTTTGTTAATGGTACAGCACAAGATGTAA
TTTTGTGTGACAAGTCCCCCAAGGGTTTGCTAGCTTGTCAATATAACACTGGCAATTTTTCAGATGGCTTTTAT
CCTTTTACTAATACTACTTTAGTTAGGGAAAAGTTCACCGTATATCGCGAAAGTAGTGTTAATACTACTCTGGC
GTTAACTAATTTCACTTTTGCTAATGTAAGTAATGCACAGCCCAATAGTGGTGGTGTTAGTACTTTTCATTTATA
TCAAACACAAACAGCTCAGAGTGGTTATTATAATTTTAATTTGTCATTTCTGAGTCAGTTTGTGTATAAGGCAA
GTGATTTTATGTATGGATCTTACCACCCTAGGTGTTCTTTTAGGCCAGAAACCATTAATAGTGGTTTATGGTTT
AACTCCTTGTCAGTTTCTCTTACTTATGGGCCTCTACAGGGAGGGTGTAAGCAATCTGTTTTTAATGGTAGGG
CAACGTGTTGTTATGCCTACTCTTATAATGGCCCAAGGGCATGTAAAGGTGTTTATTCAGGTGAATTAAGCAA
TAATTTTGAATGTGGATTGCTGGTTTATGTTACTAAGAGTGATGGCTCTCGTATACAAACTAGAACGGAGCCC
TTAGTATTAACGCAACACAATTATAATAATATTACTTTAGATAAGTGTGTTGACTATAATATATATGGCAGAGTAG
GCCAAGGTTTTATTACTAATGTGACTGATTCTGCTGCTAATTTTAGTTATTTAGCAGATGGTGGGTTAGCTATT
TTAGATACTTCGGGTGCCATAGATGTTTTTGTTGCACAGGGCAGCTATGGTCCTAATTATTACAAGGTCAATCC
TTGTGAAGATGTTAACCAACAGTTTGTAGTGTCTGGTGGCAATATAGTTGGCATTCTTACTTCTAGAAATGAA
ACAGGTTCTGAACAGGTTGAGAACCAGTTTTATGTTAAGTTAACCAATAGCTCACATCGTCGTAGGCGT
>CK_CH_XJ_TK-4_20211230.seq
ATGTTGGGGAAGTCACTGTTTTTAGTGACCATTTTGTGTGCACTATGTAGTGCAAATTTGTTTGATCCTGCCA
ATAATTATGTGTACTACTACCAAAGTGCCTTTAGGCCTTCAATTGGATGGCATTTGCAAGGGGGTGCTTATGC
AGTAGTGAATTCTACTAATTATACTAGTAATGCCGGTTCTGCAAGTCAGTGCACTGTTGGTATTATTAAGGACG
TCTATAATCAAAGTGCGGCTTCCATAGCTATGACAGCACCCTCTCAGGGTATGGCTTGGTCTAAGTCACAATT
TTGTAGTGCACACTGTAACTTTTCTGAAATTACAGTTTTTGTCACACATTGTTATAGTAGTGGTACAGGGTCTT
GCCCTATAACAGGCATGATTGAACGTGATCATATTCGTATTTCTGCAATGAAAAATGGTTCTTTATTTTATAACT
TAACAGTTAGCGTATCTAAATACTCTAGGTTTAAGTCTTTTCAATGTGTTAACAACCTCACATCTGTTTATCTAA
ATGGTGATCTTGTTTTTACTTCCAACAAAACTACTGATGTTACGTCAGCAGGTGTGTATTTTAAAGCGGGTGG
ACCTGTAAATTATAGTGTTATGAAAGAATTTAAGGTTCTTGCTTATTTTGTTAATGGTACAGTACAAGATGTAA
TTTTGTGTGACAAGTCCCCCAAGGGTTTGCTAGCTTGTCAATATAACACTGGCAATTTTTCAGATGGCTTTTAT
CCTTTTACTAATACTACTTTAGTTAGGGAAAAGTTCATCGTATATCGTGAAAGTAGTGTTAATACTACTCTGGC
GTTAACTAATTTCACTTTTACTAATGTAAGTAATGCACAGCCTAATAGTGGTGGTGTTAATACTTTTCATCTATAT
CAAACACAAACAGCTCAGAGTGGTTATTATAATTTTAATTTGTCATTTCTGAGTCAGTTTGTGTATAAGGCAA
GCGATTTTATGTATGGGTCTTATCATCCTAGGTGTTCTTTTAGACCAGAAACCATTAATAGTGGTTTATGGTTTA
ATTCCTTGTCAGTTTCTCTTACTTATGGACCCCTACAGGGAGGGTGTAAGCAATCTGTTTTTAGTGGTAAGGC
AACGTGTTGTTATGCCTACTCTTATAATGGCCCTAGGGCATGTAAAGGTGTTTATTCAGGTGAATTAAGCAAG
ACTTTTGAATGTGGATTGCTGGTTTATGTTACTAAGAGTGATGGCTCTCGTATACAAACTAGAACAGAGCCCT
TAGTATTAACGCAACACAATTATAATAATGTTACTTTAGATAAGTGTGTTAACTATAATATATATGGCAGAGTGG
GCCAAGGTTTTATTACTAATGTGACTGATTCTGCTGCTAATTTTAGTTATTTAGCAGATGGTGGGTTAGCTATT
TTAGACACTTCGGGTGCCATAGATGTCTTTGTTGCACAGGGCAGCTATGGTCCTAATTATTACAAGGTCAATC
CTTGTGAAGATGTTAACCAACAGTTTGTAGTGTCTGGTGGCAATATAGTGGGCATTCTTACTTCTAGAAATGA
AACAGGTTCTGAACAGGTTGAGAACCAGTTTTATGTTAAGTTAACCAATAGCTCACATCGTCGTAGGCGT

>CK_CH_SD_WF_PDLH-2_20210524.seq
ATGTTGGGGAAGTCACTGTTTTTAGTGACCATTTTGTGTGCACTATGTAGTGCAAATTTGTTTGATTTTGTCAA
TAATTATGTGTACTACTACCAAAGTGCCTTTAGGCCTCCAAATGGATGGCATTTGCAAGGGGGTGCTTATGCA
GTAGTGAATTCTACTAATTATACTAGTAATGCCGGTTCTGCAAGTGAGTGCACTGTTGGTATTATTAAGGACGT
CTATAATCAAAGTGCGGCTTCCATAGCTATGACAGCACCTTCTCAGGGTATGGCTTGGTCTAAGTCACAATTT
TGTAGTGCACACTGTAACTTTTCTGAAATTACAGTTCTCGTCACACATTGTTATAGTAGTGGTACAGGGTCTTG
CCCTATAACAGGCATGATTGCACGTGGTCATATTCGTATTTCTGCAATGAAAAATGGTTTCTTATTTTATAACTT
AACAGTTAGCGTATCTAAATACTCTAGGTTTAAGTCTTTTCAATGTGTTAACAACTTCACATCTGTTTATCTAAA
TGGTGATCTTGTTTTTACTTCCAACAAAACTACTGATGTTACGTCAGCAGGTGTGTATTTTAAAGCAGGTGGA
CCTGTAAATTATAGTGTTATGAAAGAATTTAAGGTTCTTGCTTATTTTGTTAATGGTACAGCACAAGATGTAAT
TTTGTGTGACAAGTCCCCCAAGGGTTTGCTAGCTTGTCAATATAATACTGGCAATTTTTCAGATGGCTTTTATC
CTTTTACTAATACTACTTTAGTTAGGGAAAAGTTCATTGTATATCGTGAAAGTAGTGTTAATACTACTCTGGTGT
TAACTAATTTCACTTTTACTAATGTAAGTAATGCACAGCCTAATAGTGGTGGTGTTAATACTTTTCATCTATATCA
AACACAAACAGCTCAGAGTGGTTATTATAATTTTAATTTGTCATTTCTGAGTCAGTTTGTGTATAAGGCAAGT
GATTTTATGTATGGGTCTTATCATCCTAGGTGTTCTTTTAGACCAGAAACCATTAATAGTGGTTTATGGTTTAAT
TCCTTGTCAGTTTCTCTTACTTATGGACCCCTACAGGGAGGGTGTAAGCAATCTGTTTTTAGTGGTAAGGCAA
CGTGTTGTTATGCCTACTCTTATAATGGCCCTAGGGCATGTAAAGGTGTTTATTCAGGTGAATTAAGCAAGAC
TTTTGAATGTGGATTGCTGGTTTATGTTACTAAGAGTGATGGCTCTCGTATACAAACTAGAACAGAGCCCTTA
GTATTAACGCAACACAATTATAATAATGTTACTTTAGATAAGTGTGTTGACTATAATATATATGGCAGAGTAGGC
CAAGGTTTTATTACTAATGTGACTGATTCTGCTGCTAATTTTAGTTATTTAGCAGATGGTGGGTTAGCTATTTTA
GATACTTCGGGTGCCATAGATGTCTTTGTTGTACAGGGCAGCTATGGTTCTAATTATTACAAGGTCAATCCTTG
TGAAGATGTTAACCAACAGTTTGTAGTGTCTGGTGGTAATATAGTTGGCATTCTTACTTCTAGAAATGAAACA
GGTTCTGAACAGGTTGAGAACCAGTTTTATGTTAAGTTAACCAATAGCTCACATCGTCATAGGCGT
>CK_CH_ZJ_JXLH_WQY_20210513.seq
ATGTTGGGGAAGTCACTGTTTTTAGTGACCATTTTGTGTGCACTATGTAGTGCAAATTTGTTTGATTCTGCCA
ATAATTATGTGTACTACTACCAAAGTGCCTTTAGGCCTCCAAATGGATGGCATTTGCAAGGGGGTGCTTATGC
AGTAGTGAATTCTACTAATTATACTAGTAATGCCGGTTCTGCAAGTGAGTGCACTGTTGGTATTATTAAGGACG
TCTATAATCAAAGTGCGGCTTCCATAGCTATGACAGCACCTTCTCAGGGTATGGCTTGGTCTAAGTCACAATT
TTGTAGTGCACACTGTAACTTTTCTGAAATTACAGTTTTCGTCACACATTGTTTTAGTAGTGGTACAGGGTCTT
GCCCTATAACAGGCATGATTGCACGTGATCATATTCGTATTTCTGCAATGAAAAATGGTTCTTTATTTTATAACT
TAACAGTTAGCGTATCTAAATACTCTAGGTTTAAGTCTTTTCAATGTGTTAACAACCTCACATCTGTTTATTTAA
ATGGTGATCTTGTTTTTACTTCCAATAAAACTACTGATGTTACGTCAGCAGGTGTGTATTTTAAAGCAGGTGG
ACCTGTAAATTATAGTGTTATGAAAGAATTTAAGGTTCTCGCTTACTTTGTTAATGGTACAGCACAAGATGTAA
TTTTGTGTGACAAGTCCCCCAAGGGTTTGCTAGCTTGTCAATATAACACTGGCAATTTTTCAGATGGCTTTTAT
CCTTTTACTAATACTACTTTAGTTAGGGAAAAGTTCATCGTATATCGTGAAAGTAGTGTTAATACTACTCTGGC
GTTAATTAATTTCACTTTTATTAATGTAAGTAATGCACAGCCTAATAGTGGTGGTGTTAATACTTTTCATCTATAT
CAAACACAAACAGCTCAGAGTGGTTATTATAATTTTAATTTGTCATTTCTGAGTCAGTTTGTGTATAAGGCAA
GTGATTTTATGTATGGGTCTTATCATCCTAGGTGTTTTTTTAGACCAGAAACCATTAATAGTGGTTTATGGTTTA
ATTCCTTGTCAGTTTCTCTTACTTATGGACCCCTACAGGGAGGGTGTAAGCAATCTGTTTTTAGTGGTAAGGC
AACGTGTTGTTATGCCTACTCTTATAATGGCCCTAGGGCATGTAAAGGTGTTTATTCAGGTGAATTAAGCAAG
ACTTTTGAATGTGGATTGCTGGTTTATGTTACTAAGAGTGATGGCTCTCGTATACAAACTAGAACAGAGCCCT
TAGTATTAACGCAACACAATTATAATAATGTTACTTTAGATAAGTGTGTTGACTATAATATATATGGCAGAGTAG
GCCAAGGTTTTATTACTAATGTGACTGATTCTGCTGCTAATTTTAGTTATTTAGCAGACGGTGGGTTAGCTATT
TTAGATACTTCGGGTGCCATAGATGTCTTCGTTGTACAGGGCAGCTATGGTTTTAATTATTACAAGGTCAATCC

TTGTGAAGATGTTAACCAACAGTTTGTAGTGTCTGGTGGTAATATAGTTGGCATTCTTACTTCTAGAAATGAA
ACAGGTTCTGAACAGGTTGAGAACCAGTTTTATGTTAAGTTAACCAATAGCTCACATCGTCGTAGGCGTT
>CK_CH_GD_QY_TN-YDZSG_20210320.seq
ATGTTGGGGAAGTCACTGTTTCTAGTGATCATTTTGTGTGCACTATGTAGTGCAAATTTGTTTGATTCTGCCAA
TAATTATGTGTACTACTACCAAAGTGCCTTTAGGCCTCCAAATGGATGGCATTTGCAAGGGGGTGCTTATGCA
GTAGTGAATTCTACTAATTATACTAATAATGCCGGTTCTGCAAGTGAGTGCACTGTTGGTATTATTAAGGACGT
CTATAATCAAAGTGCGGCTTCCATAGCTATGACAGCACCTCCTGAGGGTATGGCTTGGTCTAAGTCACAATTT
TGTAGTGCACACTGTAACTTTTCTGAAATTACAGTTTTTGTCACACATTGTTATAGTAGTGGTTCAGGGTCTTG
CCCTATAACAGGCATGATTGCACGTGATCATATTCGTATTTCTGCAATGAAAAATGGTTCTTTATTTTATAACTT
AACAGTTAGCGTATCTAGATACTCTAAGTTTAAGTCTTTTCAATGTGTTAACAACCTCACATCTGTTTATTTAAA
TGGTGATCTTGTTTTTACTTCCAATAAAACTACTGATGTTACGTCAGCAGGTGTGTATTTTAAAGCAGGTGGA
CCTGTAAATTATAGTGTTATGAAAGAATTTAAGGTTCTTGCTTATTTTGTTAATGGTACAGCACAAGATGTAAT
TTTGTGTGACAAGTCCCCCAAGGGTTTGCTAGCTTGTCAATATAACACTGGCAATTTTTCAGATGGCTTTTAT
CCTTTTACTAATACTACTTTAGTTAGGGAAAAGTTCATCGTATATCGTGAAAGTAGTGTTAATACTACTCTGGC
GTTAACTAATTTCACTTTTACTAATGTAAGTAATGCACAGCCTAATAGTGGTGGTGTTAATACTTTTCATCTATAT
CAAACACAAACAGCTCAGAGTGGTTATTATAATTTTAATTTGTCATTTCTGAGTCAGTTTGTGTATAAGGCAA
GTGATTTTATGTATGGGTCTTATCATCCTAAGTGTTCTTTTAGACCTGAAACCATTAATAGTGGTTTATGGTTTA
ATTCCTTGTCAGTTTCTCTTACTTATGGACCCCTACAGGGAGGGTGTAAGCAATCTGTTTTTAGTGGTAAGGC
AACGTGTTGTTATGCCTACTCTTATAATGGCCCTAGGGCATGTAAAGGTGTTTATTCAGGTGAATTAAGCAAG
ACTTTTGAATGTGGATTGCTGGTTTATGTTACTAAGAGTGATGGCTCTCGTATACAAACTAGAACAGAGCCCT
TAGTATTAACGCAACACAATTATAATAATGTTACTTTAGATAAGTGTGTTGACTATAATATATATGGCAGAGTAG
GCCAAGGTTTTATTACTAATGTGACTGATTCTGCTGCTAATTTTAGTTATTTAGCAGATGGTGGGTTAGCTATT
TTAGATACTTCGGGTGCCATAGATGTCTTTGTTGCACAGGGCAGCTATGGTCTTAATTATTACAAGGTCAATCC
TTGTGAAGATGTTAACCAACAGTTTGTAGTGTCTGGTGGTAATATAGTTGGCATTCTTACTTCTAGAAATGAA
ACAGGTTCTGAACAGGTTGAGAACCAGTTTTATGTTAAGTTAACCAATAGCTCACATCGTCATAGGCGT
>CK_CH_GD_QY_YF-ZXX_20210223.seq
ATGTTGGGGAAGTCACTGTTTTTAGTGACCATTTTGTGTGCACTATGTAGTGCAAATTTGTTTGATTCTGCCA
ATAATTATGTGTACTACTACCAAAGTGCCTTTAGGCCTCCAAATGGATGGCATCTGCAAGGGGGTGCTTATGC
AGTAGTGAATTCTACTAATTATACTAGTAATGCCGGTGCTGCAAGTGAGTGCACTGTTGGTATTATTAAGGAC
GTCTATAATCAAAGTGTGGCTTCCATAGCTATGACAGCACCTCCTCAGGGTATGGCTTGGTCTAAGTCACAAT
TTTGTAGTGCACACTGTAACTTTTCTGAAATTACAGTCTTTGTCACACATTGTTATAGTAGTGGTGCAGGGTCT
TGCCCTATAACAGGCATGATTGCACGTGATCATATTCGTATTTCTGCAATGAAAAATGGTTCTTTATTTTATAAC
TTAACAGTTAGCGTATCTAAATACTCTAGGTTTAAGTCTTTTCAATGTGTTAATAACCTCACATCTGTTTATTTAA
ATGGTGATCTTGTTTTTACTTCCAATAAAACTACTGATGTTACGTCAGCAGGTGTGTATTTTAAAGCAGGTGG
ACCTGTAAATTATAGTGTTATGAAAGAATTTAAGGTTCTTGCTTACTTTGTTAATGGTACAGCACAAGACGTAA
TTTTGTGTGACAATTCCCCTAAGGGTTTGCTAGCCTGTCAATATAGTACTGGCAATTTTTCAGATGGCTTCTAT
CCTTTTACTAATAGTACTTTAGTTAGGGACAAGTTCATTGTCTATCGTGAAAGTAGTGTTAATACTACTTTGAC
GTTAAATAATTTCACTTTTACTAATGTAAGTACTGCACAGCCTAATAGTGGTGGTGTTAGTACTTTTCATCTATA
TCAAACACAAACAGCTCAGAGTGGTTATTATAATTTTAATTTGTCATTTCTGAGTCAGTTTGTGTATAAGGCAA
GTGATTTTATGTATGGGTCTTATCATCCTAGGTGTTCTTTTAGACCTGAAACCATTAATAGTGGTTTATGGTTTA
ATTCCTTGTCAGTTTCTCTTACTTATGGACCCCTACAGGGAGGGTGTAAGCAATCTGTTTTTAGTGGTAAGGC
AACGTGTTGTTATGCCTACTCTTATAATGGCCCTAGGGCATGTAAAGGTGTTTATTCAGGTGAATTAAGCAAG
ACTTTTGAATGTGGATTGCTGGTTTATGTTACTAAGAGTGATGGCTCTCGTATACAAACTAGAACAGAGCCCT
TAGTATTAACGCAACACAATTATAATAATGTTACTTTAGATAAGTGTGTTGACTATAATATATATGGCAGAGTAG

GCCAAGGTTTTATTACTAATGTGACTGATTCTGCTGCTAATTTTAGTTATTTAGCAGATGGTGGGTTAGCTATT
TTAGATACTTCGGGTGCCATAGATGTCTTTGTTGTACAGGGCAGCTATGGTTCTAATTATTACAAGGTCAATCC
TTGTGAAGATGTTAACCAACAGTTTGTAGTGTCTGGTGGTAATATAGTTGGCATTCTTACTTCTAGAAATGAA
ACAGGTTCTGAACAGGTTGAGAACCAGTTTTATGTTAAGTTAACCAATAGCTCACATCGTCATAGGCGT
>CK_CH_GD_QY_YF-ZSW_20210223.seq
ATGTTGGGGAAGTCACTGTTTTTAGTGACCATTTTGTGTGCACTATGTAGTGCAAATTTGTTTGATTCTGCCA
ATAATTATGTGTACTACTACCAAAGTGCCTTTAGGCCTCGAGATGGATGGCATCTGCAAGGGGGTGCTTATGC
AGTAGTGAATTCTACTAATTATACTAGTAATGCCGGTTCTGCAAGTGAGTGCACTGTTGGTATTATTAAGGACG
TCTATAATCAAAGTGTGGCTTCCATAGCTATGACAGCACCTCCTCAGGGTATGGCTTGGTCTAAGTCACAATTT
TGTAGTGCACACTGTAACTTTTCTGAAATTACAGTCTTTGTCACACATTGTTATAGTAGTGGTGCAGGGTCTT
GCCCTATAACAGGCATGATTGCACGTGGTCATATTCGTATTTCTGCAATGAAAAATGGTTCTTTATTTTATAACT
TAACAGTTAGCGTATCTAAATACTCTAGGTTTAAGTCTTTTCAATGTGTTAATAACCTCACATCTGTTTATTTAAA
TGGTGATCTTGTTTTTACTTCCAATAAAACTACTGATGTTACGTCAGCAGGTGTGTATTTTAAAGCAGGTGGA
CCTGTAAATTATAGTGTTATGAAAGAATTTAAGGTTCTTGCTTACTTTGTTAATGGTACAGCACAAGACGTAAT
TTTGTGTGACAATTCCCCTAAGGGTTTGCTAGCCTGTCAATATAGTACTGGCAATTTTTCAGATGGCTTCTATC
CTTTTACTAATAGTACTTTAGTTAGGGACAAGTTCATTGTCTATCGTGAAAGTAGTGTTAATACTACTTTGACG
TTAACTAATTTCACTTTTACTAATGTAAGTACTGCACAGCCTAATAGTGGTGGTGTTAGTACTTTTCATCTATAT
CAAACACAAACAGCTCAGAGTGGTTATTATAATTTTAATTTGTCATTTCTGAGTCAGTTTGTGTATAAGGCAA
GTGATTTTATGTATGGGTCTTATCATCCTAGGTGTTCTTTTAGACCTGAAACCATTAATAGTGGTTTATGGTTTA
ATTCCTTGTCAGTTTCTCTTACTTATGGACCCCTACAGGGAGGGTGTAAGCAATCTGTTTTTAGTGGTAAGGC
AACGTGTTGTTATGCCTACTCTTATAATGGCCCTAGGGCATGTAAAGGTGTTTATTCAGGTGAATTAAGCAAG
ACTTTTGAATGTGGATTGCTGGTTTATGTTACTAAGAGTGATGGCTCTCGTATACAAACTAGAACAGAGCCCT
TAGTATTAACGCAACACAATTATAATAATGTTACTTTAGATAAGTGTGTTGACTATAATATATATGGCAGAGTAG
GCCAAGGTTTTATTACTAATGTGACTGATTCTGCTGCTAATTTTAGTTATTTAGCAGATGGTGGGTTAGCTATT
TTAGATACTTCGGGTGCCATAGATGTCTTTGTTGTACAGGGCAGCTATGGTTCTAATTATTACAAGGTCAATCC
TTGTGAAGATGTTAACCAACAGTTTGTAGTGTCTGGTGGTAATATAGTTGGCATTCTTACTTCTAGAAATGAA
ACAGGTTCTGAACAGGTTGAGAACCAGTTTTATGTTAAGTTAACCAATAGCTCACATCGTCATAGGCGT
>CK_CH_SD_LY_DZC-12#1_20211115.seq
ATGTTGGGGAAGTCACTGTTTTTAGTGACCATTTTGTGTGCACTATGTAGTGCAAATTTGTTTGATTCTGCCA
ATAATTATGTGTACTACTACCAAAGTGCCTTTAGGCCTCCAAATGGATGGCATTTGCAAGGGGGTGCTTATGC
AGTAGTGAATTCTACTAATTATACTAGTAATGCCGGTTCTGCAAGTGGGTGCACTGTTGGTATTATTAAGGAC
GTCTATAATCAAAGTGCGGCTTCCATAGCTATGACAGCACCTCCTCAGGGTATGGCTTGGTCTAAGTCACAAT
TTTGTAGTGCACACTGTAACTTTTCTGAAATTACAGTCTTTGTCACACATTGTTATAGTAGTGGTGCAGGGTCT
TGCCCTATAACAGGCATGATTGCACGTGATCATATTCGTATTTCTGCAATGAAAAATGGTTCTTTATTTTATAAC
TTAACAGTTAGCGTATCTAAATACTCTAGGTTTAAGTCTTTTCAATGTGTTAATAACCTCACATCTGTCTATTTAA
ATGGTGATCTTGTTTTTACTTCCAATAAAACTACTGATGTTACGTCAGCAGGTGTGTATTTTAAAGCAGGTGG
ACCTGTAAATTATAGTGTTATGAAAGAATTTAAGGTTCTTGCTTACTTTGTTAATGGTACAGCACAAGACGTAA
TTTTGTGTGACAATTCCCCTAAGGGTTTGCTAGCCTGTCAATATAGTACTGGCAATTTTTCAGATGGCTTCTAT
CCTTTTACTAATAGTACTTTAGTTAGGGACAAGTTCATTGTCTATCGTGAAAGTAGTGTTAATACTACTTTGAC
GTTAACTAATTTCACTTTTACTAATGTAAGTACTGCACAGCCTAATAGTGGTGGTGTTAGTACTTTTCATCTATA
TCAAACACAAACAGCTCAGAGTGGTTATTATAATTTTAATTTGTCATTTCTGAGTCAGTTTGTGTATAAGGCAA
GTGATTTTATGTATGGGTCTTATCATCCTAGGTGTTCTTTTAGACCAGAAACCATTAATAGTGGTTTATGGTTTA
ATTCCTTGTCAGTTTCTCTTACTTATGGACCCCTACAGGGAGGGTGTAAGCAATCTGTTTTTAGTGGTAAGGC
AACGTGTTGTTATGCCTACTCTTATAATGGCCCTAGGGCATGTAAAGGTGTTTATTCAGGTGAATTAAGCAAG

ACTTTTGAATGTGGATTGCTGGTTTATGTTACTAAGAGTGATGGCTCTCGTATACAAACTAGAACAGAGCCCT
TAGTATTAACGCAACACAATTATAATAATGTTACTTTAGATAAGTGTGTTGACTATAATATATATGGCAGAGTAG
GCCAAGGTTTTATTACTAATGTGACTGATTCTGCTGCTAATTTTAGTTATTTAGCAGATGGTGGGTTAGCTATT
TTAGATACTTCGGGTGCCATAGATGTCTTTGTTGTACAGGGCAGCTATGGTTCTAATTATTACAAGGTCAATCC
TTGTGAAGATGTTAACCAACAGTTTGTAGTGTCTGGTGGTAATATAGTTGGCATTCTTACTTCTAGAAATGAA
ACAGGTTCTGAACAGGTTGAGAACCAGTTTTATGTTAAGTTAACCAATAGCTCACATCGTCATAGGCGT
>CK_CH_SD_LY_DZC-12#2_20211115.seq
ATGTTGGGGAAGTCACTGTTTTTAGTGACCATTTTGTGTGCACTATGTAGTGCAAATTTGTTTGATTCTGCCA
ATAATTATGTGTACTACTACCAAAGTGCCTTTAGGCCTCCAAATGGATGGCATTTGCAAGGGGGTGCTTATGC
AGTAGTGAATTCTACTAATTATACTAGTAATGCCGGTTCTGCAAGTGGGTGCACTGTTGGTATTATTAAGGAC
GTCTATAATCAAAGTGCGGCTTCCATAGCTATGACAGCACCTCCTCAGGGTATGGCTTGGTCTAAGTCACAAT
TTTGTAGTGCACACTGTAACTTTTCTGAAATTACAGTCTTTGTCACACATTGTTTTAGTAGTGGTGCAGGGTC
TTGCCCTATAACAGGCATGATTGCACGTGATCATATTCGTATTTCTGCAATGAAAAATGGTTCTTTATTTTATAA
CTTAACAGTTAGCGTATCTAAATACTCTAGGTTTAAGTCTTTTCAATGTGTTAATAACCTCACATCTGTCTATTTA
AATGGTGATCTTGTTTTTACTTCCAATAAAACTACTGATGTTACGTCAGCAGGTGTGTATTTTAAAGCAGGTG
GACCTGTAAATTATAGTGTTATGAAAGAATTTAAGGTTCTTGCTTACTTTGTTAATGGTACAGCACAAGACGT
AATTTTGTGTGACAATTCCCCTAAGGGTTTGCTAGCCTGTCAATATAGTACTGGCAATTTTTCAGATGGCTTCT
ATCCTTTTACTAATAGTATTTTAGTTAGGGACAAGTTCATTGTCTATCGTGAAAGTAGTGTTAATACTACTTTGA
CGTTAACTAATTTCACTTTTACTAATGTAAGTACTGCACAGCCTAATAGTGGTGGTGTTAGTACTTTTCATCTAT
ATCAAACACAAACAGCTCAGAGTGGTTATTATAATTTTAATTTGTCATTTCTGAGTCAGTTTGTGTATAAGGCA
AGTGATTTTATGTATGGGTCTTATCATCCTAGGTGTTCTTTTAGACCAGAAACCATTAATAGTGGTTTATGGTTT
AATTCCTTGTCAGTTTCTCTTACTTATGGACCCCTACAGGGAGGGTGTAAGCAATCTGTTTTTAGTGGTAAGG
CAACGTGTTGTTATGCCTACTCTTATAATGGCCCTAGGGCATGTAAAGGTGTTTATTCAGGTGAATTAAGCAA
GACTTTTGAATGTGGATTGCTGGTTTATGTTACTAAGAGTGATGGCTCTCGTATACAAACTAGAACAGAGCCC
TTAGTATTAACGCAACACAATTATAATAATGTTACTTTAGATAAGTGTGTTGACTATAATATATATGGCAGAGTA
GGCCAAGGTTTTATTACTAATGTGACTGATTCTGCTGCTAATTTTAGTTATTTAGCAGATGGTGGGTTAGCTAT
TTTAGATACTTCGGGTGCCATAGATGTCTTTGTTGTACAGGGCAGCTATGGTTCTAATTATTACAAGGTCAATC
CTTGTGAAGATGTTAACCAACAGTTTGTAGTGTCTGGTGGTAATATAGTTGGCATTCTTACTTCTAGAAATGA
AACAGGTTCTGAACAGGTTGAGAACCAGTTTTATGTTAAGTTAACCAATAGCTCACATCGTCATAGGCGT
>CK_CH_ZJ_TZ_LH-LKY_20211129.seq
ATGTTGGGGAAGTCACTGTTTTTAGTGACCATTTTGTGTGCACTATGTAGTGCAAATTTGTTTGATTTAGCCA
ATAATTATGTGTACTACTACCAAAGTGCCTTTAGGCCTTCAAATGGATGGCATTTGCAAGGGGGTGCTTATGC
AGTAGTGAATTCTACTAATTATACTAATAATGCCGGTTCTGCAAGTGAGTGCACTGTTGGTATTATTAAGGACG
TCTATAATCAAAGTGCGGCTTCCATAGCTATGACAGCACCTCCTCAGGGTATGGCTTGGTCTAAGTCACAATT
TTGTAGTGCACACTGTAACTTTTCTGAAATTACAGTCTTTGTTACACATTGTTATAGTAGTGGTGCAGGGTCTT
GCCCTATAACAGGCATGATTGCACGTGATCATATTCGTATTTCTGCAATGAAAAATGGTTCTTTATTTTATAACT
TAACAGTTAGCGTATCTAAATACTCTAGGTTTAAGTCTTTTCAATGTGTTAATAACCTCACATCTGTTTATTTAAA
TGGTGATCTTGTTTTTACTTCCAATAAAACTACTGATGTTACGTCAGCAGGTGTGTATTTTAAAGCAGGTGGA
CCTGTAAATTATAGTGTTATGAAAGAATTTAAGGTTCTTGCTTACTTTGTTAATGGTACAGCACAAGACGTAAT
TTTGTGTGACAATTCCCCTAAGGGTTTGCTAGCCTGTCAATATAGTACTGGCAATTTTTCAGATGGCTTCTATC
CTTTTACTAACAGTACTTTAGTTAGGGACAAGTTCATTGTCTATCGTGAAAGTAGTGTTAATACTACTTTGACG
TTAACTAATTTCACTTTTACTAATGTAAGTACTGCACAGCCTAATAGTGGTGGTGTTAGTACTTTTCATCTATAT
CAAACACAAACAGCTCAGAGTGGTTATTATAATTTTAATTTGTCATTTCTGAGTCAGTTTGTGTATAAGGCAA
GTGATTTTATGTATGGGTCTTATCATCCTAGGTGTTCTTTTAGACCAGAAACCATTAATAGTGGTTTATGGTTTA

ATTCCTTGTCAGTTTCTCTTACTTATGGACCCCTACAGGGAGGGTGTAAGCAATCTGTTTTTAGTGGTAAGGC
AACGTGTTGTTATGCCTACTCTTATAATGGCCCTACGGCATGTAAAGGTGTTTATTCAGGTGAATTAAGCAAG
ACTTTTGAATGTGGATTGCTGGTTTATGTTACTAAGAGTGATGGCTCTCGTATACAAACTAGAACAGAGCCCT
TAGTATTAACGCAACACAATTATAATAATGTTACTTTAGATAAGTGTGTTGACTATAATATATATGGCAGAGTAG
GCCAGGGTTTTATTACTAATGTGACTGATTCTGCTGCTAATTTTAGTTATTTAGCAGATGGTGGGTTAGCTATT
TTAGATACTTCGGGTGCCATAGATGTCTTTGTTGTACAGGGCAGCTATGGTTCTAATTATTACAAGGTCAATCC
TTGTGAAGATGTTAACCAACAGTTTGTAGTGTCTGGTGGTAATATAGTTGGCATTCTTACTTCTAGAAATGAA
ACAGGTTCTGAACAGGTTGAGAACCAGTTTTATGTTAAGTTAACCAATAGCTCACATCGTCATAGGCGT
>CK_CH_ZJ_TZ_LH-JL_20211222.seq
ATGTTGGGGAAGTCACTGTTTTTAGTGACCATTTTGTGTGCACTATGTAGTGCAAATTTGTTTGATTTTGCCA
ATAATTATGTGTACTACTACCAAAGTGCCTTTAGGCCTTCAAATGGATGGCATTTGCAAGGGGGTGCTTATGC
AGTAGTGAATTCTACTAATTATACTAGTAATGCCGGTTCTGCAAGTGAGTGCACTGTTGGTATTATTAAGGACG
TCTATAATCAAAGTGCGGCTTCCATAGCTATGACAGCACCTCCTCAGGGTATGGCTTGGTCTAAGTCACAATT
TTGTAGTGCACACTGTAACTTTTCTGAAATTACAGTCTTTGTCACACATTGTTATAGTAGTGGTGCAGGGTCTT
GCCCTATAACAGGCATGATTGCACGTGATCGTATTCGTATTTCTGCAATGAAAAATGGTTCTTTATTTTATAATT
TAACAGTTAGCGTATCTAAATACTCTAGGTTTAAGTCTTTTCAATGTGTTAATAACCTCACATCTGTTTATTTAAA
TGGTGATCTTGTTTTTACTTCCAATAAAACTACTGATGTTACGTCAGCAGGTGTGTATTTTAAAGCAGGTGGA
CCTGTAAATTATAGTGTTATGAAAGAATTTAGGGTTCTTGCTTACTTTGTTAATGGTACAGCACAAGACGTAAT
TTTGTGTGACAATTCCCCTAAGGGTTTGCTAGCCTGTCAATATAGTACTGGCAATTTTTCAGATGGCTTCTATC
CTTTTACTAATAGTACTCTAGTTAGGGACAAGTTTATTGTCTATCGTGAAAGTAGTGTTAATACTACTTTGACGT
TAACTAATTTCACTTTTACTAATGTAAGTACTGCACAGCCTAATAGTGGTGGTGTTAGTACTTTTCATCTATATC
AAACACAAACAGCTCAGAGTGGTTATTATAATTTTAATTTGTCATTTCTGAGTCAGTTTGTGTATAAGGCAAGT
GATTTTATGTATGGGTCTTATCATCCTAGGTGTTCTTTTAGACCAGAAACCATTAATAGTGGTTTATGGTTTAAT
TCCTTGTCAGTTTCTCTTACTTATGGACCCCTACAGGGAGGGTGTAAGCAATCTGTTTTTAGTGGTAAGGCAA
CGTGTTGTTATGCCTACTCTTATAATGGCCCTAGGGCATGTAAAGGTGTTTATTCAGGTGAATTAAGCAAGAC
TTTTGAATGTGGATTGCTGGTTTATGTTACTAAGAGTGATGGCTCTCGTATACAAACTAGAACAGAGCCCTTA
GTATTAACGCAACACAATTATAATAATGTTACTTTAGATAAGTGTGTTGACTATAATATATATGGCAGAGTAGGC
CAAGGTTTTATTACTAATGTGACTGATTCTGCTGCTAATTTTAATTATTTAGCAGATGGTGGGTTAGCTATTTTA
GATACTTCGGGTGCCATAGATGTCTTTGTTGTACAGGGCATCTATGGTTCTAATTATTACAAGGTCAATCCTTG
TGAAGATGTTAACCAACAGTTTGTAGTGTCTGGTGGTAATATAGTTGGCATTCTTACTTCTAGAAATGAAACA
GGTTCTGAACAGGTTGAGAACCAGTTTTATGTTAAGTTAACCAATAGCTCACATCGTCATAGGCGT
>CK_CH_YN_KM-I2_20211222.seq
ATGTTGGGGAAGTCACTGTTTTTAGTGACCTTTTTGTGTGCACTATGTAGTGCAAATTTGTTTGATCTTGCCA
CTAATTATGTGTACTACTACCAAAGTGCCTTTAGGCCTCCAACTGGATGGCATTTGCAAGGGGGTGCTTACGC
AGTAGTGAATTCTACTAATTATACTAATAATGCCGGTTCTGCAAGTGAGTGCACTGTTGGTATTATTAAGGACG
TCTATAATCAAAGTGCGGCTTCCATAGCTATGACAGCACCTCCTCAGGGTATGGCTTGGTCTAAGTCACAATT
CTGTAGTGCACACTGTAACTTTTCTGAAATTACAGTTTTTGTCACACATTGTTATAGTAGTGGTACAGGGTCTT
GCCCTATAACAGGCATGATTGCACGTGATCATATTCGTATTTCTGCAATGAAAAATGGTTCTTTATTTTATAACT
TAACAGTTAGCGTATCTAAATACTCTAGGTTTAAGTCTTTTCAATGTGTTAACAACTTCACATCTGTTTATCTAA
ATGGTGATCTTGTTTTTACTTCCAATAAAACTACTGATGTTACGTCAGCAGGTGTGTATTTTAAATCAGGTGGA
CCTGTAAATTATAGTGTTATGAAAGAATTTAAGGTTCTTGCTTATTTTGTTAATGGTACAGCACAAGATGTAAT
TTTGTGTGACAAGTCCCCCAAGGGTTTGCTAGCTTGTCAATATAACACTGGCAATTTTTCAGATGGCTTTTAT
CCTTTTACTAATACTACTTTAGTTAGGGAAAAGTTCATCGTATATCGTGAAAGTAGTGTTAATACTATTCTGACG
TTAACTAATTTCACTTTTACTAATGTAAGTAATGCACAGCCTAATAGTGGTGGTGTTAATACTTTTCATTTATATC

AAACACAAACAGCTCAGAGTGGTTATTATAATTTTAATTTCTCATTTCTGAGTCAGTTTGTGTATAAGGCAAGT
GATTTTATGTATGGGTCTTATCATCCTATGTGTTCTTTTAGACCAGAAACCATTAATAGTGGTTTGTGGTTTAAT
TCCTTGTCAGTTTCTCTTACTTATGGACCCCTACAGGGAGGGTGTAAGCAATCTGTTTTTAGTGGTAAGGCAA
CGTGTTGTTATGCCTACTCTTATAATGGCCCTAGAGTATGTAAAGGTGTTTATTCAGGTGAATTAAGCAAGACT
TTTGAATGTGGATTGCTGGTTTATGTTACTAAGAGTGATGGCTCTCGTATACAGACTAGAACAGAGCCCTTAG
TATTAACGCAACACAATTATAATAATATTACTTTAGATAAGTGTGTTGAATATAATATATATGGCAGAGTAGGCC
AAGGTTTTATTACTAATGTGACTGATTCTGCTGCTAATTCTAGTTATTTAGCAGATGGTGGGTTAGCTATTTTA
GATACTTCGGGTGCCATAGATGTCTTTGTTGTACAGAGCAGCTATGGTCTTAATTATTACAAGGTCAATCCTTG
TGAAGATGTTAATCAACAGTTTGTAGTGTCTGGTGGTAATATAGTTGGCATTCTTACTTCTAGAAATGAAACA
GGTTCTGAACAGGTTGAGAACCAGTTTTATGTTAAGTTAACCAATAGCTCACACCGTCGTAGGCGT
>CK_CH_YN_KM_LZY-574_20210907.seq
ATGTTGGGGAAGTCACTGTTTTTAGTGACCATTTTGTGTGCACTATGTAGTGCAAATTTGTTTGATCTTGCCA
ATAATTATGTGTACTACTACCAAAGTGCCTTTAGGCCTCCAACTGGATGGCATTTGCAAGGGGGTGCTTACGC
AGTAGTGAATTCTACTAATTATACTAGTAATGCCGGTTCTGCAAGTGAGTGCACTGTTGGTATTATTAAGGACG
TCTATAATCAAAGTGCGGCTTCCATAGCTATGACAGCACCTCCTCAGGGTATGGCTTGGTCTAAGTCACAATT
CTGTAGTGCACACTGTAACTTTTCTGAAATTACAGTTTTCGTCACACATTGTTATAGTAGTGGTACAGGGTCTT
GCCCTATAACGGGCATGATTGCACGTGATCATATTCGTATTTCTGCAATGAAAAATGGTTCTTTATTTTATAACT
TAACAGTTAGCGTATCTAAATACTCTAGGTTTAAATCTTTTCAATGTGTTAACAACTTCACATCTGTTTATTTAA
ATGGTGATCTTGTTTTTACTTCCAACAAAACTACTGATGTTACGTCAGCAGGTGTGTATTTTAAAGCAGGTGG
ACCTGTAAATTATAGTGTTATGAAAGAATTTAAGGTTCTTGCTTATTTTGTTAATGGTACAGCACAAGATGTAA
TTTTGTGTGACAAGTCCCCCAAGGGTTTGCTAGCTTGTCAATATAACACTGGCAATTTTTCAGATGGCTTTTAT
CCTTTTACTAATACTACTTTAGTTAGGGAAAAGTTCATCGTATATCGTGAAAGTAGTGTTAATACTACTCTGGC
GTTAATTAATTTTACTTTTACTAATGTAAGTAATGCACAGCCTAATAGTGGTGGTGTTAATGCTTTTCATTTATAT
CAAACACAAACAGCTCAGAGTGGTTATTATAATTTTAATTTGTCATTTCTGAGTCAGTTTGTGTATAAGGCAA
GTGATTTTATGTATGGGTCTTATCATCCTAAGTGTTCTTTTAGACCAGAAACCATTAATAGTGGTTTGTGGTTTA
ATTCCTTGTCAGTTTCTCTTACTTATGGACCCCTACAGGGAGGGTGTAAGCAATCTGTTTTTAGTGGTAAGGC
AACGTGTTGTTATGCCTACTCTTATAATGGCCCTACAGCATGTAAAGGTGTTTATTCAGGTGAATTAAGCAAG
ACTTTTGAATGTGGATTGCTGGTTTATGTTACTAAGAGTGATGGCTCTCGCATACAAACTAGAACGGAGCCCT
TAGTATTAACGCAACACAATTATAATAATATTACTTTAGATAAGTGTGTTAACTATAATATATATGGCAGAGTGG
GCCAAGGTTTTATTACTAATGTGACTGATTCTGCTGCTAATTTTAGTTATTTAGCAGATGGTGGGTTAGCTATT
TTAGACACTTCGGGTGCCATAGATGTTTTTGTTGCACAGGGCAGCTATGGTCCTAATTATTACAAGGTCAATC
CTTGTGAAGATGTTAACCAACAGTTTGTAGTGTCTGGTGGCAATATAGTTGGCATTCTTACTTCTAGAAATGA
AACAGGTTCTGAACAGGTTGAGAACCAGTTTTATGTTAAGTTAACCAATAGCTCACATCGTCGTAGGCGT
>CK_CH_GD_SG_LH-ZZX_20210222.seq
ATGTTGGGGAAGTCACTGTTTTTAGTGACCATTTTGTGTGCACTATGTAGTGCAAATTTGTTTGATTTTGCCA
ATAATTATGTGTACTACTACCAAAGTGCCTATAGGCCTCCAAATGGATGGCATTTGCAAGGGGGTGCTTATGC
AGTAGTGAATTCTACTAATTATACTAATAATGCCGATTCTGCAAGTGGGTGCACTGTTGGTATTATTAAGGACG
TCTATAATCAAAGTGCGGCTTCCATAGCTATGACAGCACCTTCTCAGGGTATGGCTTGGTCTAAGTCACAATT
TTGTAGTGCACACTGTAACTTTTCTGAAATTACAGTTTTCGTTACACATTGTTATAGTAGTGGTGCAGGGTCTT
GCCCTATAACAGGCATGATTGCACGTGATCATATTCGTATTTCTGCAATGAAAAATGGTTCTTTATTTTATAACT
TAACAGTTAGCGTATCTAAATACTCTAGGTTTAAGTCTTTTCAATGTGTTAACAACTTCACATCTGTTTATCTAA
ATGGTGATCTTGTTTTTACTTCCAACAAAACTACTGATGTTACGTCAGCAGGTGTGTATTTTAAAGCAGGTGG
ACCTGTAAATTATAGTGTTATGAAAGAATTTAAGGTTCTTGCTTATTTTGTTAATGGTACAGCACAAGATGTAA
TTTTGTGTGACAAGTCCCCCAAGGGTTTGCTAGCTTGTCAATATAACACTGGCAATTTTTCAGATGGCTTTTAT

CCTTTTACTAATACTACTTTAGTTAGGGAAAAGTTCATCGTATATCGTGAAAGTAGTGTTAATACTACTCTGGA
GTTAACTAATTTCACTTTTACTAATGTAAGTAATGCACAGCCTAATAGTGGTGATGTTAATACTTTTCATTTATAT
CAAACACAAACAGCTCAGAGTGGTTATTATAATTTTAATTTCTCATTTCTGAGTCAGTTTGTGTATAAGGCAAG
TGATTATATGTATGGGTCTTTTCATCCTAGGTGTTCTTTTAGACCAGAAACCATTAATAATGGTTTGTGGTTTAA
TTCCTTGTCAGTTTCTCTTACTTATGGACCCCTACAGGGAGGGTGTAAGCAATCTGTTTTTAGTGGTAAGGCA
ACGTGTTGTTATGCCTACTCTTATAATGGCCCTAGAGTATGTAAAGGTGTTTATTCAGGTGAATTAAGCAAGAC
TTTTGAATGTGGATTGCTGGTTTATGTTACTAAGAGTGATGGCTCTCGTATACAGACTAGAACAGAGCCCTTA
GTATTAACGCAACACAATTATAATAATATTACTTTAGATAAGTGTGTTGAATATAATATATATGGCAGAGTAGGC
CAAGGTTTTATTACTAATGTGACTGATTCTGCTGCTAATTCTAGTTATTTAGCAGATGGTGGGTTAGCTATTTTA
GATACTTCGGGTGCCATAGATGTCTTTGTTGTACAGGGCAGCTATGGTCTTAATTATTACAAGGTCAATCCTTG
TGAAGATGTTAATCAACAGTTTGTAGTGTCTGGTGGTAATATAGTTGGCATTCTTACTTCTAGAAATGAAACA
GGTTCTGAACAGGTTGAGAACCAGTTTTATGTTAAGTTAACCAATAGCTCACATCGTCGTAGGCGT
>CK_CH_SC_DY_DK-GJM_20210816.seq
ATGTTGGGGAAGTTACTGTTTTTAGTGACCATTTTGTGTGCACTATGTAGTGCAAAATTGTTTGATTCTGCCA
ATAATTATGTGTACTACTACCAAAGTGCCTTTAGGCCTCCAAATGGATGGCATTTGCAAGGGGGTGCTTATGC
AGTAGTGAATTCTACTAATTATACTAATAATGCCGGTTCTGCACCTGTGTGCACTGTTGGTATTATTAAGGACG
TCTATAATCAAAGTGCGGCTTCTATAGCTATGACAGCACCTCTTCAGGGTATGGCTTGGTCTAAGTCACAATTT
TGTAGTGCACACTGTAACTTTTCTGAAATTACAGTTTTTGTCACACATTGTTATAGTAGCGGTAGTGGGTCTTG
TCCTATAACAGGCGTGATTCCACAGGGTCATATTCGCATTTCTGCAATGAAAAATGGCTCTTTATTTTATAATTT
AACAGTTAGCGTGTCTAAATACCCTAATTTTAAATCTTTTCAATGTGTTAACAACTTCACATCTGTTTATCTAAA
TGGTGATCTTGTTTTTACTTCCAACAAAACTACTGATGTTACGTCAGCAGGTGTGTATTTTAAAGCAGGTGGA
CCTGTAAATTATAGTGTTATGAAAGAATTTAAGGTTCTTGCTTATTTTGTTAATGGTACAGCACAAGATGTAAT
TTTGTGTGACAAGTCCCCCAAGGGTTTGCTAGCTTGTCAATATAACACTGGCAATTTTTCAGATGGCTTTTAT
CCTTTTACTAATACTACTTTAGTTAGGGAAAAGTTCATCGTATATCGTGAAAGTAGTGTTAATACTACTCTGGC
GTTAACTAATTTCACTTTTACTAATGTAAGTGATGCACATCCTAATAGTGGTGATGTTAATACTTTTCATTTATAT
CAAACACAAACAGCTCAGAGTGGTTATTATAATTTTAATTTCTCATTTCTGAGTCAGTTTGTGTATAAGGCAAG
TGATTTTATGTATGGGTCTTATCATCCTAGGTGTTCTTTTAGACCAGAAACCATTAATAATGGTTTGTGGTTTAA
TTCCTTGTCAGTTTCTCTTACTTATGGACCCCTACAGGGAGGGTGTAAGCAATCTGTTTTTAGTGGTAAGGCA
ACGTGTTGTTATGCCTACTCTTATAATGGCCCTAGAGTATGTAAAGGTGTTTATTCAGGTGAATTAAGAGAGA
CTTTTGAATGTGGATTGCTGGTTTATGTTACTAAGAGTGATGGCTCTCGTATACAGACTAGAACAGAGCCCTT
AGTATTAACGCAACACAATTATAATAATATTACTTTAGATAAGTGTGTTGAATATAACATATATGGCAGAGTAGG
CCAAGGTTTTATTACTAATGTGACTGATTCTGCTGCTAATTTTAGTTATTTAGCAGATGGTGGGTTAGCTATTTT
AGATACTTCGGGTGCCATAGATGTCTTTGTTGTACAGGGCAGCTATGGTCGTAATTATTACAAGGTCAATCCTT
GTGAAGATGTTAATCAACAGTTTGTAGTGTCTGGTGGTAATATAGTTGGCATTCTTACTTCTAGAAATGAAAC
AGGTTCTGAACAGGTTGAGAACCAGTTTTATGTTAAGTTAACCAATAGCTCACATCGTCGTAGGCGT
>CK_CH_YN_KM-ZY_20210620.seq
ATGTTGGGGAAGTCACTGTTTTTAGTGACCATTTTGTGTGCACTATGTAGTGCAAATTTGTTTGGTTTTGCCA
ATAATTATGTGTACTACTACCAAAGTGCCTTTAGGCCTCCAAATGGATGGCATTTGCAAGGGGGTGCTTATGC
AGTAGTGAATTCTACTAATTATACTAGTAATGCCGGTTCTGCAAGTGAGTGCACTGTTGGTATTATTAAGGACG
TCTATAATCAAAGTGCGGCTTCCATAGCTATGACAGCACCCTCTCAGGGTATGGCTTGGTCTAAGTCACAATT
TTGTAGTGCACACTGTAACTTTTCTGAAATTACAGTTTTCGTCACACATTGTTATAGTAGTGGTACAGGGTCTT
GCCCTATAACAGGCATGATTGCACGTGATTATATTCGTATTTCTGCAATGAAAAATGGTTCTTTATTTTATAACT
TAACAGTTAGCGTATCTAAATACTCTAGGTTTAAGTCTTTTCAATGTGTTAACAACCTCACATCTGTTTATTTAA
ATGGTGATCTTGTTTTTACTTCCAACAAAACTACTGATGTTACGTCAGCAGGTGTGTATTTTAAAGCAGGTGG

ACCTGTAAATTATAGTGTTATGAAAGAATTTAAGGTTCTTGCTTATTTTATTAATGGTACAGTACAAGATGTAAT
TTTGTGTGACAAGTCCCCCAAGGGTTTGCTAGCTTGTCAATATAATACTGGCAATTTTTCAGATGGCTTTTATC
CTTTTACTAATACTACTTTAGTTAGGGAAAAGTTCATCGTATATCGTGAAAGTAGTGTTAATACTACTCTGGCG
TTAACTAATTTAATTTTTACTAATGTAAGTAATGCACAGCCTAATAGTGGTGGTGTTAATACTTTTCATTTATATC
AAACACAAACAGCTCAGAGTGGTTATTATAATTTTAATTTGTCATTTCTGAGTCAGTTTGTGTATAAGGCAAGT
GATTTTATGTATGGGTCTTATCATCCTAGGTGTTCTTTTAGACCAGAAACCATTAATAATGATTTGTGGTTTAAT
TCCTTGTCAGTTTCTCTTACTTATGGACCCCTACAGGGAGGGTGTAAGCAATCTGTTTTTAGTGGTAAGGCAA
CGTGTTGTTATGCCTACTCTTATAATGGCCCTAGAGTATGCAAAGGTGTTTATTCAGGTGAATTAAGCAAGAC
TTTTGAATGTGGATTGCTGGTTTATGTTACTAAGAGTGATGGCTCTCGTATACAAACTAGAACGGAGCCCTTA
GTATTAACGCAACACAATTATAATAATATTACTTTAGATAAGTGTGTTAACTATAATATATATGGCAGAGTGGGC
CAAGGTTTTATTACTAATGTGACTGATTCTGCTGCTAATTTTAGTTATTTAGCAGATGGTGGGTTAGCTATTTTA
GACACTTCGGGTGCCATAGATGTTTTTGTTGCACAGGGCAGCTATGGTCCTAATTATTACAAGGTCAATCCTT
GTGAAGATGTTAACCAACAGTTTGTAGTGTCTGGTGGCAATATAGTTGGCATTCTTACTTCTAGAAATGAAAC
AGGTTCTGAACAGGTTGAGAACCAGTTTTATGTTAAGTTAACCAATAGCTCACATCGTCGTAGGCGT
>CK_CH_AH_FY-LH_MSZ_20211122.seq
ATGTTGGGGAAGTCACTGTTTTTAGTGACCATTTTGTGTGCACTATGTAGTGCAAATTTGTTTGATTTGGCCA
ATAATTATGTGTACTACTACCAAAGTGCCTTTAGGCCTCCAAATGGATGGCATTTGCAAGGGGGTGCTTATGC
AGTAGTGAATTCTACTAATTATACTAGTAATGCCGGTTCTGCAAGTGAGTGCACTGTTGGTATTATTAAGGACG
TCTATAATCAAAGTGCGGCTTCCATAGCTATGACAGCACCTTCTCAGGGTATGGCTTGGTCTAAGTCACAATT
TTGTAGTGCACACTGTAACTTTTCTGAAATTACAGTTTTCGTCACACATTGTTATAGTAGTGGTACAGGGTCTT
GCCCTATAACAGGCATGATTGCACGTGATCATATTCGTATTTCTGCAATGAAAAATGGTTCTTTATTTTATAACT
TAACAGTTAGCGTATCTAAATACTCTAGGTTTAAGTCTTTTCAATGTGTTAACAACCTCACATCTGTTTATCTAA
ATGGTGATCTTGTTTTTACTTCCAACAAAACTACTGATGTTACGTCAGCAGGTGTGTATTTTAAAGCAGGTGG
ACCTGTAAATTATAGTGTTATGAAAGAATTTAAGGTTCTTGCTTATTTTGTTAATGGTACAGCACAAGATGTAA
TTTTGTGTGACAAGTCCCCCAAGGGTTTGCTAGCTTGTCAATATAACACTGGCAATTTTTCAGATGGCTTTTAT
CCTTTTACTAATACTACTTTAGTTAGGGAAAAGTTCATTGTATATCGTGAAAGTAGTGTTAATACTACTCTGGC
GTTAACTAATTTCACTTTTACTAATGTAAGTAATGCACAGCCTAATAGTGGTGGTGTTAATACTTTTCATTTATAT
CAAACACAAACAGCTCAGAGTGGTTATTATAATTTTAATTTGTCATTTCTGAGTCAGTTTGTGTATAAGGCAA
GTGATTTTATGTATGGGTCTTATTATCCTAGGTGTTCTTTTAGACCAGAAACCATTAATAATGGTTTGTGGTTTA
ATTCCTTGTCAGTTTCTCTTACTTATGGACCCCTACAGGGAGGGTGTAAGCAATCTGTTTTTAGTGGTAAGGC
AACGTGTTGTTATGCCTACTCTTATAATGGCCCTAGAGCATGCAAAGGTGTTTATTCAGGCGAATTAAGCAAG
ACTTTTGAATGTGGATTGCTGGTTTATGTTACTAAGAGTGATGGCTCTCGTATACAAACTAGAACGGAGCCCT
TAGTATTAACGCAACACAATTATAATAATATTACTTTAGATAAGTGTGTTAACTATAATATATATGGCAGAGTTGG
CCAAGGTTTTATTACTAATGTGACTGATTCTGCTGCTAATTTTAGTTATTTAGCAGATGGTGGGTTAGCTATTTT
AGATACTTCGGGTGCCATAGATGTTTTTGTTGTACAGGGCAGCTATGGTCCTAATTATTACAAGGTCAATCCTT
GTGAAGATGTTAACCAACAGTTTGTAGTGTCTGGTGGCAATATAGTTGGCCTTCTTACTTCTAGAAATGAAAC
AGGTTCTGAACAGGTTGAGAACCAGTTTTATGTTAAGTTAACCAATAGCTCACATCGTCGTAGGCGT
>CK_CH_GS_SY-4#_20210104.seq
ATGTTGGGGAAGTCACTGTTTTTAGTGACCATTTTGTGTGCACTATGTAGTGCAAATTTGTTTGATTTTGCCA
ATAATTATGTGTACTACTACCAAAGTGCCTTTAGGCCTCCAAAAGGATGGCATTTGCAAGGGGGTGCTTATGC
AGTAGTGAATTCTACTAATTATACTAATAATGCCGGTTCTGCAAGTGAGTGCACTGTTGGTATTATTAAGGACG
TCTATAATCAAAGTGCGGCTTCCATAGCTATGACAGCACCTTCTCAGGGTATGGCTTGGTCTAAGTCACAATT
TTGTAGTGCACACTGTAACTTTTCTGAAATTACAGTTTTCGTTACACATTGTTATAGTAGTGGTACAGGGTCTT
GCCCTATAACAGGCATGATTGCACGTGATCATATTCGTATTTCTGCAATGAAAAATGGTTCTTTATTTTATAACT

TAACAGTTAGCGTATCTAAATACTCTAGGTTTAAGTCTTTTCAATGTGTTAACAACCTCACATCTGTTTATCTAA
ATGGTGATCTTGTTTTTACTTCCAACAAAACTACTGATGTTACGTCAGCAGGTGTGTATTTTAAAGCAGGTGG
ACCTGTAAATTATAGTGTTATGAAAGAATTTAAGGTTCTTGCTTATTTTGTTAATGGTACAGCACAAGATGTAA
TTTTGTGTGACAAGTCCCCCAAGGGTTTGCTAGCTTGTCAATATAACACTGGCAATTTTTCAGATGGCTTTTAT
CCCTTTACTAATACTACTTTAGTTAGGGAAAAGTTCATTGTATATCGTGAAAGTAGTGTTAATACTACTCTGGC
GTTAACTAATTTCACTTTTACTAATGTAAGTAATGCACAGCCTAATAGTGGTGGTGTTAATACTTTTCATTTATAT
CAAACACAAACAGCTCAGAGTGGTTATTATAATTTTAATTTGTCATTTCTGAGTCAGTTTGTGTATAAGGCAA
GTGATTTTATGTATGGGTCTTATTATCCTAGGTGTTCTTTTAGACCAGAAACCATTAATAATGGTTTGTGGTTTA
ATTCCTTGTCAGTTTCACTTACTTATGGACCCCTACAGGGAGGGTGTAAGCAATCTGTTTTTAGTGGTAAGGC
AACGTGTTGTTATGCCTACTCTTATAATGGCCCTAGAGCATGCAAAGGTGTTTATTCAGGCGAATTAAGCAAG
ACTTTTGAATGTGGATTGCTGGTTTATGTTACTAAGAGTGATGGCTCTCGTATACAAACTAGAACGGAGCCCT
TAGTATTAACGCAACACAATTATAATAATATTACTTTAGATAAGTGTGTTAACTATAATATATATGGCAGAGTGG
GCCAAGGTTTTATTACTAATGTGACTGATTCTGCTGCTAATTTTAGTTATTTAGCAGATGGTGGGTTAGCTATT
TTAGACACTTCGGGTGCCATAGATGTTTTTGTTGCACAGGGCAGCTATGGTCCTAATTATTACAAGGTCAATC
CTTGTGAAGATGTTAATCAACAGTTTGTAGTGTCTGGTGGCAATATAGTTGGCATTCTTACTTCTAGAAATGA
AACAGGTTCTGAACAGGTTGAGAACCAGTTTTATGTTAAGTTAACCAATAGCTCACATCGTCGTAGGCGT
>CK_CH_GS_SY-3#_20210104.seq
ATGTTGGGGAAGTCACTGTTTTTAGTGACCATTTTGTGTGCACTATGTAGTGCAAATTTGTTTGATTTTGCCA
ATAATTATGTGTACTACTACCAAAGTGCCTTTAGGCCTCCAAATGGATGGCATTTGCAAGGGGGTGCTTATGC
AGTAGTGAATTCTACTAATTATACTAATAATGCCGGTTCTGCAAGTGAGTGCACTGTTGGTATTATTAAGGACG
TCTATAATCAAAGTGCGGCTTCCATAGCTATGACAGCACCTTCTCAGGGTATGGCTTGGTCTAAGTCACAATT
TTGTAGTGCACACTGTAACTTTTCTGAAATTACAGTTTTCGTTACACATTGTTATAGTAGTGGTACAGGGTCTT
GCCCTATAACAGGCATGATTGCACGTGATCATATTCGTATTTCTGCAATGAAAAATGGTTCTTTATTTTATAACT
TAACAGTTAGCGTATCTAAATACTCTAGGTTTAAGTCTTTTCAATGTGTTAACAACCTCACATCTGTTTATCTAA
ATGGTGATCTTGTTTTTACTTCCAACAAAACTACTGATGTTACGTCAGCAGGTGTGTATTTTAAAGCAGGTGG
ACCTGTAAATTATAGTGTTATGAAAGAATTTAAGGTTCTTGCTTATTTTGTTAATGGTACAGCACAAGATGTAA
TTTTGTGTGACAAGTCCCCCAAGGGTTTGCTAGCTTGTCAATATAACACTGGCAATTTTTCAGATGGCTTTTAT
CCCTTTACTAATACTACTTTAGTTAGGGAAAAGTTCATTGTATATCGTGAAAGTAGTGTTAATACTACTCTGGC
GTTAACTAATTTCACTTTTACTAATGTAAGTAATGCACAGCCTAATAGTGGTGGTGTTAATACTTTTCATTTATAT
CAAACACAAACAGCTCAGAGTGGTTATTATAATTTTAATTTGTCATTTCTGAGTCAGTTTGTGTATAAGGCAA
GTGATTTTATGTATGGGTCTTATTATCCTAGGTGTTCTTTTAGACCAGAAACCATTAATAATGGTTTGTGGTTTA
ATTCCTTGTCAGTTTCACTTACTTATGGACCCCTACAGGGAGGGTGTAAGCAATCTGTTTTTAGTGGTAAGGC
AACGTGTTGTTATGCCTACTCTTATAATGGCCCTAGAGCATGCAAAGGTGTTTATTCAGGCGAATTAAGCAAG
ACTTTTGAATGTGGATTGCTGGTTTATGTTACTAAGAGTGATGGCTCTCGTATACAAACTAGAACGGAGCCCT
TAGTATTAACGCAACACAATTATAATAATATTACTTTAGATAAGTGTGTTAACTATAATATATATGGCAGAGTGG
GCCAAGGTTTTATTACTAATGTGACTGATTCTGCTGCTAATTTTAGTTATTTAGCAGATGGTGGGTTAGCTATT
TTAGACACTTCGGGTGCCATAGATGTTTTTGTTGCACAGGGCAGCTATGGTCCTAATTATTACAAGGTCAATC
CTTGTGAAGATGTTAATCAACAGTTTGTAGTGTCTGGTGGCAATATAGTTGGCATTCTTACTTCTAGAAATGA
AACAGGTTCTGAACAGGTTGAGAACCAGTTTTATGTTAAGTTAACCAATAGCTCACATCGTCGTAGGCGT
>CK_CH_GD_QY_SHHF-2_20211221.seq
ATGTTGGGGAAGTCACTGTTTTTAGTGACCATTTTGTGTGCACTATGTAGTGCAAATTTGTTTGATTTGGCCA
ATAATTATGTGTACTACTACCAAAGTGCCTTTAGGCCTCCAAATGGATGGCATTTGCAAGGGGGTGCTTATGC
AGTAGTGAATTCTACTAATTATACTAGTAATGCCGGTTCTGCAAGTGAGTGCACTGTTGGTATTATTAAGGACG
TCTATAATCAAAGTGCGGCTTCCATAGCTATGACAGCACCTTCTCAGGGTATGGCTTGGTCCAAGTCACAATT

TTGTAGTGCACACTGTAACTTTTCTGAAATTACAGTTTTCGTCACACATTGTTATAGTAGTGGTACAGGGTCTT
GCCCTATAACAGGCATGATTGCACGTGATCATATTCGTATTTCTGCAATGAAAAATGGTTCTTTATTTTATAACT
TAACAGTTAGCGTATCTAAATACTCTAGGTTTAAGTCTTTTCAATGTGTTAACAACCTCACATCTGTTTATCTAA
ATGGTGATCTTGTTTTTACTTCCAACAAAACTACTGATGTTACGTCAGCAGGTGTGTATTTTAAAGCAGGTGG
ACCTGTAAATTATAGTGTTATGAAAGAATTTAAGGTTCTTGCTTATTTTGTTAATGGTACAGCACAAGATGTAA
TTTTGTGTGACAAGTCCCCCAAGGGTTTGCTAGCTTGTCAATATAACACTGGCAATTTTTCAGATGGCTTTTAT
CCTTTTACTAATACTACTTTAGTTAGGGAAAAGTTCATTGTATATCGTGAAAGTAGTGTTAATACTACTCTGGC
GTTAACTAATTTCACTTTTACTAATGTAAGTAATGCACAGCCTAATAGTGGTGGTGTTAATACTTTTCATTTATAT
CAAACACAAACAGCTCAGAGTGGTTATTATAATTTTAATTTCTCATTTCTGAGTCAGTTTGTGTATAAGGCAAG
TGATTTTATGTATGGGTCTTATCATCCTAGGTGTTCTTTTAGACCAGAAACCATTAATAATGATTTGTGGTTTAA
TTCCTTGTCAGTTTCTCTTACTTATGGACCCCTACAGGGAGGGTGTAAGCAATCTGTTTTTAGTGGTAAGGCA
ACGTGTTGTTATGCCTACTCTTATAATGGCCCTAGAGTATGTAAAGGTGTTTATTCAGGTGAATTAAGCAAGAC
TTTTGAATGTGGATTGCTGGTTTATGTTACTAAGAGTGATGGCTCTCGCATACAGACTAGAACAGAGCCCTTA
GTATTAACGCAACACAATTATAATAATATTACTTTAGATAAGTGTGTTAACTATAATATATATGGCAGAGTGGGC
CAAGGTTTTATTACTAATGTGACTGATTCTGCTGCTAATTTTAGTTATTTAGCAGATGGTGGGTTAGCTATTTTA
GACACTTCGGGTGCCATAGATGTTTTTGTTGCACAGGGCAGCTATGGTCCTAATTATTACAAGGTCAATCCTT
GTGAAGATGTTAACCAACAGTTTGTAGTGTCTGGTGGCAGTATAGTTGGCATTCTTACTTCTAGAAATGAAAC
AGGTTCTGAACAGGTTGAGAACCAGTTTTATGTTAAGTTAACCAATAGCTCACATCGTCGTAGGCGC
>CK_CH_SD_WF_YCH-3_20211116.seq
ATGTTGGGGAAGTCACTGTTTTTAGTGACCATTTTGTGTGCACTATGTAGTGCAAATTTGTTTGATTTTGCCA
ATAATTATGTGTACTACTACCAAAGTGCCTTTAGGCCTCCAAATGGATGGCATTTGCAAGGGGGTGCTTATGC
AGTAGTGAATTCTACTAATTATACTAGTAATGCCGGTTCTGGTGAGTGCACTGTTGGTATTATTAAGGACGTCT
ATAATCAAAGTGCGGCTTCCATAGCTATGACAGCACCCTCTCAGGGTATGGCTTGGTCTAAGTCACAATTTTG
TAGTGCACACTGTAACTTTTCTGAAATTACAGTTTTCGTCACACATTGTTATAGTAGTGGTACAGGGTCTTGCC
CTATAACAGGCATGATTGCACGTGATCATATTCGTATTTCTGCAATGAAAAATGGTTCTTTATTTTATAACTTAA
CAGTTAGCGTATCTAAATACTCTAGGTTTAAGTCTTTTCAATGTGTTAACAACTTCACATCTGTCTATCTAAATG
GTGATCTTGTTTTTACTTCCAACAAAACTACTGATGTTACGTCAGCAGGTGTGTATTTTAAAGCAGGTGGACC
TGTAAATTATAGTGTTATGAAAGAATTTAAGGTTCTTGCTTATTTTGTTAATGGTACAGCACAAGATGTAATTTT
GTGTGACAAGTCCCCCAAGGGTTTGCTAGCTTGTCAATATAACACTGGCAATTTTTCAGATGGCTTTTATCCT
TTTACTAATACTACTTTAGTTAGGGAAAAGTTCATCGTATATCGTGAAAGTAGTGTTAATACTACTCTGGCGTTA
ACTAATTTCACTTTTACTAATGTAAGTAATGCACAGCCTAATAGTGGTGGTGTTAATACTTTTCATTTATATCAA
ACACAAACAGCTCAGAGTGGTTATTATAATTTTAATTTCTCATTTCTGAGTCAGTTTGTGTATAAGGCAAGTGA
TTTTATGTATGGGTCTTATCATCCTAGGTGTTCTTTTAGACCAGAAACCATTAATAATGATTTGTGGTTTAATTC
CTTGTCAGTTTCTCTTACTTATGGACCCCTACAGGGAGGGTGTAAGCAATCTGTTTTTAGTGGTAAGGCAAC
GTGTTGTTATGCCTACTCTTATAATGGCCCTAGAGTATGTAAAGGTGTTTATTCAGGTGAATTAAGCAAGACTT
TTGAATGTGGATTGCTGGTTTATGTTACTAAGAGTGATGGCTCTCGTATACAGACTAGAACAGAGCCCTTAGT
ATTAACGCAACACAATTATAATAATATTACTTTAGATAAGTGTGTTAACTATAATATATATGGCAGAGTGGGCCA
AGGTTTTATTACTAATGTGACTGATTCTGCTGCTAATTTTAGTTATTTAGCAGATGGTGGGTTAGCTATTTTAGA
CACTTCGGGTGCCATAGATGTTTTTGTTGTACAGGGCAGCTATGGTCCTAATTATTACAAGGTCAATCCTTGT
GAAGATGTTAACCAACAGTTTGTAGTGTCTGGTGGCAATATAGTTGGCATTCTTACTTCTAGAAATGAAACAG
GTTCTGAACAGGTTGAGAACCAGTTTTATGTTAAGTTAACCAATAGCTCACATCGTCGTAGGCGC
>CK_CH_SD_WF_YCH-1_20211116.seq
ATGTTGGGGAAGTCACTGTTTTTAGTGACCATTTTGTGTGCACTATGTAGTGCAAATTTGTTTGATTTTGCCA
ATAATTATGTGTACTACTACCAAAGTGCCTTTAGGCCTCCAAATGGATGGCATTTGCAAGGGGGTGCTTATGC

AGTAGTGAATTCTACTAATTATACTAGTAATGCCGGTTCTGGTGAGTGCACTGTTGGTATTATTAAGGACGTCT
ATAATCAAAGTGCGGCTTCCATAGCTATGACAGCACCCTCTCAGGGTATGGCTTGGTCTAAGTCACAATTTTG
TAGTGCACACTGTAACTTTTCTGAAATTACAGTTTTCGTCACACATTGTTATAGTAGTGGTACAGGGTCTTGCC
CTATAACAGGCATGATTGCACGTGATCATATTCGTATTTCTGCAATGAAAAATGGTTCTTTATTTTATAACTTAA
CAGTTAGCGTATCTAAATACTCTAGGTTTAAGTCTTTTCAATGTGTTAACAACTTCACATCTGTCTATCTAAATG
GTGATCTTGTTTTTACTTCCAACAAAACTACTGATGTTACGTCAGCAGGTGTGTATTTTAAAGCAGGTGGACC
TGTAAATTATAGTGTTATGAAAGAATTTAAGGTTCTTGCTTATTTTGTTAATGGTACAGCACAAGATGTAATTTT
GTGTGACAAGTCCCCCAAGGGTTTGCTAGCTTGTCAATATAACACTGGCAATTTTTCAGATGGCTTTTATCCT
TTTACTAATACTACTTTAGTTAGGGAAAAGTTCATCGTATATCGTGAAAGTAGTGTTAATACTACTCTGGCGTTA
ACTAATTTCACTTTTACTAATGTAAGTAATGCACAGCCTAATAGTGGTGGTGTTAATACTTTTCATTTATATCAA
ACACAAACAGCTCAGAGTGGTTATTATAATTTTAATTTCTCATTTCTGAGTCAGTTTGTGTATAAGGCAAGTGA
TTTTATGTATGGGTCTTATCATCCTAGGTGTTCTTTTAGACCAGAAACCATTAATAATGATTTGTGGTTTAATTC
CTTGTCAGTTTCTCTTACTTATGGACCCCTACAGGGAGGGTGTAAGCAATCTGTTTTTAGTGGTAAGGCAAC
GTGTTGTTATGCCTACTCTTATAATGGCCCTAGAGTATGTAAAGGTGTTTATTCAGGTGAATTAAGCAAGACTT
TTGAATGTGGATTGCTGGTTTATGTTACTAAGAGTGATGGCTCTCGTATACAGACTAGAACAGAGCCCTTAGT
ATTAACGCAACACAATTATAATAATATTACTTTAGATAAGTGTGTTAACTATAATATATATGGCAGAGTGGGCCA
AGGTTTTATTACTAATGTGACTGATTCTGCTGCTAATTTTATTTATTTAGCAGATGGTGGGTTAGCTATTTTAGA
CACTTCGGGTGCCATAGATGTTTTTGTTGTACAGGGCAGCTATGGTCCTAATTATTACAAGGTCAATCCTTGT
GAAGATGTTAACCAACAGTTTGTAGTGTCTGGTGGCAATATAGTTGGCATTCTTACTTCTAGAAATGAAACAG
GTTCTGAACAGGTTGAGAACCAGTTTTATGTTAAGTTAACCAATAGCTCACATCGTCGTAGGCGC
>CK_CH_SD_FX_YL_13#_20210714.seq
ATGTTGGGGAAGTCACTGTTTTTAGTGACCATTTTGTGTGCACTATGTAGTGCAAATTTGTTTGATTTTGCCA
ATAATTATGTGTACTACTACCAAAGTGCCCTTAGGCCTCCAAATGGATGGCATTTGCAAGGGGGTGCTTATGC
AGTAGTGAATTCTACTAATTATACTAGTAATGCCGGTTCTGCAAGTGAGTGCACTGTTGGTATTATTAAGGACG
TCTATAATCAAAGTGCGGCTTCCATAGCTATGACAGCACCCTCTCAGGGTATGGCTTGGTCTAAGTCACAATT
TTGTAGTGCACACTGTAACTTTTCTGAAATTACAGTTTTCGTCACACATTGTTATAGTAGTGGTACAGGGTCTT
GCCCTATAACAGGCATGATTGCACGTGATCATATTCGTATTTCTGCAATGAAAAATGGTTCTTTATTTTATAACT
TAACAGTTAGCGTATCTAAATACTCTAGGTTTAAGTCTTTTCAATGTGTTAACAACTTCACATCTGTCTATCTAA
ATGGTGATCTTGTTTTTACTTCCAACAAAACTACTGATGTTACGTCAGCAGGTGTGTATTTTAAAGCAGGTGG
ACCTGTAAATTATAGTGTTATGAAAGAATTTAAGGTTCTTGCTTATTTTGTTAATGGTACAGCACAAGATGTAA
TTTTGTGTGACAAGTCCCCCAAGGGTTTGCTAGCTTGTCAATATAACACTGGCAATTTTTCAGATGGCTTTTAT
CCTTTTACTAATACTACTTTAGTTAGGGAAAAGTTCATCGTATATCGTGAAAGTAGTGTTAATACTACTCTGGC
GTTAACTAATTTCACTTTTACTAATGTAAGTAATGCACAGCCTAATAGTGGTGGTGTTAATACTTTTCATTTATAT
CAAACACAAACAGCTCAGAGTGGTTATTATAATTTTAATTTCTCATTTCTGAGTCAGTTTGTGTATAAGGCAAG
TGATTTTATGTATGGGTCTTATCATCCTAGGTGTTCTTTTAGACCAGAAACCATTAATAATGATTTGTGGTTTAA
TTCTTTGTCAGTTTCTCTTACTTATGGACCCCTACAGGGAGGGTGTAAGCAATCTGTTTTTAGTGGTAAGGCA
ACGTGTTGTTATGCCTACTCTTATAATGGCCCTAGAGTATGTAAAGGTGTTTATTCAGGTGAATTAAGCAAGAC
TTTTGAATGTGGATTGCTGGTTTATGTTACTAAGAGTGATGGCTCTCGTATACAGACTAGAACAGAGCCCTTA
GTATTAACGCAACACAATTATAATAATATTACTTTAGATAAGTGTGTTAACTATAATATATATGGCAGAGTGGGC
CAAGGTTTTATTACTAATGTGACTGATTCTGCTGCTAATTCTAGTTATTTAGCAGATGGTGGGTTAGCTATTTTA
GACACTTCGGGTGCCATAGATGTTTTTGTTGTACAGGGCAGCTATGGTCTTAATTATTACAAGGTCAATCCTT
GTGAAGATGTTAACCAACAGTTTGTAGTGTCTGGTGGCAATATAGTTGGCATTCTTACTTCTAGAAATGAAAC
AGGTTCTGAACAGGTTGAGAACCAGTTTTATGTTAAGTTAACCAATAGCTCACATCGTCGTAGGCGC
>CK_CH_SD_FX_SGM_2#_20210714.seq

ATGTTGGGGAAGTCACTGTTTTTAGTGACCATTTTGTGTGCACTATGTAGTGCAAATTTGTTTGATTTTGCCA
ATAATTATGTGTACTACTACCAAAGTGCCCTTAGGCCTCCAAATGGATGGCATTTGCAAGGGGGTGCTTATGC
AGTAGTGAATTCTACTAATTATACTAGTAATGCCGGTTCTGCAAGTGAGTGCACTGTTGGTATTATTAAGGACG
TCTATAATCAAAGTGCGGCTTCCATAGCTATGACAGCACCCTCTCAGGGTATGGCTTGGTCTAAGTCACAATT
TTGTAGTGCACACTGTAACTTTTCTGAAATTACAGTTTTCGTCACACATTGTTATAGTAGTGGTACAGGGTCTT
GCCCTATAACAGGCATGATTGCACGTGATCATATTCGTATTTCTGCAATGAAAAATGGTTCTTTATTTTATAACT
TAACAGTTAGCGTATCTAAATACTCTAGGTTTAAGTCTTTTCAATGTGTTAACAACTTCACATCTGTCTATCTAA
ATGGTGATCTTGTTTTTACTTCCAACAAAACTACTGATGTTACGTCAGCAGGTGTGTATTTTAAAGCAGGTGG
ACCTGTAAATTATAGTGTTATGAAAGAATTTAAGGTTCTTGCTTATTTTGTTAATGGTACAGCACAAGATGTAA
TTTTGTGTGACAAGTCCCCCAAGGGTTTGCTAGCTTGTCAATATAACACTGGCAATTTTTCAGATGGCTTTTAT
CCTTTTACTAATACTACTTTAGTTAGGGAAAAGTTCATCGTATATCGTGAAAGTAGTGTTAATACTACTCTGGC
GTTAACTAATTTCACTTTTACTAATGTAAGTAATGCACAGCCTAATAGTGGTGGTGTTAATACTTTTCATTTATAT
CAAACACAAACAGCTCAGAGTGGTTATTATAATTTTAATTTCTCATTTCTGAGTCAGTTTGTGTATAAGGCAAG
TGATTTTATGTATGGGTCTTATCATCCTAGGTGTTCTTTTAGACCAGAAACCATTAATAATGATTTGTGGTTTAA
TTCTTTGTCAGTTTCTCTTACTTATGGACCCCTACAGGGAGGGTGTAAGCAATCTGTTTTTAGTGGTAAGGCA
ACGTGTTGTTATGCCTACTCTTATAATGGCCCTAGAGTATGTAAAGGTGTTTATTCAGGTGAATTAAGCAAGAC
TTTTGAATGTGGATTGCTGGTTTATGTTACTAAGAGTGATGGCTCTCGTATACAGACTAGAACAGAGCCCTTA
GTATTAACGCAACACAATTATAATAATATTACTTTAGATAAGTGTGTTAACTATAATATATATGGCAGAGTGGGC
CAAGGTTTTATTACTAATGTGACTGATTCTGCTGCTAATTCTAGTTATTTAGCAGATGGTGGGTTAGCTATTTTA
GACACTTCGGGTGCCATAGATGTTTTTGTTGTACAGGGCAGCTATGGTCTTAATTATTACAAGGTCAATCCTT
GTGAAGATGTTAACCAACAGTTTGTAGTGTCTGGTGGCAATATAGTTGGCATTCTTACTTCTAGAAATGAAAC
AGGTTCTGAACAGGTTGAGAACCAGTTTTATGTTAAGTTAACCAATAGCTCACATCGTCGTAGGCGC
>CK_CH_QY_TN-ZJC-1_20210330_.seq
ATGTTGGTGAAGTCACTGTTTTTAGCGACTCTTTTGTTTGCACTATCTAGTGCTACTTTGTATGATAATGATACG
TACGTTTACTACTACCAGAGTGCCTTCAGACCGTCTAATGGTTGGCATTTACATGGTGGCGCTTATGCAGTAG
TAAATGTTTCTTCACAAACTAACAATGCAGGTACAGCTTCAGAATGCACTGTTGGTATTATTAGTGGTGATACA
GTTGTTAATGCCTCTTCTATAGCTATGACAGCACCTGTAGGTCAAGGTATGCAGTGGTCTAAGTTACAATTTTG
TACTGCACACTGCAATTTTTCTGATTTTACAGTGTTTGTTACACATTGCTATGCCTCGGGCAGCGGTAAATGTC
CTTTAACGGGCCTTATTCCACAAGGTCATATTCGTATTTCTGCTATGCGGAATCATACTTTATTCTATAATTTAAC
AGTTAGTGTATCTAAGTACCCTACTTTTAAATCTTTGCAATGCGTTGATAATTTCACATCTGTTTACTTAAATGG
TGACCTTGTCTTCACTTCTAATCAGACGACAGACGTTATAAGTGCAGGTGTGTACTTTAAATCACGTGGGCCT
ATAACCTATAAAGTTATGAAGGAATTTAAGGTTTTGGCTTATTTTGTTAATGGTACTGCACAAGATGTTATTTT
GTGTGATGACACACCTAGAGGTTTGCTAGCATGTCAATATAATACTGGCAATTTCTCAGATGGTTTTTATCCTT
TTACTAATAGTAGCTTAGTTAAGCAAAGGTTTGTTGTTTATCGTGAGAATAGTGTTAATACTGCTCTTACTTTA
ACCAATTACACCTTTCATAATGAGACTAATGCCCAGCCTAATTCAGGTGGTGTCCATACTATCTCAACTTATCA
AACACAAACTGCTCAGAGTGGTTATTATAATTTTAATTTATCATTTCTGAGTAGTTTTGTGTATAAAGATTCTGA
TTATATGTATGGGTCCTACCACCCACGATGTAGTTTTAGACCAGAAACTATTAATAATGGCTTGTGGTTTAATTC
ACTGTCAGTCTCATTAGCTTATGGCCCCCTTCAAGGTGGGTGTAAGCAATCAGTTTTTCAAGGCACAGCTACT
TGTTGTTATGCGTATTCCTATAACGGACCACGTATGTGTAAAGGTGTTTATAGTGGTCAGTTATCACAAGATTT
TGAATGTGGACTGTTGGTTTATGTTACTAAGAGTGATGGCTCTCGTATACAAACAGCCACAAAACCACCGGT
CATAACTCAACACAATTATAATAATATTACTTTAAATACTTGTGTTGAGTACAATATATATGGCAGAGTTGGCCA
AGGTTTTATTACTAATGTAACTGACTCCGCAGCTAGCTATAATTACTTAGCAGATGCTGGATTGGCAATTTTAG
ATACTTCAGGTGCCATAGACACTTTCGTTGTACAAGGTGAATATGGTCCCAATTATTATAAGGTTAACCCTTGT
GAAGATGTTAATCAGCAGTTTGTAGTGTCAGGCGGTAAGTTAGTAGGCATTCTGACTTCTCGTAATGAAACT

GGTTCTCAGCCTCTTGAAAATCAGTTTTATATTAAGTTAACTAATGGA
>CK_CH_YN_KM-HJB_20210705_.seq
ATGTTGGTGAAGTCACTGTTTTTAGTGACTCTTTTGTTTGCACTATCTAGTGCTACTTTGTATGATAATCATACG
TACGTTTACTACTACCAGAGCGCCTTCAGACCGTCTACTGGTTGGCATTTACATGGTGGCGCTTACGCAGTAG
TAAATGTTTCTTCACAAACTAACAATGCAGGTGCAGATCCAGAATGCACTGTTGGTATTATTAGTGGTGATAC
AGTTGTTAATGCCTCTTCTATAGCTATGACAGCACCTGTAGGTCAAGGTATGCGGTGGTCTAAGTCACAATTTT
GTACTGCACACTGCAATTTTTCTGATTTTACAGTGTTTGTTACACATTGCTATGTCCCGGGCAGTGGTAAATGT
CCTTTAACGGGCCTTATCCCAGAAGGTCATATTCGTATTTCTGCTATGCGGAATCATACTTTATTTTATAATTTAA
CAGTTAGTGTATCTAATTACCCTACTTTTAAATCTTTGCAATGCGTTGATAATTTCACGTCTGTTTACTTAAATG
GTGATCTTGTCTTCACTTCTAATCAGACTACACACGTTATAAGTGCAGGTGTGTACTTTAAAGCAGGTGGGCC
TATAACCTATAAAGTTATGAAGGAATTTAAGGTTTTGGCTTATTTTGTTAATGGTACTGCACAAGATGTTATTTT
GTGTGATGACACACCTAGAGGTTTGCTAGCATGTCAATATAATACTGGCAATTTCTCAGATGGTTTCTATCCTT
TTACTAATAGTAGCTTAGTTAGGCAAAGGTTTGTTGTTTATCGTGAGAATAGTGTTAATACTACTCTTACTTTAA
CCAATTACACCTTTCATAATGAGACTACTGCTCCGCCTAATTCAGGTGATGTTCATACTATCTTAACTTATCAAA
CACAAACTGCCCAGAGTGGTTATTATAATTTTAATTTATCATTTCTGAGTAGTTTTGAGTATAAAGCTTCTGATT
ATATGTATGGGTCTTACCACCCACGATGTAGTTTTAGACCAGAAACTATTAATAATGGCTTGTGGTTTAATTCA
CTCTCAGTCTCGTTAGCCTATGGCCCCCTTCAAGGTGGGTGTAAGCAATCAGTCTTTCAAGGCAGAGCTACT
TGTTGTTATGCGTATTCATATAACGGACCACGTATGTGTAAAGGTGTTTATAGTGGTCAGTTATTACAAGATTTT
GAATGTGGACTTTTGGTTTATGTTACTAAGAGTGATGGCTCTCGTATACAAACAACCACAAAACCACCGGTTA
TAACTCAACACAATTATAATAATATTACTTTAAATACTTGTGTTGAGTATAATATATATGGCAGAGTAGGCCAAG
GCTTTATTACTAATGTAACTGACTCCGCAGCTAGCTATAATTACTTAGCAGATGCTGGATTGGCAATTCTAGAT
ACTTCAGGTGCCATAGACACTTTCGTTGTACAAGGTGAATATGGTCCCAATTATTATAAGGTTAACCCTTGTGA
AGATGTTAACCAGCAGTTTGTAGTGTCAGGCGGTAAGTTAGTAGGCATCCTGACTTCTCGTAATGAAACTGG
CTCTCAGCCTCTTGAAAATCAGTTTTATATTAAGTTAACTAATGGA
>CK_CH_GD_QY_TN-WSN_20210322.seq
GATGTTGGAGAAGTCACTGTTGTTAGTGATCATTTTGTTTGCACTATGTAGTGCTAATTTGTTTGACGACTCTG
ATAAGTATGTGTACTACTACCAGAGTGGGTTCCGTCCTYCTACTGGGTGGCACCTGCATGGTGGTGCGTATGC
AGTAGAAAAAGTCTTTAATAGATCTAACAACGCCGGCAGTGTTAGATGTACTGCTGGAGCTATTGTAGATAGT
TTGAATTTTACGGCAAGTGCAGTGGCGATCACAACACCGCTTAATGGCATGTCCTGGTCTGCTGACAATGGC
ATTTGCTCAGCACATTGCAATTTTAGTAAAATTGTTGTTTTTGTAACACATTGTTTTGTAAGTGGTCCTGGTAC
TTGTCCCCTGACAGGTCGATTAGCCAACGGTGACATTCGTATTGGTGTTCTAGATAGTAGTGGTAATTCTATTT
TTAATAAAACAGTTACCACTTCTGTTTATAATAAATTTAAATCATTACATTGCGTTAACAATTTCACTTCTGTGTA
TTTAAATGGTGATCTTGTTTACACGTCTAATGAAACTTCTGATATTACTGGTTTTGGTGTAGATTTTAAGACAG
GAGGACCTGTTACTTATAAAATTATGAAAGAACATAAGGTTTTAGCATATTTTGAAAATGGTACTGCACATGA
CATAATATTATGTGATGACAGTCCCCGTGGTAGGTTAGCTTGTCAGTATAATACAGGCAATTTTTCTGATGGGT
TTTACCCCTTTAGTGTTGTTAATGAAGTTAATGAAACCTTCATAGTTTTTGAAAATAATTCAGTAAACACTACA
CTTACGTTGAATAATTTTACATACTTTAATGAGAGTGGTGCTCTACCTAATTTGCAGGAAGATTTACCTGGTGT
TTCTAAATTTTTATATTATCAACAGAATAGTGCTGTTCCTGGTTATAATAATTTTAATTTTTCATTTTTGAATGCTT
TCACTTATGTAAATAGTGATTATAATAGGGGTTCTTATCACCCTAGTTGTAATTTTAGGCCTGAAGATATTAATA
AAAATCGCAGGTTTAATCAACTGTCTGTCTCGTTATCTTATGGTCCTCGGAATGGAGGCTGTAAGCAAGCATG
TTTTAGTAGTAAGAGTTCTTGTTGTTGCTATATGTACTCTTACGGTGGTAAAACCCTGTGTAAAGGTGTTTATA
GTGGTGACTTAAAAACCAATTTTGAGTGCGTATTGCTTGTGTTTATTAAACATAGTCCAGGCAGTCGCATATT
CACATCGGAATCAGTACCATTTTTTACAACCAATTTTGTTAATAATGTGGTTATAGATAAGTGTGTTGATTATAA
TATTTATGTTAGTTATGGCAGGTTTTG

>CK_CH_GD_MX-LS-16_20210222_.seq
ATGTTGGAGAAGTTACTGTTTTTAGTGACCATTTTGTGTGCACTATGTAGTGCAAATTTGTTTGATGCTGATAA
TAGTTATGTGTACTACTACCAGAGTGGATTTAGACCCCCTTCAGGTTGGCACCTCTATGGTGGTGCGTATGCA
GTAGAACGGTTTTTTAATGAAACCAACAACGCAGGCTCTGGTGACTGTACTGCTGGAGCCATTGTACATAGT
TTAAATGTTACTGCAAGTGCAGTTGCGATTACTACACCTGTTAATGGCATGCATTGGTCATCTAGTACAGGAG
TGTGTTCAATACATTGCAATTTTAGTACAATTGTTGTTTTTGTTACACATTGTTTTAAAAATGGACAAGGAATAT
GTCCCTTGACAGGTAAATTAAAGGAGGGTGCTATTCGTATTGGTGTTCTAGATAGTAGTGGTAATTCTATTTTT
AATAAAACAGTTACCACTTCTAGTTATAGTAAATTTAAATCATTACATTGCGTTAACAATTTCACTTCTGTATATT
TAAATGGTGATCTTGTTTACACGTCTAATGAAACTTCAGATATTACTGGTTTTGGTGTACATTTCAAGACAGGA
GGACCTGTTACTTATAAAATTATGAAAGAACATAAGGTTCTAGCATATTTTGAAAATGGTACTGCACACGACA
TTATTTTATGTGATGACAGTCCCCGTGGTAGGTTAGCTTGTCAGTATAATACAGGCAATTTTTCTGACGGTTTG
TACCCTTTTAGCGTAAGCAGTGAAGTTAATGAAACTTTTATAGTTTTTGAAAAGAATACAGAAACTACTATGC
TTACATTAAATAATTTCACTTTTTTTAATCAGAGTGGGGCTCAACCTAATCAAAAGGAACCTTCACCTGGTGTT
TCAAATTTTGTGTATTATCAACAGACTAGTGCTGTTCCTGGTTATAATAATTTTAATTTTTCCTTTTTGAGTTCTT
TTACTTATTTAAGTAGTGATTATACGAAGGGTTCTTTTCACCCAAGTTGTACTTTTAGGCCTGAAGATATTAATA
AAAATCGCAGGTTTAATCATTTGTCTGTATCTTTATCTTATGGTCCTCGTAATGGAGGCTGTAAGCAAGCATGC
TTTAATACTAGGAGTTCATGTTGTTGTTTCATGTACTCTTATAATGGTCAACCTCTTTGTAAAGGTGTGTATAGT
GGTGATTTAAATCAAGATTTTGAGTGCGTATTGCTTGTGTTTATTAATCATAGCCCAGGCAGTCGTATATTTAC
TTCTGAAACAGTACCTACTGTCACCGCTAATTTTGCAAATAATGTGGTTTTAGATAGGTGTGTTGATTATAATAT
CTATGGTAGAGTAGGCAAAGGTTT
>CK_CH_JS_LH_NNY_20210601.seq
ATGTTGGAGAAGTTACTGTTTTTAGTGACCACTTTGTGTGCACTATGTAGTGCAAATTTGTTTGATGCTGATAA
TAGTTATGTGTACTACTACCAGAGTGGATTTAGACCTCCTTCAGGTTGGCACCTTTATGGTGGTGCGTATGCA
GTAGAACGTTTTTTTAATGAAACCAGCAATGCAGGCTCTGGTGCCTGTACTGCTGGAGCCATTGTACATAGTT
TAAATGTTACTGCAAGTGCAGTTGCGATTACTACACCTGTTAATGGCATGCATTGGTCATCTTATACAGGAGTG
TGTTCAATACATTGCAATTTTAGTACAATTGTTGTTTTTGTTACACATTGTTTTAAAAATGGACAAGGAATATGT
CCCTTGACAGGTAAATTAAAGGAGGGTGATATTCGTATTGGTGTTCTAGATAGTAGTGGTAATTCTATTTTTAA
TAAAACAGTTACCACTTCTAGTTATAGTAAATTTAAATCATTACATTGCGTTAACAATTTCACTTCTGTATATTTA
AATGGTGATCTTGTTTACACGTCTAATGAAACTTCAGATATTACTGGTTTTGGTGTACATTTTAAGACAGGAG
GACCTGTTACTTATAAAATTATGAAAGAACATAAGGTTCTAGCATATTTTGAAAATGGTACTGCACATGACATT
ATTTTATGTGATGACAGTCCCCGTGGTAGGTTAGCTTGTCAGTATAATACAGGCAATTTTTCTGACGGTTTGTA
CCCTTTTAGCGTAAGCAGTGAAGTTAATGAAACTTTTATAGTTTTTGAAAAGAATACAGAAACTACTATGCTT
ACATTAAATAATTTCACTTTTTTTAATCAGAGTGGGGCTCAACCTAATCAAAAGGAACCTTCACCTGGTGTTT
CAAATTTTGTGTATTATCAACAGATTAGTGCTGTTCCTGGTTATAATAATTTTAATTTTTCCTTTTTGAGTTCTTT
TACTTATTTAAGTAGTGATTATACGAGGGGTTCTTTTCACCCAAGTTGTACTTTTAGGCCTGAAGATATTAATA
AAAATCGCAGGTTTAATCATTTGTCTATATCTTTATCTTATGGTCCTCGTAATGGAGGCTGTAAGCAAGCATGC
TTTAATACTAGGAGTTCATGTTGTTGTTTCATGTACTCTTATAATGGTCAACCTTCTTGTAAAGGTGTGTATAGT
GGTTATTTAAATCAAGATTTTGAGTGCGTATTGCTTGTGTTTATTAATCATAGCCCAGGCAGTCGTATATTTACT
TCTGAAACAGTACCTACTGTCACTGCTAATTTTGCAAATAATGTGGTTTTAGATAGGTGTGTTGATTATAATATC
TATGGTAGAGTAGGTCAAGGTTGTATTTCTAATATAACTGATTCTGTGAAGGATTCAAATTATTTGGATTCTTCT
GGTTTAGCTATATTGGATCAATCGGGTGCTGTTGACACTTTCGTGATAAAAGGTCAATCAGGACCTAATTATTA
TAAAGTTAATCCATGTAAAGATGTTAATCAACAATATGTTGTTTCAGGCGGCAATATAGTCGGTCTTTTGTTCA
GTCTTAATAGTAGTGGTTCCCAGTTATTAGAGGACCAGTACTATGTTAGATTAACCAATACCTCACATAGGCAT
AAGCGT

>CK_CH_SC_YC-DK_LMB_20210104.seq
ATGTTGGAGAAGTTACTGTTTTTAGTGACCATTTTGTGTGCACTATGTAGTGCAAATTTGTTTGATGCTGATAA
TAGTTATGTGTACTACTACCAGAGTGGATTTAGACCTCCTGTAGGTTGGCACCTTTATGGTGGTGCGTATGCA
GTAGAACGGTTTTTTAATGAAACCAGCAATGCAGGCTCTGGTGCCTGTACTGCTGGAGCCATTGTACATAGT
TTAAATGTTACTGCAAGTGCAGTTGCGATTACTACACCTGTTAATGGCATGCATTGGTCATCTAGTACAGGAG
TGTGTTCAATACATTGCAATTTTAGTACAATTGCTGTTTTTGTTACACATTGTTTTAAAAATGGACAAGGAATA
TGTCCCTTGACAGGTAAATTAAAGAAGGGTGATATTCGTATTGGTGTTCTAGATAGTAGTGGTAATTCTATTTT
TAATAAAACAGTTACCACTTCTAGTTATAGTAAATTTAAATCATTACATTGCGTTAACAATTTCACTTCTGTATAT
TTAAATGGTGATCTTGTTTACACGTCTAATGAAACTTTAGATATTACTGGTTTTGGTGTACATTTTAAGACAGG
AGGACCTGTTACTTATAAAATTATGAAAGAACATAAGGTTCTAGCATATTTTGAAAATGGTACTGCACATGACA
TTATTTTATGTGATGACAGTCCCCGTGGTAGGTTAGCTTGTCAGTATAATACAGGCAATTTTTCTGACGGTTTG
TACCCTTTTAGCGTAAGCAGTGAAGTTAATGAAACTTTTATAGTTTTTGAAAAGAATACAGAAACTACTATGC
TTACATTAAATAATTTCACTTTTTTTAATCAGAGTGGGGCTCAACCTAATCAAAAGGAACCTTCACCTGGTGTT
TCAAATTTTGTGTATTATCAACAGATTAGTGCTGTTCCTGGTTATAATAATTTTAATTTTTCCTTTTTGAGTTCTT
TTACTTATTTAAGTAGTGATTATACGAGGGGTTCTTTTCACCCAAGTTGTACTTTTAGGCCTGAAGATATTAAT
AAAAATCGCAGGTTTAACCATTTGTCTATATCTTTATCTTATGGTCCTCGTAATGGAGGCTGTAAGCAAGCATG
CTTTAATACTAGGAGTTCATGTTGTTGTTTCATGTACTCTTATAATGGTCAACCTTTTTGTAAAGGTGTGTATAG
TGGTGATTTAAATCAAGATTTTGAGTGCGTATTGCTTGTGTTTATTAATCATAGCCCAGGCAGTCGTATATTTAC
TTCTGAAACAGTACCTACTGTCACTGCTAATTTTGCAAATAATGTGGTTTTAGATAGGTGTGTTGATTATAATAT
CTATGTTAGAGTGG
>CK_CH_SD_LY_CHJC-6_20210306.seq
ATGTTGGAGAAGTTACTGTTTTTAGTGACCATTTTGTGTGCACTATGTAGTGCAAATTTGTTTGATGCTGATAA
TAGTTATGTGTACTACTACCAGAGTGGATTTAGACCTCCTGTAGGTTGGCACCTTTATGGTGGTGCGTATGCA
GTAGAACGGTTTTTTAATGAAACCAGCAATGCAGGCTCTGGTGCCTGTACTGCTGGAGCCATTGTACATAGT
TTAAATGTTACTGCAAGTGCAGTTGCGATTACTACACCTGTTAATGGCATGCATTGGTCATCTAGTACAGGAG
TGTGTTCAATACATTGCAATTTTAGTACAATTGCTGTTTTTGTTACACATTGTTTTAAAAATGGACAAGGAATA
TGTCCCTTGACAGGTAAATTAAAGGAGGGTGATATTCGTATTGGTGTTCTAGATAGTAGTGGTAATTCTATTTT
TAACAAAACAGTTACCACTTCTAGTTATAGTAAATTTAAATCATTACATTGCGTTAACAATTTCACTTCTGTATAT
TTAAATGGTGATCTTGTTTACACGTCTAATGAAACTTCAGACATTACTGGTTTTGGTGTACATTTTAAGACAGG
AGGACCTGTTACTTATAAAATTATGAAAGAACATAAGGTTCTAGCATATTTTGAAAATGGTACTGCACATGACA
TTATTTTATGTGATGACAGTCCCCGTGGTAGGTTAGCTTGTCAGTATAATACAGGCAATTTTTCTGACGGTTTG
TACCCTTTTAGCGTAAGCAGTGAAGTTAATGAAACTTTTATAGTTTTTGAAAAGAATACAGAAACTACTATGC
TTACATTAAATAATTTCACTTTTTTTAATCAGAGTGGGGCTCAACCTAATCAAAAGGAACCTTCACCTGGTGTT
TCAAATTTTGTGTATTATCAACAGATTAGTGCTGTTCCTGGTTATAATAACTTTAATTTTTCCTTTTTGAGTTCTT
TTACTTATTTAAGTAGTGATTATACGAGGGGTTCTTTTCACCCAAGTTGTACTTTTAGGCCTGAAGATATTAAT
AAAAATCGCAGGTTTAACCATTTGTCTATATCTTTATCTTATGGTCCTCGTAATGGAGGCTGTAAGCAAGCATG
CTTTAATACTAGGAGTTCATGTTGTTGTTTCATGTACTCTTATAATGGTCAACCTTTTTGTAAAGGTGTGTATAG
TGGTGATTTAAATCAAGATTTTGAGTGCGTATTGCTTGTGTTTATTAACCATAGCCCAGGCAGTCGTATATTTA
CTTCTGAAACAGTACCTACTGTCACTGCTAATTTTGCAAATAATGTGGTTTTAGATAGGTGTGTTGATTATAATA
TCTATGGTAGAGTAGGTCAGGGTTGTATTTCTAATATAACTGATTCTGTGAAGGATTCAAATTATTTGGATTCT
TCTGGTTTAGCTATATTGGATCAATCGGGTGCTGTTGACACTTTCGTGATAAAAGGTCAATCAGGACCTAATTA
TTATAAAGTTAATCCATGTAAAGATGTTAATCAACAATATGTTGTTTCAGGCGGCAATATAGTCGGTCTTTTGTT
CAGTCTTAA
>CK_CH_SC_YC-DK_YL_20210104.seq

ATGTTGGAGAAGTTACTGTTTTTAGTGACCATTTTGTGTGCACTATGTAGTGCAAATTTGTTTGATGCTGATAA
TAGTTATGTGTACTACTACCAGAGTGGATTTAGACCTCCTGTAGGTTGGCACCTTTATGGTGGTGCGTATGCA
GTAGAACGGTTTTTTAATGAAACCAGCAATGCAGGCTCTGGTGCCTGTACTGCTGGAGCCATTGTACATAGT
TTAAATGTTACTGCAAGTGCAGTTGCGATTACTACACCTGTTAATGGCATGCATTGGTCATCTAGTACAGGAG
TGTGTTCAATACATTGCAATTTTAGTACAATTGCTGTTTTTGTTACACATTGTTTTAAAAATGGACAAGGAATA
TGTCCCTTGACAGGTAAATTAAAGGAGGGTGATATTCGTATTGGTGTTCTAGATAGTAGTGGTAATTCTATTTT
TAATAAAACAGTTACCACTTCTAGTTATAGTAAATTTAAATCATTACATTGCGTTAACAATTTCACTTCTGTATAT
TTAAATGGTGATCTTGTTTACACGTCTAATGAAACTTTAGATATTACTGGTTTTGGTGTACATTTTAAGACAGG
AGGACCTGTTACTTATAAAATTATGAAAGAACATAAGGTTCTAGCATATTTTGAAAATGGTACTGCACATGACA
TTATTTTATGTGATGACAGTCCCCGTGGTAGGTTAGCTTGTCAGTATAATACAGGCAATTTTTCTGACGGTTTG
TACCCTTTTAGCGTAAGCAGTGAAGTTAATGAAACTTTTATAGTTTTTGAAAAGAATACAGAAACTACTATGC
TTACATTAAATAATTTCACTTTTTTTAATCAGAGTGGGGCTCAACCTAATCAAAAGGAACCTTCACCTGGTGTT
TCAAATTTTGTGTATTATCAACAGATTAGTGCTGTTCCTGGTTATAATAATTTTAATTTTTCCTTTTTGAGTTCTT
TTACTTATTTAAGTAGTGATTATACGAGGGGTTCTTTTCACCCAAGTTGTACTTTTAGGCCTGAAGATATTAAT
AAAAATCGCAGGTTTAACCATTTGTCTATATCTTTATCTTATGGTCCTCGTAATGGAGGCTGTAAGCAAGCATG
CTTTAATACTAGGAGTTCATGTTGTTGTTTCATGTACTCTTATAATGGTCAACCTTTTTGTAAAGGTGTGTATAG
TGGTGATTTAAATCAAGATTTTGAGTGCGTATTGCTTGTGTTTATTAATCATAGCCCAGGCAGTCGTATATTTAC
TTCTGAAACAGTACCTACTGTCACTGCTAATTTTGCAAATAATGTGGTTTTAGATAGGTGTGTTGATTATAATAT
CTATGTTAGTTGG
>CK_CH_GD_YF-XX_PHZ-MSZ_20211206.seq
ATGTTGGTAACACCTCTTTTACTAGTGACTCTTTTGTGTGCACTATGTAGTGCTGTTTTGTATGACAGTAGTTC
TTACGTGTACTACTACCAAAGTGCCTTCAGACCACCTGATGGTTGGCATTTACATGGGGGTGCGTATGCGGTT
GTTAATATTTCTAGTGAATCTAATAATGCAGGCTCTTCATCTGGGTGTACTGTTGGTATTATTCATGGTGGTCGT
GTTGTTAATGCTTCTTCTATAGCTATGACGGCACCGTCATCAGGTATGGCTTGGTCTAGCAGTCAGTTTTGTAC
TGCATACTGTAACTTTTCAGATACTACAGTGTTTGTTACACATTGTTATAAACATGTTGGGTGTCCTATAACTGG
CATGCTTCAACAGCATTCTATACGTGTTTCTGCTATGAAAAATGGCCAGCTTTTTTATAATTTAACAGTTAGTGT
AGCTAATTACCCTACTTTTAAATCATTTCAGTGTGTTAATAATTTAACATCCGTATATTTAAATGGTGATCTTGTT
TACACCTCTAATGAGACCACAGATGTTACATCTGCAGGTGTTTATTTTAAAGCTGGTGGACCTATAACTTATAA
AGTTATGAGAGAAGTTAGAGCCCTGGCTTATTTTGTTAATGGTACTGCACAAGATGTTATTTTGTGTGATGGG
TCACCTAGAGGCTTGTTAGCATGCCAGTATAATACTGGCAATTTTTCAGATGGCTTTTATCCTTTTACTAATAGT
AGTTTAGTTAAGCAGAAGTTTATTGTCTATCGTGAAAATAGTGTTAATACTACTTTTACGTTACACAATTTCACT
TTTCATAATGAGACTGGCGCCAACCCAAATCCTAGTGGTGTCCAGAATATTCAAACTTACCAAACACAAACA
GCTCAGAGTGGTTATTATAATTTTAATTTTTCCTTTCTGAGTAGTTTTGTTTATAAGGAGTCTAATTTTATGTATG
GATCTTATCACCCAAGTTGTAATTTTAGACTAGAAACTATTAATAATGGTTTGTGGTTTAATTCACTTTCAGTTT
CAATTGCTTACGGTCCTCTTCAAGGTGGTTGCAAGCAATCTGTCTTTAGTGGTAGAGCAACCTGTTGTTATGC
TTACTCATATGGAGGTCCTTTGCTGTGTAAAGGTGTTTATTCAGGTGAGTTAGATCATAATTTTGAATGTGGAC
TGTTAGTTTATGTTACTAAGAGCGGTGGCTCTCGTATACAAACAGCCACTGAACCGCCAGTTATAACTCAACA
CAATTATAATAATATTACTTTAAATACTTGTGTTGATTATAATATATATGGCAGAACTGGCCAAGGTTTTATTACT
AATGTAACCGACTCAGCTGTTAGTTATAATTATCTAGCAGACGCAGGTTTGGCTATTTTAGATACATCTGGTTC
CATAGACATCTTTGTCGTACAAAGTGAATATGGTCTTAATTATTATAAGGTTAACCCTTGCGAAGATGTCAACC
AGCAGTTTGTAGTTTCTGGTGGTAAATTAGTAGGTATTCTTACTTCACGTAATGAGACTGGTTCCCAGCTTCTT
GAGAATCAGTTTTACATCAAAATCACTAATGGAACACGTCGTTTTAGACGT
>CK_CH_GX_NN_LY-ZDC_20211213.seq
ATGTTGGGGAAGTCACTGTTGCTAGTGACCATTTTGTGTGTACTATGTAGTGCAAATTTGTTTGATTCTAAGTA

TGTTTACTACTATCAAAGTGCCTTTAGACCATCAGGTGGATGGCATTTGCATGGGGGTGCTTATGCAGTAGTG
AATGCTACTAATAAAACTAATAATGCAGGCGCCGCTACAGAGTGTTCTGTAGGTGTTCTTTTTAATTATACTAA
CGGAAATGACGTTGGTTATAAATATAATGCTTCTTCTATAGCCATGACAGCACCGTTGCCGGGTATGTCTTGGT
CTAAAACACAATTTTGTACTGCCCACTGTAACTTTTCGGATTTTACAGTGTTTGTTACACATTGTTTTGCAAAT
TCTTGTCCTTTAACAGGTAGGATACAAGAGAACCATATTCGTATTTCTGCTATGAGAAATGGTTCTCTCTTTTAT
AATTTAACAGTTAGTGTATCTAACTACCCTAAATTTAAATCGCTTCAATGTGTTAACAATTTCACTTCTGTTTATT
TAAATGGTGATCTTGTTTTTACTTCTAACGAAACCACTGATGTTATAGGTGCTGGTGTGTATTTCAAATCAGGT
GGGCCTATAACCTATAAAATTATGAAAGAATTTAAGGTTTTGGCTTATTTTGTAAATGGCACTGCGCAAGATG
TAATTCTGTGTGATGACACACCTAGAGGCTTGCTTGCGTGTCAATATAATACCGGTAATTTTACGGATGGTTTT
TATCCATTTACTAATAGCAGTTTAGTTAAGGAAAGATTTATTGTTTACCGTGAAAGTAGTGTTAATACTACTTTG
GCGTTAACTAATTTTACTTTTTATAATGAGACTAATGCACAACCTAATTCTGTAGGTGGTGTTAATAGTATTCAA
ACTTATCAAACACAAACAGCTCAGAGTGGTTATTATAATTTTAATTTATCATTTCTGAGTAGTTTTTTGTATGTA
GATTCCAATTATATGTATGGTTCTTATCACCGTAGCTGTAATTTTAGACCAGAAAATATTAATAATGGCTTATGG
TTTAATTCACTCTCAGTTTCAGTTGCATATGGACCTCTTCAAGGTGGGTGTAAGCAGTCTGTTTTTAGTGGTA
GAGCGACTTGTTGTTATGCTTATTCTTATAACGGTCCTTACGCTTGTAAAGGTGTTTACCCAGGTGAGTTAAC
AAAGAGTTTTGAATGTGGTTTATTGGTTTATGTTACTAAGAGCGATGGCTCTCGTATACAAACAGCCAATGAA
GCACCAGTTATAACTCAACACAATTATAATAATATTACTTTAAATACTTGTGTTGATTATAATATATATGGCAGAG
TAGGCCAAGGGCTTATTACTAATGTAACTGAGTCAGCTTCTAGTTATAATTATCTAGCAGATGCAGGTTTAGCT
ATTTTAGATACTTCAGGAGCCATAGACATATTTGTTGTTCGAGGTGCATATGGTCCTAATTATTATAAGGTTAAT
CCCTGTGAAGATGTTAATCAACAGTTTGTAGTGTCTGGTGGCAATATAGTTGGCATTCTTACTTCTAGAAATG
AAACAGGTTCTGAACAGGTTGAGAACCAGTTTTATGTTAAGTTAACCAATAGCCCACATCGTCGCAGGCGT
>CK_CH_YN_KM_I2_20210506_2.seq
ATGTTGGGGAAGTCACTGTTTTTAATGACCATTTTGTCTGCACTATGTAGTGCAGTTTTGTATGATGATAATAC
TTATGTGTACTACTACCAAAGTGCCTTTAGACCATCAAATGGATGGCATTTGCAGGGGGGTGCTTATGCAGTA
GTGAATTCTACTATTAAATTTAACAATGCAGGCTCCGCTAGAGAGTGTTCTGTAGGTGTTCTTTTTAATTATTCT
AATGGAAATGATGTTGGTTATAATAATAGTGCTTCTTCCGTAGCCATGACAGCACCGTTGTCTGGTATGTCTTG
GTCTAAAAGCGAATTTTGTACTGCCCACTGTAATTTTTCGGATATTACAGTGTTTGTTACACATTGTTATGCAC
AATCTTGTCCTTTAACAGGTCAGATACAGAAAGGCCATATTCGTATTTCTGCTATGAGAAATGGTTTTCTATTT
TATAATTTAACAGTTAGTGTATCCCAATACCCTAAATTTAAATCGCTTCAATGTGTCAACAACCTTACTTCTGTTT
ATTTAAATGGTGACCTTGTTTTTTCTTTTAATAAAAGCATTGATGTTACAGGTGCTGGTGTGTACTTTAAATCA
GGTGGGCCTATAACCTACAAAATTATGAAAGAATTTAAGGTTTTGGCTTATTTTCTAAATGGCACTGCGCAAG
ATGTAATTTTGTGTGATGACACACCGAGAGGCTTGCTTGCATGTCAATATAATACTGGTAATTTTTCAGATGG
GTTTTATCCTTTTACTAATTCTAGTTTAGTTAAGGAAAAGTTTATTGTTTATCGTGAGAATAGTGTTAATACTAC
TCTTACTTTAACTAACTATACTTTTTTTAATGTGACTGATGCCTCTCCTAATCAAGGTGGTGTTCATTCTATTCTA
ACTTATCAAACACAAACAGCTCAGGATGGTTATTATAATTTTAATTTATCATTTCTGAGTAGTTTTGTGTATAAA
GAGTCTAATTACATGTATGGGTCTTATCACCCTGCATGTAATTTTAGATTAGAAACTATTAACAATGGCTTGTG
GTTTAATTCACTGTCAGTTTCGTTAGCTTATGGACCACTTCAAGGTGGGTGTAAGCAGTCGGTATTTAGTGAT
AGAGCCACTTGTTGTTATGCTTATTCATATAATGGTCCTCGCCTCTGTAAGGGTGTTTACAGTGGCGAGTTACA
ACAAAGTTTTGAGTGTGGACTGCTGGTTTATGTTACTAAGCGCGATGGCTCTCGTATACAAACAGCCACCAG
TCCACCAGTTATAACTCAACACAATTATAATAATATTACTTTAAATAAGTGTGTTGATTATAATATATATGGCAGA
TTTGGCCAAGGGTTTATTACTAATGTAACTGACTCAGCTGCTAGCTATAATTATCTAGCTGATGCAGGGTTAGC
TATTTTAGATACGTCTGGAGCCATAGATATCTTTGTTGTACAAGGTGCATATGGTCTTAATTATTATAAGGTTAA
CCCTTGTGAGGATGTTAACCAACAGCTTGTAGTGTCTGGTGGTGGTATAGTTGGCATGCTTACTTCTAGAAAT
GAAACAGGTTCTGAACTGGTTGAGAACATGTTTTATGTTAGGTTAGCTAATAGCTCGCGTCGCTTCAGGCGT

>CK_CH_YN_KM_I2_20210506_1.seq
ATGTTGGTGAAGTCACTGTTTTTAGTGACCATTTTGTCTGCACTATGTAGTGCAGTTTTGTATGATAATAATACT
TATGTGTACTACTACCAAAGTGCCTTTAGACCATCAAATGGATGGCATTTGCAGGGGGGTGCTTATGCAGTAG
TGAATTCTACTATTAAATATAACAATGCAGGCTCCGCTAGTGGGTGTTCTGTAGGTGTTCTTTTTAATTATTCTA
ATGGAAATGATGTTGGTTATAATAATAGTGCTTCTTCCGTAGCCATGACAGCACCGTTGTTTGGTATGTCTTGG
TCTAAAGGAGAATTTTGTACTGCCCACTGTAATTTTTCGGATATTACAGTGTTTGTTACACATTGTTATGCACA
ATCTTGTCCTTTAACAGGTCAGTTACAGAAAGGCCATATTCGTATTTCTGCTATGAGAAATGGTTCTCTATTTTA
TAATTTAACAGTTAGTGTATCCCAATACCCTAAATTTAAATCGCTTCAATGTGTCAACAACCTTACTTCTGTTTA
TTTAAATGGTGACCTTGTTTTTCTTTTAATAAAAGCATTGATGTTACAGGTGCTGGTGTGTACTTTAAAGCAG
GTGGGCCTATAACCTACAAAATTATGAAAGAATTTAAGGTTTTGGCTTATTTTCTAAATGGCACTGCGCAAGA
TATAATTTTGTGTGATGACACACCGAGAGGCTTGCTTGCATGTCAATATAATACTGGTAATTTTTCAGATGGGT
TTTATCCTTTTACTAATTCTAGTTTAGTTAAGGAAAAGTTTATTGTTTATCGTGAGAATAGTGTTAATACTACTC
TTACTTTAACTAACTATACTTTTTTTAATGAGACTAATGCCTCTCCTAATCAAGGTGATGTTCATTCTATTTCAAC
TTATCAAACACAAACAGCTCAGGATGGTTATTATAATTTTAATTTATCATTTCTGAGTAGTTTTGTGTATAAAGA
GTCTAATTACATGTATGGGTCTTATCACCCTGCATGTAATTTTAGATTAGAAACTATTAACAATGGCTTGTGGTT
TAATTCACTGTCAGTTTCGTTAGCTTATGGACCACTTCAAGGTGGGTGTAAGCAGTCGGTCTTTAGTGGTAGA
GCCACTTGTTGTTATGCTTATTCATATAGTGGTCCTCACCTCTGTAAGGGTGTTTACAGTGGCGAGTTACAAA
AAAGTTTTGAGTGTGGACTGCTGGTTTATGTTACTAAGCGCGATGGCTCTCGTATACAAACAGCCACCATTCC
ACCAGTTATAACTCAACACAATTATAATAATATTACTTTAAATAAGTGTGTTGATTATAATATATATGGCAGATTT
GGCCAAGGGTTTATTACTAATGTAACTGACTCAGCTGCTAGCTATAATTATCTAGCTGATGCAGGGTTAGCTAT
TTTAGATACGTCTGGAGCCATAGATATCTTTGTTGTACAAGGTGCATATGGTCTTAATTACTATAAGGTTAACCC
TTGTGAGGATGTTAACCAACAGCTTGTAGTGTCTGGTGGTGGTATAGTTGGCATGCTTACTTCTAGAAATGAA
ACAGGTTCTGAACTGGTTGAGAACATGTTTTATGTTAGGTTAGCTAATAGCTCGCGTCGCTTCAGGCGT
>CK_CH_ZJ_TZ-LH_LCH_20210201.seq
ATGTTGGGGAAGTCACTGTTTTTAGTGACCATTTTGTGTGCACTATGTAGTGCAAATTTGTTTGGTTCTGCCA
ATAATTATGTGTACTACTACCAAAGTGCCTTTAGGCCTCCAAATGGATGGCACTTGCATGGGGGTGCTTATGC
AGTAGTGAATTCTACTATTAAATATAACAATGCAGGCTCCGCTAGTGCGTGTTCTGTAGGTGTTCTTTTTAATTA
TTCTAACGGAAATGATGTTGGTTATAATAATAGTGCTTCTTCTGTAGCCATGACAGCACCCTTGTCTGGTATGT
CTTGGTCTAAAGATGAATTTTGTACTGCCCACTGTAACTTTTCGGATATTACAGTGTTTGTTACACATTGTTATG
CACAATCTTGTCCTTTAACGGGTCAGTTAGGTAAGGGCCATATTCGTATTTCTGCTATGAGAAATGGTTCTCTA
TTTTATAATTTAACAGTTGGTGTATCTCAATACCCTAAATTTAAATCGCTTCAATGTGTTAACAACTTCACTTCT
GTTTATTTAAATGGTGACCTTGTTTTTACTTCTAATGAAAGCATTGATGTTATAGGTGCTGGTGTGTACTTTAA
AGCAGGTGGGCCTATAACCTACAAAATTATGAGAGAATTTAAGGTTTTGGCTTATTTTTTAAATGGCACTGCG
CAAGATGTAATTTTGTGTGATGGCACACCGAGAGGCTTGCTTGCATGTCAATATAATACTGGTAATTTTTCAG
ATGGGTTTTATCCTTTTACTAATTCTAGTTTAGTTAAGGAAAAGTTTATTGTTTATCGTCAGAATAGTGTCAATA
CTACTCTTACCTTAACTAACTATACTTTTTTTAATGAGACTAACGCCCCTCCTAATCCAGGTGATGTTCATTCTAT
TCCAACTTATCAAACACAAACAGCTCAGAGTGGTTATTATAATTTTAATTTATCATTTCTGAGTAGTTTTGTGTA
TAAGGAGTCTAATTACATGTACGGGTCTTATCACCATGCATGCAATTTTAGATTAGAAAATATTAATAATGGCTT
GTGGTTTAATTCACTGTCAGTTACGCTAGCTTATGGACCACTTCAAGGTGGGTGTAAGCAGTCGGTCTTTAGT
GGTAGAGCCACTTGTTGTTATGCTTATTCATATAATGGTCCTCACCTCTGTAAGGGTGTTTACAGCGGCGAGTT
AAAACAAAGTTTTGAATGTGGATTGTTGGTTTATGTTACTAAGCGTGATGGCTCTCGTATACAAACAGCCACC
GTTCCACCAGTTATAACTCAACACAATTATAATAATATTACTTTAAATAAGTGTGTTGACTATAATATATATGGCA
GAGTAGGCCAAGGTTTTATTACTAATGTGACTGATTCTGCTGCTAATTTTAGTTATTTAGCAGATGGTGGGTTA
GCTATTTTAGATACTTCGGGTGCCATAGATGTTTTTGTTGTACAGGGCAGCTTTGGTCTTAATTATTACAAGGT

CAATCCTTGTGAAGATGTTAACCAACAGTTTGTAGTGTCTGGTGGCAACATAGTTGGCATTCTTACTTCTAGA
AATGAAACAGGTTCTGAACAGGTTGAGAACCAGTTTTATGTTAAGTTAACCAATAGCTCACATCGTCGTAAG
CGT
>CK_CH_GD_QY_HHH-60d_20210616.seq
ATGTTGGGGAAGTCACTGTTTTTAGTGACCATTTTGTGTGCACTATGTAGTGCAAATTTGTTTGATTCTGCTAA
TAATTATGTGTACTACTACCAAAGTGCCTTTAGGCCTCCAAATGGATGGCATTTGCAAGGGGGTGCTTATGCA
GTAGTGAATTCTACTAATTATACTAATAATGCAGGTCCTGCAAGTGAGTGCACTATTGGTGTTATTAAGGACGT
CTATAATCAAAGTGCGGCTGCTATAGCTATGACAGCACCTCTTCAGGGTATGACTTGGTCTAAGTCACAATTTT
GTAGTGCACACTGTAACTTTTCTGAAATTACAGTTTTTGTCACACATTGTTATAGTAGCGGTAGTTCGTCTTGT
CCTATAACAGGCATGATTCCACAGGGTCATATTCGCATTTCTGCAATGAAAAATGGCTCTTTATTTTATAATTTA
ACAGTTAGCGTGTCTAAATACCCTAATTTTAAATCGTTTCAATGTGTTAACAACTTCACATCTGTTTATTTAAAT
GGTGATCTTGTTTTTACTTCTAACACAACTACTGATGTTAAGTCAGCAGGTGTGTATTTTAAAGCAGGTGGAC
CTGTAAATTATAATATTATGAAAGAATTTAAGGTTCTGGCTTATTTTGTCAATGGTACTGTGCGAGATGTAATTC
TGTGTGATGACACACCGAGAGGCTTGCTTGCATGTCAATATAATAATGGTAATTTTTCAGATGGGTTTTACCCT
TTTACTAATTCTAGTTTAGTTAAGGAAAAGTTTATTGTTTATCGTGAGAATAGTGTTAATACTACTCTTACTTTA
ACTAACTATACTTTTTATAATGTGACTAATGCCTCGCCTAATGGAGGTGGTGTTCAGTCTATTCCAACTTATCAA
ACACAAACAGCTCAGAGTGGTTATTATAATTTTAATTTATCATTTCTGAGTAGTTTTGTGTATAAAGAGTCTAAT
TACATGTATGGGTCTTACCACCCTGCATGTAATTTTAGATTAGAAACTATTAATAATGGCTTGTGGTTTAATTCA
TTGTCAGTTTCGCTTGCTTATGGACCACTTCAAGGTGGGTGTAAGCAGTCGGTTTTTAGTAGTAGAGCCACT
TGTTGTTATGCTTATTCATATAATGGTCCTCGCGCATGTAAGGGTGTTTACGCAGGCGAGTTACAACAAGATTT
TGAATGTGGACTGTTGGTTTATGTTACTAAGAGCGATGGCTCTCGTATACAAACAGCCACCGTTCCACCAGTT
GTAACTCAACACAATTATAATAATATTACTTTAAATACTTGTGTTGATTATAATATATATGGCAGAGTTGGTCGA
GGTTTTATTACTAATGTAACTGACTCATCATCTAGTTATAATTATTTAGCAGATGCAGGGTTGGCTATTTTAGAT
ACATCAGGTGCCATAGACATCTTTGTTGTACAAGGTGAACATGGTCTTAATTATTACAAGGTTAATCCCTGTGA
AGATGTAAACCAGCAGTTTGTAGTTTCTGGTGGTAAATTAGTAGGTATTCTTACCTCACGTAATGCAACAGGT
TCTCAGCCTCTTGAGAATCAATTCTACATTAAACTCACTAAAGAGACACGTCGTTTTAGACGT
>CK_CH_JS_LH-GHP_20210205.seq
ATGTTGGGGAAGTCACTGTTTTTAGTGACCATTTTGTGTGCACTATGTAGTGCAAAATTGTTTGATTCTGCTAA
TAATTATGTGTACTACTACCAAAGTGCCTTTAGGCCTCCAAATGGATGGCATTTGCAAGGGGGTGCTTATGCA
GTAGTGAATTCTACTAATTATACTAATAATGCAGGTTCTGCAAATGAGTGCACTATTGGTGTTATTAAGGACGT
CTATAATCAAAGTGCGGCTGCTATAGCTATGACAGCACCTCTTCAGGGTATGGCTTGGTCTAAGTCACAATTT
TGTAGTGCACACTGTAACTTTTCTGAAATTACAGTTTTTGTCACACATTGTTATAGTAGCGGTAGTTGGTCTTG
TCCTATAACAGGCATGATTCCACAGGGTCATATTCGCATTTCTGCAATGAAAAATGGCTCTTTATTTTATAATTT
AACAGTTAGCGTGTCTAAATACCCTAATTTTAAATCGTTTCAATGTGTTAACAACTTCACATCTGTTTATTTAAA
TGGTGATCTTGTTTTTACTTCTAACACAACTACTGATGTTAAGTCAGCAGGTGTGTATTTTAAAGCAGGTGGA
CCTGTAAATTATAATATTATGAAAGAATTTAAGGTTCTGGCTTATTTTGTCAATGGTACTGTGCAAGATGTAATT
CTGTGTGATGACACACCGAGAGGCTTGCTTGCATGTCAATATAATAATGGTAATTTTTCAGATGGGTTTTACC
CTTTTACTAATTCTAGTTTAGTTAAGGAAAAGTTTATTGTTTATCGTGAGAATAGTGTTAATACTACTCTTACTT
TAACTAACTATACTTTTTATAATGTGACTAATGCCTCGCCTAATCGAGGTGGTGTTCAGTCTATTCCAACTTATC
AAACACAAACAGCTCAGAGTGGTTATTATAATTTTAATTTATCATTTCTGAGTAGTTTTGTGTATAAAGAGTCT
AATTACATGTATGGGTCTTACCACCCTGCATGTAATTTTAGATTAGAAACTATTAATAATGGCTTGTGGTTTAAT
TCATTGTCAGTTTCGCTTGCTTATGGACCACTTCAAGGTGGGTGTAAGCAGTCGGTTTTTAGTAGTAGAGCC
ACTTGTTGTTATGCTTATTCATATAATGGTCCTCGCGCATGTAAGGGTGTTTACGCAGGCGAGTTACTACAAAA
TTTTGAATGTGGACTGTTGGTTTATGTTACTAAGAGCGATGGCTCTCGTATACAAACAGCCACCGTTCCACCA

GTTGTAACTCAACACAATTATAATAATATTACTTTAAATACTTGTGTTGATTATAATATATATGGCAGAGTTGGTC
GAGGTTTTATTACTAATGTAACTGACTCATCATCTAGTTATAATTATTTAGCAGATGCAGGGTTGGCTATTTTAG
ATACATCAGGTGCCATAGACATCTTTGTTGTACAAGGTGAACATGGTCTTAATTATTACAAGGTTAATCCCTGT
GAAGATGTAAACCAGCAGTTTGTAGTTTCTGGTGGTAAATTAGTAGGTATTCTTACCTCACGTAATGCAACAG
GTTCTCAGCCTCTTGAGAATCAATTCTACATTAAACTCACTAAAGAGACACGTCGTTTTAGACGT
>CK_CH_ZJ_TZ-LH_ZJH-2_20211110.seq
ATGTTGGGGAAGTCACTGTTTTTAGTGACCATTTTGTGTGCACTATGTAGTGCAAAATTGTTTGATTCTGCTAA
TAATTATGTGTACTACTACCAAAGTGCCTTTAGGCCTCCAAATGGATGGCATTTGCAAGGGGGTGCTTATGCA
GTAGTGAATTCTACTAATTATACTAATAATGCAGGTTCTGCAAATGAGTGCACTATTGGTGTTATTAAGGACGT
CTATAATCAAAGTGCGGCTGCTATAGCTATGACAGCACCTCTTCAGGGTATGGCTTGGTCTAAGTCACAATTT
TGTAGTGCACACTGTAACTTTTCTGAAATTACAGTTTTTGTCACACATTGTTATAGTAGCGGTAGTTGGTCTTG
TCCTATAACAGGCATGATTCCACAGGGTCATATTCGCATTTCTGCAATGAAAAATGGCTCTTTATTTTATAATTT
AACAGTTAGCGTGTCTAAATACCCTAATTTTAAATCGTTTCAATGTGTTAACAACTTCACATCTGTTTATTTAAA
TGGTGATCTTGTTTTTACTTCTAACACAACTACTGATGTTAAGTCAGCAGGTGTGTATTTTAAAGCAGGTGGA
CCTGTAAATTATAATATTATGAAAGAATTTAAGGTTCTGGCTTATTTTGTCAATGGTACTGTGCAAGATGTAATT
CTGTGTGATGACACACCGAGAGGCTTGCTTGCATGTCAATATAATAATGGTAATTTTTCAGATGGGTTTTACC
CTTTTACTAATTCTAGTTTAGTTAAGGAAAAGTTTATTGTTTATCGTGAGAATAGTGTTAATACTACTCTTACTT
TAACTAACTATACTTTTTATAATGTGACTAATGCCTCGCCTAATCGAGGTGGTGTTCAGTCTATTCCAACTTATC
AAACACAAACAGCTCAGAGTGGTTATTATAATTTTAATTTATCATTTCTGAGTAGTTTTGTGTATAAAGAGTCT
AATTACATGTATGGGTCTTACCACCCTGCATGTAATTTTAGATTAGAAACTATTAATAATGGCTTGTGGTTTAAT
TCATTGTCAGTTTCGCTTGCTTATGGACCACTTCAAGGTGGGTGTAAGCAGTCGGTTTTTAGTAGTAGAGCC
ACTTGTTGTTATGCTTATTCATATAATGGTCCTCGCGCATGTAAGGGTGTTTACGCAGGCGAGTTACTACAAAA
TTTTGAATGTGGACTGTTGGTTTATGTTACTAAGAGCGATGGCTCTCGTATACAAACAGCCACCGTTCCACCA
GTTGTAACTCAACACAATTATAATAATATTACTTTAAATACTTGTGTTGATTATAATATATATGGCAGAGTTGGTC
GAGGTTTTATTACTAATGTAACTGACTCATCATCTAGTTATAATTATTTAGCAGATGCAGGGTTGGCTATTTTAG
ATACATCAGGTGCCATAGACATCTTTGTTGTACAAGGTGAACATGGTCTTAATTATTACAAGGTTAATCCCTGT
GAAGATGTAAACCAGCAGTTTGTAGTTTCTGGTGGTAAATTAGTAGGTATTCTTACCTCACGTAATGCAACAG
GTTCTCAGCCTCTTGAGAATCAATTCTACATTAAACTCACTAAAGAGACACGTCGTTTTAGACGT
>CK_CH_GD_SG_LH-XQJ_20210222.seq
ATGTTGGGGAAGTCACTGTTTTTAGTGACCATTTTGTGTGCACTATGTAGTGCAAATTTGTTTGATCTTGCCA
ATAATTATGTGTACTACTACCAAAGTGCCTATAGGCCTCCAAATGGATGGCATTTGCAAGGGGGTGCTTATGC
AGTAGTGAATTCTACTAATTATACTAATAATGCCGATTCTGCAAGTGGGTGCACTGTTGGTATTATTAAGGACG
TCTATAATCAAAGTGCGGCTTCTATAGCTATGACAGCACCTTCTCAGGGTATGGCTTGGTCTAAGTCACAATTT
TGTAGTGCACACTGTAACTTTTCTGAAATTACAGTTTTCGTTACACATTGTTATAGTAGTGGTGCAGGGTCTTG
CCCTATAACAGGCATGATTGCACGTGATCATATTCGTATTTCTGCAATGAAAAATGGTTCTTTATTTTATAACTT
AACAGTTAGCGTATCTAAATACTCTAGGTTTAAGTCTTTTCAATGTGTTAACAACTTCACATCTGTTTATCTAAA
TGGTGATCTTGTTTTTACTTCCAACAAAACTACTGATGTTACGTCAGCAGGTGTGTATTTTAAAGCAGGTGGA
CCTGTAAATTATAGTGTTATGAAAGAATTTAAGGTTCTTGCTTATTTTGTTAATGGTACAGCACAAGATGTAAT
TTTATGTGATAACTCACCTAGAGGTTTGCTTGCATGTCAGTATAACACTGGTAATTTTTCAGATGGATTCTACC
CTTTTACTAATTCTTCTTTAGTTAAGGATAGGTTTATTGTATATCGAGAAAGTAGCACTAACACTACTTTAGAGT
TAACTAATTTCACTTTTACTAATGTAAGTAATGCTTCTCCTAATTCAGGTGGCGTTGATACTTTCCAATTATATCA
AACACATACTGCTCAGGATGGTTATTATAATTTTAATTTATCATTTCTGAGTAGTTTTGTGTATAAACCATCTGAT
TTTATGTATGGGTCATACCACCCAAATTGTAATTTTAGACCAGAGAATATTAATAATGGCTTATGGTTTAATTCA
TTATCTGTGTCACTTACTTACGGACCCATTCAAGGTGGTTGTAAGCAATCTGTTTTTAGTAATAAAGCAACTTG

TTGCTATGCTTATTCTTACCGAGGTCCTACTAGATGTAAGGGTGTTTATAGAGGGGAGCTAACGCAATACTTT
GAATGTGGACTTCTAGTTTATGTAACTAAGAGTGATGGCTCTCGTATACAAACTAGAAGTGAACCACTGGTGT
TAACTCAATATAATTATAACAACATTACTTTAAATAAGTGTGTTGAGTATAATATATATGGTAGAGTTGGTCAAG
GTTTTATTACTAATGTAACTGAAGCAACTGCTAATTATAGTTATCTAGCAGATGGTGGTTTAGCTATTTTAGATA
CTTCAGGAGCCATAGACATATTTGTTGTTCGAGGTGCATATGGTCTTAATTATTACAAGGTCAATCCTTGTGAA
GATGTTAATCAACAGTTTGTAGTGTCTGGTGGTAATATAGTTGGCATTCTTACTTCTAGAAATGAAACAGGTTC
TGAACAGGTTGAGAACCAGTTTTATGTTAAGTTAACCAATAGCTCACATCGTCGTAGGCGT
>CK_CH_YN_KM_LZY-ZYL_20211026.seq
ATGTTGGGGAAGTCACTGTTTTTAGTGATCATTTTGTGTGCACTATGTAGTGCAAATTTGTTTGATCCTGCTAA
TACTTACGTTTACTACTACCAAAGTGCCTTTAGGCCTGGTCCAGGTTGGCATTTACATGGGGGCGCTTATGCA
GTAGATAAGGTTTTTAATGAAACCAACAATGCAGGCAGTGCATCTGATTGCACTGCTGGTACTTTTTATGAAA
GCTATAATATTTCTGCTGCTTCTGTAGCCATGACAGTACCACCTGCTGGTATGTCTTGGTCAGTTTCACAGTTT
TGTACAGCTCATTGTAACTTCTCAGACTTTACAGTGTTTGTTACACATTGTTTTAAAAGTCAACAAGGTAGTT
GTCCATTGACAGGTATGATTCCTCAGAATCAAATTCGTATTTCTGCTATGAGATCTGGATTTTTGTTTTATAATT
TAACAGTTAGCGTATCTAAATACCCTAAATTTAAATCTCTTCAATGTGTTAGCAATTCTACATCTGTCTATTTAAA
TGGTGATCTTGTTTTCACTTCTAATGAAACAACTCACGTTACGGGTGCAGGCATTTATTTTAAAAGTGGTGGG
CCTGTAACTTATAAAGTTATGAAAGAAGTTAAAGCCCTAGCCTACTTTATTAATGGTACCGCACAAGAGGTTA
TTTTATGTGATAACTCACCTAGAGGTTTGCTTGCATGTCAGTATAACACTGGTAATTTTTCAGATGGATTCTAC
CCTTTTACTAATTCTTCTTTAGTTAAGGGTAGGTTTATTGTATATCGAGAAAGTAGCATTAACACTACTTTAGA
GTTAACTAATTTCACTTTTACTAATGTAAGTAATGCCTCTCCTAATTCAGGTGGCGTTGATACTTTCCAATTATAT
CAAACACATACTGCTCAGGATGGTTATTATAATTTTAATTTATCATTTCTGAGTAGTTTTGTGTATAAACCATCT
GATTTTATGTATGGGTCATACCACCCAAATTGTAATTTTAGACCAGAGAATATTAATAATGGCTTATGGTTTAAT
TCATTATCTGTGTCACTTACTTACGGACCCATTCAAGGTGGTTGTAAGCAATCTGTTTTTAGTAATAAAGCAAC
TTGTTGCTATGCTTATTCTTACCGAGGTCCTACTAGATGTAAGGGTGTTTATAGAGGGGAGCTAACGCAATAC
TTTGAATGTGGACTTCTAGTTTATGTAACTAAGAGTGATGGCTCTCGTATACAAACTAGAAGTGAACCACTGG
TGTTAACTCAATATAATTATAACAACATTACTTTAAATAAGTGTGTTGAGTATAATATATATGGTAGAGTTGGTCA
AGGTTTTATTACTAATGTAACTGAAGCAACTGCTAATTATAGTTATTTAGCAGATGGTGGTCTAGCTATTTTAGA
TACTTCAGGAGCCATAGACATATTTGTTGTTCGAGGTGCATATGGTCCTAATTATTATAAGGTTAATCCTTGTG
AAGATGTTAACCAACAGTTTGTAGTGTCTGGTGGCAATTTAGTTGGTGTTCTCACATCTCATAATGAAACAGA
TTCTGAATTTATTGAGAACCAGTTTTATGTCAAACTCACTAACGGAACACGTCGCTCTAGACGT
>CK_CH_GD_ZJZD_Gan8-27_20210105.seq
ATGTTGGGCAAACCGCTTTTACTAGTGACTCTTTGGTATGCACTATGTAGTGCTTTGCTTTATGATAAAAATAC
TTACGTTTACTACTACCAAAGTGCCTTTAGGCCTGGTCAAGGTTGGCATCTACATGGGGGTGCTTATGCAGTA
GATAAGGTTTTTAATGAAACCAACAATGCAGGCAGTGTATCTGATTGCACTGCTGGTACTTTTTATGAAAGCT
ATAATATTTCTGCTGCTTCTGTAGCCATGACAGTACCACCTGCTGGTATGTCTTGGTCAGTTGCACAGTTTTGT
ACAGCTCATTGTAACTTCTCAGACTTTACAGTGTTTGTTACGCATTGTTTTAAAAATCAACAAGGTAGTTGTCC
ATTGACAGGTATGATTCCTCAGCATCATATTCGTATTTCTGCTATGAGATCTGGACTTTTGTTTTATAATTTAAC
AGTTAGCGTATCTAAATACCCTAAATTTAAATCGCTTCAATGTGTTGGCAATTCTACATCTGTCTATTTAAATGG
TGATCTTGTTTTCACTTCTAATGAAACAACTCACGTTACGGGTGCAGGCGTTTATTTTAAAAGTGGTGGGCCT
GTAACTTATAAAGTTATGAAAGAAGTTAAAGCCCTAGCCTACTTTATTAATGGTACCGCACAAGAGGTTATTTT
ATGTGATAACTCACCTAGAGGTTTGCTTGCATGTCAGTATAACACTGGTAATTTTTCAGATGGATTCTACCCTT
TTACTAATTCTTCTTTAGTTAAGGATAGGTTTATTGTATATCGAGAAAGTAGCACTAACACTACTTTAGAGTTAA
CTAATTTCACTTTTACTAATGTAAGTAATGCTTCTCCTAATTCAGGTGGCGTTGATACTTTCCAATTATATCAAA
CACATACTGCTCAGGATGGTTATTATAATTTTAATTTATCATTTCTGAGTAGTTTTGTGTATAAACCATCTGATTT

TATGTATGGGTCATACCACCCAAATTGTAATTTTAGACCAGAGAATATTAATAATGGCTTATGGTTTAATTCATT
ATCTGTGTCACTTACTTACGGACCCATTCAAGGTGGTTGTAAGCAATCTATTTTTAGTAATAAAGCAACTTGTT
GCTATGCTTATTCTTACCAAGGTCCTACTAGATGTAAGGGTGTTTATAGAGGGGAGCTAACGCAATACTTTGA
ATGTGGACTTCTAGTTTATGTAACTAAGAGTGATGGCTCTCGTATACAAACTAGAAGTGAACCACTGGTGTTA
ACTCAATATAATTATAACAACATTACTTTAAATAAGTGTGTTGAGTATAATATATATGGTAGAGTTGGTCAAGGT
TTTATTACTAATGTAACTGAAGCAACTGCTAATTATAGTTATCTAGCAGATGGTGGTTTAGCTATTTTAGATACT
TCAGGAGCCATAGACATATTTGTTGTTCGAGGTGCATATGGTCTTAATTATTATAAGGTTAATCCCTGTGAAGA
TGTTAACCAACAGTTTGTAGTGTCTGGTGGCAATTTAGTTGGCATTCTTACATCTCATAATGAAACAGATTCTG
AATTTATTGAGAACCAGTTTTACATCAAACTCACTAATGGAACACGTCGCTCTAGACGT
>CK_CH_SD_LC_FX-AXN_20211129.seq
ATGTTGGGCAAACCGCTTTTACTAGTGACTCTTTGGTATGCACTATGTAGTGCTTTGCTTTATGATAAAAATAC
TTACGTTTACTACTACCAAAGTGCCTTTAGGCCTGGTCAAGGTTGGCATCTACATGGGGGTGCTTATGCAGTA
GATAAGGTTTTTAATGGAACCAACAATGCAGTCAGTGTATCTGATTGCACTGCTGGTACTTTTTATGAAAGCT
ATAATATTTCTGCTGCTTCTGTAGCCATGACAGTACCACCTGCTGGTATGTCTTGGTCAGTTTCACAGTTTTGT
ACAGCTCATTGTAACTTCTCAGACTTTACAGTGTTTGTTACGCATTGTTTTAAAAGTCAACAAGGTAGTTGTC
CATTGACAGGTATGATTCCTCAGAATCATATTCGTATTTCTGCTATGAGATCTGGATTTTTGTTTTATAATTTAAC
AGTTAGCGTATCTAAATACCCTAAATTTAAATCGCTTCAATGTGTTGGCAATTCTACATCTGTCTATTTAAATGG
TGATCTTGTTTTCACTTCTAATGAAACAACTCACGTTACGGGTGCAGGCGTTTATTTTAAAAGTGGTGGGCCT
GTAACTTATAAAGTTATGAAAGAAGTTAAAGCCCTAGCCTACTTTATTAATGGTACCGCACAAGAGGTTATTTT
ATGTGATAACTCACCTAGAGGTTTGCTTGCATGTCAGTATAACACTGGTAATTTTTCAGATGGATTCTACCCTT
TTACTAATTCTTCTTTAGTTAAGGATAGGTTTATTGTATATCGAGAAAGTAGCACTAACACTACTTTAGAGTTAA
CTAATTTCACTTTTACTAATGTAAGTAATGCTTCTCCTAATTCAGGTGGCGTTGATACTTTCCAATTATATCAAA
CACATACTGCTCAGGATGGTTATTATAATTTTAATTTATCATTTCTGAGTAGTTTTGTGTATAAACCATCTGATTT
TATGTATGGGTCATACCACCCAAATTGTAATTTTAGACCAGAGAATATTAATAATGGCTTATGGTTTAATTCATT
ATCTGTGTCACTTACTTACGGACCCATTCAAGGTGGTTGTAAGCAATCTGTTTTTAGTAATAAAGCAACTTGTT
GCTATGCTTATTCTTACCGAGGTCCTACTAGATGTAAGGGTGTTTATAGAGGGGAGCTAACGCAATACTTTGA
ATGTGGACTTCTAGTTTATGTAACTAAGAGTGATGGCTCTCGTATACAAACTAGAAGTGAACCACTGGTGTTA
ACTCAATATAATTATAACAACATTACTTTAAATAAGTGTGTTGAGTATAATATATATGGTAGAGTTGGTCAAGGT
TTTATTACTAATGTAACTGAAGCAACTGCTAATTATAGTTATCTAGCAGATGGTGGTTTAGCTATTTTAGATACT
TCAGGAGCCATAGACATATTTGTTGTTCGAGGTGCATATGGTCTTAATTATTATAAGGTTAATCCCTGTGAAGA
TGTTAATCAACAATTTGTAGTGTCTGGTGGCAATATAGTTGGCATTCTTACTTCTAGAAATGAAACAGGTTCTG
AACAGGTTGAGAACCAGTTTTATGTTAAGTTAACCAATAGCTCACATCGTCGTAGGCGT
>CK_CH_CQ_BS_DK-LWC_20210913.seq
ATGTTGGGGAAGTCACTGTTGTTAGTGACCATTTTGTGTGCACTATGTAGTGCAAATTTGTTTGATTCTGGTA
ATTATGTGTACTATTACCAAAGTCAATTTAGGCCTTCAGGTGGATGGCATGTGCACGGGGGCGCCTATGCAGT
AGTGAATTCTACTTCTAAAAGCAACAATGCAGGCAATGCTAATGAGTGTTCTGTAGGTGTTCTTTTTAATTATA
GTAACGGAAATGACGTTGGATATAATAATAGTGCTGCTTCCATAGCCATGACAGCACCGCCTAGTGGTATGTC
TTGGTCTAAATCAGAATTTTGTACTGCCCACTGTAACTTTTCGGATTTTACAGTGTTTGTTACACATTGTTATGC
ACAATCTTGCCCTTTAACAGGTAAGATAGAGCAGAACCACATTCGTATTTCTGCTATGAGAAATGGTTCTCTAT
TTTATAATTTAACAGTTAGTACACTTAAGTACCCTAACTTTAAATCGTTTCAATGTGTTAACAACTTCACTTCTG
TTTATTTAAATGGTGATCTTGTTTTTACTTCTAATGAAACCACTGATGTTAAAGGTGCTGGTGTGTATTTTAAA
GCAGGTGGGCCTATATCCTATAAAGTTATGAAAGAATTTAGGGTTCTTGCTTACTTTGTTAATGGTACAGCAC
AAGATGTAATTTTGTGCGACAAGTCCCCCAAGGGTTTGCTAGCTTGTCAATATAACACTGGCAATTTTTCAGA
TGGCTTTTATCCTTTTACTAATACTACTTTAGTTAGGGAAAAATTCATCGTACATCGCGAAAGTAGTGTTAATAC

TACTCTGGCGTTAACTAATCTCACTTTTAATAATGAAAGTAATGCACAGCCTAATAGTGGTGGTGTTAATACTT
TTCATCTATATCAAACACAAACAGCTCAGAGTGGTTATTATAATTTTGATTTGTCATTTCTGAGTCAGTTTGTGT
ATAAGGCAAGTGATTTTATGTATGGGTCGTACCACCCTAGTTGTTCTTTTAGACCAGAAACCATTAATAGTGG
TTTGTGGTTTAATTCTTTGTCAGTTTCTCTAGCTTACGGACCACTTCAAGGTGGGTGTAAGCAGTCAGTTTTT
AGTGGTAGGGCAACGTGTTGCTATGCTTATTCTTACAATGGCCCGATAGCCTGTAAAGGTGTCTATTCAGGCG
AGTTAATGACTAATTTTGAATGTGGATTGCTGATTTATGTTACTAAGAGTGATGGCTCTCGTATACAGACTAGA
ACAGAGCCCTTAGTATTAACGCAACACAATTATAATAATATTACTTTAGATAAGTGTGTTGACTATAATATATATG
GCAGAGTAGGCCAAGGTTTTATTACTAATGTGACTGATTCTGCTGCTAATTTTGGTTATTTAGCAGATGGTGG
GTTAGCTATTTTAGATACTTCGGGTGCCATAGATGTCTTTGTTGTACAGGGCAGCTATGGTCTTAATTATTACA
AGGTCAATCCTTGTGAAGATGTTAATCAACAGTTTGTAGTGTCTGGTGGCAATATAGTCGGCATTCTTACTTCT
AGAAATGAAACAGGTTCTGAACAGGTTGAGAACCAGTTTTATGTTAAATTAACCAATAGCCCACATCGTCGT
AGGCGT
>CK_CH_GD_ZJZD_Hun22_20210105.seq
ATGTTGGGGAAGTCACTGTTTTTAGTGACCATTTTGTGTGCACTATGTAGTGCAAATTTGTTTGATTTTGCCA
ATAATTATGTGTACTACTACCAAAGTGCCTTTAGGCCTCCAAATGGATGGCATCTGCAAGGGGGTGCTTATGC
AGTAGTGAATTCTACTAATTATACCGGTTCTGCAAGTGAGTGCACTCTTGGTGTTATTAAGGACGTCTATAATC
AAAGTGCGGCTTCCATAGCTATGACAGCACCTCCTCAGGGTATGGCTTGGTCTAAGGCACAATTTTGTAGTG
CACACTGTAACTTTTCTGAAATTACAGTTTTTGTCACACATTGTTATATTAGTGGTGCAGATTCTTGTCCTATAA
CAGGCATGATTGAACGTGGTTATATTCGTATTTCTGCAATGAAAAATGGTTCTTTATTTTATAATTTAACAGTTA
GCGTATCTAAATACCCTACGTTTAAATCTTTTCAATGTGTTAACAATTTCACATCTGTTTATCTAAATGGTGATCT
TGTTTTTACTTCTAACAAAACTGCTGATGTTACGTCAGCAGGTGTGTATTTTAAAGCAGGTGGACCCGTAAAT
TATAGTGTTATGAAAGAATTTAAGGTTCTTGCTTACTTTGTTAATGGTACAGCACAAGATGTAATTTTGTGCGA
CAATTCCCCCAAGGGTTTGCTGGCTTGTCAATATAATACTGGCAATTTTTCAGATGGCTTTTATCCTTTTACTAA
TAGTACTTTAGTTAGGGACAAGTTCACTGTCTATCGTGAAAGTAGTGTCAATACTACTTTGACGTTAACTAATT
TCACTTTTACTAATGTAAGTACTGCACAGCCTAATAGTGGTGGTGTTAGTACTTTTCATTTATATCAAACACAA
ACAGCTCAGAGTGGTTATTATAATTTTAATTTGTCATTTCTGAGTCAGTTTGTGTATAAGGCAAGTGATTTTAT
GTATGGGTCTTACCACCCTAGGTGTTCTTTTAGACCAGAAACCATTAATAGTGGTTTATGGTTTAATTACTTGT
CAGTTTCCCTTACTTATGGACCCCTACAGGGAGGGTGTAAGCAATCTGTTTTTAATGGTAAGGCAACGTGTT
GTTATGCCTACTCTTATAATGGCCCAAGGGCATGTAAAGGTGTTTATCCAGGTGAATTAAGCAAGACTTTTGA
ATGTGGATTGCTGGTTTATGTTACTAAGCGTGATGGCTCTCGTCTACAGACTAGAACGGAGCCCTTAGTATTA
ATGCAACACAATTATAATAATATTACTTTAGATAAGTGTGTTGACTATAATATATATGGCAGAGTAGGCCAAGGT
TTTATTACTAATGTGACTGATTCTGCTGCTAATTTTAGTTATTTAGCAGATGGTGGGTTAGCTATTTTAGATACT
TCGGGTGCCATAGATGTCTTTGTTGTACAGGGCAGCTATGGTCTTAATTATTACAAGGTCAATCCTTGTGACG
ATGTTAACCAACAGTTTGTAGTGTCTGGTGGCAATATAGTTGGCATTCTTACTTCTAGAAATGAAACAGGTTC
TGAACAGGTTGAGAACCAGTTTTATGTTAAGTTAACCAATAGCTCACATCGTCACAGGCGT
>CK_CH_JS_LH-HQX_20210324.seq
ATGTTGGGGAAGTCACTGTTTTTAGTGACCATTTTGTGTGCACTATGTAGTGCAAATTTGTTTGATCCTGCCA
ATAATTATGTGTACTACTACCAAAGTGCCTTTAGGCCTCCAACTGGATGGCATTTGCAAGGGGGTGCTTATGC
AGTAGTGAATTCTACTAATTATACTAGTAATGCCGGTTCTGCAAGTGAGTGCACTGTTGGTATTATTAAGGACG
TCTATAATCAAAGTGCGGCTTCCATAGCTATGACAGCACCTTCTCAGGGTATGGCTTGGTCTAAGTCACAATT
TTGTAGTGCACACTGTAACTTTTCTGAAATTACAGTTTTCGTCACACATTGTTATAGTAGTGGTACAGGGTCTT
GCCCTATAACAGGCATGATTGCACGTGATCATATTCGTATTTCTGCAATGAAAAATGGTTCTTTATTTTATAACT
TAACAGTTAGCGTATCTAAATACTCTAGGTTTAAGTCTTTTCAATGTGTTAACAACTTCACATCTGTTTATCTAA
ATGGTGATCTTGTTTTTACTTCCAACAAAACTACTGATGTTACGTCAGCAGGTGTGTATTTTAAAGCAGGTGG

ACCTGTAAATTATAGTATTATGAAAGAATTTAAGGTTCTTGCTTATTTTGTTAATGGTACAGCACAAGATGTAAT
TTTGTGTGACAAGTCCCCCAAGGGTTTGCTAGCTTGTCAATATAACACTGGCAATTTTTCAGATGGCTTTTAT
CCTTTTACTAATACTACTTTAGTTAGGGAAAAGTTCATCGTATATCGCGAAAGTAGTGTTAATACTACTCTGGC
GTTAACTAATCTTACTTTTTCTAATGTGAGTAATGCACAGCCTAATAGTGGTGGTGTTAATACTTTTCATTTATAT
CAAACACAAACAGCTCAGAGTGGTTATTATAATTTTAATTTGTCATTTCTGAGTCAGTTTGTGTATAAGGCAA
GTGATTTTATGTATGGGTCTTATCACCCTAGTTGTTCTTTCAGACCAGAAACCATTAATAGTGGTTTGTGGTTT
AATTCCTTGTCAGTTTCTCTAGCTTATGGACCACTTCAAGGTGGGTGTAAGCAGTCAGTTTTTAGTGGTAGG
GCAACGTGTTGCTATGCCTACTCTTACAATGGCCCGGTAGCCTGTAAAGGTGTTTATGCAGGCGAATTACAG
ACTAATTTTGAATGTGGATTGCTGATTTATGTTACTAAGAGTGATGGCTCTCGTATACAGACTAGAACAGAGC
CCTTAGTATTAACGCAACACAATTATAATAATATTACTTTAGATAAGTGTGTTGACTATAATATATATGGCAGAGT
AGGCCAAGGTTTTATTACTAATGTGACTGATTCTGCTGCTAATTTTAGTTATTTAGCAGATGGTGGGTTAGCTA
TTTTAGATACCTCCGGTGCCATAGATGTTTTTGTTGTACAGGGCAGCTATGGTCTTAATTATTACAAGGTCAAT
CCTTGTGAAGATGTTAACCAACAGTTTGTAGTGTCTGGTGGCAATATAGTTGGTATTCTTACTTCTAGAAATG
AAACAGGTTCTGAACAGGTTGAGAACCAGTTTTATGTTAAGTTAACCAATAGCTCACATCGTCGTAGGCGT
>CK_CH_GD_SG_LH-CZZ_20210310.seq
ATGTTGGGGAAGTCACTGTTTTTAGTGACCATTTTGTGTGCACTATGTAGTGCAAATTTGTTTGATCCTGCCA
ATAATTATGTGTACTACTACCAAAGTGCCTTTAGGCCTCCAACTGGATGGCATTTGCAAGGGGGTGCTTATGC
AGTAGTGAATTCTACTAATTATACTAGTAATGCCGGTTCTGCAAGTGAGTGCACTGTTGGTATTATTAAGGACG
TCTATAATCAAAGTGCGGCTTCCATAGCTATGACAGCACCTTCTCAGGGTATGGCTTGGTCTAAGTCACAATT
TTGTAGTGCACACTGTAACTTTTCTGAAATTACAGTTTTCGTCACACATTGTTATAGTAGTGGTACAGGGTCTT
GCCCTATAACAGGCATGATTGCACGTGATCATATTCGTATTTCTGCAATGAAAAATGGTTCTTTATTTTATAACT
TAACAGTTAGCGTATCTAAATACTCTAGGTTTAAGTCTTTTCAATGTGTTAACAACTTCACATCTGTTTATCTAA
ATGGTGATCTTGTTTTTACTTCCAACAAAACTACTGATGTTACGTCAGCAGGTGTGTATTTTAAAGCAGGTGG
ACCTGTAAATTATAGTATTATGAAAGAATTTAAGGTTCTTGCTTATTTTGTTAATGGTACAGCACAAGATGTAAT
TTTGTGTGACAAGTCCCCCAAGGGTTTGCTAGCTTGTCAATATAACACTGGCAATTTTTCAGATGGCTTTTAT
CCTTTTACTAATACTACTTTAGTTAGGGAAAAGTTCATCGTATATCGCGAAAGTAGTGTTAATACTACTCTGGC
GTTAACTAATCTTACTTTTTCTAATGTGAGTAATGCACAGCCTAATAGTGGTGGTGTTAATACTTTTCATTTATAT
CAAACACAAACAGCTCAGAGTGGTTATTATAATTTTAATTTGTCATTTCTGAGTCAGTTTGTGTATAAGGCAA
GTGATTTTATGTATGGGTCTTATCACCCTAGTTGTTCTTTCAGACCAGAAACCATTAATAGTGGTTTGTGGTTT
AATTCCTTGTCAGTTTCTCTAGCTTATGGACCACTTCAAGGTGGGTGTAAGCAGTCAGTTTTTAGTGGTAGG
GCAACGTGTTGCTATGCCTACTCTTACAATGGCCCGGTAGCCTGTAAAGGTGTTTATGCAGGCGAATTACGG
ACTAATTTTGAATGTGGATTGCTGATTTATGTTACTAAGAGTGATGGCTCTCGTATACAGACTAGAACAGAGC
CCTTAGTATTAACGCAACACAATTATAATAATATTACTTTAGATAAGTGTGTTGACTATAATATATATGGCAGAGT
AGGCCAAGGTTTTATTACTAATGTGACTGATTCTGCTGCTAATTTTAGTTATTTAGCAGATGGTGGGTTAGCTA
TTTTAGATACCTCCGGTGCCATAGATGTTTTTGTTGTACAGGGCAGCTATGGTCTTAATTATTACAAGGTCAAT
CCTTGTGAAGATGTTAACCAACAGTTTGTAGTGTCTGGTGGCAATATAGTTGGTATTCTTACTTCTAGAAATG
AAACAGGTTCTGAACAGGTTGAGAACCAGTTTTATGTTAAGTTAACCAATAGCTCACATCGTCGTAGGCGT
>CK_CH_ZJ_TZ_LH-YJW_20210928.seq
ATGTTGGGGAAGTCACTGTTTTTAGTGACCATTTTGTGTGCACTATGTAGTGCAAATTTATTCGATCCTGCTAA
TACTTATGTGTACTACTACCAAAGTGCCTTTAGGCCTCCAAATGGATGGCACCTACAAGGGGGTGCTTATGCA
GTAGTCAATTCCACTAATTATACTAATAATGCCGGTTCTGCACAACATTGCACTGTTGGTGTTATTAAGGACGT
CTATAATCAAAGTGCGGCTTCCATAGCTATGACAGCACCTCTTCAGGGTATGGCTTGGTCTAAGTCACAATTT
TGTAGTGCACACTGTAACTTTTCTGAAATTACAGTTTTTGTCACACATTGTTATAGTAGTGGTAGCGGGTCTTG
TCCTATAACAGGCATGATTGCACGTGATCATATTCGTATTTCTGCAATGAAAAATGGTACTTTATTTTATAATTTA

ACAGTTAGCGTATCTAAATACCCTAATTTTAAATCTTTTCAATGCGTTAATAATTTCACATCTGTTTATCTAAATG
GTGATCTTGTTTTTACTTCCAACAAAACTACTGATGTTACGTCAGCAGGTGTGTATTTTAAAGCAGGTGGACC
TGTAAATTATAGTATTATGAAAGAATTTAAGGTTCTTGCTTACTTTGTTAATGGTACAGCACAAGATGTAATTTT
GTGCGACAATTCCCCCAAGGGTTTGCTAGCTTGTCAATATAACACTGGCAATTTTTCAGATGGCTTTTATCCTT
TTACTAATAGTACTTTAGTTAGGGAAAAGTTCATCGTATATCGCGAAAGTAGTGTTAATACTACTCTGGCGTTA
ACTAATTTCACTTTTACTAATGTAAGTAATGCACAGCCTAATAGTGGTGGTGTTAATACTTTTCATCTATATCAA
ACACAAACAGCTCAGAGTGGTTATTATAATTTTAATTTGTCATTTCTGAGTCAGTTTGTGTATAAGGCAAGTGA
TTTTATGTATGGGTCCTACCACCCTAGTTGTTCTTTTAGACCAGAAACCATTAATAGTGGTTTGTGGTTTAATT
CTTTGTCAGTTTCTCTAGCTTACGGACCACTTCAAGGTGGGTGTAAGCAGTCAGTTTTTAGTGGTAGGGCAA
CGTGTTGCTATGCCTACTCTTACAATGGCCCGATAGCCTGTAAAGGTGTTTATTCAGGCGAATTACGGACTAA
TTTTGAATGTGGATTGCTGATTTATGTTACTAAGAGTGATGGTTCTCGTATACAGACTAGAACAGAGCCCTTA
GTATTAACGCAACACAATTATAATAATATTACTTTAGATAAGTGTGTTGACTATAATATATATGGCAGAGTAGGC
CAAGGTTTTATTACTAATGTGACTGATTCTGCTGCTAATTTTAGTTATTTAGCAGATGGTGGGTTAGCTATTTTA
GATACTTCGGGTGCCATAGATGTCTTTGTTGTACAGGGCAGCTATGGTCTTAATTATTACAAGGTCAATCCTTG
TGAAGATGTTAACAAACAGTTTGTAGTGTCTGGTGGCAATATAGTTGGCATTCTTACTTCTAGAAATGAAACA
GGTTCTGAACAGGTTGAGAACCAGTTTTATGTTAAGTTAACCAATAGCTCACATCGTCGCAGGCGT
>CK_CH_GD_QY_TN-YMC-C_20220221.seq
ATGTTGGGGAAGTCACTGTTTTTAGTGACCATTTTGTGTGCACTATGTAGTGCAAATTTGTTTGATTTGGCCA
ATAATTATGTGTACTACTACCAAAGTGCCTTTAGGCCTCCAAATGGATGGCATTTGCAAGGGGGTGCTTATGC
AGTAGTGAATTCTACTAATTATACTAGTAATGCCGGTTCTGCAAGTGAGTGCACTGTTGGTATTATTAAGGATG
TCTATAATCAAAGTGCGGCTTCCATAGCTATGACAGCACCTTTTCAGGGTATGGCTTGGTCTAAGTCACAATT
TTGTAGTGCACACTGTAACTTTTCTGAAATTACAGTTTTCGTCACACATTGTTATAGTAGTGGTACAGGGTCTT
GCCCTATAACAGGCATGATTGCACGTGATCATATTCGTATTTCTGCAATGAAAAATGGTTCTTTATTTTATAACT
TAACAGTTAGCGTATCTAAATACTCTAGGTTTAAGTCTTTTCAATGTGTTAACAACCTCACATCTGTTTATCTAA
ATGGTGATCTTGTTTTTACTTCCAACAAAACTACTGATGTTACGTCAGCAGGTGTGTATTTTAAAGCAGGTGG
ACCTGTAAATTATAGTGTTATGAAAGAATTTAAGGTTCTTGCTTATTTTGTTAATGGTACAGCACAAGATGTAA
TTTTGTGTGACAAGTCCCCCAAGGGTTTGCTAGCTTGTCAATATAACACTGGCAATTTTTCAGATGGCTTTTAT
CCTTTTACTAATACTACTTTAGTTAGGGAAAAGTTCATTGTATATCGTGAAAGTAGTGTTAATACTACTCTGGC
GTTAACTAATTTCACTTTTACTAATGTAAGTAATGCACAGCCTAATAGTGGTGGTGTTAATACTTTTCATTTATAT
CAAACACAAACAGCTCAGAGTGGTTATTATAATTTTAATTTGTCATTTCTGAGTCAGTTTGTGTATAAGGCAA
GTGATTTTATGTATGGGTCTTATTATCCTAGGTGTTCTTTTAGACCAGAAACCATTAATAATGGTTTGTGGTTTA
ATTCCTTGTCAGTTTCTCTTACTTATGGACCCCTACAGGGAGGGTGTAAGCAATCTGTTTTTAGTGGTAAGGC
AACGTGTTGTTATGCCTACTCTTATAATGGCCCTAGAGCATGCAAAGGTGTTTATTCAGGCGAATTAAGCAAG
ACTTTTGAATGTGGATTGCTGGTTTATGTTACTAAGAGTGATGGCTCTCGTATACAAACTAGAACGGAGCCCT
TAGTATTAACGCAACACAATTATAATAATATTACTTTAGATAAGTGTGTTAACTATAATATATATGGCAGAGTTGG
CCAAGGTTTTATTACTAATGTGACTGATTCTGCTGCTAATTTTAGTTATTTAGCAGATGGTGGGTTAGCTATTTT
AGACACTTCGGGTGCCATAGATGTTTTTGTTGTACAGGGCAGCTATGGTCCTAATTATTACAAGGTCAATCCT
TGTGAAGATGTTAACCAACAGTTTGTAGTGTCTGGTGGCAATATAGTTGGCATTCTTACTTCTAGAAATGAAA
CAGGTTCTGAACAGGTTGAGAACCAGTTTTATGTTAAGTTAACCAATAGCTCACATCGTCGTAGGCGT
>CK_CH_SD_LY_LWK-1#_20220310.seq
ATGTTGGGGAAGTCACTGTTTTTAGTGACCATTTTGTGTGCACTATGTAGTGCAAATTTGTTTGATTTGGCCA
ATAATTATGTGTACTACTACCAAAGTGCCTTTAGGCCTCCAAATGGATGGCATTTGCAAGGGGGTGCTTATGC
AGTAGTGAATTCTACTAATTATACTAGTAATGCCGGTTCTGCAAGTCAGTGCACTATTGGTATTATTAAGGACG
TCTATAATCAAAGTGCGGCTTCCATAGCTATGACAGCACCTTCTCAGGGTATGGCTTGGTCTAAGTCACAATT

TTGTAGTGCACACTGTAACTTTTCTGAAATTACAGTTTTCGTCACACATTGTTATAGTAGTGGTACAGGGTCTT
GCCCTATAACAGGCCTGATTGCACGTGATCATATTCGTATTTCTGCAATGAAAAATGGTTCTTTATTTTATAACT
TAACAGTTAGCGTATCTAAATACTCTAGGTTTAAGTCTTTTCAATGTGTTAACAACCTCACATCTGTTTATCTAA
ATGGTGATCTTGTTTTTACTTCCAACAAAACTACTGATGTTACGTCAGCAGGTGTGTATTTTAAAGCAGGTGG
ACCTGTAAATTATAGTGTTATGAAAGAATTTAAGGTTCTTGCTTATTTTGTTAATGGTACAGCACAAGATGTAA
TTTTGTGTGACAAGTCCCCCAAGGGTTTGCTAGCTTGTCAATATAACACTGGCAATTTTTCAGATGGCTTTTAT
CCTTTTACTAATACTACTTTAGTTAGGGAAAAGTTCATTGTATATCGTGAAAGTAGTGTTAATACTACTCTGGC
GTTAACTAATTTCACTTTTACTAATGTAAGTAATGCACAGCCTAATAGTGGTGGTGTTAATACTTTTCATTTATAT
CAAACACAAACAGCTCAGAGTGGTTATTATAATTTTAATTTGTCATTTCTGAGTCAGTTTGTGTATAAGGCAA
GTGATTTTATGTATGGGTCTTATTATCCTAGGTGTTCTTTTAGACCAGAAACCATTAATAATGGTTTGTGGTTTA
ATTCCTTGTCAGTTTCTCTTACTTATGGACCCCTACAGGGAGGGTGTAAGCAATCTGTTTTTAGTGGTAAGGC
AACGTGTTGTTATGCCTACTCTTATAATGGCCCTAGAGCATGCAAAGGTGTTTATTCAGGCGAATTAAGCAAG
ACTTTTGAATGTGGATTGCTGGTTTATGTTACTAAGAGTGATGGCTCTCGTATACAAACTAGAACGGAGCCCT
TAGTATTAACGCAACACAATTATAATAATATTACTTTAGATAAGTGTGTTAACTATAATATATATGGCAGAGTTGG
CCAAGGTTTTATTACTAATGTGACTGATTCTGCTGCTAATTTTAGTTATTTAGCAGATGGTGGGTTAGCTATTTT
AGACACTTCGGGTGCCATAGATGTTTTTGTTGTACAGGGCAGCTATGGTCCTAATTATTACAAGGTCAATCCT
TGTGAAGATGTTAACCAACAGTTTGTAGTGTCTGGTGGCAATATAGTTGGCATTCTTACTTCTAGAAATGAAA
CAGGTTCTGAACAGGTTGAGAACCAGTTTTATGTTAAGTTAACCAATAGCTCACATCGTCGTAGGCGT
>CK_CH_SD_XXW-8_20220114.seq
ATGTTGGGGAAGTCACTGTTTTTAGTGACCATTTTGTGTGCACTATGTAGTGCAAATTTGTTTGATTTTGCCA
ATAATTATGTGTACTACTACCAAAGTGCCTTTAGGCCTCCAAATGGATGGCATTTGCAAGGGGGTGCTTATGC
AGTAGTGAATTCTACTAATTATACTAGTAATGCCGGTTCTGCAAGTGAGTGCACTGTTGGTATTATTAAGGACG
TCTATAATCAAAGTGCGGCTTCCATAGCTATGACAGCACCCTCTCAGGGTATGGCTTGGTCTAAGTCACAATT
TTGTAGTGCACACTGTAACTTTTCTGAAATTACAGTTTTCGTCACACATTGTTATAGTAGTGGTACAGGGTCTT
GCCCTATAACAGGCATGATTGCACGTGATCATATTCGTATTTCTGCAATGAAAAATGGTTCTTTATTTTATAACT
TAACAGTTAGCGTATCTAAATACTCTAGGTTTAAGTCTTTTCAATGTGTTAACAACTTCACATCTGTCTATCTAA
ATGGTGATCTTGTTTTTACTTCCAACAAAACTACTGATGTTACGTCAGCAGGTGTGTATTTTAAAGCAGGTGG
ACCTGTAAATTATAGTGTTATGAAAGAATTTAAGGTTCTTGCTTATTTTGTTAATGGTACAGCACAAGATGTAA
TTTTGTGTGACAAGTCCCCCAAGGGTTTGCTAGCTTGTCAATATAACACTGGCAATTTTTCAGATGGCTTTTAT
CCTTTTACTAATACTACTTTAGTTAGGGAAAAGTTCATCGTATATCGTGAAAGTAGTGTTAATACTACTCTGGC
GTTAACTAATTTCACTTTTACTAATGTAAGTAATGCACAGCCTAATAGTGGTGGTGTTAATACTTTTCATTTATAT
CAAACACAAACAGCTCAGAGTGGTTATTATAATTTTAATTTCTCATTTCTGAGTCAGTTTGTGTATAAGGCAAG
TGATTTTATGTATGGGTCTTATCATCCTAGGTGTTCTTTTAGACCAGAAACCATTAATAATGATTTGTGGTTTAA
TTCCTTGTCAGTTTCTCTTACTTATGGACCCCTACAGGGAGGGTGTAAGCAATCTGTTTTTAGTGGTAAGGCA
ACGTGTTGTTATGCCTACTCTTATAATGGCCCTAGAGTATGTAAAGGTGTTTATTCAGGTGAATTAAGCAAGAC
TTTTGAATGTGGATTGCTGGTTTATGTTACTAAGAGTGATGGCTCTCGTATACAGACTAGAACAGAGCCCTTA
GTATTAACGCAACACAATTATAATAATATTACTTTAGATAAGTGTGTTAACTATAATATATATGGCAGAGTGGGC
CAAGGTTTTATTACTAATGTGACTGATTCTGCTGCTAATTTTAGTTATTTAGCAGATGGTGGGTTAGCTATTTTA
GACACTTCGGGTGCCATAGATGTTTTTGTTGCACAGGGCAGCTATGGTCCTAATTATTACAAGGTCAATCCTT
GTGAAGATGTTAACCAACAGTTTGTAGTGTCTGGTGGCAATATAGTTGGCTTTCTTACTTCTAGAAATGAAAC
AGGTTCTGAACAGGTTGAGAACCAGTTTTATGTTAAGTTAACCAATAGCTCACATCGTCGTAGGCGC
>CK_CH_GD_ZQ_SHHF-6_20221229.seq
ATGTTGGGGAAGTCACTGTTTTTAGTGACCATTTTGTGTGCACTATGTAGTGCAAATTTGTTTGATTTTGCCA
ATAATTATGTGTACTACTACCAAAGTGCCTTTAGGCCTCCAAATGGATGGCATTTGCAAGGGGGTGCTTATGC

AGTAGTGAATTCTACTAATTATACTAGTAATGCCGGTTCTGCAAGTGATTGCACTATTGGTATTATTAAGGACG
TCTATAATCAAAGTGCGGCTTCCATAGCTATGACAGCACCCTCTCAGGGTATGGCTTGGTCTAAGTCACAATT
TTGTAGTGCACACTGTAACTTTTCTGAAATTACAGTTTTCGTCACACATTGTTATAGTAGTGGTACAGGGTCTT
GCCCTATAACAGGCATGATTGCACGTGATCATATTCGTATTTCTGCAATGAAAAATGGTTCTTTATTTTATAACT
TAACAGTTAGCGTATCTAAATACTCTAGGTTTAAGTCTTTTCAATGTGTTAACAACTTCACATCTGTCTATCTAA
ATGGTGATCTTGTTTTTACTTCCAACAAAACTACTGATGTTACGTCAGCAGGTGTGTATTTTAAAGCAGGTGG
ACCTGTAAATTATAGTGTTATGAAAGAATTTAAGGTTCTTGCTTATTTTGTTAATGGTACAGCACAAGATGTAA
TTTTGTGTGACAAGTCCCCCAAGGGTTTGCTAGCTTGTCAATATAACACTGGCAATTTTTCAGATGGCTTTTAT
CCTTTTACTAATACTACTTTAGTTAGGGAAAAGTTCATCGTATATCGTGAAAGTAGTGTTAATACTACTCTGGC
GTTAACTAATTTCACTTTTACTAATGTAAGTAATGCACAGCCTAATAGTGGTGGTGTTAATACTTTTCATTTATAT
CAAACACAAACAGCTCAGAGTGGTTATTATAATTTTAATTTCTCATTTCTGAGTCATTTTGTGTATAAGGCAAG
TGATTTTATGTATGGGTCTTATCATCCTAGGTGTTCTTTTAGACCAGAAACCATTAATAATGATTTGTGGTTTAA
TTCCTTGTCAGTTTCTCTTACTTATGGACCCCTACAGGGAGGGTGTAAGCAATCTGTTTTTAGTGGTAAGGCA
ACGTGTTGTTATGCCTACTCTTATAATGGCCCTAGAGTATGTAAAGGTGTTTATTCAGGTGAATTAAGCAAGAC
TTTTGAATGTGGATTGCTGGTTTATGTTACTAAGAGTGATGGCTCTCGTATACAGACTAGAACAGAGCCCTTA
GTATTAACGCAACACAATTATAATAATATTACTTTAGATAAGTGTGTTAACTATAATATATATGGCAGAGTGGGC
CAAGGTTTTATTACTAATGTGACTGATTCTGCTGCTAATTTTAGTTATTTAGCAGATGGTGGGTTAGCTATTTTA
GACACTTCGGGTGCCATAGATGTTTTTGTTGCACAGGGCAGCTATGGTCCTAATTATTACAAGGTTAATCCTT
GTGAAGATGTTAACCAACAGTTTGTAGTGTCTGGTGGCAATATAGTTGGCATTCTTACTTCTAGAAATGAAAC
AGGTTCTGAACAGGTTGAGAACCAGTTTTATGTTAAGTTAACCAATAGCTCACATCGTCGTAGGCGC
>CK_CH_SD_XXW-2_20220114.seq
ATGTTGGGGAAGTCACTGTTTTTAGTGACCATTTTGTGTGCACTATGTAGTGCAAATTTGTTTGATTTTGCCA
ATAATTATGTGTACTACTACCAAAGTGCCTTTAGGCCTCCAAATGGATGGCATTTGCAAGGGGGTGCTTATGC
AGTAGTGAATTCTACTAATTATACTAGTAATGCCGGTTCTGCAAGTGAGTGCACTGTTGGTATTATTAAGGACG
TCTATAATCAAAGTGCGGCTTCCATAGCTATGACAGCACCCTCTCAGGGTATGGCTTGGTCTAAGTCACAATT
TTGTAGTGCACACTGTAACTTTTCTGAAATTACAGTTTTCGTCACACATTGTTATAGTAGTGGTACAGGGTCTT
GCCCTATAACAGGCATGATTGCACGTGATCATATTCGTATTTCTGCAATGAAAAATGGTTCTTTATTTTATAACT
TAACAGTTAGCGTATCTAAATACTCTAGGTTTAAGTCTTTTCAATGTGTTAACAACTTCACATCTGTCTATCTAA
ATGGTGATCTTGTTTTTACTTCCAACAAAACTACTGATGTTACGTCAGCAGGTGTGTATTTTAAAGCAGGTGG
ACCTGTAAATTATAGTGTTATGAAAGAATTTAAGGTTCTTGCTTATTTTGTTAATGGTACAGCACAAGATGTAA
TTTTGTGTGACAAGTCCCCCAAGGGTTTGCTAGCTTGTCAATATAACACTGGCAATTTTTCAGATGGCTTTTAT
CCTTTTACTAATACTACTTTAGTTAGGGAAAAGTTCATCGTATATCGTGAAAGTAGTGTTAATACTACTCTGGC
GTTAACTAATTTCACTTTTACTAATGTAAGTAATGCACAGCCTAATAGTGGTGGTGTTAATACTTTTCATTTATAT
CAAACACAAACAGCTCAGAGTGGTTATTATAATTTTAATTTCTCATTTCTGAGTCAGTTTGTGTATAAGGCAAG
TGATTTTATGTATGGGTCTTATCATCCTAGGTGTTCTTTTAGACCAGAAACCATTAATAATGATTTGTGGTTTAA
TTCCTTGTCAGTTTCTCTTACTTATGGACCCCTACAGGGAGGGTGTAAGCAATCTGTTTTTAGTGGTAAGGCA
ACGTGTTGTTATGCCTACTCTTATAATGGCCCTAGAGTATGTAAAGGTGTTTATTCAGGTGAATTAAGCAAGAC
TTTTGAATGTGGATTGCTGGTTTATGTTACTAAGAGTGATGGCTCTCGTATACAGACTAGAACAGAGCCCTTA
GTATTAACGCAACACAATTATAATAATATTACTTTAGATAAGTGTGTTAACTATAATATATATGGCAGAGTGGGC
CAAGGTTTTATTACTAATGTGACTGATTCTGCTGCTAATTTTAGTTATTTAGCAGATGGTGGGTTAGCTATTTTA
GACACTTCGGGTGCCATAGATGTTTTTGTTGCACAGGGCAGCTATGGTCCTAATTATTACAAGGTCAATCCTT
GTGAAGATGTTAACCAACAGTTTGTAGTGTCTGGTGGCAATATAGTTGGCTTTCTTACTTCTAGAAATGAAAC
AGGTTCTGAACAGGTTGAGAACCAGTTTTATGTTAAGTTAACCAATAGCTCACATCGTCGTAGGCGC
>CK_CH_AH_FY_LH-LDD_20220316.seq

ATGTTGGGGAAGTCACTGTTTTTAGTGACCATTTTGTGTGCACTATGTAGTGCAAATTTGTTTGATCCTGCCA
ATAATTATGTGTACTACTACCAAAGTGCCTTTAGGCCTCCAAATGGATGGCATTTTCAAGGGGGTGCTTATGC
AGTAGTGAATTCTACTAATTATACTAGTAATGCCGGTTCTGCAAGTGAGTGCACTGTTGGTATTATTAAGGACG
TCTATAATCAAAGTGCGGCTTCCATAGCTATGACAGCACCCTCTCAGGGTATGGCTTGGTCTAAGTCACAATT
TTGTAGTGCACACTGTAACTTTTCTGAAATTACAGTTTTCGTCACACATTGTTATAGTAGTGGTACAGGGTCTT
GCCCTATAACAGGCATGATTGCACGTGATCATATTCGTATTTCTGCAATGAAAAATGGTTCTTTATTTTATAACT
TAACAGTTAGCGTATCTAAATACTCTAGGTTTAAGTCTTTTCAATGTGTTAACAACTTCACATCTGTCTATCTAA
ATGGTGATCTTGTTTTTACTTCCAACAAAACTACTGATGTTACGTCAGCAGGTGTGTATTTTAAAGCAGGTGG
ACCTGTAAATTATAGTGTTATGAAAGAATTTAAGGTTCTTGCTTATTTTGTTAATGGTACAGCACAAGATGTAA
TTTTGTGTGACAAGTCCCCCAAGGGTTTGCTAGCTTGTCAATATAACACTGGCAATTTTTCAGATGGCTTTTAT
CCTTTTACTAATACTACTTTAGTTAGGGAAAAGTTCATCGTATATCGTGAAAGTAGTGTTAATACTACTCTGGC
GTTAACTAATTTCACTTTTACTAATGTAAGTAATGCACAGCCTAATAGTGGTGGTGTTAATACTTTTCATTTATAT
CAAACACAAACAGCTCAGAGTGGTTATTATAATTTTAATTTCTCATTTCTGAGTCAGTTTGTGTATAAGGCAAG
TGATTTTATGTATGGGTCTTATCATCCTAGGTGTTCTTTTAGACCAGAAACCATTAATAATGATTTGTGGTTTAA
TTCCTTGTCAGTTTCTCTTACTTATGGACCCTTACAGGGAGGGTGTAAGCAATCTGTTTTTAGTAGTAAGGCA
ACGTGTTGTTATGCCTACTCTTATAATGGCCCTAGAGTATGTAAAGGTGTTTATTCAGGTGAATTAAGCAAGAC
TTTTGAATGTGGATTGCTGGTTTATGTTACTAAGAGTGATGGCTCTCGTATACAGACTAGAACAGAGCCCTTA
GTATTAACGCAACACAATTATAATAATATTACTTTAGATAAGTGTGTTAACTATAATATATATGGCAGAGTGGGC
CAAGGTTTTATTACTAATGTGACTGATTCTGCTGCTAATTTTAGTTATTTAGCAGATGGTGGGTTAGCTATTTTA
GACACTTCGGGTGCCATAGATGTTTTTGTTGCACAGGGCAGCTATGGTCCTAATTATTACAAGGTTAATCCTT
GTGAAGATGTTAACCAACAGTTTGTAGTGTCTGGTGGCAATATAGTTGGCATTCTTACTTCTAGAAATGAAAC
AGGTTCTGAACAGGTTGAGAACCAGTTTTATGTTAAGTTAACCAATAGCTCACATCGTCGTAGGCGC
>CK_CH_GD_QY_YF-YD-11-007-5_20221115.seq
ATGTTGGGGAAGTCACTGTTTTTAGTGACCATTTTGTGTGCACTATGTAGTGCAAATTTGTTTGATTTTGCCA
ATAATTATGTGTACTACTACCAAAGTTCCTTTAGGCCCCCAGATGGATGGCATTTGCAAGGGGGTGCTTATGC
AGTAGTGAATTCTACTAATTATACTAGTAATGCCGGTTCTGCAAGTGGGTGCACTGTTGGTATTATTAAGGAC
GTCTATAATCAAAGTGCGGCTTCCATAGCTATGACAGCACCCTCTCAGGGTATGGCTTGGTCTAAGTCACAAT
TTTGTAGTGCACACTGTAACTTTTCTGAAATTACAGTTTTCGTCACACATTGTTATAGTAGTGGTACAGGGTCT
TGCCCTATAACAGGCATGATTGCACGTGATCATATTCGTATTTCTGCAATGAAAAATGGTTCTTTATTTTATAAC
TTAACAGTTAGCGTATCTAAATACTCTAGGTTTAAGTCTTTTCAATGTGTTAACAACTTCACATCTGTCTATCTA
AATGGTGATCTTGTTTTTACTTCCAACAAAACTACTGATGTTACGTCAGCAGGTGTGTATTTTAAAGCAGGTG
GACCTGTAAATTATAGTGTTATGAAAGAATTTAAGGTTCTTGCTTATTTTGTTAATGGTACAGCACAAGATGTA
ATTTTGTGTGACAAATCCCCTAAGGGTTTGCTAGCTTGTCAATATAACACTGGCAATTTTTCGGATGGCTTTTA
TCCTTTTACTAATACTACTTTAGTTAGGGAAAAGTTCATCGTATATCGTGAAAGTAGTGTTAATACTACTCTGGC
GTTAACTAATTTCACTTTTACTAATGTAAGTAATGCACAGCCTAATAGTGGTGGTGTTAATACTTTTCATTTATAT
CAAACACAAACAGCTCAGAGTGGTTATTATAATTTTAATTTCTCATTTCTGAGTCAGTTTGTGTATAAGGCAAG
TGATTTTATGTATGGGTCTTATCATCCTAGGTGTTCTTTTAGACCAGAAACCATTAATAATGATTTGTGGTTTAA
TTCCTTGTCAGTTTCTCTTACTTATGGACCCCTACAGGGAGGGTGTAAGCAATCTGTTTTTAGTGGTAAGGCA
ACGTGTTGTTATGCCTACTCTTATAATGGCCCTAGAGTATGTAAAGGTGTTTATTCAGGTGAATTAAGCAAGAC
TTTTGAATGTGGATTGCTGGTTTATGTTACTAAGAGTGATGGCTCTCGTATACAGACTAGAACAGAGCCCTTA
GTATTAACGCAACACAATTATAATAATATTACTTTAGATAAGTGTGTTAACTATAATATATATGGCAGAGTGGGC
CAAGGTTTTATTATTAATGTGACTGATTCTGCTGCTAATTTTAGTTATTTAGCAGATGGTGGGTTAGCTATTTTA
GACACTTCGGGTGCCATAGATGTTTTTGTTGCACAGGGCAGCTATGGTCCTAATTATTACAAGGTCAATCCTT
GTGAAGATGTTAACCAACAGTTTGTAGTGTCTGGTGGCAATATAGTTGGCATTCTTACTTCTAGAAATGAAAC

AGGTTCTGAACAGGTTGAGAACCAGTTTTATGTTAAGTTAACCAATAGCTCACATCGTCGTAGGCGC
>CK_CH_GD_HY_LH-LCZ_20220216.seq
ATGTTGGGGAAGTCACTGTTTTTAGTGACCATTTTGTGTGCACTATGTAGTGCAAATTTATTCGATTTAGCCAA
TAATTATGTGTACTATTACCAAAGTGCCTTTAGGCCTTCAAATGGATGGCACTTGCATGGGGGTGCTTATGCA
GTAGTGAATTCCACTATTAAATATAACAATGCAGGCTCCGCTAGTGCGTGTTCTGTAGGTGTTCTCTTTAATTAT
TCTAACGGAAATGATGTTGGTTATAATAATAGTGCTTCTTCTGTAGCCATGACAGCACCATTGTCTGGTATGTC
TTGGTCTAAAGAAGAATTTTGTACTGCCCACTGTAACTTTTCGGATATTACAGTGTTTGTTACACATTGTTATG
CACAATCTTGTCCTTTAACGGGTCAGTTAGGTAAGGGCCATATTCGTATTTCTGCTATGAGAAATGGTTCTCTA
TTTTATAATTTAACAGTTGGTGTATCCCAATACCCTAAATTTAAATCGCTTCAATGTGTTAACAACCTCACTTCT
GTTTATTTAAATGGTGATCTTGTTTTTACTTCTAATGCAAGCATTGATGTTATAGGTGCTGGTGTGTACTTTAAA
GCAGGTGGGCCTATAACCTACAAAATTATGAGAGAATTTAAGGTTTTGGCTTATTTTTTAAATGGCACTGCGC
AAGATGTAATTTTGTGTGATGACACACCGAGAGGCTTGCTTGCATGTCAATATAATACTGGTAATTTTTCAGAT
GGGTTTTATCCTTTTACTAATTCTAGTTTAGTTAAGGAAAAGTTTATTGTTTATCGTCAGAATAGTGTCAATACT
ACTCTTACCTTAACTAACTATACTTTTTCTAATGAGACTAATGCCCCTCCTAATTCAGGTGATGTTCATTCTATTC
CAACTTATCAAACACAAACAGCTCAGAGTGGTTATTATAATTTTAATTTATCATTTCTGAGTAGTTTTGTGTATA
AAGAGTCTAATTACATGTACGGGTCTTATCACCGTGCATGCAATTTTAGATTAGAAAATATTAATAATGGCTTG
TGGTTTAATTCACTGTCAGTTACGCTAGCTTATGGACCACTTCAAGGTGGGTGTAAGCAGTCAGTCTTTAGTG
GTAGAGCCACTTGTTGTTATGCTTATTCATATAATGGTCCTCACCTCTGTAAGGGTGTTTACAGCGGCGAGTTA
AAAAAAAGTTTTGAATGTGGATTGTTGGTTTATGTTACTAAGCGTGATGGCTCTCGTATACAAACAGCCACCG
TTCCACCAGCTATAACTCAACACAATTATAATAATATTACTTTAAATAAGTGTGTTGACTATAATATATATGGCAG
AGTAGGCCAAGGTTTTATTACTAATGTGACTGATTCTGCTGCTAATTTTAGTTATTTAGCAGATGGTGGGTTAG
CTATTTTAGATACTTCGGGTGCCATAGATGTTTTTGTTGTACAGGGCAGCTATGGTCTTAATTATTACAAGGTT
AATCCTTGTGAAGATGTTAACCAACAGTTTGTAGTGTCTGGTGGCAACATAGTTGGCATGCTTACTTCTAGAA
ACGAAACAGGTTCTGAACAGGTTGAGAACCAGTTTTATGTTAAGTTAACCAATAGCTCACATCGTCGCAAGC
GT
>CK_CH_GD_ZQ_SHHF-5_20221229.seq
ATGTTGGAGAAGTTACTGTTTTTAGTGACCATTTTGTGTGCACTATGTAGTGCAAATTTGTTTGATGCTGATAA
TAGTTATGTGTACTACTACCAGAGTGGATTTAGACCCCCTACAGGTTGGCACCTTTATGGTGGTGCGTATGCA
GTAGAACGGTTTTTTAATGAAACCAGCAATGCAGGCTCTCACGAATGTACTGCTGGAGCTATTGTACATAGTT
TCAATGTTAGTGCAAGTGCAGTTGCGATTACTACACCTGTTAATGGCATGCATTGGTCATCTAGTAATGGAGT
GTGTTCAATACATTGCAATTTTAGTACAATTGTTGTTTTTGTTACACATTGTTTTAAAAATGGACAAGGAATAT
GTCCCTTGACAGGTAGATTAAGGGAGGGTGATATTCGTATTGGTGTTCTAGATAGTAGTGGTAATTCTATTTTT
AATAAAACAGTTACCACTTCTAGTTATAGTAAATTTAAATCATTACATTGCGTTAACAATTTCACTTCTGTATATT
TAAATGGTGATCTTGTTTACACGTCTAATGAAACTTCAGATATTACTGGTTTTGGTGTACATTTTAAGACAGGA
GGACCTGTTACTTATAAAATTATGAAAGAACATAAGGTTCTAGCATATTTTGAAAATGGTACTGCACACGACA
TTATTTTATGTGATGACAGTCCCCGTGGTAGGTTAGCTTGTCAGTATAATACAGGCAATTTTTCTGACGGTTTG
TACCCTTTTAGCGTAAGCAGTGAAGTTAATGAAACTTTTATAGTTTTTGAAAAGAATACAGAAACTACTATGC
TTACATTAAATAATTTCACTTTTTTTAATCAGAGTGGGGCTCAACCTAATCAAAAGGAACCTTCACCTGGTGTT
TCAAATTTTGTGTATTATCAACAGATTAGTGCTGTTCCTGGTTATAATAATTTTAATTTTTCCTTTTTGCGTTCTT
TTACTTATTTAAGTAGTGATTATATGAGGGGTTCTTTTCACCCAAGTTGTACTTTTAGGCCTGAAGATATTAATA
AAAATCGCAGGTTTAATCATTTGTCTATATCCTTATCTTACGGTCCTCGTAATGGAGGCTGTAAGCAAGCATGC
TTTAATACTAGGAGTTCATGTTGTTGTTTCATGTACTCTTATAATGGTCAACCTCTTTGTAAAGGTGTGTATAGT
GGTGATTTAAATCAAGATTTTGAGTGCGTATTGCTTGTGTTTATTAAACATAGCCCAGGCAGCCGTATATTTAC
TTCTGAAACAGTACCTACTTTCACTGCTAATTTTGTAAATAATGTGGTTTTAGATAGGTGTGTTGATTATAATAT

CTATGTTAGTTATGGCCGGTTTTGA
>CK_CH_LN_DL_DC-WB_20221225(1).
seqATGTTGGAGAAGTTACTGTTTTTAGTGACCATTTTGTGTGCACTATGTAGTGCGAATTTGTTTGATGCTGA
TAATAGTTATGTGTACTACTACCAGAGTGGATTTAGACCTCCGTCAGGTTGGCACCTTTATGGTGGTGCGTAT
GCAGTAGAACGGTTTTTTAATGAAACCAGCAATGCAGGCTCTCATGAATGTACTGCTGGAGCTATTGTACATA
GTTTGAATGTTAGTGCAAGTGCAGTTGCGATTACTACACCTGTTAATGGCATGCGTTGGTCATCTAGTAATGG
AGTGTGTTCAATACATTGCAATTTTAGTACAATTGTTGTTTTTGTTACACATTGTTTTAAAAATGGACAAGGAA
TATGTCCCTTGACAGGTAAATTAAGGGAGGGTGATATTCGTATTGGTGTTCTAGATAGTAGTGGTAATTCTATT
TTTAATAAAACAGTTACCACTTCTAGTTATAGTAAATTTAAATCATTACATTGTGTTAACAATTTCACTTCTGTGT
ATTTAAACGGTGATCTTGTTTACACGTCTAATGAAACTTCTGATATTATTGGTTTTGGTGTACATTTTAAGACA
GGAGGTCCTGTTACTTATAAAATTATGAAAGAACATAAGGTTCTAGCATATTTTGAAAATGGTACTGCACATG
ACATTATTTTATGTGATGACAGTCCCCGTGGTAGGTTAGCTTGTCAGTATAATACAGGCAATTTTTCTGACGGT
TTGTACCCTTTTAGCGTAAGCAGTGAAGTTAATGAAACTTTTATAGTTTTTGAAAAGAATACAGAAACTACTA
TGCTTACATTAAATAATTTCACTTTTTTTAATCAGAGTGGGGCTCAACCTAATCAAAGGGAACCTTCACCTGG
TGTTTCAAATTTTGTATATTATCAACAGATTAGTGCCGTTCCTGGTTATAATAATTTTAATTTTTCCTTTTTGCGT
TCTTTTACTTATTTAAGTAGTGATTATATGAGGGGTTCTTTTCACCCAAGTTGTACTTTTAGGCCTGAAGATATT
AATAAAAATCGCAGGTTTAATCATTTGTCTATATCCTTATCTTACGGTCCTCGTAATGGAGGCTGTAAGCAAGC
ATGCTTTAATACTAGGAGTTCATGTTGTTGTTTCATGTACTCTTATAATGGTCAACCTCTTTGTAAAGGTGTGTA
TAGTGGTGATTTAAATCAAGATTTTGAGTGCGTATTGCTTGTGTTTATTAAACATAGCCCAGGCAGTCGTATAT
TTACTTCTGAAACAGTACCTACTTTCACTGCTAATTTTGTAAATAATGTGGTTTTAGATAGGTGTGTTGATTATA
ATATCTATGTTAGATAAGGCCAGGGTTTAA
>CK_CH_GD_QY_YF_20220110.seq
ATGTTGGTAACACCTCTTTTACTAGTGACTCTTTTGTGTGCACTATGTAGTGCTGCTTTGTATGACAGTAGTTC
TTACGTGTACTACTACCAAAGTGCCTTCAGACCACCTGATGGTTGGCATTTACATGGGGGTGCGTATGCGGTT
GTTAATATTTCTAGTGAATCTAATAATGCAGGCTCTTCATCTGAGTGTACTGTTGGTATTATTCATGGTGGTCGT
GTTGTTAATGCTTCTTCTATAGCTATGACGGCACCGTCATCAGGTATGGCTTGGTCTAGCAGTCAGTTTTGTAC
TGCATACTGTAACTTTTCAGATACTACAGTGTTTGTTACACATTGTTACAAACATGTTGGGTGTCCTATAACTG
GCATGCTTCAACAGCATTCTATACGTGTTTCTGCTATGAAAAATGGCCAGCTTTTTTATAATTTAACAGTTAGT
GTAGCTAAGTACCCTACTTTTAAATCATTTCAGTGTGTTAATAATTTAACATCCGTATATTTAAATGGTGATCTTG
TTTACACCTCTAATGAGACCACAGATGTTACATCTGCAGGTGTTTATTTTAAAGCTGGTGGACCTATAACTTAT
AAAGTTATGAGACAAGTTAGAGCCCTGGCTTATTTTGTTAATGGTACTGCACAAGATGTTATTTTGTGTGATG
GGTCACCTAGAGGCTTGTTAGCATGCCAGTATAATACTGGCAATTTTTCAGATGGCTTTTATCCTTTTACTAAT
AGTAGTTTAGTTAAGCAGAAGTTTATTGTCTATCGTGAAAATAGTGTTAATACTACTCTTACGTTACACAATTTC
ACTTTTCATAATGAGACTGGCGCCAACCCAAATCCTAGTGGTGTCCAGAATATTCAAACTTACCAAACACAAA
CAGCTCAGAGTGGTTATTATAATTTTAATTTTTCCTTTCTGAGTAGTTTTGTTTATAAGGAGTCTAATTTTATGTA
TGGATCTTATCACCCAAGTTGTAATTTTAGACTAGAAACTATTAATAATGGTTTGTGGTTTAATTCACTTTCAGT
TTCAATTGCTTACGGTCCTCTTCAAGGTGGTTGCAAGCAATCTGTCTTTAGTGGTAGAGCAACCTGTTGTTAT
GCTTACTCATATGGAGGTCCTTTGTTGTGTAAAGGTGTTTATTCAGGTGAGTTAGATCATAATTTTGAATGTGG
ACTGTTAGTTTATGTTACTAAGAGCGGTGGCTCTCGTATACAAACAGCCACTGAACCGCCAGTTATAACTCAA
CACAATTATAATAATATTACTTTAAATACTTGTGTTGATTATAATATATATGGCAGAACTGGCCAAGGTTTTATTA
CTAATGTAACCGACTCAGCTGTTAGTTATAATTATCTAGCAGACGCAGGTTTGGCTATTTTAGATACATCTGGT
TCCATAGACATCTTTGTCGTACAAAGTGAATATGGTCTTAATTATTATAAGGTTAACCCTTGCGAAGATGTCAA
CCAGCAGTTTGTAGTTTCTGGTGGTAAATTAGTAGGTATTCTTACTTCACGTAATGAGACTGGTTCCCAGCTT
CTTGAGAATCAGTTTTACATCAAAATCACTAATGGAACACGTCGTTTTAGACGT

>CK_CH_GZ_GY_XF-DK_20220222.seq
ATGTTGGTAACACCTCTTTTACTAGTGACTCTTTTGTGTGCACTATGTAGTGCTGCTTTGTATGACAGTAGTTC
TTACGTGTACTACTACCAAAGTGCCTTCAGACCACCTGATGGTTGGCATTTACATGGGGGTGCGTATGCGGTT
GTTAATATTTCTAGTGAATCTAATAATGCAGGCTCTTCATCTGGGTGTACTGTTGGTATTATTCATGGTGGTCGT
GTTGTTAATGCTTCTTCTATAGCTATGACGGCACCGTCATCAGGTATGGCTTGGTCTAGCAGTCAGTTTTGTAC
TGCATACTGTAACTTTTCAGATACTACAGTGTTTGTTACACATTGTTATAAACATGTTGGGTGTCCTATAACTGG
CATGCTTCAACAGCATTCTATACGTGTTTCTGCTATGAAAAATGGCCAGCTTTTTTATAATTTAACAGTTAGTGT
AGCTAAGTACCCTACTTTTAAATCATTTCAGTGTGTTAATAATTTAACATCCGTATATTTAAATGGTGATCTTGTT
TACACCTCTAATGAGACCACAGATGTTACATCTGCAGGTGTTTATTTTAAAGCTGGTGGACCTATAACTTATAA
AGTTATGAGAGAAGTTAGAGCCCTGGCTTATTTTGTTAATGGTACTGCACAAGATGTTATTTTGTGTGATGGG
TCACCTAGAGGCTTGTTAGCATGCCAGTATAATACTGGCAATTTTTCAGATGGCTTTTATCCTTTTACTAATAGT
AGTTTAGTTAAGCAGAAGTTTATTGTCTATCGTGAAAATAGTGTTAATACTACTTTTACGTTACACAATTTCACT
TTTCATAATGAGACTGGCGCCAACCCAAATCCTAGTGGTGTCCAGAATATTCAAACTTACCAAACACAAACA
GCTCAGAGTGGTTATTATAATTTTAATTTTTCCTTTCTGAGTAGTTTTGTTTATAAGGAGTCTAATTTTATGTATG
GATCTTATCACCCAAGTTGTAATTTTAGACTAGAAACTATTAATAATGGTTTGTGGTTTAATTCACTTTCAGTTT
CAATTGCTTACGGTCCTCTTCAAGGTGGTTGCAAGCAATCTGTCTTTAGTGGTAGAGCAACCTGTTGTTATGC
TTACTCATATGGAGGTCCTTTGCTGTGTAAAGGTGTTTATTCAGGTGAGTTAGATCATAATTTTGAATGTGGAC
TGTTAGTTTATGTTACTAAGAGCGGTGGCTCTCGTATACAAACAGCCACTGAACCGCCAGTTATAACTCAACA
CAATTATAATAATATTACTTTAAATACTTGTGTTGATTATAATATATATGGCAGAACTGGCCAAGGTTTTATTACT
AATGTAACCGACTCAGCTGTTAGTTATAATTATCTAGCAGACGCAGGTTTGGCTATTTTAGATACATCTGGTTC
CATAGACATCTTTGTCGTACAAAGTGAATATGGTCTTAATTATTATAAGGTTAACCCTTGCGAAGATGTCAACC
AGCAGTTTGTAGTTTCTGGTGGTAAATTAGTAGGTATTCTTACTTCACGTAATGAGACTGGTTCCCAGCTTCTT
GAGAATCAGTTTTACATCAAAATCACTAATGGAACACGTCGTTTTAGACGT
>CK_CH_YN_KM_DK-SWH_20221117.seq
ATGTTGGTGAAGTCACTGTTTTTAGTGACTCTTTTGTTTGCACTATCTAGTGCTACTTTGTATGATAATGATACG
TACGTTTACTACTACCAGAGCGCCTTCAGACCGTCTAGTGGTTGGCATTTACATGGTGGCGCTTATGCAGTAG
TAAATGTTTCTTCACAAACTAACAATGCAGGTGCAGCTTCAGAATGCACTGTTGGTATTATTAGTGGTGATAC
AGTTGTTAATGCCTCTTCTATAGCTATGACAGCACCTGTAGGTCAAGGTATGCAGTGGTCTAAGTTACAATTTT
GTACTGCACACTGCAATTTTTCTGATTTTACAGTGTTTGTTACACATTGCTATGCCTCGGGCAGTGGTAAATGT
CCTTTAACGGGCCTTATCCCACAAGGTCATATTCGTATTTCTGCTATGCGGAATCATACTTTATTTTATAATTTAA
CAGTTAGTGTATCTAATTACCCTACTTTTAAATCTTTGCAATGCGTTGATAATTTCACGTCTGTTTACTTAAATG
GTGATCTTGTCTTCACTTCTAATCAGACTACAAACGTTATAAGTGCAGGTGTGTACTTTAAAGCAGGTGGGCC
TATAACCTATAAAGTTATGAAGGAATTTAAGGTTTTGGCTTATTTTGTTAATGGTACTGCACAAGATGTTATTTT
GTGTGATGACACACCTAGAGGTTTGCTAGCATGTCAATATAATACTGGCAATTTCTCAGATGGTTTCTATCCTT
TTACTAATAGTAGCTTAGTTAGGCAAAGGTTTGTTGTTTATCGTGAGAATAGTGTTAATACTACTCTTACTTTAA
CCAATTACACCTTTCATAATGAGACTACTGCTCAGCCTAATTCAGGTGATGTTCATACTATCTTAACTTATCAAA
CACAAACTGCCCAGAGTGGTTATTATAATTTTAATTTATCATTTCTGAGTAGTTTTGAGTATAAAGCTTCTGATT
ATATGTATGGGTCTTACCACCCACGATGTAGTTTTAGACCAGAAACTATTAATAATGGCTTGTGGTTTAATTCA
CTCTCAGTCTCGTTAGCCTATGGCCCCCTTCAAGGTGGGTGTAAGCAATCAGTCTTTCAAGGCAGAGCTACT
TGTTGTTATGCGTATTCATATAACGGACCACGTATGTGTAAAGGTGTTTATAGTGGTCAGTTATTACAAGATTTT
GAATGTGGACTGTTGGTTTATGTTACTAAGAGTGATGGCTCTCGTATACAAACAACCACAAAACCACCGGTTA
TAACTCAACACAATTATAATAATATTACTTTAAATACTTGTGTTGAGTACAATATATATGGCAGAGTAGGCCAAG
GCTTTATTACTAATGTAACTGACTCCGCAGCTAGCTATAATTACTTAGCAGATGCTGGATTGGCAATTTTAGAT
ACTTCAGGTGCCATAGACACTTTCGTTGTACAAGGTGAATATGGTCCCAATTATTATAAGGTTAACCCTTGTGA

AGATGTTAACCAGCAGTTTGTAGTGTCAGGCGGTAAGTTAGTAGGCATCCTGACTTCTCGTAATGAAACTGG
CTCTCAGCCTCTTGAAAATCAGTTTTATATTAAGTTAACTAATGGAAGCCGTCGTTCTAGACGT
>CK_CH_GZ_NY_QHNM_20220421.seq
ATGTTGGGGAAGTCACTGTTTCTAGTGACCATTTTGTGTGCACTATGTAGTGCAAATTTGTTTGATTTAGCCA
ATAATTATGTGTACTACTACCAAAGTGCCTTTAGGCCTCCAAATGGATGGCACTTGCATGGGGGTGCTTATGC
AGTAGTGAATTCTACTATTAAATATAACAATGCAGGCTCTGCTAGTGCGTGTTCTGTAGGTGTTCTCTTTAATTA
TTCTAACGGAAATGATGTTGGTTATAATAATAGTGCTTCTTCTGTAGCCATGACAGCACCGTTGTCTGGTATGT
CTTGGTCTAAAGAAGAATTTTGTACTGCCCACTGTAACTTTTCGGATATTACAGTGTTTGTTACACATTGTTAT
GCACAATCTTGTCCTTTAACGGGTCAGTTAGGTAAGGGCCATATTCGTATTTCTGCTATGAGAAATGGTTCTCT
ATTTTATAATTTAACAGTTGGTGTATCTCAATACCCTAAATTTAAATCGCTTCAATGTGTTAACAACTTCACTTCT
GTTTATTTAAATGGTGACCTTGTTTTTACTTCTAATGAAAGCAGTGATGTTATAGGTGCTGGTGTGTACTTTAA
AGCAGGTGGGCCTATAACCTACAAAATTATGAGAGAATTTAAGGTTTTGGCTTATTTCTTAAATGGCACTGCG
CAAGATGTAATTTTGTGTGATGACACACCGAGAGGCTTGCTTGCATGTCAATATAATACTGGTAATTTTTCAGA
TGGGTTTTACCCTTTTACTAATTCTAGTTTAGTTAAGGAAAAGTTTATTGTTTATCGTCAGAATAGTGTCAATAC
TACTCTTACCTTAACTAATTATACTTTTTCTAATGAGACTAATGCCCCTCCTAATTCAGGTGATGTTCATTCTATT
CCAACTTATCAAACACAAACAGCTCAGAGTGGTTATTATAATTTTAATTTATCATTTCTGAGTAGTTTTGTGTAT
GAAGAGTCTAATTACATGTACGGGTCTTATCACCATGCATGCAATTTTAGATTAGAAAATATTAACAATGGCTT
GTGGTTTAATTCACTGTCAGTTACGCTAGCTTATGGACCACTTCAAGGTGGGTGTAAGCAGTCAGTCTTTAGT
GGTAGAGCCACTTGTTGTTATGCTTATTCATATAATGGTCCTCACCTCTGTAAGGGTGTTTACAGCGGCGAGTT
AAAAAAAAGTTTTGAATGTGGATTGTTGGTTTATGTTACTAAGCGCGATGGCTCTCGTATACAAACAGCCACC
GTTCCACCAGTTATAACTCAACACAATTATAATAATATTACTTTAAATAAGTGTGTTGACTATAATATATATGGCA
GAGTAGGCCAAGGTTTTATTACTAATGTGACTGATTCTGCTGCTAATTTTAGTTATTTAGCAGATGGTGGGTTA
GCTATTTTAGATACGTCGGGTGCCATAGATGTTTTTGTTGTACAGGGCAGCTATGGTCTTAATTATTACAAGGT
TAATCCCTGTGAAGATGTTAACCAACAGTTTGTAGTGTCTGGTGGCAATATAGTTGGCATTCTTACTTCTAGAA
ATGAAACAGGTTCTGAACAGGTTGAGAACCAGTTTTATGTTAAGTTAACCAATAGCTCACATCGTCGCAGGC
GT
>CK_CH_YN_KM_LZY-FMQ_20220413.seq
ATGTTGGGCAAACCGCTTTTACTAGTGACTCTTTGGTATGCACTATGTAGTGCTTTGCTTTATGATAAAAATAC
TTACGTTTACTACTACCAAAGTGCCTTTAGGCCTGGTCAAGGTTGGCATCTACATGGGGGTGCTTATGCAGTA
GATAAGGTTTTTAATGAAACCAACAATGCAGTCAGTGTATCTGATTGCACTGCTGGTACTTTTTATGAAAGCT
ATAATATTTCTGCTGCTTCTGTAGCCATGACAGTACCACCTGCTGGTATGTCTTGGTCAGTTTCACAGTTTTGT
ACAGCTCATTGTAACTTCTCAGACTTTACAGTGTTTGTTACGCATTGTTTTAAAAGTCAACAAGGTAGTTGTC
CATTGACAGGTATGATTCCTCAGAATCATATTCGTATTTCTGCTATGAGATCTGGATTTTTGTTTTATAATTTAAC
AGTTAGCGTATCTAAATACCCTAAATTTAAATCGCTTCAATGTGTTGGCAATTCTACATCTGTCTATTTAAATGG
TGATCTTGTTTTCACTTCTAATGAAACAACTCACGTTACGGGTGCAGGCGTTTATTTTAAAAGTGGTGGGCCT
GTAACTTATAAAGTTATGAAAGAAGTTAAAGCCCTAGCCTACTTTATTAATGGTACCGCACAAGAGGTTATTTT
ATGTGATAACTCACCTAGAGGTTTGCTTGCATGTCAGTATAACACTGGTAATTTTTCAGATGGATTCTACCCTT
TTACTAATTCTTCTTTAGTTAAGGATAGGTTTATTGTATATCGAGAAAGTAGCACTAACACTACTTTAGAGTTAA
CTAATTTCACTTTTACTAATGTAAGTAATGCTTCTCCTAATTCAGGTGGCGTTGATACTTTCCAATTATATCAAA
CACATACTGCTCAGGATGGTTATTATAATTTTAATTTATCATTTCTGAGTAGTTTTGTGTATAAACCATCTGATTT
TATGTATGGGTCATACCACCCAAATTGTAATTTTAGACCAGAGAATATTAATAATGGCTTATGGTTTAATTCATT
ATCTGTGTCACTTACTTACGGACCCATTCAAGGTGGTTGTAAGCAATCTGTTTTTAGTAATAAAGCAACTTGTT
GCTATGCTTATTCTTACCGAGGTCCTACTAGATGTAAGGGTGTTTATAGAGGGGAGCTAACGCAATACTTTGA
ATGTGGACTTCTAGTTTATGTAACTAAGAGTGATGGCTCTCGTATACAAACTAGAAGTGAACCACTGGTGTTA

ACTCAATATAATTATAACAACATTACTTTAAATAAGTGTGTTGAGTATAATATATATGGTAGAGTTGGTCAAGGT
TTTATTACTAATGTAACTGAAGCAACTGCTAATTATAGTTATCTAGCAGATGGTGGTTTAGCTATTTTAGATACT
TCAGGAGCCATAGACATATTTGTTGTTCGAGGTGCATATGGTCTTAATTATTATAAGGTTAATCCCTGTGAAGA
TGTTAACCAACAGTTTGTAGTGTCTGGTGGCAATCTAGTTGGCATTCTTACATCTCATAATGAAACAGATTCTG
AATTTATTGAGAACCGGTTTTACATCAAACTCACTAACGGAACACGTCGCTCTAGACGT
>CK_CH_GD_HZ_LH-BXH_20221227.seq
ATGTTGGGGAAGTCACTGTTCTTAATGATCATTTTGTGTGCACTATGTAGTGCAAATTTGTTTGATTTAGCCAA
TAATTATGTGTACTACTACCAAAGTGCCTTTAGGCCTCCAAATGGATGGCACTTGCATGGGGGTGCTTATGCA
GTAGTGAATTCTACTATTAAATATAACAATGCAGGCTCCGCTAGTGCGTGTTCTGTAGGTGTTCTCTTTAATTAT
TCTAACGGAAATGATGTTGGTTATAATAATAGTGCTTCTTCTGTAGCCATGACAGCACCATTGTCTGGTATGTC
TTGGTCTAAAGAAGAATTTTGTACTGCCCACTGTAACTTTTCGGATATTACAGTGTTTGTTACACATTGTTATG
CACAATCTTGTCCTTTAACGGGTCAGTTAGGTAAGGGCCATATTCGTATTTCTGCTATGAGAAATGGTTCTCTA
TTTTATAATTTAACAGTTGGTGTATCCCAATACCCTAAATTTAAATCGCTTCAATGTGTTAACAACCTCACTTCT
GTTTATCTAAATGGTGACCTTGTTTTTACTTCTAATGAAAGCACTAATGTTATAGGTGCTGGTGTGTACTTTAA
AGCAGGTGGGCCTATAACCTACAAAATTATGAGAGAATTTAAGGTTTTGGCTTATTTTTTAAATGGCACTGCG
CAAGATGTAATTTTGTGTGATGACACACCGAGAGGCTTGCTTGCATGTCAATATAATACTGGTAATCTTTCAGA
TGGGTTTTATCCTTTTACTAATTCTAGTTTAGTTAAGGAAAAGTTTATTGTTTATCGTCAGAATAGTGTCAATAC
CACTCTTACCTTAACTAACTATACTTTTTCTAATGAGACTAATGCCCCTCCTAATTCAGGTGATGTTCATTCTATT
CCAACTTATCAAACACAAACAGCTCAGAGTGGTTATTACAATTTTAATTTATCATTTCTGAGTAGTTTTGTGTAT
AAAGAGTCTAATTACATGTACGGGTCTTATCACCGTGCATGCAATTTTAGATTAGAAAATATTAATAATGGCTT
GTGGTTTAATTCACTGTCAGTTACGCTAGCTTATGGACCACTTCAAGGTGGGTGTAAGCAGTCAGTCTTTAGT
GGTAGAGCCACTTGTTGTTATGCTTATTCATATAATGGTCCTCACCTCTGTAAGGGTGTTTACAGCGGCGAGTT
ACAAAAAAGTTTTGAATGTGGATTGTTGGTTTATGTTACTAAGCGTGATGGCTCTCGTATACAAACAGCCACC
GTTCCACCAGTTATAACTCAACACAATTATAATAATATTACTTTAAATAAGTGTGTTGACTATAATATATATGGCA
GAGTAGGCCAAGGTTTTATTACTAATGTGACTGATTCTGCTGCTAATTTTAGTTATTTAGCAGATGGTGGGTTA
GCTATTTTAGATACTTCGGGTGCCATAGATGTTTTTGTTGTACAGGGCAGCTATGGTCTTAATTATTACAAGGT
TAATCCTTGTGAAGATGTTAACCAACAGTTTGTAGTGTCTGGTGGCAACATAGTTGGCATGCTTACTTCTAGA
AATGAAACAGGTTCTGAACAGGTTGAGAACCAGTTTTATGTTAAGTTAACCAATAGCTCACATCGTCGCAAG
CGT
>CK_CH_GD_HZ_LH-YHL_20221227.seq
ATGTTGGGGAAGTCACTGTTCTTAATGATCATTTTGTGTGCACTATGTAGTGCAAATTTGTTTGATTTAGCCAA
TAATTATGTGTACTACTACCAAAGTGCCTTTAGGCCTCCAAATGGATGGCACTTGCATGGGGGTGCTTATGCA
GTAGTGAATTCTACTATTAAATATAACAATGCAGGCTCCGCTAGTGCGTGTTCTGTAGGTGTTCTCTTTAATTAT
TCTAACGGAAATGATGTTGGTTATAATAATAGTGCTTCTTCTGTAGCCATGACAGCACCATTGTCTGGTATGTC
TTGGTCTAAAGAAGAATTTTGTACTGCCCACTGTAACTTTTCGGATATTACAGTGTTTGTTACACATTGTTATG
CACAATCTTGTCCTTTAACGGGTCAGTTAGGTAAGGGCCATATTCGTATTTCTGCTATGAGAAATGGTTCTCTA
TTTTATAATTTAACAGTTGGTGTATCCCAATACCCTAAATTTAAATCGCTTCAATGTGTTAACAACCTCACTTCT
GTTTATCTAAATGGTGACCTTGTTTTTACTTCTAATGAAAGCACTAATGTTATAGGTGCTGGTGTGTACTTTAA
AGCAGGTGGGCCTATAACCTACAAAATTATGAGAGAATTTAAGGTTTTGGCTTATTTTTTAAATGGCACTGCG
CAAGATGTAATTTTGTGTGATGACACACCGAGAGGCTTGCTTGCATGTCAATATAATACTGGTAATCTTTCAGA
TGGGTTTTATCCTTTTACTAATTCTAGTTTAGTTAAGGAAAAGTTTATTGTTTATCGTCAGAATAGTGTCAATAC
CACTCTTACCTTAACTAACTATACTTTTTCTAATGAGACTAATGCCCCTCCTAATTCAGGTGATGTTCATTCTATT
CCAACTTATCAAACACAAACAGCTCAGAGTGGTTATTACAATTTTAATTTATCATTTCTGAGTAGTTTTGTGTAT
AAAGAGTCTAATTACATGTACGGGTCTTATCACCGTGCATGCAATTTTAGATTAGAAAATATTAATAATGGCTT

GTGGTTTAATTCACTGTCAGTTACGCTAGCTTATGGACCACTTCAAGGTGGGTGTAAGCAGTCAGTCTTTAGT
GGTAGAGCCACTTGTTGTTATGCTTATTCATATAATGGTCCTCACCTCTGTAAGGGTGTTTACAGCGGCGAGTT
ACAAAAAAGTTTTGAATGTGGATTGTTGGTTTATGTTACTAAGCGTGATGGCTCTCGTATACAAACAGCCACC
GTTCCACCAGTTATAACTCAACACAATTATAATAATATTACTTTAAATAAGTGTGTTGACTATAATATATATGGCA
GAGTAGGCCAAGGTTTTATTACTAATGTGACTGATTCTGCTGCTAATTTTAGTTATTTAGCAGATGGTGGGTTA
GCTATTTTAGATACTTCGGGTGCCATAGATGTTTTTGTTGTACAGGGCAGCTATGGTCTTAATTATTACAAGGT
TAATCCTTGTGAAGATGTTAACCAACAGTTTGTAGTGTCTGGTGGCAACATAGTTGGCATGCTTACTTCTAGA
AATGAAACAGGTTCTGAACAGGTTGAGAACCAGTTTTATGTTAAGTTAACCAATAGCTCACATCGTCGCAAG
CGT
>CK_CH_GD_HZ_LH-LZL_20221227.seq
ATGTTGGGGAAGTCACTGTTCTTAATGATCATTTTGTGTGCACTATGTAGTGCAAATTTGTTTGATTTAGCCAA
TAATTATGTGTACTACTACCAAAGTGCCTTTAGGCCTCCAAATGGATGGCACTTGCATGGGGGTGCTTATGCA
GTAGTGAATTCTACTATTAAATATAACAATGCAGGCTCCGCTAGTGCGTGTTCTGTAGGTGTTCTCTTTAATTAT
TCTAACGGAAATGATGTTGGTTATAATAATAGTGCTTCTTCTGTAGCCATGACAGCACCATTGTCTGGTATGTC
TTGGTCTAAAGAAGAATTTTGTACTGCCCACTGTAACTTTTCGGATATTACAGTGTTTGTTACACATTGTTATG
CACAATCTTGTCCTTTAACGGGTCAGTTAGGTAAGGGCCATATTCGTATTTCTGCTATGAGAAATGGTTCTCTA
TTTTATAATTTAACAGTTGGTGTATCCCAATACCCTAAATTTAAATCGCTTCAATGTGTTAACAACCTCACTTCT
GTTTATCTAAATGGTGACCTTGTTTTTACTTCTAATGAAAGCACTAATGTTATAGGTGCTGGTGTGTACTTTAA
AGCAGGTGGGCCTATAACCTACAAAATTATGAGAGAATTTAAGGTTTTGGCTTATTTTTTAAATGGCACTGCG
CAAGATGTAATTTTGTGTGATGACACACCGAGAGGCTTGCTTGCATGTCAATATAATACTGGTAATCTTTCAGA
TGGGTTTTATCCTTTTACTAATTCTAGTTTAGTTAAGGAAAAGTTTATTGTTTATCGTCAGAATAGTGTCAATAC
CACTCTTACCTTAACTAACTATACTTTTTCTAATGAGACTAATGCCCCTCCTAATTCAGGTGATGTTCATTCTATT
CCAACTTATCAAACACAAACAGCTCAGAGTGGTTATTACAATTTTAATTTATCATTTCTGAGTAGTTTTGTGTAT
AAAGAGTCTAATTACATGTACGGGTCTTATCACCGTGCATGCAATTTTAGATTAGAAAATATTAATAATGGCTT
GTGGTTTAATTCACTGTCAGTTACGCTAGCTTATGGACCACTTCAAGGTGGGTGTAAGCAGTCAGTCTTTAGT
GGTAGAGCCACTTGTTGTTATGCTTATTCATATAATGGTCCTCACCTCTGTAAGGGTGTTTACAGCGGCGAGTT
ACAAAAAAGTTTTGAATGTGGATTGTTGGTTTATGTTACTAAGCGTGATGGCTCTCGTATACAAACAGCCACC
GTTCCACCAGTTATAACTCAACACAATTATAATAATATTACTTTAAATAAGTGTGTTGACTATAATATATATGGCA
GAGTAGGCCAAGGTTTTATTACTAATGTGACTGATTCTGCTGCTAATTTTAGTTATTTAGCAGATGGTGGGTTA
GCTATTTTAGATACTTCGGGTGCCATAGATGTTTTTGTTGTACAGGGCAGCTATGGTCTTAATTATTACAAGGT
TAATCCTTGTGAAGATGTTAACCAACAGTTTGTAGTGTCTGGTGGCAACATAGTTGGCATGCTTACTTCTAGA
AATGAAACAGGTTCTGAACAGGTTGAGAACCAGTTTTATGTTAAGTTAACCAATAGCTCACATCGTCGCAAG
CGT
>CK_CH_JX_GZ_XL-HXHZS_20220106.seq
ATGTTGGGCAAACCGCTTTTACTAGTGACTCTTTGGTATGCACTATGTAGTGCTTTGCTTTATGATAAAAATAC
TTACGTTTACTACTACCAAAGTGCCTTTAGGCCTGGTCAAGGTTGGCATCTACATGGGGGTGCTTATGCAGTA
GATAAGGTTTTTAATGGAACCAACAATGCAGTCAGTGTATCTGATTGCACTGCTGGTACTTTTTATGAAAGCT
ATAATATTTCTGCTGCTTCTGTAGCCATGACAGTACCACCTGCTGGTATGTCTTGGTCAGTTGCACAGTTTTGT
ACAGCTCATTGTAACTTCTCAGACTTTACAGTGTTTGTTACGCATTGTTTTAAAAGTCAACAAGGTAGTTGTC
CATTGACAGGTATGATTCCTCAGAATCATATTCGTATTTCTGCTATGAGATCTGGATTTTTGTTTTATAATTTAAC
AGTTAGCGTATCTAAATACCCTAAATTTAAATCGCTTCAATGTGTTGGCAATTCTACATCTGTCTATTTAAATGG
TGATCTTGTTTTCACTTCTAATGAAACAACTCACGTTACGGGTGCAGGCGTTTATTTTAAAAGTGGTGGGCCT
GTAACTTATAAAGTTATGAAAGAAGTTAAAGCCCTAGCCTACTTTATTAATGGTACCGCACAAGAGGTTATTTT
ATGTGATAACTCACCTAGAGGTTTGCTTGCATGTCAGTATAACACTGGTAATTTTTCAGATGGATTCTACCCTT

TTACTAATTCTTCTTTAGTTAAGGATAGGTTTATTGTATATCGAGAAAGTAGCACTAACACTACTTTAGAGTTAA
CTAATTTCACTTTTACTAATGTAAGTAATGCTTCTCCTAATTCAGGTGGCGTTGATACTTTCCAATTATATCAAA
CACATACTGCTCAGGATGGTTATTATAATTTTAATTTATCATTTCTGAGTAGTTTTGTGTATAAACCATCTGATTT
TATGTATGGGTCATACCACCCAAATTGTAATTTTAGACCAGAGAATATTAATAATGGCTTATGGTTTAATTCATT
ATCTGTGTCACTTACTTACGGACCCATTCAAGGTGGTTGTAAGCAATCTGTTTTTAGTAATAAAGCAACTTGTT
GCTATGCTTATTCTTACCGAGGTCCTACTAGATGTAAGGGTGTTTATAGAGGGGAGCTAACGCAATACTTTGA
ATGTGGACTTCTAGTTTATGTAACTAAGAGTGATGGCTCTCGTATACAAACTAGAAGTGAACCACTGGTGTTA
ACTCAATATAATTATAACAACATTACTTTAAATAAGTGTGTTGAGTATAATATATATGGTAGAGTTGGTCAAGGT
TTTATTACTAATGTAACTGAAGCAACTGCTAATTATAGTTATCTAGCAGATGGTGGTTTAGCTATTTTAGATACT
TCAGGAGCCATAGACATATTTGTTGTTCGAGGTGCATATGGTCTTAATTATTATAAGGTTAATCCCTGTGAAGA
TGTTAACCAACAGTTTGTAGTGTCTGGTGGCAATTTAGTTGGCATTCTTACATCTCATAATGAAACAGATTCTG
AATTTATTGAGAACCAGTTTTACATCAAACTCACTAACGGAACACGTCGCTCTAGACGT
>CK_CH_GX_WM_LY-HZH_20220321.seq
ATGTTGGGGAAGTCACTGTTGTTAGTGACCATTTTGTGTGCACTATGTAGTGCAAATTTGTTTGATTCTGGTA
ATTATGTGTACTATTACCAAAGTCAATTTAGGCCTTCAGGTGGATGGCATGTGCACGGGGGCGCCTATGCAGT
AGTGAATTCTACTTCTAAAAGCAACAATGCAGGCAATGCTAATGAGTGTTCTGTAGGTGTTCTTTTTAATTATA
GTAACGGAAATGACGTTGGATATAATAATAGTGCTGCTTCCATAGCCATGACAGCACCGCCTAGTGGTATGTC
TTGGTCTAAATCAGAATTTTGTACTGCCCACTGTAACTTTTCGGATTTTACAGTGTTTGTTACACATTGTTATGC
ACAATCTTGCCCTTTAACAGGTAAGATAGAGCAGAACCACATTCGTATTTCTGCTATGAGAAATGGTTCTCTAT
TTTATAATTTAACAGTTAGTACACTTAAGTACCCTAACTTTAAATCGTTTCAATGTGTTAACAACTTCACTTCTG
TTTATTTAAATGGTGATCTTGTTTTTACTTCTAATGAAACCACTGATGTTAAAGGTGCTGGTGTGTATTTTAAA
GCAGGTGGGCCTATATCCTATAAAGTTATGAAAGAATTTAAGGTTCTTGCTTACTTTGTTAATGGTACAGCAC
AAGATGTAATTTTGTGCGACAAGTCCCCCAAGGGTTTGCTAGCTTGTCAATATAACACTGGCAATTTTTCAGA
TGGCTTTTATCCTTTTACTAATACTACTTTAGTTAGGGAAAAATTCATCGTATATCGCGAAAGTAGTGTTAATAC
TACTCTGGCGTTAACTAATCTCACTTTTAATAATGAAAGTAATGCACAGCCTAATAGTGGTGGTGTTAATACTT
TTCATCTATATCAAACACAAACAGCTCAGAGTGGTTATTATAATTTTGATTTGTCATTTCTGAGTCAGTTTGTGT
ATAAGGCAAGTGATTTTATGTATGGGTCGTACCACCCTAGTTGTTCTTTTAGACCAGAAACCATTAATAGTGG
TTTGTGGTTTAATTCTTTGTCAGTTTCTCTAGCTTACGGACCACTTCAAGGTGGGTGTAAGCAGTCAGTTTTT
AGTGGTAGGGCAACGTGTTGCTATGCTTATTCTTACAATGGCCCGATAGCCTGTAAAGGTGTCTATTCAGGCG
AGTTAAAGACTAATTTTGAATGTGGATTGCTGGTTTATGTTACTAAGAGTGATGGCTCTCGTATACAGACTAG
AACAGAGCCCTTAGTATTAACGCAACACAATTATAATAATATTACTTTAGATAAGTGTGTTGACTATAATATATAT
GGCAGAGTAGGCCAAGGTTTTATTACTAATGTGACTGATTCTGCTGCTAATTTTGGTTATTTAGCAGATGGTG
GGTTAGCTATTTTAGATACTTCGGGTGCCATAGATGTCTTTGTTGTACAGGGCAGCTATGGTCTTAATTATTAC
AAGGTCAATCCTTGTGAAGATGTTAATCAACAGTTTGTAGTGTCTGGTGGCAATATAGTCGGCATTCTTACTT
CTAGAAATGAAACAGGTTCTGAACAGGTTGAGAACCAGTTTTATGTTAAATTAACCAATAGCCCACATCGTC
GTAGGCGT
>CK_CH_CQ_BS_DK-WGC_20220324.seq
ATGTTGGGGAAGTCACTGTTTTTAGTGACCATTTTGTGTGCACTATGTAGTGCAAATTTATTCGATCCTGCTAA
TACTTATGTGTACTACTACCAAAGTGCCTTTAGGCCTCCAAATGGATGGCACTTACAAGGGGGTGCTTATGCA
GTAGTCAATTCCACTAATTATACTAATAATGCCGGTTCTGCACAACATTGCACTGTTGGTGTTATTAAGGACGT
CTATAATCAAAGTGCGGCTTCCATAGCTATGACAGCACCTCTTCAGGGTATGGCTTGGTCTAAGTCACAATTT
TGTAGTGCACACTGTAACTTTTCTGAAATTACAGTTTTTGTCACACATTGTTATAGTAGTGGTAGCGGGTCTTG
TCCTATAACAGGCATGATTGCACGTGATCATATTCGTATTTCTGCAATGAAAAATGGTACTTTATTTTATAATTTA
ACAGTTAGCGTATCTAAATACCCTAATTTTAAATCTTTTCAATGCGTTAATAATCTCACATCTGTTTATCTAAATG

GTGATCTTGTTTTTACTTCCAACAAAACTACTGATGTTACGTCAGCAGGTGTGTATTTTAAAGCAGGTGGACC
TGTAAATTATAGTATTATGAAAGAATTTAAGGTTCTTGCTTACTTTGTTAATGGTACAGCACAAGATGTAATTTT
GTGCGACAATTCCCCCAAGGGTTTGCTAGCTTGTCAATATAACACTGGCAATTTTTCAGATGGCTTTTATCCTT
TTACTAATAGTACTTTAGTTAGGGAAAAGTTCATCGTATATCGCGAAAGTAGTGTTAATACTACTCTGGCGTTA
ACTAATTTCACTTTTACTAATGTAAGTAATGCACAGCCTAATAGTGGTGGTGTTAATACTTTTCATCTATATCAA
ACACAAACAGCTCAGAGTGGTTATTATAATTTTAATTTGTCATTTCTGAGTCAGTTTGTGTATAAGGCAAGTGA
TTTTATGTATGGGTCCTACCACCCTAGTTGTTCTTTTAGACCAGACACCATTAATAATGGTTTGTGGTTTAATTC
TTTGTCAGTTTCTCTAGCTTACGGACCACTTCAAGGTGGGTGTAAGCAGTCAGTTTTTAGTGGTAGGGCAAC
GTGTTGCTATGCCTACTCTTACAATGGCCCGATAGCCTGTAAAGGTGTTTATTCAGGCGAATTACGGACTAAT
TTTGAATGTGGATTGCTGATCTATGTTACTAAGAGTGATGGTTCTCGTATACAGACTAGAACAGAGCCCTTAG
TATTAACGCAACACAATTATAATAATATTACTTTAGATAAGTGTGTTGACTATAATATATATGGCAGAGTAGGCC
AAGGTTTTATTACTAATGTGACTGATTCTGCTGCTAATTTTAGTTATTTAGCAGATGGTGGGTTAGCTATTTTA
GATACTTCGGGTGCCATAGATGTCTTTGTTGTACAGGGCAGCTATGGTCTTAATTATTACAAGGTCAATCCTTG
TGAAGATGTTAACAAACAGTTTGTAGTGTCTGGTGGCAATATAGTTGGCATTCTTACTTCTACAAATGAAACA
GGTTCTGAACAGGTTGAGAACCAGTTTTATGTTAAGTTAACCAATAGCTCACATCGTCGCAGGCGT
>CK_CH_ZJ_TZ_LH-QXC_20220114.seq
ATGTTGGGGAAGTCACTGTTTTTAGTGACCATTTTGTGTGCACTATGTAGTGCAAATTTATTCGATCCTGCTAA
TACTTATGTGTACTACTACCAAAGTGCCTTTAGGCCTCCAAATGGATGGCACCTACAAGGGGGTGCTTATGCA
GTAGTCAATTCCACTAATTATACTAATAATGCCGGTTCTGCACAACATTGCACTGTTGGTGTTATTAAGGACGT
CTATAATCAAAGTGCGGCTTCCATAGCTATGACAGCACCTCTTCAGGGTATGGCTTGGTCTAAGTCACAATTT
TGTAGTGCACACTGTAACTTTTCTGAAATTACAGTTTTTGTCACACATTGTTATAGTAGTGGTAGCGGGTCTTG
TCCTATAACAGGCATGATTGCACGTGATCATATTCGTATTTCTGCAATGAAAAATGGTACTTTATTTTATAATTTA
ACAGTTAGCGTATCTAAATACCCTAATTTTAAATCTTTTCAATGCGTTAATAATCTCACATCTGTTTATCTAAATG
GTGATCTTGTTTTTACTTCCAACAAAACTACTGATGTTACGTCAGCAGGTGTGTATTTTAAAGCAGGTGGACC
TGTAAATTATAGTATTATGAAAGAATTTAAGGTTCTTGCTTACTTTGTTAATGGTACAGCACAAGATGTAATTTT
GTGCGACAATTCCCCCAAGGGTTTGCTAGCTTGTCAATATAACACTGGCAATTTTTCAGATGGCTTTTATCCTT
TTACTAATAGTACTTTAGTTAGGGAAAAGTTCATCGTATATCGCGAAAGTAGTGTTAATACTACTCTGGCGTTA
ACTAATTTCACTTTTACTAATGTAAGTAATGCACAGCCTAATAGTGGTGGTGTTAATACTTTTCATCTATATCAA
ACACAAACAGCTCAGAGTGGTTATTATAATTTTAATTTGTCATTTCTGAGTCAGTTTGTGTATAAGGCAAGTGA
TTTTATGTATGGGTCCTACCACCCTAGTTGTTCTTTTAGACCAGACACCATTAATAGTGGTTTGTGGTTTAATTT
TTTGTCAGTTTCTCTAGCTTACGGACCACTTCAAGGTGGGTGTAAGCAGTCAGTTTTTAGTGGTAGGGCAAC
GTGTTGCTATGCCTACTCTTACAATGGCCCGATAGCCTGTAAAGGTGTTTATTCAGGCGAATTACGGACTAAT
TTTGAATGTGGATTGCTGATTTATGTTACTAAGAGTGATGGTTCTCGTATACAGACTAGAACAGAGCCCTTAG
TATTAACGCAACACAATTATAATAATATTACTTTAGATAAGTGTGTTGACTATAATATATATGGCAGAGTAGGCC
AAGGTTTTATTACTAATGTGACTGATTCTGCTGCTAATTTTAGTTATTTAGCAGATGGTGGGTTAGCTATTTTA
GATACTTCGGGTGCCATAGATGTCTTTGTTGTACAGGGCAGCTATGGTCTTAATTATTACAAGGTCAATCCTTG
TGAAGATGTTAACAAACAGTTTGTAGTGTCTGGTGGCAATATAGTTGGCATTCTTACTTCTAGAAATGAAACA
GGTTCTGAACAGGTTGAGAACCAGTTTTATGTTAAGTTAACCAATAGCTCACATCGTCGCAGGCGT
>CK_CH_GD_QY_YF_20220111.seq
ATGTTGGGGAAGTCACTGTTTTTAGTGACCATTTTGTGTGCACTATGTAGTGCAAATTTGTTTGATTCTGCCA
ATAATTATGTGTACTACTACCAAAGTGCCTTTAGGCCTCCAAATGGATGGCATCTGCAAGGGGGTGCTTATGC
AGTAGTGAATTCTACTAATTATACTAATAATGCCGGTTTTGCAAGTGAGTGCACTATTGGTGTTATTAAGGACG
TCTATAATCAAAGTGCGGCTTCCATAGCTATGACAGCACCTCCTCAGGGTATGGCTTGGTCTAAGTCACAATT
TTGTAGTGCACACTGTAACTTTTCTGAAATTACAGTTTTTGTCACACATTGTTATGGTAGTGGTGCAGGTTCTT

GCCCTATAACAGGCATGATTGCACGTGATCATATTCGTATTTCTGCAATGAAAAATGGTTCTTTATTTTATAATT
TAACAGTTAGCGTATCTAAATACCCTACGTTTAAATCTTTTCAATGTGTTAACAATTTCACATCTGTTTATCTAAA
TGGTGATCTTGTTTTTACTTCTAACAAAACTGCTGATGTTACGTCAGCAGGTGTGTATTATAAAGCAGGTGGA
CCCGTAAATTATAGTGTTATGAAAGAATTTAAGGTTCTTGCTTATTTTGTTAATGGTACAGCACAAGATGTAAT
TTTGTGTGACAATTCCCCCAAGGGTTTGCTGGCTTGTCAATATAACACTGGCAATTTTTCAGATGGCTTTTATC
CTTTTACTAATACTACTTTAGTTAGGGAAAAGTTCATTGTATATCGTGAAAGTAGTGTCAATACTACTCTGACG
TTAACTAATTTCACTTTTACTAATGTAAGTAATGCACAGCCTAATAGTGGTGGTGTTAATACTTTTCATTTATATC
AAACACAAACAGCTCAGAGTGGTTATTATAATTTTAATTTGTCATTTCTGAGTCAGTTTGTGTATAAGGCAAGT
GATTTTATGTATGGGTCTTATTATCCTAGGTGTTCTTTTAGACCAGAAACCATTAATAGTGGTTTGTGGTTTAAT
TCCTTGTCAGTTTCTCTTACTTATGGACCCCTACAGGGAGGGTGTAAGCAATCTGTTTTTAATGGTAAGGCAA
CGTGTTGTTATGCCTACTCTTATAATGGCCCAAGAGCATGTAAAGGTGTTTATTCAGGTGAATTAAGCAAGAC
TTTTGAATGTGGATTGCTGGTTTATGTTACTAAGAGTGATGGCTCTCGTATACAAACTAGAACGGAGCCCTTA
GTATTAATGCAACACAATTATAATAATATTACTTTAGATAAGTGTGTTGACTATAATATATATGGCAGAGTAGGC
CAAGGTTTTATTACTAATGTGACTGATTCTGCTGCTAATTTTAGTTATTTAGCAGATGGTGGGTTAGCTATTTTA
GACACTTCGGGTGCCATAGATGTCTTTGTTGTACAGGGCAGCTATGGTCTTAATTATTACAAGGTCAATCCTT
GTGAAGATGTTAACCAACAGTTTGTAGTGTCTGGTGGCAATATAGTTGGCATTCTTACTTCTAGAAATGAAAC
AGGTTCTGAACAGGTTGAGAACCAGTTTTATGTTAAGTTAACCAATAGCTCACATCGTCGCAGGCGT
>CK_CH_ZJ_TZ_LH-WYY_20220420.seq
ATGTTGGGGAAGTCACTGTTTTTAGTGACCATTTTGTGTGCACTATGTAGTGCAAATTTGTTTGATTTTGCCA
ATAATTATGTGTACTACTACCAAAGTGCCTTTAGGCCTTCAAATGGATGGCATTTGCAAGGGGGTGCTTATGC
AGTAGTGAACTCTACTAATTATACTAGTAATGCCGGTTCTGCAAGTGAGTGCACTGTTGGTATTATTAAGGAC
GTCTATAATCAAAGTGCTGCTTCCATAGCTATGACAGCACCTCCTCAGGGTATGGCTTGGTCTAAGGCACAAT
TTTGTAGTGCACACTGTAACTTTTCTGAAATTACAGTCTTTGTCACACATTGTTATAGTAGTGGTGCAGGGTCT
TGCCCTATAACAGGCATGATTGAACGTGGTCATATTCGTATTTCTGCAATGAAAAATGGTTCTTTATTTTATAAC
TTAACAGTTAGTGTATCTAAATACTCTAGGTTTAAGTCTTTTCAATGTGTTAACAACTTCACATCTGTCTATCTA
AATGGTGATCTTGTTTTTACTTCCAACAAAACTACTGATGTTACGTCAGCAGGTGTGTATTTTAAAGCAGGTG
GACCTGTAAATTATAGTGTTATGAAAGAATTTAAGGTTCTTGCTTATTTTGTTAATGGTACAGCACAAGATGTA
ATTTTGTGTGACAAGTCCCCCAAGGGTTTGCTAGCTTGTCAATATAACACTGGCAATTTTTCAGATGGCTTTT
ATCCTTTTACTAATACTACTTTAGTTAGGGAAAAGTTCATCGTATATCGTGAAAGTAGTGTTAATACTACTCTGG
CGTTAACTAATTTCACTTTTACTAATGTAAGTAATGCACAGCCTAATAGTGGTGGTGTTAATACTTTTCATCTAT
ATCAAACACAAACAGCTCAGAGTGGTTATTATAATTTTAATTTGTCATTTCTGAGTCAGTTTGTGTATAAGGCA
AGTGATTTTATGTATGGGTCTTATCATCCTAGGTGTTCTTTTAGACCAGAAACCATTAATAGTGGTTTATGGTTT
AATTCCTTGTCAGTTTCTCTTACTTATGGACCCCTACAGGGAGGGTGTAAGCAATCTGTTTTTAGTGGTAAGG
CAACGTGTTGTTATGCCTACTCTTATAATGGCCCTAGGGCATGTAAAGGTGTTTATTCAGGTGAATTAAGCAA
GACTTTTGAATGTGGATTGCTGGTTTATGTTACTAAGAGTGATGGCTCTCGTATACAAACTAGAACAGAGCCC
TTAGTATTAACGCAACACAATTATAATAATGTTACTTTATATAAGTGTGTTGACTATAATATATATGGCAGAGTAG
GCCAAGGTTTTATTACTAATGTGACTGATTCTGCTGCTAATTTTAGTTATTTAGCAGATGGTGGGTTAGCTATT
TTAGATACTTCGGGTGCCATAGATGTCTTTGTTGTACAGGGCAGCTATGGTTCAAATTATTACAAGGTCAATCC
TTGTGAAGATGTTAACCAACAGTTTGTAGTGTCTGGTGGTAATATAGTTGGCATTCTTACTTCTAGAAATGAA
ACAGGTTCTGAACAGGTTGAGAACCAGTTTTATGTTAAGTTAACCAATAGCTCACATCGTCATAGGCGT
>CK_CH_SD_RZ_JR-7_20220214.seq
ATGTTGGGGAAGTCACTGTTTTTAGTGACCATTTTGTGTGCACTATGTAGTGCAAATTTGTTTGATTATGTGTA
CTACTACCAAAGTGCCTTTAGGCCTCCAAATGGATGGCATTTGCAAGGGGGTGCTTATGCAGTAGTGAATTC
TACTAATTATACTAGTAATGCCGGTTCTGCAAGTGAGTGCACTGTTGGTATTATTAAGGACGTCTATAATCAAA

GTGCGGCTTCCATAGCTATGACAGCACCCTCTCAGGGTATGGCTTGGTCTAAGTCACAATTTTGTAGTGCACA
CTGTAACTTTTCTGAAATTACAGTTTTCGTCACACATTGTTATAGTAGTGGTACAGGGTCTTGCCCTATAACAG
GCATGATTGCACGTGATCATATTCGTATTTCTGCAATGAAAAATGGTTCTTTATTTTATAACTTAACAGTTAGCG
TATCTAAATACTCTAGGTTTAAGTCTTTTCAATGTGTTAACAACTTCACATCTGTCTATCTAAATGGTGATCTTGT
TTTTACTTCCAACAAAACTACTGATGTTACGTCAGCAGGTGTGTATTTTAAAGCAGGTGGACCTGTAAATTAT
AGTGTTATGAAAGAATTTAAGGTTCTTGCTTATTTTGTTAATGGTACAGCACAAGATGTAATTTTGTGTGACA
AGTCCCCCAAGGGTTTGCTAGCTTGTCAATATAACACTGGCAATTTTTCAGATGGCTTTTATCCTTTTACTAAT
ACTACTTTAGTTAGGGAAAAGTTCATTGTATATCGTGAAAGTAGTGTTAATACTACTCTGGCGTTAACTAATTT
CACTTTTACTAATGTAAGTAATGCACAGCCTAATAGTGGTGGTGTTAATACTTTTCATCTATATCAAACACAAAC
AGCTCAGAGTGGTTATTATAATTTTAATTTGTCATTTCTGAGTCAGTTTGTGTATAAGGCAAGTGATTTTATGTA
TGGGTCTTATCATCCTAGGTGTTCTTTTAGACCAGCAACCATTAATAGTGGTTTATGGTTTAATTCCTTGTCAG
TTTCTCTTACTTATGGACCCCTACAGGGAGGGTGTAAGCAATCTGTTTTTAGTGGTAAGGCAACGTGTTGTTA
TGCCTACTCTTATAATGGCCCTAGGGCATGTAAAGGTGTTTATTCAGGTGAATTAAGTAAGACTTTTGAATGT
GGATTGCTGGTTTATGTTACTAAGAGTGATGGCTCTCGTATACAAACTAGAACAGAGCCCTTAGTATTAACGC
AACACAATTATAATAATGTTACTTTAGATAAGTGTGTTGACTATAATATATATGGCAGAGTAGGCCAAGGTTTTA
TTACTAATGTGACTGATTCTGCTGCTAATTTTAGTTATTTAGCAGATGGTGGGTTAGCTATTTTAGATACTTCGG
GTGCCATAGATGTCTTTGTTGTACAGGGCAGCTATGGTTCTAATTATTACAAGGTCAATCCTTGTGAAGATGTT
AACCAACAGTTTGTAGTGTCTGGTGGTAATATAGTTGGCATTCTTACTTCTAGAAATGAAACAGGTTCTGAAC
AGGTTGAGAACCAGTTTTATGTTAAGTTAACCAATAGCTCACATCGTCATAGGCGT
>CK_CH_SD_RZ_JR-2_20220214.seq
ATGTTGGGGAAGTCACTGTTTTTAGTGACCATTTTGTGTGCACTATGTAGTGCAAATTTGTTTGATTTTGCCA
ATAATTATGTGTACTACTACCAAAGTGCCTTTAGGCCTCCAAATGGATGGCATTTGCAAGGGGGTGCTTATGC
AGTAGTGAATTCTACTAATTATACTAGTAATGCCGGTTCTGCAAGTGAGTGCACTGTTGGTATTATTAAGGACG
TCTATAATCAAAGTGCGGCTTCCATAGCTATGACAGCACCCTCTCAGGGTATGGCTTGGTCTAAGTCACAATT
TTGTAGTGCACACTGTAACTTTTCTGAAATTACAGTTTTCGTCACACATTGTTATAGTAGTGGTACAGGGTCTT
GCCCTATAACAGGCATGATTGCACGTGATCATATTCGTATTTCTGCAATGAAAAATGGTTCTTTATTTTATAACT
TAACAGTTAGCGTATCTAAATACTCTAGGTTTAAGTCTTTTCAATGTGTTAACAACTTCACATCTGTCTATCTAA
ATGGTGATCTTGTTTTTACTTCCAACAAAACTACTGATGTTACGTCAGCAGGTGTGTATTTTAAAGCAGGTGG
ACCTGTAAATTATAGTGTTATGAAAGAATTTAAGGTTCTTGCTTATTTTGTTAATGGTACAGCACAAGATGTAA
TTTTGTGTGACAAGTCCCCCAAGGGTTTGCTAGCTTGTCAATATAACACTGGCAATTTTTCAGATGGCTTTTAT
CCTTTTACTAATACTACTTTAGTTAGGGAAAAGTTCATTGTATATCGTGAAAGTAGTGTTAATACTACTCTGGC
GTTAACTAATTTCACTTTTACTAATGTAAGTAATGCACAGCCTAATAGTGGTGGTGTTAATACTTTTCATCTATAT
CAAACACAAACAGCTCAGAGTGGTTATTATAATTTTAATTTGTCATTTCTGAGTCAGTTTGTGTATAAGGCAA
GTGATTTTATGTATGGGTCTTATCATCCTAGGTGTTCTTTTAGACCAGAAACCATTAATAGTGGTTTATGGTTTA
ATTCCTTGTCAGTTTCTCTTACTTATGGACCCCTACAGGGAGGGTGTAAGCAATCTGTTTTTAGTGGTAAGGC
AACGTGTTGTTATGCCTACTCTTATAATGGCCCTAGGGCATGTAAAGGTGTTTATTCAGGTGAATTAAGTAAG
ACTTTTGAATGTGGATTGCTGGTTTATGTTACTAAGAGTGATGGCTCTCGTATACAAACTAGAACAGAGCCCT
TAGTATTAACGCAACACAATTATAATAATGTTACTTTAGATAAGTGTGTTGACTATAATATATATGGCAGAGTAG
GCCAAGGTTTTATTACTAATGTGACTGATTCTGCTGCTAATTTTAGTTATTTAGCAGATGGTGGGTTAGCTATT
TTAGATACTTCGGGTGCCATAGATGTCTTTGTTGTACAGGGCAGCTATGGTTCTAATTATTACAAGGTCAATCC
TTGTGAAGATGTTAACCAACAGTTTGTAGTGTCTGGTGGTAATATAGTTGGCATTCTTACTTCTAGAAATGAA
ACAGGTTCTGAACAGGTTGAGAACCAGTTTTATGTTAAGTTAACCAATAGCTCACATCGTCATAGGCGT
>CK_CH_SD_XXW-6#_20220118.seq
ATGTTGGGGAAGTCACTGTTTTTAGTGACCATTTTGTGTGCACTATGTAGTGCAAATTTGTTTGATTCTGCCA

ATAATTATGTGTACTACTACCAAAGTGCCTTTAGGCCTCCAACTGGATGGCATTTGCAAGGGGGTGCTTATGC
AGTAGTGAATTCTACTAATTATACTAGTAATGCCGGTTCTGCAAGTGGGTGCACTGTTGGTATTATTAAGGAC
GTCTATAATCAAAGTGCGGCTTCCATAGCTATGACAGCACCTCCTCAGGGTATGGCTTGGTCTAAGTCACAAT
TTTGTAGTGCACACTGTAACTTTTCTGAAATTACAGTTTTTGTCACACATTGTTATAGTAGTGGTGCAGGGTCT
TGCCCTATAACAGGCATGATTGCACGTGACCATATTCGTATTTCTGCAATGAAAAATGGTTCTTTATTTTATAAC
TTAACAGTTAGCGTATCTAAATACCCTAGATTTAAGTCTTTTCAATGTGTTAACAACTTCACATCTGTTTATTTA
AATGGTGATCTTGTTTTTACTTCTAATAAAACTACTGATGTTACGTCAGCAGGTGTGTATTTTAAAGCAGGTG
GACCTGTAAATTATAGTGTTATGAAAGAATTTAAGGTTCTTGCTTACTTTGTTAATGGTACAGCACAAGATGTA
ATTTTGTGTGACAATTCCCCTAAGGGTTTGCTAGCTTGTCAATATAACACTGGCAATTTTTCAGACGGCTTTTA
TCCTTTTACTAATAGTACTTTAGTTAGGGACAAGTTCATTGTCTATCGTGAAAGTAGTGTTAATACTACTTTGAC
GTTAACTAATTTCACTTTTACTAATGTAAGTACTGCACAGCCTAATAGTGGTGGTGTTAGTACTTTTCATCTATA
TCAAACACAAACAGCTCAGAGTGGTTATTATAATTTTAATTTGTCATTTCTGAGTCAGTTTGTGTATAAGGCAA
GTGATTTTATGTATGGGTCTTATCATCCTAGGTGTTCTTTTAGACCAGAAACCATTAATAGTGGTTTATGGTTTA
ATTCCTTGTCAGTTTCTCTTACTTATGGACCCCTACAGGGAGGGTGTAAGCAATCTGTTTTTAGTGGTAAGGC
AACGTGTTGTTATGCCTACTCTTATAATGGCCCTAGGGCATGTAAAGGTGTTTATTCAGGTGAATTAAGCAAG
ACTTTTGAATGTGGATTGCTGGTTTATGTTACTAAGAGTGATGGCTCTCGTATACAGACTAGAACAGAGCCCT
TAGTATTAACGCAACACAATTATAATAATATTACTTTAGATAAGTGTGTTGACTATAATATATATGGCAGAGTAG
GCCAAGGTTTTATTACTAATGTGACTGATTCTGCTGCTAATTCTAGTTATTTAGCAGATGGTGGGTTAGCTATT
TTAGATACTTCGGGTGCCATAGATGTCTTTGTTGTACAGGGCAGCTATGGTTTTAATTATTACAAGGTCAATCC
TTGTGAAGATGTTAATCAACAGTTTGTAGTGTCTGGTGGCAATATAGTTGGCATGCTTACTTCTAGAAATGAA
ACAGGTTCTGAACAGGTTGAGAACCAGTTTTATGTTAAGTTAACCAATAGCTCACATCGTCGTAGGCGT
>CK_CH_SX_DJ-TCmianshizi_20220117.seq
ATGTTGGGGAAGTCACTGTTTTTAGTGACCATTTTGTGTGCACTATGTAGTGCAAATTTGTTTGATCCTGCCA
ATAATTATGTGTACTACTACCAAAGTGCCTTTAGGCCTCCAAATGGATGGCATCTGCAAGGGGGTGCTTATGC
AGTAGTGAATTCTACTAATTATACTAGTAATGCCGGTTCTGCAAGTGAGTGCACTGTTGGTATTATTAAGGACG
TCTATAATCAAAGTGCGGCTTCCATAGCTATGACAGCACCTCCTCAGGGTATGGCTTGGTCTAAGTCACAATT
TTGTAGTGCACACTGTAACTTTTCTGAAATTACAGTCTTTGTCACACATTGTTATAGTAGTGGTGCAGGGTCTT
GCCCTATAACAGGCATGATTGCACGTGATCATATTCGTATTTCTGCAATGAAAAATGGTTCTTTATTTTATAACT
TAACAGTTAGCGTATCTAAATACTCTAGGTTTAAGTCTTTTCAATGTGTTAATAACCTCACATCTGTTTATTTAAA
TGGTGATCTTGTTTTTACTTCCAATAAAACTACTGATGTTACGTCAGCAGGTGTGTATTTTAAAGCAGGTGGA
CCTGTAAATTATAGTGTTATGAAAGAATTTAAGGTTCTTGCTTACTTTGTTAATGGTACAGCACAAGACGTAAT
TTTGTGTGACAATTCCCCTAAGGGTTTGCTAGCCTGTCAATATAGTACTGGCAATTTTTCAGATGGTTTCTATC
CTTTTACTAATAGTACTTTAGTTAGGGACAAGTTCATTGTCTATCGTGAAAGTAGTGTTAATACTACTTTGACG
TTAACTAATTTCACTTTTACTAATGTAAGTACTGCACAGCCTAATAGTGGTGGTGTTAGTACTTTTCATCTATAT
CAAACACAAACAGCTCAGAGTGGTTATTATAATTTTAATTTGTCATTTCTGAGTCAGTTTGTGTATAAGGCAA
GTGATTTTATGTATGGGTCTTATCATCCTAGGTGTTCTTTTAGACCAGAAACCATTAATAGTGGTTTATGGTTTA
ATTCCTTGTCAGTTTCTCTTACTTATGGACCTCTACAGGGAGGGTGTAAGCAATCCGTTTTTAGTGGTAAGGC
AACGTGTTGTTATGCCTACTCTTATAATGGCCCTAGGGCATGTAAAGGTGTTTATTCAGGTGAATTAAGCAAG
ACTTTTGAATGTGGATTGCTGGTTTATGTTACTAAGAGTGATGGCTCTCGTATACAAACTAGAACGGAGCCCT
TAGTATTAACGCAACACAATTATAATAATATTACTTTAGATAAGTGTGTTAATTATAATATATATGGCAGAGTTGG
CCAAGGTTTTATTACTAATGTGACTGATTCTGCTGCTAATTTTAGTTATTTAGCAGATGGTGGGTTAGCTATTTT
AGACACTTCGGGTGCCATAGATGTTTTTATTGCACAGGGCAGCTATGGTCCTAATTATTACAAGGTCAATCCT
TGTGAAGATGTTAACCAACAGTTTGTAGTGTCTGGTGGCAATATAGTTGGCATTCTTACTTCTAGAAATGAAA
CAGGTTCTGAACAGGTTGAGAACCAGTTTTATGTTAAGTTAACCAATAGCTCACATCGTCGTAGGCGT

>CK_CH_GD_KP_LH-NF_20220317.seq
ATGTTGGGGAAGTCACTGTTTTTAGTGACCATTTTGTGTGCACTATGTAGTGCAAATTTGTTTGATTCTGCCA
GTAATTATGTGTACTACTACCAAAGTGCCTTTAGGCCTCCAAATGGATGGCATCTGCAAGGGGGTGCTTATGC
AGTAGTGAATTCTATTAATTATACTAGTAATGCCGGTTCTGCAAGTGAGTGCACTGTTGGTATTATTAAGGACG
TCTATAATCAAAGTGTGGCTTCCATAGCTATGACAGCACCTCCTCAGGGTATGACTTGGTCTAAGTCACAATTT
TGTAGTGCACACTGTAACTTTTCTGAAACTACAGTCTTTGTCACACATTGTTATAGTAGTGGTGCAGGGTCTT
GCCCTATAACAGGCATGATTGCACGTGATCATATTCGTATTTCTGCAATGAAAAATGGTTCTTTATTTTATAACT
TAACAGTTAGCGTATCTAAATACTCTAGGTTTAAGTCTTTTCAATGTGTTAATAATCTCACATCTGTTTATTTAAA
TGGTGATCTTGTTTTTACTTCCAATAAAACTACTGATGTTACGTCAGCAGGTGTGTATTTTAAAGCAGGTGGA
CCTGTAAATTATAGTGTTATGAAAGAATTTAAGGTTCTTGCTTACTTTGTTAATGGTACAGCACAAGACGTAAT
TTTGTGTGACAATTCCCCTAAGGGTTTGCTAGCCTGTCAATATAACACTGGCAATTTTTCAGATGGCTTCTATC
CTTTTACTAATAGTACTTTAGTTAGGGACAAGTTCATTGTTTATCGTGAAAGTAGTGTTAATACTACTTTGACG
TTAAATAATTTCACTTTTACTAATGTAAGTACTGCACAGCCTAATAGTGGTGGTGTTAGTACTTTTCATCTATATC
AAACACAAACAGCTCAGAGTGGTTATTATAATTTTAATTTGTCATTTCTGAGTCAGTTTGTGTATAAGGCAAGT
GATTTTATGTATGGGTCTTATCATCCTAGGTGTTCTTTTAGACCTGAAACCATTAATAGTGGTTTATGGTTTAAT
TTTTTGTCAGTTTCTCTTACTTATGGACCCCTACAGGGAGGGTGTAAGCAATCTGTTTTTAGTGGTAAGGCAA
CGTGTTGTTATGCCTACTCTTATAATGGCCCTAGGGCATGTAAAGGTGTTTATTCAGGTGAATTAAGCAAGAC
TTTTGAATGTGGATTGCTGGTTTATGTTACTAAGAGTGATGGCTCTCGTATACAAACTAGAACAGAGCCCTTA
GTATTAACGCAACACAATTATAATAATGTTACTTTAGATAAGTGTGTTGACTATAATATATATGGCAGAGTAGGC
CAAGGTCTTATTACTAATGTGACTGATTCTGCTGCTAATTTTAGTTATTTAGCAGATGGTGGGTTAGCTATTTTA
GATACTTCGGGTGCCATAGATGTCTTTGTTGTACAGGGCAGCTATGGTTCTAATTATTACAAGGTCAATCCTTG
TGAAGATGTTAACCAACAGTTTGTAGTGTCTGGTGGTAATATAGTTGGCATTCTTACTTCTAGAAATGAAACA
GGTTCTGAACAGGTTGAGAACCAGTTTTATGTTAAGTTAACCAATAGCTCACATCGTCATAGGCGT
>CK_CH_SD_RZ_JR-4_20220214.seq
ATGTTGGGGAAGTCACTGTTTTTAGTGACCATTTTGTGTGCACTATGTAGTGCAAATTTGTTTGATCCTGCCA
ATAATTATGTGTACTACTACCAAAGTGCCTTTAGGCCTCCAACTGGATGGCATTTGCAAGGGGGTGCTTATGC
AGTAGTGAATTCTACTAATTATACTAGTAATGCCGGTTCTGCAAGTGAGTGCACTGTTGGTATTATTAAGGACG
TTTATAATCAAAGTGCGGCTTCTATAGCTATGACAGCACCTTCTCAGGGTATGGCTTGGTCTAAGTCACAATTT
TGTAGTGCACACTGTAACTTTTCTGAAATTACAGTCTTTGTCACACATTGTTATAGTAGTGCTGCAGGGTCTTG
CCCTATAACAGGCATGATTGCACGTGATCATATTCGTATTTCTGCAATGAAAAATGGTTCTTTATTTTATAACTT
AACAGTTAGCGTATCTAAATACTCTAGGTTTAAGTCTTTTCAATGTGTTAATAACCTCACATCTGTTTATTTAAA
TGGTGATCTTGTTTTTACTTCCAATAAAACTACTGATGTTACGTCAGCAGGTGTGTATTTTAAAGCAGGTGGA
CCTGTAAATTATAGTGTTATGAAAGAATTTAAGGTTCTTGCTTACTTTGTTAATGGTACAGCACAAGACGTAAT
TTTGTGTGACAATTCTCCTAAGGGTTTGCTAGCCTGTCAATATAGTACTGGCAATTTTTCAGATGGCTTCTATC
CTTTTACTAATAGTACTTTAGTTAGGGACAAGTTCATTGTCTATCGTGAAAGTAGTGTTAATACTACTTTGACG
TTAACTAATTTCACTTTTACTAATGTAAGTACTGCACAGCCTAATAGTGGTGGTGTTAGTACTTTTCATCTATAT
CAAACACAAACAGCTCAGAGTGGTTATTATAATTTTAATTTGTCATTTCTGAGTCAGTTTGTGTATAAGGCAA
GTGATTTTATGTATGGGTCTTATCATCCTAGGTGTTCTTTTAGACCAGAAACCATTAATAGTGGTTTATGGTTTA
ATTCCTTGTCAGTTTCTCTTACTTATGGACCCCTACAGGGAGGGTGTAAGCAATCTGTTTTTAGTGGTAAGGC
AACGTGTTGTTATGCCTACTCTTATAATGGCCCTAGGGCATGTAAAGGTGTTTATTCAGGTGAATTAAGCAAG
ACTTTTGAATGCGGATTGCTGGTTTATGTTACTAAGAGTGATGGCTCTCGTATACAAACTAGAACAGAGCCCT
TAGTATTAACGCAACACAATTATAATAATGTTACTTTAGATAAGTGTGTTGACTATAATATATATGGCAGAGTAG
GCCAAGGTTTTATTACTAATGTGACTGATTCTGCTGCTAATTTTAGTTATTTAGCAGATGGTGGGTTAGCTATT
TTAGATACTTCGGGTGCCATAGATGTCTTTGTTGTACAGGGCAGCTATGGTTCTAATTATTACAAGGTCAATCC

TTGTGAAGATGTTAACCAACAGTTTGTAGTGTCTGGTGGTAATATAGTTGGCATTCTTACTTCTAGAAATGAA
ACAGGTTCTGAACAGGTTGAGAACCAGTTTTATGTTAAGTTAACCAATAGCTCACATCGTCATAGGCGT
>CK_CH_SD_LY_GH-1_20220104.seq
ATGTTGGGGAAGTCACTGTTTTTAGTGACCATTTTGTGTGCACTATGTAGTGCAAATTTGTTTGATCCTGCCA
ATAATTATGTGTACTACTACCAAAGTGCCTTTAGGCCTCCAAGTGGATGGCATTTGCAAGGGGGTGCTTATGC
AGTAGTGAATTCTACTAATTATACTAGTAATGCCGGTTCTGCAAGTGGTTGCACTGTTGGTATTATTAAGGACG
TCTATAATCAAAGTGCGGCTTCCATAGCTATGACAGCACCTCCTCAGGGTATGGCTTGGTCTAAGTCACAATT
TTGTAGTGCACACTGTAACTTTTCTGAAATTACAGTCTTTGTCACACATTGTTTTAGTAGTGGTGCAGGGTCTT
GCCCTATAACAGGCATGATTGCACGTGATCATATTCGTATTTCTGCAATGAAAAATGGTTCTTTATTTTATAACT
TAACAGTTAGCGTATCTAAATACTCTAGGTTTAAGTCTTTTCAATGTGTTAATAACCTCACATCTGTTTATTTAAA
TGGTGATCTTGTTTTTACTTCCAATAAAACTACTGATGTTACGTCAGCAGGTGTGTATTTTAAAGCAGGTGGA
CCTGTAAATTATAGTGTTATGAAAGAATTTAAGGTTCTTGCTTACTTTGTTAATGGTACAGCACAAGACGTAAT
TTTGTGTGACAATTCCCCTAAGGGTTTGCTAGCCTGTCAATATAGTACTGGCAATTTTTCAGATGGCTTCTATC
CTTTTACTAATAGTACTTTAGTTAGGGACAAGTTCATTGTCTATCGTGAAAGTAGTGTTAATACTACTTTGACG
TTAACTAATTTCACTTTTACTAATGTAAGTACTGCACAGCCTAATAGTGGTGGTGTTAGTACTTTTCATCTATAT
CAAACACAAACAGCTCAGAGTGGTTATTATAATTTTAATTTGTCATTTCTGAGTCAGTTTGTGTATAAGGCAA
GTGATTTTATGTATGGTTCTTATCATCCTAGGTGTTCTTTTAGACCAGAAACCATTAATAGTGGTTTATGGTTTA
ATTCCTTGTCAGTTTCTCTTACTTATGGACCCCTACAGGGAGGGTGTAAGCAATCTGTTTTTAGTGGTAAGGC
AACGTGTTGTTATGCCTACTCTTATAATGGCCCTAGGGCATGTAAAGGTGTTTATTCAGGTGAATTAAGCAAG
ACTTTTGAATGTGGATTGCTGGTTTATGTTACTAAGAGTGATGGCTCTCGTATACAAACTAGAACAGAGCCCT
TAGTATTAACGCAACACAATTATAATAATGTTACTTTAGATAAGTGTGTTGACTATAATATATATGGCAGAGTAG
GCCAAGGTTTTATTACTAATGTGACTGATTCTGCTGCTAATTTTAGTTATTTAGCAGATGGTGGGTTAGCTATT
TTAGATACTTCGGGTGCCATAGATGTCTTTGTTGTACAGGGCAGCTATGGTTCTAATTATTACAAGGTCAATCC
TTGTGAAGATGTTAACCAACAGTTTGTAGTGTCTGGTGGTAATATAGTTGGCATTCTTACTTCTAGAAATGAA
ACAGGTTCTGAACAGGTTGAGAACCAGTTTTATGTTAAGTTAACCAATAGCTCACATCGTCATAGGCGT
>CK_CH_SD_LY_LN1chang-11_20220428.seq
ATGTTGGGGAAGTCACTGTTTTTAGTGACCATTTTGTGTGCACTATGTAGTGCAAATTTGTTTGATTCTGCCA
ATAATTATGTGTACTACTACCAAAGTGCCTTTAGGCCTCCAAATGGATGGCATTTGCAAGGGGGTGCTTATGC
AGTAGTGAATTCTACTAATTATACTAGTAATGCCGGTTCTGCAAGTGAGTGCACTGTTGGTATTATTAAGGACG
TCTATAATCAAAGTGCGGCTTCCATAGCTATGACAGCACCTCCTCAGGGTATGGCTTGGTCTAAGTCACAATT
TTGTAGTGCACACTGTAACTTTTCTGAAATTACAGTCTTTGTCACACATTGTTATAGTAGTGGCGCAGGGTCTT
GCCCTATAACAGGCATGATTGCACGTGATCATATTCGTATTTCTGCAATGAAAAATGGTTCTTTATTTTATAACT
TAACAGTTAGCGTATCTAAATACTCTAGGTTTAAGTCTTTTCAATGTGTTAATAACCTCACATCTGTTTATTTAAA
TGGTGATCTTGTTTTTACTTCCAATAAAACTACTGATCTTACGTCAGCAGGTGTGTATTTTAAAGCAGGTGGA
CCTGTAAATTATAGTGTTATGAAAGAATTTAAGGTTCTTGCTTACTTTGTTAATGGTACAGCACAAGACGTAAT
TTTGTGTGACAATTCCCCTAAGGGTTTGCTAGCCTGTCAATATAGTACTGGCAATTTTTCAGATGGCTTCTATC
CTTTTACTAATAGTACTTTAGTTAGGGACAAGTTCATTGTCTATCGTGAAAGTAGTGTTAATACTACTTTGACG
TTAACTAATTTCACTTTTACTAATGTAAGTACTGCACAGCCTAATAGTGGTGGTGTTAGTACTTTTCATCTATAT
CAAACACAAACAGCTCAGAGTGGTTATTATAATTTTAATTTGTCATTTCTGAGTCAGTTTGTGTATAAGGCAA
GTGATTTTATGTATGGGTCTTATCATCCTAGGTGTTCTTTTAGACCAGAAACCATTAATAGTGGTTTATGGTTTA
ATTCCTTGTCAGTTTCTCTTACTTATGGACCCCTACAGGGAGGGTGTAAGCAATCTGTTTTTAGTGGTAAGGC
AACGTGTTGTTATGCCTACTCTTATAATGGCCCTAGGGCATGTAAAGGTGTTTATTCAGGTGAATTAAGCAAG
ACTTTTGAATGTGGATTGCTGGTTTATGTTACTAAGAGTGATGGCTCTCGTATACAAACTAGAACAGAGCCCT
TAGTATTAACGCAACACAATTATAATAATGTTACTTTAGATAAGTGTGTTGACTATAATATATATGGCAGAGTAG

GCCAAGGTTTTATTACTAATGTGACTGATTCTGCTGCTAATTTTAGTTATTTAGCAGATGGTGGGTTAGCTATT
TTAGATACTTCGGGTGCCATAGATGTCTTTGTTGTACAGGGCAGCTATGGTTCTAATTATTACAAGGTCAATCC
TTGTGAAGATGTTAACCAACAGTTTGTAGTGTCTGGTGGTAATATAGTTGGCATTCTTACTTCTAGAAATGAA
ACAGGTTCTGAACAGGTTGAGAACCAGTTTTATGTTAAGTTAACCAATAGCTCACATCGTCATAGGCGT
>CK_CH_ZJ_TZ_LZJ_20220117.seq
ATGTTGGGGAAGTCACTGTTTTTAGTGACCATTTTGTGTGCACTATGTAGTGCAAATTTGTTTGATTTTGCCA
ATAATTATGTGTACTACTACCAAAGTGCCTTTAGGCCTTCAAATGGATGGCATTTGCAAGGGGGTGCTTATGC
AGTAGTGAATTCTACTAATTATACTAGTAATGCCGGTTCTGCAAGTGAGTGCACTGTTGGTATTATTAAGGACG
TCTATAATCAAAGTGCGGCTTCCATAGCTATGACAGCACCTCCTCAGGGTATGGCTTGGTCTAAGTCACAATT
TTGTAGTGCACACTGTAACTTTTCTGAAATTACAGTCTTTGTCACACATTGTTATAGTAGTGGTGCAGGGTCTT
GCCCTATAACAGGCATGATTGCACGTGATCGTATTCGTATTTCTGCAATGAAAAATGGTTCTTTATTTTATAATT
TAACAGTTAGCGTATCTAAATACTCTAGGTTTAAGTCTTTTCAATGTGTTAATAACCTCACATCTGTTTATTTAAA
TGGTGATCTTGTTTTTACTTCCAATAAAACTACTGATGTTACGTCAGCAGGTGTGTATTTTAAAGCAGGTGGA
CCTGTAAATTATAGTGTTATGAAAGAATTTAGGGTTCTTGCTTACTTTGTTAATGGTACAGCACAAGACGTAAT
TTTGTGTGACAATTCCCCTAAGGGTTTGCTAGCCTGTCAATATAGTACTGGCAATTTTTCAGATGGCTTCTATC
CTTTTACTAATAGTACTCTAGTTAGGGACAAGTTTATTGTCTATCGTGAAAGTAGTGTTAATACTACTTTGACGT
TAACTAATTTCACTTTTACTAATGTAAGTACTGCACAGCCTAATAGTGGTGGTGTTAGTACTTTTCATCTATATC
AAACACAAACAGCTCAGAGTGGTTATTATAATTTTAATTTGTCATTTCTGAGTCAGTTTGTGTATAAGGCAAGT
GATTTTATGTATGGGTCTTATCATCCTAGGTGTTCTTTTAGACCAGAAACCATTAATAGTGGTTTATGGTTTAAT
TCCTTGTCAGTTTCTCTTACTTATGGACCCCTACAGGGAGGGTGTAAGCAATCTGTTTTTAGTGGTAAGGCAA
CGTGTTGTTATGCCTACTCTTATAATGGCCCTAGGGCATGTAAAGGTGTTTATTCAGGTGAATTAAGCAAGAC
TTTTGAATGTGGATTGCTGGTTTATGTTACTAAGAGTGATGGCTCTCGTATACAAACTAGAACAGAGCCCTTA
GTATTAACGCAACACAATTATAATAATGTTACTTTAGATAAGTGTGTTGACTATAATATATATGGCAGAGTAGGC
CAAGGTTTTATTACTAATGTGACTGATTCTGCTGCTAATTTTAATTATTTAGCAGATGGTGGGTTAGCTATTTTA
GATACTTCGGGTGCCATAGATGTCTTTGTTGTACAGGGCATCTATGGTTCTAATTATTACAAGGTCAATCCTTG
TGAAGATGTTAACCAACAGTTTGTAGTGTCTGGTGGTAATATAGTTGGCATTCTTACTTCTAGAAATGAAACA
GGTTCTGAACAGGTTGAGAACCAGTTTTATGTTAAGTTAACCAATAGCTCACATCGTCATAGGCGT
>CK_CH_GX_GL_LY-CLY_20220321.seq
ATGTTGGGGAAGTCACTGTTTTTAGTGACCATTTTGTGTGCACTATGTAGTGCAAATTTGTTTGATTTGGCCA
ATAATTATGTGTACTACTACCAAAGTGCCTTTAGGCCTCCAAATGGATGGCATTTGCAAGGGGGTGCTTATGC
AGTAGTGAATTCTACTAATTATACTAGTAATGCCGGTTCTGCAAGTGAGTGCACTGTTGGTATTATTAAGGACG
TCTATAATCAAAGTGCGGCTTCCATAGCTATGACAGCACCTTCTCAGGGTATGGCTTGGTCTAAGTCACAATT
TTGTAGTGCACACTGTAACTTTTCTGAAATTACAGTTTTCGTCACACATTGTTATAGTAGTGGTACAGGGTCTT
GCCCTATAACAGGCATGATTGCACGTGATCATATTCGTATTTCTGCAATGAAAAATGGTTCTTTATTTTATAACT
TAACAGTTAGCGTATCTAAATACTCTAGGTTTAAGTCTTTTCAATGTGTTAACAACCTCACATCTGTTTATCTAA
ATGGTGATCTTGTTTTTACTTCTAACAAAACTACTGATGTTACGTCAGCAGGTGTGTATTTTAAAGCAGGTGG
ACCTGTAAATTATAGTGTTATGAAAGAATTTAAGGTTCTTGCTTATTTTGTTAATGGTACAGCACAAGATGTAA
TTTTGTGTGACAAGTCCCCCAAGGGTTTGCTAGCTTGTCAATATAACACTGGCAATTTTTCAGATGGCTTTTAT
CCTTTTACTAATACTACTTTAGTTAGGGAAAAGTTCATTGTATATCGTGAAAGTAGTGTTAATACTACTCTGGC
GTTAACTAATTTCACTTTTACTAATGTAAGTAATGCACAGCCTAATAGTGGTGGTGTTAATACTTTTCATTTATAT
CAAACACAAACAGCTCAGAGTGGTTATTATAATTTTAATTTGTCATTTCTGAGTCAGTTTGTGTATAAGGCAA
GTGATTTTATGTATGGGTCTTATTATCCTAGGTGTTCTTTTAGACCAGAAACCATTAATAATGGTTTGTGGTTTA
ATTCCTTGTCAGTTTCTCTTACTTATGGACCCCTACAGGGAGGGTGTAAGCAATCTGTTTTTAGTGGTAAGGC
AACGTGTTGTTATGCCTACTCTTATAATGGCCCTAGAGCATGCAAAGGTGTTTATTCAGGCGAATTAAGCAAG

ACTTTTGAATGTGGATTGCTGGTTTATGTTACTAAGAGTGATGGCTCTCGTATACAAACTAGAACGGAGCCCT
TAGTATTAACGCAACACAATTATAATAATATTACTTTAGATAAGTGTGTTAACTATAATATATATGGCAGAGTTGG
CCAAGGTTTTATTACTAATGTGACTGATTCTGCTGCTAATTTTAGTTATTTAGCAGATGGTGGGTTAGCTATTTT
AGACACTTCGGGTGCCATAGATGTTTTTGTTGTACAGGGCAGCTATGGTCCTAATTATTACAAGGTCAATCCT
TGTGAAGATGTTAACCAACAGTTTGTAGTGTCTGGTGGCAATATAGTTGGCATTCTTACTTCTAGAAATGAAA
CAGGTTCTGAACAGGTTGAGAACCAGTTTTATGTTAAGTTAACCAATAGCTCACATCGTCGTAGGCGT
>CK_CH_GX_GL_LY-ZXMB_20220321.seq
ATGTTGGGGAAGTCACTGTTTTTAGTGACCATTTTGTGTGCACTATGTAGTGCAAATTTGTTTGATTTGGCCA
ATAATTATGTGTACTACTACCAAAGTGCCTTTAGGCCTCCAAATGGATGGCATTTGCAAGGGGGTGCTTATGC
AGTAGTGAATTCTACTAATTATACTAGTAATGCCGGTTCTGCAAGTGAGTGCACTGTTGGTATTATTAAGGACG
TCTATAATCAAAGTGCGGCTTCCATAGCTATGACAGCACCTTCTCAGGGTATGGCTTGGTCTAAGTCACAATT
TTGTAGTGCACACTGTAACTTTTCTGAAATTACAGTTTTCGTCACACATTGTTATAGTAGTGGTACAGGGTCTT
GCCCTATAACAGGCATGATTGCACGTGATCATATTCGTATTTCTGCAATGAAAAATGGTTCTTTATTTTATAACT
TAACAGTTAGCGTATCTAAATACTCTAGGTTTAAGTCTTTTCAATGTGTTAACAACCTCACATCTGTTTATCTAA
ATGGTGATCTTGTTTTTACTTCTAACAAAACTACTGATGTTACGTCAGCAGGTGTGTATTTTAAAGCAGGTGG
ACCTGTAAATTATAGTGTTATGAAAGAATTTAAGGTTCTTGCTTATTTTGTTAATGGTACAGCACAAGATGTAA
TTTTGTGTGACAAGTCCCCCAAGGGTTTGCTAGCTTGTCAATATAACACTGGCAATTTTTCAGATGGCTTTTAT
CCTTTTACTAATACTACTTTAGTTAGGGAAAAGTTCATTGTATATCGTGAAAGTAGTGTTAATACTACTCTGGC
GTTAACTAATTTCACTTTTACTAATGTAAGTAATGCACAGCCTAATAGTGGTGGTGTTAATACTTTTCATTTATAT
CAAACACAAACAGCTCAGAGTGGTTATTATAATTTTAATTTGTCATTTCTGAGTCAGTTTGTGTATAAGGCAA
GTGATTTTATGTATGGGTCTTATTATCCTAGGTGTTCTTTTAGACCAGAAACCATTAATAATGGTTTGTGGTTTA
ATTCCTTGTCAGTTTCTCTTACTTATGGACCCCTACAGGGAGGGTGTAAGCAATCTGTTTTTAGTGGTAAGGC
AACGTGTTGTTATGCCTACTCTTATAATGGCCCTAGAGCATGCAAAGGTGTTTATTCAGGCGAATTAAGCAAG
ACTTTTGAATGTGGATTGCTGGTTTATGTTACTAAGAGTGATGGCTCTCGTATACAAACTAGAACGGAGCCCT
TAGTATTAACGCAACACAATTATAATAATATTACTTTAGATAAGTGTGTTAACTATAATATATATGGCAGAGTTGG
CCAAGGTTTTATTACTAATGTGACTGATTCTGCTGCTAATTTTAGTTATTTAGCAGATGGTGGGTTAGCTATTTT
AGACACTTCGGGTGCCATAGATGTTTTTGTTGTACAGGGCAGCTATGGTCCTAATTATTACAAGGTCAATCCT
TGTGAAGATGTTAACCAACAGTTTGTAGTGTCTGGTGGCAATATAGTTGGCATTCTTACTTCTAGAAATGAAA
CAGGTTCTGAACAGGTTGAGAACCAGTTTTATGTTAAGTTAACCAATAGCTCACATCGTCGTAGGCGT
>CK_CH_AH_FY_LH-LSL_20220316.seq
ATGTTGGGGAAGTCACTGTTTTTAGTGACCATTTTGTGTGCACTATGTAGTGCAAATTTGTTTGATCTTGCCA
ATAATTATGTGTACTACTACCAAAGTGCCTTTAGGCCTCCAAATGGATGGCATTTGCAAGGGGGTGCTTATGC
AGTAGTGAATTCTACTAATTATACTAATAATGCCGGTTCTGCAAGTGAGTGCACTGTTGGTATTATTAAGGATG
TCTATAATCAAAGTGTGGCTTCCATAGCTATGACAGCACCTTCTCAGGGTATGGCTTGGTCTAAGTCACAATTT
TGTAGTGCACACTGTAACTTTTCTGAAATTACAGTTTTCGTCACACATTGTTATAGTAGTGGTATAGGGTCTTG
CCCTATAACAGGCATGATTGCACGTGATCATATTCGTATTTCTGCAATGAAAAATGGTTCTTTATTTTATAACTT
AACAGTTAGCGTATCTAAATACTCTAGGTTTAAGTCTTTTCAATGTGTTAACAACCTCACATCTGTTTATCTAAA
TGGTGATCTTGTTTTTACTTCCAACAAAACTACTGATGTTACGTCAGCAGGTGTGTATTTTAAAGCAGGTGGA
CCTGTAAATTATAGTGTTATGAAAGAATTTAAGGTTCTTGCTTATTTTGTTAATGGTACAGCACAAGATGTAAT
TTTGTGTGACAAGTCCCCCAAGGGTTTGCTAGCTTGTCAATATAACACTGGCAATTTTTCAGATGGCTTTTAT
CCTTTTACTAATACTACTTTAGTTAGGGAAAAGTTCATTGTATATCGTGAAAGTAGTGTTAATACTACTCTGGC
GTTAACTAATTTCACTTTTACTAATGTAAGTAATGCACAGCCTAATAGTGGTGGTGTTAATACTTTTCATTTATAT
CAAACACAAACAGCTCAGAGTGGTTATTATAATTTTAATTTTTCATTTCTGAGTCAGTTTGTGTATAAGGCAAG
TGATTTTATGTATGGGTCTTATTATCCTAGGTGTTCTTTTAGACCAGAAACCATTAATAATGGTTTGTGGTTTAA

TTCCTTGTCAGTTTCTCTTACTTATGGACCCCTACAGGGAGGGTGTAAGCAATCTGTTTTTAGTGGTAAGGCA
ACGTGTTGTTATGCCTACTCTTATAATGGCCCTAGAGCATGCAAAGGTGTTTATTCAGGCGAATTAAGCAAGA
CTTTTGAATGTGGATTGCTGGTTTATGTTACTAAGAGTGATGGCTCTCGTATACAAACTAGAACGGAGCCCTT
AGTATTAACGCAACACAATTATAATAATATTACTTTAGATAAGTGTGTTAACTATAATATATATGGCAGAGTTGG
CCAAGGTTTTATTACTAATGTGACTGATTCTGCTGCTAATTTTAGTTATTTAGCAGATGGTGGGTTAGCTATTTT
AGACACTTCGGGTGCCATAGATGTTTTTGTTGTACAGGGCAGATATGGTCCTAATTATTACAAGGTCAACCCT
TGTGAAGATGTTAACCAACAGTTTGTAGTGTCTGGTGGCAATATAGTTGGCATTCTTACTTCTAGAAATGAAA
CAGGTTCTGAACAGGTTGAGAACCAGTTTTATGTTAAGTTAACCAATAGCTCACATCGTCGTAGGCGT
>CK_CH_GD_QY_YF-HX_20220106.seq
ATGTTGGGGAAGTCACTGTTTTTAGTGACCATTTTGTGTGCACTATGTAGTGCAAATTTGTTTGATTTGGCCA
ATAATTATGTGTACTACTACCAAAGTGCCTTTAGGCCTCCAAATGGATGGCATTTGCAAGGGGGTGCTTATGC
AGTAGTGAATTCTACTAATTATACTAGTAATGCCGGTTCTGCAAGTGAGTGCACTGTTGGTATTATTAAGGATG
TCTATAATCAAAGTGCGGCTTCCATAGCTATGACAGCACCTTTTCAGGGTATGGCTTGGTCTAAGTCACAATT
TTGTAGTGCACACTGTAACTTTTCTGAAATTACAGTTTTCGTCACACATTGTTATAGTAGTGGTACAGGGTCTT
GCCCTATAACAGGCATGATTGCACGTGATCATATTCGTATTTCTGCAATGAAAAATGGTTCTTTATTTTATAACT
TAACAGTTAGCGTATCTAAATACTCTAGGTTTAAGTCTTTTCAATGTGTTAACAACCTCACATCTGTTTATCTAA
ATGGTGATCTTGTTTTTACTTCCAACAAAACTACTGATGTTACGTCAGCAGGTGTGTATTTTAAAGCAGGTGG
ACCTGTAAATTATAGTGTTATGAAAGAATTTAAGGTTCTTGCTTATTTTGTTAATGGTACAGCACAAGATGTAA
TTTTGTGTGACAAGTCCCCCAAGGGTTTGCTAGCTTGTCAATATAACACTGGCAATTTTTCAGATGGCTTTTAT
CCTTTTACTAATACTACTTTAGTTAGGGAAAAGTTCATTGTATATCGTGAAAGTAGTGTTAATACTACTCTGGC
GTTAACTAATTTCACTTTTACTAATGTAAGTAATGCACAGCCTAATAGTGGTGGTGTTAATACTTTTCATTTATAT
CAAACACAAACAGCTCAGAGTGGTTATTATAATTTTAATTTGTCATTTCTGAGTCAGTTTGTGTATAAGGCAA
GTGATTTTATGTATGGGTCTTATTATCCTAGGTGTTCTTTTAGACCAGAAACCATTAATAATGGTTTGTGGTTTA
ATTCCTTGTCAGTTTCTCTTACTTATGGACCCCTACAGGGAGGGTGTAAGCAATCTGTTTTTAGTGGTAAGGC
AACGTGTTGTTATGCCTACTCTTATAATGGCCCTAGAGCATGCAAAGGTGTTTATTCAGGCGAATTAAGCAAG
ACTTTTGAATGTGGATTGCTGGTTTATGTTACTAAGAGTGATGGCTCTCGTATACAAACTAGAACGGAGCCCT
TAGTATTAACGCAACACAATTATAATAATATTACTTTAGATAAGTGTGTTAACTATAATATATATGGCAGAGTTGG
CCAAGGTTTTATTACTAATGTGACTGATTCTGCTGCTAATTTTAGTTATTTAGCAGATGGTGGGTTAGCTATTTT
AGACACTTCGGGTGCCATAGATGTTTTTGTTGTACAGGGCAGCTATGGTCCTAATTATTACAAGGTCAATCCT
TGTGAAGATGTTAACCAACAGTTTGTAGTGTCTGGTGGCAATATAGTTGGCATTCTTACTTCTAGAAATGAAA
CAGGTTCTGAACAGGTTGAGAACCAGTTTTATGTTAACTTAACCAATAGCTCACATCGTCGTAGGCGT
>CK_CH_GD_QY_YF-2_20230111.seq
ATGTTGGGGAAGTCACTGTTTTTAGTGACCATTTTGTGTGCACTATGTAGTGCAAATTTGTTTGATTTTGCCA
ATAATTATGTGTACTACTACCAAAGTGCCTTAAGGCCTCCAAATGGATGGCATTTGCAAGGGGGTGCTTATGC
AGTAGTGAATTCTACTAATTATACTAGTAATGCCGGTTCTGCAAGTGAGTGCACTGTTGGTATTATTAAGGACG
TCTATAATCAAAGTGCGGCTTCCATAGCTATGACAGCACCCTTTCAGGGTATGGCTTGGTCTAAGTCACAATT
TTGTAGTGCACACTGTAACTTTTCTGAAATTACAGTTTTCGTCACACATTGTTATAGTAGTGGTACAGGGTCTT
GCCCTATAACAGGCATGATTGCACGTGATCATATTCGTATTTCTGCAATGAAAAATGGTTCTTTATTTTATAACT
TAACAGTTAGCGTATCTAAATACTCTAGGTTTAAGTCTTTTCAATGTGTTAACAACTTCACATCTGTCTATCTAA
ATGGTGATCTTGTTTTTACTTCCAACAAAACTACTGATGTTACGTCAGCAGGTGTGTATTTTAAAGCAGGTGG
ACCTGTAAATTATAGTGTTATGAAAGAATTTAAGGTTCTTGCTTATTTTGTTAATGGTACAGCACAAGATGTAA
TTTTGTGTGACAAGTCCCCCAAGGGTTTGCTAGCTTGTCAATACAACACTGGCAATTTTTCAGATGGCTTTTA
TCCTTTTACTAATACTACTTTAGTTAGGGAAAAGTTCATCGTATATCGTGAAAGTAGTGTTAATACTACTCTGGC
GTTAACTAATTTCACTTTTACTAATGTAAGTAATGCACAGCCTAATAGTGGTGGTGTTAATACTTTTCATTTATAT

CAAACACAAACAGCTCAGAGTGGTTATTATAATTTTAATTTCTCATTTCTGAGTCAGTTTGTGTATAAGGCAAG
TGATTTTATGTATGGGTCTTATCATCCTAGGTGTTCTTTTAGACCAGAAACCATTAATAATGATTTGTGGTTTAA
TTCCTTGTCAGTTTCTCTTACTTATGGACCCCTACAGGGAGGGTGTAAGCAATCTGTTTTTAGTGGTAAGGCA
ACGTGTTGTTATGCCTACTCTTATAATGGCCCTAGAGTATGTAAAGGTGTTTATTCAGGTGAATTAAGCAAGAC
TTTTGAATGTGGATTGCTGGTTTATGTTACTAAGAGTGATGGCTCTCGTATACAGACTAGAACAGAGCCCTTA
GTATTAACGCAACACAATTATAATAATATTACTTTAGATAAGTGTGTTAATTATAATATATATGGCAGAGTGGGC
CAAGGTTTTATTACTAATGTGACTGATTCTGCTGCTAATTTTAGTTATTTAGCAGATGGTGGGTTAGCTATTTTA
GACACTTCGGGTGCCATAGATGTTTTTGTTGCACAGGGCAGCTATGGTCCTAATTATTACAAGGTCAATCCTT
GTGAAGATGTTAACCAACAGTTTGTAGTGTCTGGTGGCAATATAGTTGGCATTCTTACTTCTAGAAATGAAAC
AGGTTCTGAACAGGTTGAGAACCAGTTTTATGTTAAGTTAACCAATAGCTCACATCGTCGTAGGCGC
>CK_CH_GD_QY_YF-1_20230111.seqATGTTGGGGAAGTCACTGTTTTTAGTGACCATTTTGTGTGCACTA
TGTAGTGCAAATTTGTTTGATTTTGCCAATAATTATGTGTACTACTACCAAAGTGCCTTTAGGCCTCCAAATGG
ATGGCATTTGCAAGGGGGTGCTTATGCAGTAGTGAATTCTACTAATTATACTAGTAATGCCGGTTCTGCAAGT
GGGTGCACTGTTGGTATTATTAAGGACGTCTATAATCAAAGTGCGGCTTCCATAGCTATGACAGCACCCTCTC
AGGGTATGGCTTGGTCTAAGTCACAATTTTGTAGTGCACACTGTAACTTTTCTGAAATTACAGTTTTCGTCAC
ACATTGTTATAGTAGTGGTACAGGGTCTTGCCCTATAACAGGCATGATTGCACGTGATCATATTCGTATTTCTG
CAATGAAAAATGGTTCTTTATTTTATAACTTAACAGTTAGCGTATCTAAATACTCTAGGTTTAAGTCTTTTCAAT
GTGTTAACAACTTCACATCTGTCTATCTAAATGGTGATCTTGTTTTTACTTCCAACAAAACTACTGATGTTACGT
CAGCAGGTGTGTATTTTAAAGCAGGTGGACCTGTAAATTATAGTGTTATGAAAGAATTTAAGGTTCTTGCTTA
TTTTGTTAATGGTACAGCACAAGATGTAATTTTGTGTGACAAGTCCCCCAAGGGTTTGCTAGCTTGTCAATAT
AACACTGGCAATTTTTCAGATGGCTTTTATCCTTTTACTAATACTACTTTAGTTAGGGAAAAGTTCATCGTATAT
CGTGAAAGTAGTGTTAATACTACTCTGGCGTTAACTAATTTCACTTTTACTAATGTAAGTAATGCACAGCCTAAT
AGTGGTGGTGTTAATACTTTTCATTTATATCAAACACAAACAGCTCAGAGTGGTTATTATAATTTTAATTTCTCA
TTTCTGAGTCAGTTTGTGTATAAGGCAAGTGATTTTATGTATGGGTCTTATCATCCTAGGTGTTCTTTTAGACC
AGAAACCATTAATAATGATTTGTGGTTTAATTCCTTGTCAGTTTCTCTTACTTATGGACCCCTACAGGGAGGGT
GTAAGCAGTCTGTTTTTAGTGGTAAGGCAACGTGTTGTTATGCCTACTCTTATAATGGCCCTAGAGTATGTAAA
GGTGTTTATTCAGGTGAATTAAGCAAGACTTTTGAATGTGGATTGCTGGTTTATGTTACTAAGAGTGATGGCT
CTCGTATACAGACTAGAACAGAGCCCTTAGTATTAACGCAACACAATTATAATAATATTACTTTAGATAAGTGT
GTTAACTATAATATATATGGCAGAGTGGGCCAAGGTTTTATTACTAATGTGACTGATTCTGCTGCTAATTTTAGT
TATTTAGCAGATGGTGGGTTAGCTATTTTAGACACTTCGGGTGCCATAGATGTTTTTGTTGCACAGGGCAGCT
ATGGTCCTAATTATTACAAGGTTAATCCTTGTGAAGATGTTAACCAACAGTTTGTAGTGTCTGGTGGCAATATA
GTTGGCATTCTTACTTCTAGAAATGAAACAGGTTCTGAACAGGTTGAGAACCAGTTTTATGTTAAGTTAACC
AATAGCTCACATCGTCGTAGGCGC
>CK_CK_XJ_CJ_TK-4_20230625.seq
ATGTTGGGGAAGTCACTGTTTTTAGTGACCATTTTGTGTGCACTATGTAGTGCAAATTTGTTTGATTTGGCCA
ATAATTATGTGTACTACTACCAAAGTGCCTTTAGGCCTCCAAATGGATGGCATTTGCAAGGGGGTGCTTATGC
AGTAGTGAATTCTACTAATTATACTAGTAATGCCGGTTCTGCAAGTGAGTGCACTGTTGGTATTATTAAGGACG
TCTATAATCAAAGTGCGGCTTCCATAGCTATGACAGCACCTTCTCAGGGTATGGCTTGGTCTAAGTCACAATT
TTGTAGTGCACACTGTAACTTTTCTGAAATTACAGTTTTCGTCACACATTGTTATAGTAGTGGTACAGGGTCTT
GCCCTATAACAGGCATGATTGCACGTGATCATATTCGTATTTCTGCAATGAAAAATGGTTCTTTATTTTATAACT
TAACAGTTAGCGTATCTAAATACTCTAGGTTTAAGTCTTTTCAATGTGTTAACAACTTCACATCTGTCTATCTAA
ATGGTGATCTTGTTTTTACTTCCAACAAAACTACTGATGTTACGTCAGCAGGTGTGTATTTTAAAGCAGGTGG
ACCTGTAAATTATAGTGTTATGAAAGAATTTAAGGTTCTTGCTTATTTTGTTAATGGTACAGCACAAGATGTAA
TTTTGTGTGACAAGTCCCCCAAGGGTTTGCTAGCTTGTCAATATAACACTGGCAATTTTTCAGATGGCTTTTAT

CCTTTTACTAATATTACTTTAGTTAGGGAAAAGTTCATCGTATATCGTGAAAGTAGTGTTAATACTACTCTGGCG
TTAACTAATTTCACTTTTACTAATGTAAGTAATGCACAGCCTAATAGTGGTGGTGTTAATACTTTTCATTTATATC
AAACACAAACAGCTCAGAGTGGTTATTATAATTTTAATTTCTCATTTCTGAGTCAGTTTGTGTATAAGGCAAGT
GATTTTATGTATGGGTCTTATCATCCTAGGTGTTCTTTTAGACCAGAAACCATTAATAATGATTTGTGGTTTAAT
TCCTTGTCAGTTTCTCTTACTTATGGACCCCTACAGGGAGGGTGTAAGCAATCTGTTTTTAGTGGTAGGGCAA
CGTGTTGTTATGCCTACTCTTATAATGGCCCTAGAGTATGTAAAGGTGTTTATTCAGGTGAATTAAGCAAGACT
TTTGAATGTGGATTGCTGGTTTATGTTACTAAGAGTGATGGCTCTCGTATACAGACTAGAACAGAGCCCTTAG
TATTAACGCAACACAATTATAATAATATTACTTTAGATAAGTGTGTTAACTATAATATATATGGCAGAGTGGGCC
AAGGTTTTATTACTAATGTGACTGATTCTGCTGCTAATTTTAGTTATTTAGCAGATGGTGGGTTAGCTATTTTA
GACACTTCGGGTGCCATAGATGTCTTTGTTGCACAGGGCAGCTATGGTCCTAATTATTACAAGGTCAATCCTT
GTGAAGATGTTAACCAACAGTTTGTAGTGTCTGGTGGCAATATAGTTGGCATTCTTACTTCTAGAAATGAAAC
AGGTTCTGAACAGGTTGAGAACCAGTTTTATGTTAAGTTAACCAATAGCTCACATCGTCGTAGGCGC
>CK_CH_AH_XC-WXS_20230404.seq
ATGTTGGGGAAGTCACTGTTTTTAGTGACCATTTTGTGTGCACTATGTAGTGCAAATTTGTTTGATCTTGCCA
ATAATTATGTGTACTACTACCAAAGTGCCTTTAGGCCTCCAAATGGATGGCATTTGCAAGGGGGTGCTTATGC
AGTAGTGAATTCTACTAATTATACTAGTAATGCCGGTTCTGCAAGTGAGTGCACTGTTGGTATTATTAAGGATG
TCTATAATCAAAGTGCTGCTTCCATAGCTATGACAGCACCCTCTCAGGGTATGGCTTGGTCTAAGTCACAATTT
TGTAGTGCACACTGTAACTTTTCTGAAATTACAGTTTTCGTCACACATTGTTATAGTAGTGGTACAGGGTCTTG
CCCTATAACAGGCATGATTCCACGTGATCATATTCGTATTTCTGCAATGAAAAATGGTTCTTTATTTTATAACTT
AACAGTTAGCGTATCTAAATACTCTAGGTTTAAGTCTTTTCAATGTGTTAACAACTTCACATCTGTCTATCTAAA
TGGTGATCTTGTTTTTACTTCCAACAAAACTACTGATGTTACGTCAGCAGGTGTGTATTTTAAAGCAGGTGGA
CCTGTAAATTATAGTGTTATGAAAGAATTTAAGGTTCTTGCTTATTTTTGTTAATGGTACAGCACAAGATGTAA
TTTTGTGTGACAAGTCCCCCAAGGGTTTGCTAGCTTGTCAATATAACACTGGCAATTTTTCAGATGGCTTTTAT
CCTTTTACTAATATTACTTTAGTTAGGGAAAAGTTCATCGTATATCGTGAAAGTAGTGTTAATACTACTCTGGCG
TTAACTAATTTCACTTTTACTAATGTAAGTAATGCACAGCCTAATAGTGGTGGTGTTAATACTTTTCATTTATATC
AAACACAAACAGCTCAGAGTGGTTATTATAATTTTAATTTCTCATTTCTGAGTCAGTTTGTGTATAAGGCAAGT
GATTTTATGTATGGGTCTTATCATCCTAGGTGTTCTTTTAGACCAGAAACCATTAATAATGATTTGTGGTTTAAT
TCCTTGTCAGTTTCTCTTACTTATGGACCCCTACAGGGAGGGTGTAAGCAATCTGTTTTTAGTGGTAGGGCAA
CGTGTTGTTATGCCTACTCTTATAATGGCCCTAGAGTATGTAAAGGTGTTTATTCAGGTGAATTAAGCAAGACT
TTTGAATGTGGATTGCTGGTTTATGTTACTAAGAGTGATGGCTCTCGTATACAGACTAGAACAGAGCCCTTAG
TATTAACGCAACACAATTATAATAATATTACTTTAGATAAGTGTGTTAACTATAATATATATGGCAGAGTGGGCC
AAGGTTTTATTACTAATGTGACTGATTCTGCTGCTAATTTTAGTTATTTAGCAGATGGTGGGTTAGCTATTTTA
GACACTTCGGGTGCCATAGATGTTTTTGTTGCACAAGGCAGCTATGGTCCTAATTATTACAAGGTCAATCCTT
GTGAAGATGTTAACCAACAGTTTGTAGTGTCTGGTGGCAATATAGTTGGCATTCTTACTTCTAGAAATGAAAC
AGGTTCTGAACAGGTTGAGAACCAGTTTTATGTTAAGTTAACCAATAGCTCACATCGTCGTAGGCGC
>CK_CH_AH_XC-CSM_20230404.seq
ATGTTGGGGAAGTCACTGTTTTTAGTGACCATTTTGTGTGCACTATGTAGTGCAAATTTGTTTGATCTTGCCA
ATAATTATGTGTACTACTACCAAAGTGCCTTTAGGCCTTCAAATGGATGGCATTTGCAAGGGGGTGCTTATGC
AGTAGTGAATTCTACTAATTATACTAGTAATGCCGGTTCTGCAAGTGAGTGCACTGTTGGTATTATTAAGGATG
TCTATAATCAAAGTGCTGCTTCCATAGCTATGACAGCACCCTCTCAGGGTATGGCTTGGTCTAAGTCACAATTT
TGTAGTGCACACTGTAACTTTTCTGAAATTACAGTTTTCGTCACACATTGTTATAGTAGTGGTACAGGGTCTTG
CCCTATAACAGGCATGATTCCACGTGATCATATTCGTATTTCTGCAATGAAAAATGGTTCTTTATTTTATAACTT
AACAGTTAGCGTATCTAAATACTCTAGGTTTAAGTCTTTTCAATGTGTTAACAACTTCACATCTGTCTATCTAAA
TGGTGATCTTGTTTTTACTTCCAATAAAACTACTGATGTTACGTCAGCAGGTGTGTATTTTAAAGCAGGTGGA

CCTGTAAATTATAGTGTTATGAAAGAATTTAAGGTTCTTGCTTATTTTGTTAATGGTACAGCACAAGATGTAAT
TTTGTGTGACAAGTCCCCCAAGGGTTTGCTAGCTTGTCAATATAACACTGGCAATTTTTCAGATGGCTTTTAT
CCTTTTACTAATATTACTTTAGTTAGGGAAAAGTTCATCGTATATCGTGAAAGTAGTGTTAATACTACTCTGGCG
TTAACTAATTTCACTTTTACTAATGTAAGTAATGCACAGCCTAATAGTGGTGGTGTTAATACTTTTCATTTATATC
AAACACAAACAGCTCAGAGTGGTTATTATAATTTTAATTTCTCATTTCTGAGTCAGTTTGTGTATAAGGCAAGT
GATTTTATGTATGGGTCTTATCATCCTAGGTGTTCTTTTAGACCAGAAACCATTAATAATGATTTGTGGTTTAAT
TCCTTGTCAGTTTCTCTTACTTATGGACCCCTACAGGGAGGGTGTAAGCAATCTGTTTTTAGTGGTAGGGCAA
CGTGTTGTTATGCCTACTCTTATAATGGCCCTAGAGTATGTAAAGGTGTTTATTCAGGTGAATTAAGCAAGACT
TTTGAATGTGGATTGCTGGTTTATGTTACTAAGAGTGATGGCTCTCGTATACAGACTAGAACAGAGCCCTTAG
TATTAACGCAACACAATTATAATAATATTACTTTAGATAAGTGTGTTAACTATAATATATATGGCAGAGTGGGCC
AAGGTTTTATTACTAATGTGACTGATTCTGCTGCTAATTTTAGTTATTTAGCAGATGGTGGGTTAGCTATTTTA
GACACTTCGGGTGCCATAGATGTTTTTGTTGCACAGGGCAGCTATGGTCCTAATTATTACAAGGTCAATCCTT
GTGAAGATGTTAACCAACAGTTTGTAGTGTCTGGTGGCAATATAGTTGGCATTCTTACTTCTAGAAATGAAAC
AGGTTCTGAACAGGTTGAGAACCAGTTTTATGTTAAGTTAACCAATAGCTTACATCGTCGTAGGCGC
>CK_CH_YN_KM_SL-DK_HCM-B_20230223.seq
ATGTTGGGGAAGTCACTGTTTTTAGTGACCATTTTGTGTGCACTATGTAGTGCAAATTTGTTTGATTTTGCCA
ATAATTATGTGTACTACTACCAAAGTGCCCTTAGGCCTCCAAATGGATGGCATTTGCAAGGGGGTGCTTATGC
AGTAGTGAATTCTACTAATTATACTAGTAATGCCGGTTCTGCAAGTGAGTGCACTGTTGGTATTATTAAGGACG
TCTATAATCAAAGTGCGGCTTCCATAGCTATGACAGCACCCTCTCAGGGTATGGCTTGGTCTAAGTCACAATT
TTGTAGTGCACACTGTAACTTTTCTGAAATTACAGTTTTCGTCACACATTGTTATAGTAGTGGTACAGGGTCTT
GCCCTATAACAGGCATGATTGCACGTGATCATATTCGTATTTCTGCAATGAAAAATGGTTCTTTATTTTATAACT
TAACAGTTAGCGTATCTAAATACTCTAGGTTTAAGTCTTTTCAATGTGTTAACAACTTCACATCTGTCTATCTAA
ATGGTGATCTTGTTTTTACTTCCAACAAAACTACTGATGTTACGTCAGCAGGTGTGTATTTTAAAGCAGGTGG
ACCTGTAAATTATAGTGTTATGAAAGAATTTAAGGTTCTTGCTTATTTTGTTAATGGTACAGCACAAGATGTAA
TTTTGTGTGACAAGTCCCCCAAGGGTTTGCTAGCTTGTCAATATAACACTGGCAATTTTTCAGATGGCTTTTAT
CCTTTTACTAATACTACTTTAGTTAGGGAAAAGTTCATCGTATATCGTGAAAGTAGTGTTAATACTACTCTGGC
GTTAACTAATTTCACTTTTACTAATGTAAGTAATGCACAGCCTAATAGTGGTGGTGTTAATACTTTTCATTTATAT
CAAACACAAACAGCTCAGAGTGGTTATTATAATTTTAATTTCTCATTTCTGAGTCAGTTTGTGTATAAGGCAAG
TGATTTTATGTATGGGTCTTATCATCCTAGGTGTTCTTTTAGACCAGAAACCATTAATAATGATTTGTGGTTTAA
TTCCTTGTCAGTTTCTCTTACTTATGGACCCCTACAGGGAGGGTGTAAGCAATCTGTTTTTAGTGGTAAGGCA
ACGTGTTGTTATGCCTACTCTTATAATGGCCCTAGAGTATGTAAAGGTGTTTATTCAGGTGAATTAAGCAAGAC
TTTTGAATGTGGATTGCTGGTTTATGTTACTAAGAGTGATGGCTCTCGTATACAGACTAGAACAGAGCCCTTA
GTATTAACGCAACACAATTATAATAATATTACTTTAGATAAGTGTGTTAACTATAATATATATGGCAGAGTGGGC
CAAGGTTTTATTACTAATGTGACTGATTCTGCTGCTAATTTTAGTTATTTAGCAGATGGTGGGTTAGCTATTTTA
GACACTTCGGGTGCCATAGATGTTTTTGTTGCACAGGGCAGCTATGGTCCTAATTATTACAAGGTTAATCCTT
GTGAAGATGTTAACCAACAGTTTGTAGTGTCTGGTGGCAATATAGTTGGCATTCTTACTTCTAGAAATGAAAC
AGGTTCTGAACAGGTTGAGAACCAGTTTTATGTTAAGTTAACCAATAGCTCACATCGTCGTAGGCGC
>CK_CH_XJ_CJ_TK-BJ-6_20230831.seq
ATGTTGGGGAAGTCACTGTTTTTAGTGACCATTTTGTGTGCACTATGTAGTGCAAATTTGTTTGATTTTGCCA
ATAATTATGTGTACTACTACCAAAGTGCCCTTAGGCCTCCAAATGGATGGCATTTGCAAGGGGGTGCTTATGC
AGTAGTGAATTCTACTAATTATACTAATAATGCCGGTTCTGCAAGTGAGTGCACTGTTGGTATTATTAAGGACG
TCTATAATCAAAGTGCGGCTTCCATAGCTATGACAGCACCCTCTCAGGGTATGGCTTGGTCTAAGTCAGAATT
TTGTAGTGCACACTGTAACTTTTCTGAAATTACAGTTTTCGTCACACATTGTTATAGTAGTGGTATAGGGTCTT
GCCCTATAACAGGCATGATTGCAAGTGGTCATATTCGTATTTCTGCAATGAAAAATGGTTCTTTATTTTATAACT

TAACAGTTAGCGTATCTAAATACTCTAGGTTTAAGTCTTTTCAATGTGTTAACAACTTCACATCTGTCTACTTAA
ATGGTGATCTTGTTTTTACTTCCAACAAAACCACTGATGTTACGTCAGCAGGTGTGTATTTTAAAGCAGGTGG
ACCTGTAAATTATAGTGTTATGAAAGAATTTAAGGTTCTTGCTTATTTTGTTAATGGTACAGCACAAGATGTAA
TTTTGTGTGACAAGTCCCCCAAGGGTTTGCTAGCTTGTCAATATAACACTGGCAATTTTTCAGATGGCTTTTA
CCCTTTTACTAATACTACTTTAGTTAGGGAAAAGTTCATCGTATATCGTGAAAGTAGTGTTAATACTACTCTGGC
GTTAACTAATTTCACTTTTACTAATGTAAGTAATGCACAGCCTAATAGTGGTGGTGTTAATACTTTTCATTTATAT
CAAACACAAACAGCTCAGAGTGGTTATTATAATTTTAATTTCTCATTTCTGAGTCAGTTTGTGTATAAGGCAAG
TGATTTTATGTATGGGTCTTATCATCCTAAGTGTTCTTTTAGACCAGAAACCATTAATAATGATTTGTGGTTTAA
TTCCTTGTCAGTTTCTCTTACTTATGGACCCCTACAGGGAGGGTGTAAGCAATCTGTTTTTAGTGGTAGGGCA
ACGTGTTGTTATGCTTACTCTTATAATGGCCCTAGAGTATGTAAAGGTGTTTATTCAGGTGAATTAAGCAAGAC
TTTTGAATGTGGATTGCTGGTTTATGTTACTAAGAGTGATGGCTCTCGTATACAGACTAGAACAGAGCCCTTA
GTATTAACGCAACACAATTATAATAATATTACTTTAGATAAGTGTGTTAACTATAATATATATGGCAGAGTTGGC
CAAGGTTTTATTACTAATGTGACTGATTCTGCTGCTAATTTTAGTTATTTAGCAGATGGTGGGTTAGCTATTTTA
GACACTTCGGGTGCCATAGATGTTTTTGTTGTACAGGGCAGCTATGGTCCTAATTATTACAAGGTTAATCCTT
GTGAAGATGTTAACCAACAGTTTGTAGTGTCTGGTGGCAATATAGTTGGCATTCTTACTTCTAGAAATGAAAC
AGGTTCTGAACAGGTTGAGAACCAGTTTTATGTTAAGTTAACCAATAGCTCACATCGTCGCAGGCGT
>CK_CK_XJ_CJ_TK-3_20230625.seq
ATGTTGGGGAAGTCACTGTTTTTAGTGACCATTTTGTGTGCACTATGTAGTGCAAATTTGTTTGATTTTGCCA
ATAATTATGTGTACTACTACCAAAGTGCCTTTAGGCCTCCAAATGGATGGCATTTGCAAGGGGGTGCTTATGC
AGTAGTGAATTCTACTAATTATACTAGTAATGCCGGTTCTGCAAGTGAGTGCACTGTTGGTATTATTAAGGACG
TCTATAATCAAAGTGCGGCTTCCATAGCTATGACAGCACCCTCTCAGGGTATGGCTTGGTCTAAGTCACAATT
TTGTAGTGCACACTGTAACTTTTCTGAAATTACAGTTTTCGTCACACATTGTTATAGTAGTGGTACAGGGTCTT
GCCCTATAACAGGCATGATTGCACGTGATCATATTCGTATTTCTGCAATGAAAAATGGTTCTTTATTTTATAACT
TAACAGTTAGCGTATCTAAATACTCTAGGTTTAAGTCTTTTCAATGTGTTAACAACTTCACATCTGTCTACTTAA
ATGGTGATCTTGTTTTTACTTCCAACAAAACTACTGATGTTACGTCAGCAGGTGTGTATTTTAAAGCAGGTGG
ACCTGTAAATTATAGTGTTATGAAAGAATTTAAGGTTCTTGCTTATTTTGTTAATGGTACAGCACAAGATGTAA
TTTTGTGTGACAAGTCCCCCAAGGGTTTGCTAGCTTGTCAATATAACACTGGCAATTTTTCAGATGGCTTTTA
CCCTTTTACTAATACTACTTTAGTTAGGGAAAAGTTCATCGTATATCGTGAAAGTAGTGTTAATACTACTCTGGC
GTTAACTAATTTCACTTTTACTAATGTAAGTAATGCACAGCCTAATAGTGGTGGTGTTAATACTTTTCATTTATAT
CAAACACAAACAGCTCAGAGTGGTTATTATAATTTTAATTTCTCATTTCTGAGTCAGTTTGTGTATAAGGCAAG
TGATTTTATGTATGGGTCTTATCATCCTAGGTGTTCTTTTAGACCAGAAACCATTAATAATGATTTGTGGTTTAA
TTCCTTGTCAGTTTCTCTTACTTATGGACCCCTACAGGGAGGGTGTAAGCAATCTGTTTTTAGTGGTAAGGCA
ACGTGTTGTTATGCTTACTCTTATAATGGCCCTAGAGTATGTAAGGGTGTTTATTCAGGTGAATTAAGCAAGAC
TTTTGAATGTGGATTGCTGGTTTATGTTACTAAGAGTGATGGCTCTCGTATACAGACTAGAACAGAGCCCTTA
GTATTAACGCAACACAATTATAATAATATTACTTTAGATAAGTGTGTTAACTATAATATATATGGCAGAGTTGGC
CAAGGTTTTATTACTAATGTGACTGATTCTGCTGCTAATTTTAGTTATTTAGCAGATGGTGGGTTAGCTATTTTA
GACACTTCGGGTGCCATAGATGTTTTTGTTGTACAGGGCAGCTATGGTCCTAATTATTACAAGGTTAATCCTT
GTGAAGATGTTAACCAACAGTTTGTAGTGTCTGGTGGCAATATAGTTGGCATTCTTACTTCTAGAAATGAAAC
AGGTTCTGAACAGGTTGAGAACCAGTTTTATGTTAAGTTAACCAATAGCTCACATCGTCGTAGGCGC
>CK_CH_XJ_CJ_TK-2_20231206.seq
ATGTTGGGGAAGTCACTGTTTTTAGTGACCATTTTGTGTGCACTATGTAGTGCAAATTTGTTTGATTTTGCCA
ATAATTATGTGTACTACTACCAAAGTGCCTTTAGGCCTCCAAATGGATGGCATTTGCAAGGGGGTGCTTATGC
AGTAGTGAATTCTACTAATTATACTAGTAATGCCGGTTCTGCAAGTGGGTGCACTGTTGGTATTATTAAGGAC
GTCTATAATCAAAGTGCGGCTTCCATAGCTATGACAGCACCCTCTCAGGGTATGGCTTGGTCTAAGTCACAAT

TTTGTAGTGCACACTGTAACTTTTCTGAAATTACAGTTTTCGTCACACATTGTTATAGTAGTGATACAGGGTCT
TGCCCTATAACAGGCATGATTGCACGTGATCATATTCGTATTTCTGCAATGAAAAATGGTTCTTTATTTTATAAC
TTAACAGTTAGCGTATCTAAATACTCTAGGTTTAAGTCTTTTCAATGTGTTAACAACTTCACATCTGTCTACTTA
AATGGTGATCTTGTTTTTACTTCCAACAAAACTACTGATGTTACGTCAGCAGGTGTGTATTTTAAAGCAGGTG
GACCTGTAAATTATAGTGTTATGAAAGAATTTAAGGTTCTTGCTTATTTTGTTAATGGTACAGCACAAGATGTA
ATTTTGTGTGACAAGTCCCCCAAGGGTTTGCTAGCTTGTCAATATAACACTGGCAATTTTTCAGATGGCTTTT
ACCCTTTTACTAATACTACTTTAGTTAGGGAAAAGTTCATCGTATATCGTGAAAGTAGTGTTAATACTACTCTG
GCGTTAACTAATTTCACTTTTACTAATGTAAGTAATGCACAGCCTAATAGTGGTGGTGTTAATACTTTTCATTTA
TATCAAACACAAACAGCTCAGAGTGGTTATTGTAATTTTAATTTCTCATTTCTGAGTCAGTTTGTGTATAAGGC
AAGTGATTTTATGTATGGGTCTTATCATCCTAGGTGTTCTTTTAGACCAGAAACCATTAATAATGATTTGTGGTT
TAATTCCTTGTCAGTTTCTCTTACTTATGGACCCCTACAGGGAGGGTGTAAGCAATCTGTTTTTAGTGGTAAG
GCAACGTGTTGTTATGCTTACTCTTATAATGGCCCTAGAGTATGTAAAGGTGTTTATTCAGGTGAATTAAGCAA
GACTTTTGAATGTGGATTGCTGGTTTATGTTACTAAGAGTGATGGCTCTCGTATACAGACTAGAACAGAGCCC
TTAGTATTAACGCAACACAATTATAATAATATTACTTTAGATAAGTGTGTTAACTATAATATATATGGCAGAGTTG
GCCAAGGTTTTATTACTAATGTGACTGATTCTGCTGCTAATTTTAGTTATTTAGCAGATGGTGGGTTAGCTATT
TTAGACACTTCGGGTGCCATAGATGTTTTTGTTGTACAGGGCAGCTATGGTCCTAATTATTACAAGGTTAACC
CTTGTGAAGATGTTAATCAGCAGTTTATAGTGTCAGGCGGTAAATTAGTAGGCATTCTGACTTCTCGTAATGA
AACTGGTTCTCAGCCTCTTGAAAATCAGTTTTATATTAAGTTAACTAATGGAACCCGCCGTTTTAGACGT
>CK_CH_GD_GZ_AN-XW-2_20231025.seq
ATGTTGGTGAAGTCACTGTTTTTAGCGACTCTTTTGTTTGCACTATCTAGTGCTACTTTGTATGATAATGATACG
TACGTTTACTACTACCAGAGCGCCTTCAGACCGCCTAATGGTTGGCATTTACATGGTGGCGCTTATGCAGTAG
TAAATGTTTCTTCACAAACTAACAATGCAGGTACAGCTTCAGAATGCACTGTTGGTATTATTAGTGGTGATACA
GTTGTTAATGCCTCTTCTATAGCTATGACAGCACCTGTAGGTCAAGGTATGCAGTGGTCTAAGTTACAATTTTG
TACTGCACACTGCAATTTTTCTGATTTTACAGTGTTTGTTACACATTGCTATGCCTCGGGCAGCGGTAAATGTC
CTTTAACGGGCCTTATTTCACAAGGTCATATTCGTATTTCTGCTATGCGGAATCATACTTTATTCTATAATTTAAC
AGTTAGTGTATCTAATTACCCTACTTTTAAATCTTTGCAATGCGTTGATAATTTCACATCTGTTTACTTAAATGGT
GACCTTGTCTTCACTTCTAATCAGACTACAGACGTTATAAGTGCAGGTGTGTACTTTAAATCAGGTGGGCCTA
TAACCTATAAAGTTATGAAGGAATTTAAGGTTTTGGCTTATTTTGTTAATGGTACTGCACAAGATGTTATTTTG
TGTGATGACACACCTAGAGGTTTGCTAGCATGTCAATATAATACTGGCAATTTCTCAGATGGTTTTTATCCTTTT
ACTAATAGTAGCTTAGTTAAGCAAAGGTTTGTTGTTTATCGTGAGAATAGTGTTAATACTACTCTTACTTTAAC
CAATTACACCTTTCATAATGAGACTAATGCCCAGCCTAATTTAGGTGGTGTCCATACTATCTTAACTTATCAAAC
ACAAACTGCTCAGAGTGGTTATTATAATTTTAATTTATCATTTCTGAGTAGTTTTGTGTATAAAGATTCTGATTAT
ATGTATGGGTCCTATCACCCACGATGTAGTTTTAGACCAGAAACTATTAATAATGGCTTGTGGTTTAATTCACT
GTCAGTCTCATTAGCTTATGGCCCCCTTCAAGGTGGGTGTAAGCAATCAGTTTTTCAAGGCAGAGCTACTTG
TTGTTATGCGTATTCCTATAACGGACCACGTATGTGTAAAGGTGTTTATAGTGGTCAGTTATCACAAGATTTTG
AATGTGGACTGTTGGTTTATGTTACTAAGAGTGATGGCTCTCGTATACAAACAGCCACAAAACCACCGGTCAT
AACTCAACACAATTATAATAATATTACTTTAAATACTTGTGTTGAGTACAATATATATGGCAGAGTTGGCCAAG
GCTTTATTACTAATGTAACTGACTCCGCAGCTAGCTATAATTACTTAGCAGATGCTGGATTGGCAATTTTAGAT
ACTTCAGGTGCCATAGACACTTTCGTTGTACAAGGTGAATATGGTCCCAATTATTATAAGGTTAACCCTTGTGA
AGATGTTAATCAGCAGTTTGTAGTGTCAGGCGGTAAGTTAGTAGGCATTCTGACTTCTCGTAATGAAACTGG
TTCTCAGCCTCTTGAAAATCAGTTTTATATTAAGTTAACTAATGGAAGCCGTCGTTTTAGACGT
>CK_CH_JS_SQ_ZDYX-ZH2chang_20230223.seq
ATGTTGGAGAAGTTACTGTTTTTAGTGACCACTTTGTGTGCACTATGTAGTGCAAATTTGTTTGATGCTGATAA
TAGTTATGTGTACTACTACCAGAGTGGATTTAGACCTCCTTCAGGTTGGCACCTTTATGGTGGTGCGTATGCA

GTAGAACGGTTTTTTAATGAAACCAGCAATGCAGGCTCTGGTGCCTGTACTGCTGGAGCCATTGTACATAGT
TTAAATGTTACTGCAAGTGCAGTTGCGATTACTACACCTGTTAATGGCATGCATTGGTCATCTAGTACAGGAG
TGTGTTCAATACATTGCAATTTTAGTACAATTGTTGTTTTTGTTACACATTGTTTTAAAAATGGACAAGGAATAT
GTCCCTTGACAGGTAAATTAAGGGAGGGTGATATTCGTATTGGTGTTCTAGATAGTAGTGGTAATTCTATTTTT
AACAAAACAGTTACCACTTCTAGTTATAGTAAATTTAAATCATTACATTGCGTTAACAATTTCACTTCTGTATAT
TTAAATGGTGATCTTGTTTACACGTCTAATGAAACTTCAGATATTACTGGTTTTGGTGTACATTTTAAGACAGG
AGGACCTGTTACTTATAAAATTATGAAAGAACATAAGGTTCTAGCATATTTTGAAAATGGTACTGCACATGACA
TTATTTTATGTGATGACAGTCCCCGTGGTAGGTTAGCTTGTCAGTATAATACAGGCAATTTTTCTGACGGTTTG
TACCCTTTTAGCGTAAGCAGTGAAGTTAATGAAACTTTTATAGTTTTTGAAAAGAATACAGAAACTACTATGC
TTACATTAAATAATTTCACTTTTTTTAATCAGAGTGGGGCTCAACCTAATCAGAAGGAACCTTCACCTGGTGT
TTCAAATTTTGTGTATTATCAACAGATTAGTGCTGTTCCTGGTTATAATAATTTTAATTTTTCCTTTTTGAGTTCT
TTTACTTATTTAAGTAGTGATTATACGAGGGGTTCTTTTCACCCAAGTTGTACTTTTAGGCCTGAAGATATTAAT
AAAAATCGCAGGTTTAATCATTTGTCTATATCTTTATCTTATGGTCCTCGTAATGGAGGCTGTAAGCAAGCATG
CTTTAATACTAGGAGTTCATGTTGTTGTTTCATGTACTCTTATAATGGTCAACCTTCTTGTAAAGGTGTGTATAG
TGGTGATTTAAATCAAGATTTTGAGTGCGTATTGCTTGTGTTTATTAATCATAGCCCAGGCAGTCGTATATTTAC
TTCTGAAACAGTACCTACTGTCACTGCTAATTTTGCAAATAATGTGGTTTTAGATAGGTGTGTTGATTATAATAT
CTATGTTAGTTA
>CK_CH_GD_SG_DGZ-YSZ_20230216.seq
ATGTTGGAGAAGTTACTGTTTTTAGTGACCAGCTTGTGTGCACTATGTAGTGCAAATTTGTTTGATGCTGATA
ATAGTTATGTGTACTACTACCAGAGTGGATTTAGACCCCCTTTGGGTTGGCACCTCTATGGTGGTGCGTATGC
AGTAGAACGGTTTTTTAATGAAACCAGCAATGCAGGCTCTGGTGACTGTACTGCTGGAGCCATTGTACATAG
TTTAAATGTTACTGCAAGTGCAGTTGCGATTACTACACCTGTTAATGGCATGCATTGGTCATCTAGTACAGGA
GTGTGTTCAATACATTGCAATTTTAGTACAATTGTTGTTTTTGTTACGCATTGTTTTAAAAATGGACAAGGAAT
ATGTCCCTTGACAGGTAAATTAAAGGAGGGTGACATTCGTATTGGTGTTCTAGATAGTAGTGGTAATTCTATT
TTTAATAAAACAGTTACCACTTCTAGTTATAGTAAATTTAAATCATTACATTGCGTTAACAATTTCACTTCTGTAT
ATTTAAATGGTGATCTTGTTTACACGTCTAGTGAAACTTTAGATATTACAGGTTTTGGTGTACACTTTAAGACA
GGAGGACCTGTTACTTATAAAATTATGAAAGAACATAAGGTTCTAGCATATTTTGAAAATGGTACTGCACACG
ACATTATTTTATGTGATGACAGTCCCCGTGGTAGGTTAGCTTGTCAGTATAATACAGGCAATTTTTCTGACGGT
TTGTACCCTTTTAGCGTAAGCAGTGAAGTTAATGAAACTTTTATAGTTTTTGAAAAGAATACAGAAACTACTA
TGCTTACATTAAATAATTTCACTTTTTTAAATCAGAGTGGGGCTCAACCTAATCAAAAGGAACCTTCACCTGG
TGTTTCAAATTTTGTGTATTATCAACAGATTAGTGCTGTTCCTGGTTATAATAATTTTAATTTTTCCTTTTTGAGG
TCTTTTACTTATTTAAGTAGTGATTATATGAAGGGTTCTTTTCACCCAAGTTGTACTTTTAGGCCTGAAGATATT
AATAAAAATCGCAGGTTTAATCATTTGTCTATATCTTTATCTTATGGTCCTCGTAATGGAGGCTGTAAGCAAGC
ATGCTTTAATACTAGGAGTTCATGTTGTTGTTTCATGTACTCTTATAATGGTCAACCTCTTTGTAAAGGTGTGTA
TAGTGGTGATTTAAATCAAGATTTTGAGTGCGTATTGCTTGTGTTTATTAATCATAGCCCAGGCAGTCGTATAT
TTACTTCAGAAACAGTACCTACTGTCACTGCTAATTTTGCAAATAATGTGGTTTTAGATAGGTGTGTTGATTAT
AATATCTATGTTAGTTATGGCAGGTTTTGA
>CK_CH_GD_HY_LH-ZJT_20231011.seq
ATGTTGGTAACACCTCTTTTACTAGTGACTCTTTTGTGTGCACTATGTAGTGCTGCTTTGTATGACAGTAGTTC
TTACGTGTACTACTACCAAAGTGCCTTCAGACCACCTGATGGTTGGCATTTACATGGGGGTGCGTATGCGGTT
GTTAATATTTCTAGTGAATCTAATAATGCAGGCTCTTCATCTGGGTGTACTGTTGGTATTATTCATGGTGGTCGT
GTTGTTAATGCTTCTTCTATAGCTATGACGGCACCGTCATCAGGTATGGCTTGGTCTAGCAGTCAGTTTTGTAC
TGCATACTGTAACTTTTCAGATACTACAGTGTTTGTTACACATTGTTACAAACATGTTGGGTGTCCTATAACTG
GCATGCTTCAACAGCATTCTATACGTGTTTCTGCTATGAAAAATGGCCAGCTTTTTTATAATTTAACAGTTAGT

GTAGCTAAGTACCCTACTTTTAAATCATTTCAGTGTGTTAATAATTTAACATCCGTATATTTAAATGGTGATCTTG
TTTACACCTCTAATGAGACCACAGATGTTACATCTGCAGGTGTTTATTTTAAAGCTGGTGGACCTATAACTTAT
AAAGTTATGAGACAAGTTAGAGCCCTGGCTTATTTTGTTAATGGTACTGCACAAGATGTTATTTTGTGTGATG
GGTCACCTAGAGGCTTGTTAGCATGCCAGTATAATACTGGCAATTTTTCAGATGGCTTTTATCCTTTTACTAAT
AGTAGTTTAGTTAAGCAGAAGTTTATTGTCTATCGTGAAAATAGTGTTAATACTACTCTTACGTTACACAATTTC
ACTTTTCATAATGAGACTGGCGCCAACCCAAATCCTAGTGGTGTCCAGAATATTCAAACTTACCAAACACAAA
CAGCTCAGAGTGGTTATTATAATTTTAATTTTTCCTTTCTGAGTAGTTTTGTTTATAAGGAGTCTAATTTTATGTA
TGGATCTTATCACCCAAGTTGTAATTTTAGACTAGAAACTATTAATAATGGTTTGTGGTTTAATTCACTTTCAGT
TTCAATTGCTTACGGTCCTCTTCAAGGTGGTTGCAAGCAATCTGTCTTTAGTGGTAGAGCAACCTGTTGTTAT
GCTTACTCATATGGAGGTCCTTTGTTGTGTAAAGGTGTTTATTCAGGTGAGTTAGATCATAATTTTGAATGTGG
ACTGTTAGTTTATGTTACTAAGAGCGGTGGCTCTCGTATACAAACAGCCACTGAACCGCCAGTTATAACTCAA
CACAATTATAATAATATTACTTTAAATACTTGTGTTGATTATAATATATATGGCAGAACTGGCCAAGGTTTTATTA
CTAATGTAACCGACTCAGCTGTTAGTTATAATTATCTAGCAGACGCAGGTTTGGCTATTTTAGATACATCTGGT
TCCATAGACATCTTTGTCGTACAAAGTGAATATGGTCTTAATTATTATAAGGTTAACCCTTGCGAAGATGTCAA
CCAGCAGTTTGTAGTTTCTGGTGGTAAATTAGTAGGTATTCTTACTTCACGTAATGAGACTGGTTCCCAGCTT
CTTGAGAATCAGTTTTACATCAAAATCACTAATGGAACACGTCGTTTTAGACGT
>CK_CH_GX_GL_SYXA-xiaojimian_20231109.seq
ATGTTGGTAACACCTCTTTTACTAGTGACTCTTTTGTGTGCACTATGTAGTGCTGTTTTGTATGACAGTAGTTC
TTACGTGTACTACTACCAAAGTGCCTTCAGACCACCTGATGGTTGGCATTTACATGGGGGTGCGTATGCGGTT
GTTAATATTTCTAGTGAATCTAATAATGCAGGCTCTTCATCTGGGTGTACTGTTGGTATTATTCATGGTGGTCGT
GTTGTTAATGCTTCTTCTATAGCTATGACGGCACCGTCATCAGGTATGGCTTGGTCTAGCAGTCAGTTTTGTAC
TGCATACTGTAACTTTTCAGATACTACAGTGTTTGTTACACATTGTTATAAACATGGTGGGTGTCCTATAACTG
GCATGCTTCAACAGCATTCTATACGTGTTTCTGCTATGAAAAATGGCCAGCTTTTTTATAATTTAACAGTTAGT
GTAGCTAAGTACCCTACTTTTAAATCATTTCAGTGTGTTAATAATTTAACATCCGTATATTTAAATGGTGATCTTG
TTTACACCTCTAATGAGACCACAGATGTTACATCTGCAGGTGTTTATTTTAAAGCTGGTGGACCTATAACTTAT
AAAGTTATGAGAGAAGTTAGAGCCCTGGCTTATTTTGTTAATGGTACTGCACAAGATGTTATTTTGTGTGATG
GGTCACCTAGAGGCTTGTTAGCATGCCAGTATAATACTGGCAATTTTTCAGATGGCTTTTATCCTTTTACTAAT
AGTAGTTTAGTTAAGCAGAAGTTTATTGTCTATCGTGAAAATAGTGTTAATACTACTTTTACGTTACACAATTTC
ACTTTTCATAATGAGACTGGCGCCAACCCAAATCCTAGTGGTGTCCAGAATATTCAAACTTACCAAACACAAA
CAGCTCAGAGTGGTTATTATAATTTTAATTTTTCCTTTCTGAGTAGTTTTGTTTATAAGGAGTCTAATTTTATGTA
TGGATCTTATCACCCAAGTTGTAATTTTAGACTAGAAACTATTAATAATGGTTTGTGGTTTAATTCACTTTCAGT
TTCAATTGCTTACGGTCCTCTTCAAGGTGGTTGCAAGCAATCTGTCTTTAGTGGTAGAGCAACCTGTTGTTAT
GCTTACTCATATGGAGGTCCTTTGCTGTGTAAAGGTGTTTATTCAGGTGAGTTAGATCATAATTTTGAATGTG
GACTGTTAGTTTATGTTACTAAGAGCGGTGGCTCTCGTATACAAACAGCCACTGAACCGCCAGTTATAACTCA
ACACAATTATAATAATATTACTTTAAATACTTGTGTTGATTATAATATATATGGCAGAACTGGCCAAGGTTTTATT
ACTAATGTAACCGACTCAGCTGTTAGTTATAATTATCTAGCAGACGCAGGTTTGGCTATTTTAGATACATCTGG
TTCCATAGACATCTTTGTCGTACAAAGTGAATATGGTCTTAATTATTATAAGGTTAACCCTTGCGAAGATGTCA
ACCAGCAGTTTGTAGTTTCTGGTGGTAAATTAGTAGGTATTCTTACTTCACGTAATGAGACTGGTTCCCAGCT
TCTTGAGAATCAGTTTTACATCAAAATCACTAATGGAACACGTCGTTTTAGACGT
>CK_CH_XJ_CJ_TK-BJ-2_20230831.seq
ATGTTGGTGAAGTCACTGTTTTTAGTGACCATTTTGTCTGCACTATGTAGTGCAGTTTTGTATGATAATAATACT
TATGTGTACTACTACCAAAGTGCCTTTAGACCATCAAATGGATGGCATTTGCAGGGGGGTGCTTATGCAGTAG
TGAATTCTACTATTAAATATAACAATGCAGGCTCCGCTAGCGAGTGTTCTGTAGGTGTTCTTTTTAATTATTCTA
ATGGAAATGATGTTGGTTATAATAATAGTGCTTCTTCCGTAGCCATGACAGCACCGTTGTCTGGTATGTCTTGG

TCTAAACAAGAATTTTGTACTGCCCACTGTAATTTTTCGGATATTACAGTGTTTGTTACACATTGTTATGCACAA
TTTTGTCCTTTAACAGGTCCGTTACAGCAAGGCCGTATTCGTATTTCTGCTATGAGAAATGGTTCTCTATTTTAT
AATTCAACAGTTAGTGTATCCCAATACCCTAAATTTAAATCGCTTCAATGTGTCAACAACTTTACTTCTGTTTAT
TTAAATGGTGACCTTGTTTTTTCTTTTAATAAAAGCATTGATGTTACAGGTGCTGGTGTGTACTTTAAAGCAG
GTGGGCCTATAACCTACAAAATTATGAGACAATTTAAGGTTTTGGCTTATTTTCTAAATGGCACTGTGCAAGAT
ATAATTTTGTGTGATGACACACCGAGAGGCTTGCTTGCATGTCAATATAATACTGGTAATTTTTCAGATGGGTT
TTATCCTTTTACTAATTCTAGTTTAGTTAAGGAAAAGTTTATTGTTTATCGTGAGAATAGTGTTAATACTACTCTT
ACTTTAACTAATTATACTTTTTTTAATGAGACTAATGCCTCTCCTAATCAAGGTGATGTTCATACTATCTTAACTT
ATCAAACACAAACTGCCCAGAGTGGTTATTATAATTTTAATTTATCATTTCTGAGTAGTTTTGAGTATAAAGCTT
CTGATTATATGTATGGGTCTTACCACCCACGATGTAGTTTTAGACCAGAAACTATTAATAATGGCTTGTGGTTT
AATTCACTCTCAGTCTCGTTAGCCTATGGCCCCCTTCAAGGTGGGTGTAAGCAATCAGTCTTTCAAGGCAGA
GCTACTTGTTGTTATGCGTATTCATATAACGGACCACGTATCTGTAAGGGTGTTTACATTGGCGAGTTACAAAA
AAGTTTTGAGTGTGGACTGCTGGTTTATGTTACTAAGCGCGATGGCTCTCGTATACAGACAGCCACCATTCCA
CCAGTTATAACTCAACACAATTATAATAATATTACTTTAAATAAGTGTGTTGAGTACAATATATATGGCAGAGTA
GGCCAAGGTTTTATTACTAATATAACTGACTCCGCAGCTAGCTATAATTACTTAGCAGATGCTGGATTGGCAAT
TTTAGATACTTCAGGTGCCATAGACACTTTCGTTGTACAAGGTGAATATGGTCGCAATTATTATAAGGTTAACC
CTTGTGAGGATGTTAACCAACAGCTTGTAGTGTCTGGTGGTGGTATAGTTGGCATGCTTACTTCTAGAAATGA
AACAGGTTCTGAACTGGTTGAGAACATGTTTTATGTTAGGTTAGCTAATAGCTCGCGTCGCCTCAGGCGT
>CK_CH_SX_TC_ENNM_20230525.seq
ATGTTGGGGAAGTCACTGTTTTTAGTGACCATTTTGTGTGCACTATGTAGTGCAAATTTGTTTGATTTAGCCA
ATAATTATGTATACTACTACCAAAGTGCCTACAGGCCTCCAAATGGATGGCATGTGCATGGGGGTGCTTATGC
AGTAGTGAATTCTACTATTAAATATAACAATGCAGGCTCCGCTAGTGCGTGTTCTGTAGGTGTTCTTTTTAATTA
TTCTAACGGAAATGATGTTGGTTATAATAATAGTGCTTCTTCCGTAGCCATGACAGCACCGTTGTCTGGTATGT
CTTGGTCTAAAAAAGAATTTTGTACTGCCCACTGTAACTTTTCTGATATTACAGTGTTTGTTACACATTGTTATG
CACAATCTTGTCCTTTAACGGGTCCGTTAGGTAAAGGCCATATTCGTATTTCTGCTATGAGAAATGGTTCTCTA
TTTTATAATTTAACAGTTAGTGTATCTCAATACCCTAAATTTAAATCGCTTCAATGTGTTAACAACCTCACTTCTG
TTTATTTAAATGGTGACCTTGTTTTTACTTCTAATGAAAGCATTGATCTTACAGGTGCTGGTGTGTACTTTAAA
GCAGGTGGGCCTATAACCTACAAAATTATGAGAGAATTTAAGGTTTTGGCTTATTTTTTAAATGGCACTGTGC
AAGATGTAATTTTGTGTGATGACACACCGAGAGGCTTGCTTGCATGTCAATATAATACTGGTAATTTTTCAGAT
GGGTTTTATCCTTTTACTAATTCTAGTTTAGTTAAGGAAAAGTTTATTGTTTATCGTCAGAATAGTGTCAATACT
ACTCTTACCTTAACTAACTATACTTTTTTTAATGAGACTAACGCCCCTCCTAATTCAGGTGATGTTTATTCTATTC
CAACTTATCAAACACAAACAGCTCAGAGTGGTTATTATAATTTTAATCTATCATTTCTGAGTGGTTTTGTGTATA
AAGAGTCTAATTACATGTATGGGTCTTATCACCATGCATGCAATTTTAGATTAGAAAATATTAATAATGGCTTGT
GGTTTAATTCACTGTCAGTTACGCTAGCTTATGGACCAATTCAAGGTGGGTGTAAGCAGTCGGTCTTTAGTG
GTAGAGCCACTTGTTGTTATGCTTATTCATATAACGGTCCTCACCTCTGTAAGGGTGTTTACAGCGGCGAGTT
ACAAAGAAGTTTTGAATGTGGATTGTTGGTTTATGTTACTAAGAGTGATGGCTCTCGTATACAAACAGCCACC
GTTCCACCAGTTATAACTCAACACAATTATAATAATATTACTTTAAATAAGTGTGTTGACTATAATATATATGGCA
GAGTAGGCCAAGGTTTTATTACTAATGTGACTGATTCTGTTGCTAATTTTAGTTATTTAGCAGATGGTGGGTTA
GCTATTTTAGATACTTCGGGTGCCATAGATGTCTTTGTTGTACAGGGCAGCTATGGTCTTAATTATTACAAGGT
CAATCCTTGTGAAGATGTTAACCAACAGTTTGTAGTGTCTGGTGGCAATATAGTTGGCGTGCTTACTTCTAGA
AATGAAACAGGTTCTGAACAGGTTGAGAACCAGTTTTATGTTAAGTTAAACAATAGCTCACATCGCCGCAAG
CGT
>CK_CH_GD_QY_TN-LS_20231113.seq
ATGTTGGGGAAGTCACTGTTCTTAGTGATCATTTTGTGTGCACTATGTAGTGCAAATTTGTTTGATTTAGCCAA

TAATTATGTGTACTACTACCAAAGTGCCTTTAGGCCTCCAAATGGATGGCATTTGCATGGGGGTGCTTATGCA
GTAGTGAATTCTACTATTAAATATAACAATGCAGGCTCCGCTAGTGCGTGTTCTGTAGGTGTTCTCTTTAATTAT
TCTAACGGAAATGATGTCGGTTATAATAATAGTGCTTCTTCTGTAGCCATGACAGCACCATTGTCTGGTATGTC
TTGGTCTAAAGAAGAATTTTGTACTGCCCACTGTAACTTTTCGGATATTACAGTGTTTGTTACACATTGTTATG
CACAATCTTGTCCTTTAACGGGTCAGTTAGGTAAGGGCCATATTCGTATTTCTGCTATGAGAAATGGTTCTCTA
TTTTATAATTTAACAGTTGATGTATCCCAATACCCTAAATTTAAATCGCTTCAATGTGTTAACAACCTCACTTCT
GTTTATCTAAATGGTGACCTTGTTTTTACTTCTAATGAAAGCACTAATGTTATAGGTGCTGGTGTGTACTTTAA
AGCAGGTGGGCCTATAACCTACAAAATTATGAGAGAATTTAAGGTTTTGGCTTATTTTTTAAATGGCACTGCG
CAAGATGTAATTTTGTGTGATGACACACCGAGAGGCTTGCTTGCATGTCAATATAATACTGGTAATCTTTCAGA
TGGGTTTTATCCTTTTACTAATTCTAGTTTAGTTAAGGAAAAGTTTATTGTTTATCGTCAGAATAGTGTCAATAC
CACTCTTACCTTAACTAACTATACTTTTTCTAATGAGACTAATGCCCCTCCTAATTCAGGTGATGTTCATTCTATT
CCAACTTATCAAACACAAACAGCTCAGAGTGGTTATTATAATTTTAATTTATCATTTCTGAGTAGTTTTGTGTAT
AAAGAGTCTAATTACATGTACGGGTCTTATCACCGTGCATGCAATTTTAGATTAGAAAATATTAATAATGGCTT
GTGGTTTAATTCACTGTCAGTTACGCTAGCTTATGGACCACTTCAAGGTGGGTGTAAGCAGTCAGTCTTTAGT
GGTAGAGCCACTTGTTGTTATGCTTATTCATATAATGGTCCTCACCTCTGTAAGGGTGTTTACAGCGGCGAGTT
ACAAAAAAGTTTTGAATGTGGATTGTTGGTTTATGTTACTAAGCGTGATGGCTCTCGTATACAAACAGCCACC
GTTCCACCAGTTATAACTCAACACAATTATAATAATATTACTTTAAATAAGTGTGTTGACTATAATATATATGGCA
GAGTAGGCCAAGGTTTTATTACTAATGTGACTGATTCTGCTGCTAATTTTAGTTATTTAGCAGATGGTGGGTTA
GCTATTTTAGATACTTCGGGTGCCATAGATGTTTTTGTTGTACAGGGCAGCTATGGTCTTAATTATTACAAGGT
TAATCCTTGTGAAGATGTTAACCAACAGTTTGTAGTGTCTGGTGGCAACATAGTTGGCATGCTTACTTCTAGA
AATGAAACAGGTTCTGAACAGGTTGAGAACCAGTTTTATGTTAAGTTAACCAATAGCTCACATCGTCGCAAG
CGT
>CK_CH_GX_LZ_LH-WYJ_20230516.seq
ATGTTGGGGAAGTCACTGTTTTTAGTGACCATTTTGTGTGCACTATGTAGTGCAAAATTGTTTGATTCTGCTAA
TAATTATGTGTACTACTACCAAAGTGCCTTTAGGCCTCCAAATGGATGGCATTTGCAAGGGGGTGCTTATGCA
GTAGTGAATTCTACTAATTATACTAATAATGCAGGTTCTGCAAATGAGTGCACTATTGGTGTTATTAAGGACGT
CTATAATCAAAGTGCGGCTGCTATAGCTATGACAGCACCTCTTCAGGGTATGGCTTGGTCTAAGTCACAATTT
TGTAGTGCACACTGTAACTTTTCTGAAATTACAGTTTTTGTCACACATTGTTATAGTAGCGGTAGTTGGTCTTG
TCCTATAACAGGCATGATTCCACAGGGTCATATTCGCATTTCTGCAATGAAAAATGGCTCTTTATTTTATAATTT
AACAGTTAGCGTGTCTAAATACCCTAATTTTAAATCGTTTCAATGTGTTAACAACTTCACTTCTGTTTATTTAAA
TGGTGATCTTGTTTTTACTTCTAACACAACTACTGATGTTAAGTCAGCAGGTGTGTATTTTAAAGCAGGTGGA
CCTGTAAATTATAATATTATGAAAGAATTTAAGGTTCTGGCTTATTTTGTCAATGGTACTGTGCAAGATGTAATT
CTGTGTGATGACACACCGAGAGGCTTGCTTGCATGTCAATATAATAATGGTAATTTTTCAGATGGGTTTTACC
CTTTTACTAATTCTAGTTTAGTTAAGGAAAAGTTTATTGTTTATCGTGAGAATAGTGTTAATACTACTCTTACTT
TAACTAACTATACTTTTTATAATGTGACTAATGCCTCGCCTAATCGAGGTGGTGTTCAGTCTATTCCAACTTATC
AAACACAAACAGCTCAGAGTGGTTATTATAATTTTAATTTATCATTTCTGAGTAGTTTTGTGTATAAAGAGTCT
AATTACATGTATGGGTCTTACCACCCTGCATGTAATTTTAGATTAGAAACTATTAATAATGGCTTGTGGTTTAAT
TCATTGTCAGTTTCGCTTGCTTATGGACCACTTCAAGGTGGGTGTAAGCAGTCGGTTTTTAGTAGTAGAGCC
ACTTGTTGTTATGCTTATTCATATAATGGTCCTCGCGCATGTAAGGGTGTTTACGCAGGCGAGTTACTACAAAA
TTTTGAATGTGGACTGTTGGTTTATGTTACTAAGAGCGATGGCTCTCGTATACAAACAGCCACCGTTCCACCA
GTTGTAACTCAACACAATTATAATAATATTACTTTAAATACTTGTGTTGATTATAATATATATGGCAGAGTTGGTC
GAGGTTTTATTACTAATGTAACTGACTCATCATCTAGTTATAATTATTTAGCAGATGCAGGGTTGGCTATTTTAG
ATACATCAGGTGCCATAGACATCTTTGTTGTACAAGGTGAACATGGTCTTAATTATTACAAGGTTAATCCCTGT
GAAGATGTAAACCAGCAGTTTGTAGTTTCTGGTGGTAAATTAGTAGGTATTCTTACCTCACGTAATGCAACAG

GTTCTCAGCCTCTTGAGAATCAATTCTACATTAAACTCACTAAAGAGACACGTCGTTTTAGACGT
>CK_CH_XJ_CJ_TK-4-20231206.seq
ATGTTGGGGAAGTCACTGTTCTTAGTGACCATTTTGTGTGCACTATGTAGTGCAAATTTGTTTGATTTGGCCA
ATAATTATGTGTACTACTACAAAAGTGCCTTTAGGCCTCCAGATGGATGGCATTTGCAAGGGGGTGCTTATGC
AGTAGTGAATTCTACTAAGTATACTAATAATGCCGGTTCTGCAAGTGAGTGCACTGTTGGTATTATTAAGGAC
GTCTATAATCAAAGTGCGGCTTCCATAGCTATGACAGCACCTTCTCAGGGTATGGCTTGGTCTAAGTCACAAT
TTTGTAGTGCACACTGTAACTTTTCTGAAATTACAGTTTTCGTCACACATTGTTTTAGTAGTGGTATAGGGTCT
TGCCCTATAACAGGCATGATTGCACGTGATCATATTCGTATTTCTGCAATGAAAAATGGTTCTTTATTTTATAAC
TTAACAGTTAGCGTATCTAAATACTCTAGGTTTAAGTCTTTTCAATGTGTTAACAACCTCACATCTGTTTATCTA
AATGGTGATCTTGTTTTTACTTCCAACAAAACTACTGATGTTACGTCAGCAGGTGTGTATTTTAAAGCAGGTG
GACCTGTAAATTATAGTGTTATGAAAGAATTTAAGGTTCTTGCTTATTTTGTTAATGGTACAGCACAAGATGTA
ATTTTGTGTGATAACTCACCTAGAGGTTTGCTTGCATGTCAGTATAACACTGGTAATTTTTCAGATGGATTCTA
CCCTTTTACTAATTCTTCTTTAGTTAAGGATAGGTTTATTGTACATCGAGAAAGTAGCACTAACACTACTTTAG
AGTTAACTAATTTCACTTTTACTAATGTAAGTAATGCTTCTCCTAATTCAGGTGGCGTTGATACTTTCCAATTAT
ATCAAACACATACTGCTCAGGATGGTTATTATAATTTTAATTTATCATTTCTGAGTAGTTTTGTGTATAAACCATC
TGATTTTATTTATGGGTCATACCACCCAAATTGTAATTTTAGACCAGCGAATATTAATAATGGCTTATGGTTTAA
TTCATTATCAGTTTCTCTTACTTATGGACCCCTACAGGGAGGGTGTAAGCAATCTGTTTTTAGTGGTAAGGCA
ACGTGTTGTTATGCCTACTCTTATAATGGCCCTAGAGCATGCAAAGGTGTTTATTCAGGCGAATTAAGCAAGA
CTTTTGAATGTGGATTGCTGGTTTATGTTACTAAGAGTGATGGCTCTCGTATACAAACTAGAACGGAGCCCTT
AGTATTAACGCAACACAATTATAATAATATTACTTTAGATAAGTGTGTTAACTATAATATATATGGCAGAGTTGG
CCAAGGTTTTATTACTAATGTGACTGATTCTGCTGCTAATTTTAGTTATTTAGCAGATGGTGGGTTAGCTATTTT
AGATACTTCGGGTGCCATAGATGTTTTTGTTGTACAGGGCAGCTATGGTCTTAATTATTACAAGGTCAATCCTT
GTGAAGATGTTAACCAACAGTTTGTAGTGTCTGGTGGCAATATAGTTGGCATTCTTACTTCTAGAAATGAAAC
AGGTTCTGAACAGGTTGAGAACCAGTTTTATGTTAAGTTAACCAATAGCTCACATCGTCGTAGGCGT
>CK_CH_GD_QY_TN-1B_20230227.seq
ATGTTGGGGAAGTCACTGTTTTTAGTGACCATTTTGTGTGCACTATGTAGTGCAAATTTGTTTGATTTGGCCA
ATAATTATGTGTACTACTACCAAAGTGCCTATAGACCACCAAATGGATGGCATTTGCAAGGGGGTGCTTATGC
AGTAGTGAATTCCACTAATAAATTTAACAATGCAGGCGCCGCTAGCGAGTGTTCTGTAGGTGTTCTTTTTAATT
ATACTAACGGAAATGACGTTGGTTATAATAATAGTGCTTCTTCCGTAGCCATGACAGCACCGCTTTCTGGTATG
TCTTGGTCTAAAGCACAATTTTGTACTGCCCATTGTAACTTTTCGGATTTTACAGTGTTTGTTACACATTGTTTT
GCAGCTTTTTGTCCTTTAACAGGTACTATAGAGAAAAACCATATCCGTATTTCTGCTATGAGAAATGGTTCTCT
ATTTTATAATTTAACAGTTAGTGTATCCAAATACCCTAAGTTTAAATCGCTTCAATGTGTTAACAATTTCACTTCT
GTTTATTTAAATGGTGACCTTGTTTTTACTTCTAATAAAACCACTGATGTTATAGGTGCTGGTGTGTATTTTAAA
GCAGGTGGGCCTATAACCTACAAAATTATGAAAGAATTTAAGGTTTTGGCTTATTTTGTCAATGGCACTGTGC
AAGATGTAATTCTGTGTGATAACTCACCTAGAGGTTTGCTTGCATGTCAGTATAACACTGGTAATTTTTCAGAT
GGATTCTACCCTTTTACTAATTCTTCTTTAGTTAAGGATAGGTTTATTGTATATCGAGAAAGTAGCACTAACACT
ACTTTAGAGTTAACTAATTTTACTTTTACTAATGTAAGTAATGCTTCTCCTAATTCAGGTGGCGTTGATACTTTC
CAATTATATCAAACACATACTGCTCAGGATGGTTATTATAATTTTAATTTATCATTTCTGAGTAGTTTTGTGTATA
AACCATCTGATTTTATGTATGGGTCATACCACCCAAAGTGTAATTTTAGACCAGAGAATATTAATAATGGCTTAT
GGTTTAATTCATTATCTGTGTCACTTACTTACGGACCCATTCAAGGTGGTTGTAAGCAATCTGTTTTTAGTAAT
AAAGCAACTTGTTGCTATGCTTATTCTTACCGAGGTCCTACTAGATGCACTGGTGTTTATAGAGGGGAGCTAA
TGCAATACTTTGAATGTGGACTTCTAGTTTATGTAACTAAGAGTGATGGCTCTCGTATACAAACTAGAAGTGA
ACCACTGGTGTTAACTCAATATAATTATAACAACATTACTTTAAATAAGTGTGTTGATTATAATATATATGGTAGA
GTTGGTCAAGGTTTTATTACTAATGTAACTGAAGCAACTGCTAATTATAGTTATCTAGCAGATGGTGGTTTAGC

TATTTTAGATACTTCAGGAGCCATAGACATATTTGTTGTTCGAGGTGCATATGGTCTTAATTATTACAAGGTCAA
TCCTTGTGAAGATGTTAATCAACAGTTTGTAGTGTCTGGTGGCAACATAGTTGGCATGCTTACCTCTAGAAAT
GAAACAGGTTCTGAACAGGTTGAGAACCAGTTTTATGTTAAGTTAACCAATAGCTCACATCGTCGCAAGCGT
>CK_CH__GS_SYNM-MB_XY-1-2#_7d_20230821.seq
ATGTTGGGCAAACCGCTTTTACTAGTGACTCTTTGGTATGCACTATGTAGTGCTTTGCTTTATGATAAAAATAC
TTACGTTTACTACTACCAAAGTGCCTTTAGGCCTGGTCAAGGTTGGCATCTACATGGGGGTGCTTATGCAGTA
GATAAGGTTTTTAATGGAACCAACAATGCAGTCAGTGTATCTGATTGCACTGCTGGTACTTTTTATGAAAGCT
ATAATATTTCTGCTGCTTCTGTAGCCATGACAGTACCACCTGCTGGTATGTCTTGGTCAGTTGCACAGTTTTGT
ACAGCTCATTGTAACTTCTCAGACTTTACAGTGTTTGTTACGCATTGTTTTAAAAGTCAACAAGGTAGTTGTC
CATTGACAGGTATGATTCCTCAGAATCATATTCGTATTTCTGCTATGAGATCTGGATTTTTGTTTTATAATTTAAC
AGTTAGCGTATCTAAATACCCTAAATTTAAATCGCTTCAATGTGTTGGCAATTCTACATCTGTCTATTTAAATGG
TGATCTTGTTTTCACTTCTAATGAAACAACTCACGTTACGGGTGCAGGCGTTTATTTTAAAAGTGGTGGGCCT
GTAACTTATAAAGTTATGAAAGAAGTTAAAGCCCTAGCCTACTTTATTAATGGTACCGCACAAGAGGTTATTTT
ATGTGATAACTCACCTAGAGGTTTGCTTGCATGTCAGTATAACACTGGTAATTTTTCAGATGGATTCTACCCTT
TTACTAATTCTTCTTTAGTTAAGGATAGGTTTATTGTATATCGAGAAAGTAGCACTAACACTACTTTAGAGTTAA
CTAATTTCACTTTTACTAATGTAAGTAATGCTTCTCCTAATTCAGGTGGCGTTGATACTTTCCAATTATATCAAA
CACATACTGCTCAGGATGGTTATTATAATTTTAATTTATCATTTCTGAGTAGTTTTGTGTATAAACCATCTGATTT
TATGTATGGGTCATACCACCCAAATTGTAATTTTAGACCAGAGAATATTAATAATGGCTTATGGTTTAATTCATT
ATCTGTGTCACTTACTTACGGACCCATTCAAGGTGGTTGTAAGCAATCTGTTTTTAGTAATAAAGCAACTTGTT
GCTATGCTTATTCTTACCGAGGTCCTACTAGATGTAAGGGTGTTTATAGAGGGGAGCTAACGCAATACTTTGA
ATGTGGACTTCTAGTTTATGTAACTAAGAGTGATGGCTCTCGTATACAAACTAGAAGTGAACCACTGGTGTTA
ACTCAATATAATTATAACAACATTACTTTAAATAAGTGTGTTGAGTATAATATATATGGTAGAGTTGGTCAAGGT
TTTATTACTAATGTAACTGAAGCAACTGCTAATTATAGTTATCTAGCAGATGGTGGTTTAGCTATTTTAGATACT
TCAGGAGCCATAGACATATTTGTTGTTCGAGGTGCATATGGTCTTAATTATTATAAGGTTAATCCCTGTGAAGA
TGTTAACCAACAGTTTGTAGTGTCTGGTGGCAATTTAGTTGGCATTCTTACATCTCATAATGAAACAGATTCTG
AATTTATTGAGAACCAGTTTTACATCAAACTCACTAACGGAACACGTCGCTCTAGACGT
>CK_CH_GD_SH_HF-qiangshizi_20231120.seq
ATGTTGGGCAAACCGCTTTTACTAGTGACTCTTTGGTATGCACTATGTAGTGCTTTGCTTTATGATAAAAATAC
TTACGTTTACTACTACCAAAGTGCCTTTAGGCCTGGTCAAGGTTGGCATCTACATGGGGGTGCTTATGCAGTA
GATAAGGTTTTTAATGGAACCAACAATGCAGTCAGTGTATCTGATTGCACTGCTGGTACTTTTTATGAAAGCT
ATAATATTTCTGCTGCTTCTGTAGCCATGACAGTACCACCTGCTGGTATGTCTTGGTCAGTTTCACAGTTTTGT
ACAGCTCATTGTAACTTCTCAGACTTTACAGTGTTTGTTACGCATTGTTTTAAAAGTCAACAAGGTAGTTGTC
CATTGACAGGTATGATTCCTCAGAATCATATTCGTATTTCTGCTATGAGATCTGGATTTTTGTTTTATAATTTAAC
AGTTAGCGTATCTAAATACCCTAAATTTAAATCGCTTCAATGTGTTGGCAATTCTACATCTGTCTATTTAAATGG
TGATCTTGTTTTCACTTCTAATGAAACAACTCACGTTACGGGTGCAGGCGTTTATTTTAAAAGTGGTGGGCCT
GTAACTTATAAAGTTATGAAAGAAGTTAAAGCCCTAGCCTACTTTATTAATGGTACCGCACAAGAGGTTATTTT
ATGTGATAACTCACCTAGAGGTTTGCTTGCATGTCAGTATAACACTGGTAATTTTTCAGATGGATTCTACCCTT
TTACTAATTCTTCTTTAGTTAAGGATAGGTTTATTGTATATCGAGAAAGTAGCACTAACACTACTTTAGAGTTAA
CTAATTTCACTTTTACTAATGTAAGTAATGCTTCTCCTAATTCAGGTGGCGTTGATACTTTCCAATTATATCAAA
CACATACTGCTCAGGATGGTTATTATAATTTTAATTTATCATTTCTGAGTAGTTTTGTGTATAAACCATCTGATTT
TATGTATGGGTCATACCACCCAAATTGTAATTTTAGACCAGAGAATATTAATAATGGCTTATGGTTTAATTCATT
ATCTGTGTCACTTACTTACGGACCCATTCAAGGTGGTTGTAAGCAATCTGTTTTTAGTAATAAAGCAACTTGTT
GCTATGCTTATTCTTACCGAGGTCCTACTAGATGTAAGGGTGTTTATAGAGGGGAGCTAACGCAATACTTTGA
ATGTGGACTTCTAGTTTATGTAACTAAGAGTGATGGCTCTCGTATACAAACTAGAAGTGAACCACTGGTGTTA

ACTCAATATAATTATAACAACATTACTTTAAATAAGTGTGTTGAGTATAATATATATGGTAGAGTTGGTCAAGGT
TTTATTACTAATGTAACTGAAGCAACTGCTAATTATAGTTATCTAGCAGATGGTGGTTTAGCTATTTTAGATACT
TCAGGAGCCATAGACATATTTGTTGTTCGAGGTGCATATGGTCTTAATTATTATAAGGTTAATCCCTGTGAAGA
TGTTAACCAACAGTTTGTAGTGTCTGGTGGCAATTTAGTTGGCATTCTTACATCTCATAATGAAACAGATTCTG
AATTTATTGAGAACCAGTTTTACATCAAACTCACTAACGGAACACGTCGCTCTAGACGT
>CK_CH_GD_JM_KP-STZJC-gangshizi_20231101.seq
ATGTTGGGCAAACCGCTTTTACTAGTGACTCTTTGGTATGCACTATGTAGTGCTTTGCTTTATGATAAAAATAC
TTACGTTTACTACTACCAAAGTGCCTTTAGGCCTGGTCAAGGTTGGCATCTACATGGGGGTGCTTATGCAGTA
GATAAGGTTTTTAATGGAACCAACAATGCAGTCAGTGTATCTGGTTGCACTGCTGGTACTTTTTATGAAAGCT
ATAATATTTCTGCTGCTTCTGTAGCCATGACAGTACCACCTGCTGGTATGTCTTGGTCAGTTTCACAGTTTTGT
ACAGCTCATTGTAACTTCTCAGACTTTACAGTGTTTGTTACGCATTGTTTTAAAAGTCAACAAGGTAGTTGTC
CATTGACAGGTATGATTCCTCAGAATCATATTCGTATTTCTGCTATGAGATCTGGATTTTTGTTTTATAATTTAAC
AGTTAGCGTATCTAAATACCCTAAATTTAAATCGCTTCAATGTGTTGGCAATTCTACATCTGTCTATTTAAATGG
TGATCTTGTTTTCACTTCTAATGAAACAACTCACGTTACGGGTGCAGGCGTTTATTTTAAAAGTGGTGGGCCT
GTAACTTATAAAGTTATGAAAGAAGTTAAAGCCCTAGCCTACTTTATTAATGGTACCGTACAAGAGGTTATTTT
ATGTGATAACTCACCTAGAGGTTTGCTTGCATGTCAGTATAACACTGGTAATTTTTCAGATGGATTCTACCCTT
TTACTAATTCTTCTTTAGTTAAGGATAGGTTTATTGTATATCGAGAAAGTAGCACTAACACTACTTTAGAGTTAA
CTAATTTCACTTTTACTAATGTAAGTAATGCTTCTCCTAATTCAGGTGGCGTTGATACTTTCCAATTATATCAAA
CACATACTGCTCAGGATGGTTATTATAATTTTAATTTATCATTTCTGAGTAGTTTTGTGTATAAACCATCTGATTT
TATGTATGGGTCATACCACCCAAATTGTAATTTTAGACCAGAGAATATTAATAATGGCTTATGGTTTAATTCATT
ATCTGTGTCACTTACTTACGGACCCATTCAAGGTGGTTGTAAGCAATCTGTTTTTAGTAATAAAGCAACTTGTT
GCTATGCTTATTCTTACCGAGGTCCTACTAGATGTAAGGGTGTTTATAGAGGGGAGCTAACGCAATACTTTGA
ATGTGGACTTCTAGTTTATGTAACTAAGAGTGATGGCTCTCGTATACAAACTAGAAGTGAACCACTGGTGTTA
ACTCAATATAATTATAACAACATTACTTTAAATAAGTGTGTTGAGTATAATATATATGGTAGAGTTGGTCAAGGT
TTTATTACTAATGTAACTGAAGCAACTGCTAATTATAGTTATCTAGCAGATGGTGGTTTAGCTATTTTAGATACT
TCAGGAGCCATAGACATATTTGTTGTTCGAGGTGCATATGGTCTTAATTATTATAAGGTTAATCCCTGTGAAGA
TGTTAACCAACAGTTTGTAGTGTCTGGTGGCAATTTAGTTGGCATTCTTACATCTCATAATGAAACAGATTCTG
AATTTATTGAGAACCGGTTTTACATCAAACTCACTAACGGAACACGTCGCTCTAGACGT
>CK_CH_XJ_CJ_TK-1_20230111.seq
ATGTTGGTGAAGTCACTGTTTTTAGTGACTCTTTTGTTTGCACTATCTAGAGCTACTTTGTATGATAATGATAC
GTACGTTTACTACTACCAGAGCTCCTTCAGACCGTCTGGTGGTTGGCATTTACATGGTGGCGCTTATGCAGTA
GTAAATGTTTCTTCACAAACTAACAATGCAGGTACAGCTCCAGAATGCACTGTTGGTATTATTAGTGGTGATA
CAGTTGTTAATGCCTCTTCTATAGCTATGACAGCACCTGTAGGTCAAGGTATGCAGTGGTCCAAGTTACAATT
TTGTACTGCACACTGCAATTTTTCTGATTTTACAGTGTTTGTTACACATTGCTATGCCTCGGGCAGCGGTAAAT
GTCCTTTAACGGGCCTTATTCCACAAGGTCATATTCGTATTTCTGCTATGCGGAATCATACTTTATTCTATAATTT
AACAGTTAGTGTATCTAAGTACCCTACTTTTAAATCTCTGCAATGCGTTGCTAATTTCACATCTGTTTACTTAAA
TGGTAACCTTGTCTTCACTTCTAATCAGACTACAGACGTTATAAGTGCAGGTGTGTACTTTAAAGCAGGTGGG
CCTATAACCTATAAAGTTATGAAGGAATTTAAGGTTTTGGCTTACTTTGTTAATGGTACAGCACAAGACGTAAT
TTTGTGTGACAATTCCCCTAAGGGTTTGCTAGCCTGTCAATATAGTACTGGCAATTTTTCAGATGGCTTCTATC
CTTTTACTAATGGCACTTTGGTTAGGGACAAGTTCATTGTCTATCGTGAAAGTAGTGTTAATACTACTTTGACG
TTAACTAATTTCACTTTTACTAATGTAAGTAATGCACAGCCTAATAGTGGTGGTGTTAATACTTTTCATCTATATC
AAACACAAACAGCTCAGAGTGGTTATTATAATTTTAATTTGTCATTTCTGAGTCAGTTTGTGTATAAGGCAAGT
GATTTTATGTATGGGTCTTACCATCCTAGGTGTTCTTTTAGACCAGAAACCATTAATAGTGGTTTATGGTTTAAT
TCCTTGTCAGTTTCTCTTACTTATGGACCCCTACAGGGAGGGTGTAAGCAATCTGTTTTTAGTGGTAAGGCAA

CTTGTTGTTATGCCTACTCTTATAATGGCCCTAGGGCATGTAAAGGTGTTTATTCAGGTGAATTAAGCAAGACT
TTTGAATGTGGATTGCTGGTTTATGTTACTAAGAGTGATGGCTCTCGTATACAAACTAGAACAGAACCCTTAG
TATTAACGCAACACAATTATAATAATGTTACTTTAGATAAGTGTGTTGACTATAATATATATGGCAGAGTAGGCC
AAGGTTTTATTACTAATGTGACTGATTCTGCTGCTAATTTTAATTATTTAGCAGACGGTGGGTTAGCTATTTTA
GATACTTCGGGTGCCATAGATGTCTTTGTTGTACATGGCAGCTATGGTCTTAATTATTACAAGGTCAATCCTTG
TGAAGATGTTAATCAACTGTTTGTAGTGTCTGGTGGTAATATAGTTGGCATTCTTACTTCTAGAAATGAAACAG
GTTCTGAACAGGTTGAGAACCAGTTTTATGTTAAGTTAACCAATAGCTCACATCGTCGTAGGCGT
>CK_CH_GD_HY_LH-ZDM_20230308.seq
ATGTTGGGGAAGTCACTGTTTTTAGTGACCATTTGGTGTGCACTATGTAGTGCAAATTTGTTTGATCCTGCTA
ATACTTATGTGTACTACTACCAAAGTGCCTTTAGGCCTCCAAATGGGTGGCACCTACAAGGGGGTGCTTATGC
AGTAGTCAATTCTACTAATTATACTAATAATGCCGGTTCTGCAGATTCTTGCACTGTTGGTATTATTAAGGACGT
CTATAATCAAAGTGCGGCTTCCATAGCTATGACAGCACCCCCTCAGGGTATGGCTTGGTCTAAGTCACAATTT
TGTAGTGCACACTGTAACTTTTCTGAAATTACAGTTTTTGTCACACATTGTTATAGTAGTGGTGCAGGTTCTTG
TCCTATAACAGGCATGATTGCACGTGATCATATTCGTATTTCTGCAATGAAAAATGGTTCTTTATTTTATAATTTA
ACAGTTAGCGTATCTAAATACCCTACGTTTAAATCTTTTCAATGTGTTAACAATTTGACATCTGTTTATCTAAAT
GGTGATCTTGTTTTTACTTCTAACAAAACTGCTGATGTTACGTCAGCAGGTGTGTATTATAAAGCAGGTGGAC
CCGTAAATTATAGTGTTATGAAAGAATTTAAGGTTCTTGCTTACTTTGTTAATGGTACAGCACAAGATGTAATT
TTGTGCGATAATTCACCGAAGGGTTTGCTAGCTTGTCAATATAACACTGGCAATTTCTCAGATGGCTTTTATCC
TTTTACTAATAGTACTTTAGTTAGGGAAAAGTTCACCGTATATCGCGAAAGTAGTGTTAATACTACTCTGGCGT
TAACTAATTTCACTTTTTCTAATGTAAGTAATGCACGGCCTAATAGTGGTGGTGTTAATACTTTTCATTTATATCA
AACACAAACAGCTCAGAGTGGTTATTATAATTTTAATTTGTCATTTCTGAGTCAGTTTGTGTATAAGGCAAGT
GATTTCATGTATGGGTCCTATCACCCTAGTTGTTCTTTTAGACCAGAAACCATTAATAGTGGTTTGTGGTTTAA
TTCCTTGTCAGTTTCTCTAGCTTATGGACCACTTCAAGGTGGGTGTAAGCAGTCAGTTTTTAGTGGTAAAGCA
ACGTGTTGCTATGCCTACTCTTACAATGGCCCGGTAGCCTGTAAAGGTGTTTATTCAGGCGAATTACAGACTA
ATTTTGAATGTGGATTGCTGATTTATGTTACTAAGAGTGATGGCTCTCGTATACAGACTAGAACAGAGCCCTT
AGTATTAACGCAACACAATTATAATAATATTACTTTAGATAAGTGTGTTGACTATAATATATATGGCAGAGTAGG
CCAAGGTTTTATTACTAATGTGACTGATTCTGCTGCTAATTTTAGTTATTTAGCAGATGGTGGGTTAGCTATTTT
AGATACTTCTGGTGCCATAGATGTTTTTGTTGTACAGGGCAGCTATGGTCGTAATTATTACAAGGTCAATCCTT
GTGAAGATGTTAACCAACAGTTTGTAGTGTCTGGTGGCAATATAGTTGGTATTCTTACTTCTAGAAATGAAAC
AGGTTCTGAACAGGTTGAGAACCAGTTTTATGTTAAGTTAACCAATAGCTCACATCGTCGCAAGCGT
>CK_CH_GD_SG_LH_20230508.seq
ATGTTGGGGAAGTCACTGTTTTTAGTGACCATTTTGTGTGCACTATGTAGTGCAAATTTATTCGATCCTGCTAA
TACTTATGTGTACTACTACCAAAGTGCCTTTAGGCCTCCAAATGGATGGCACCTACAAGGGGGTGCTTATGCA
GTAGTCAATTCCACTAATTATACTAATAATGCCGGTTCTGCACAACATTGCACTGTTGGTGTTATTAAGGACGT
CTATAATCAAAGTGCGGCTTCCATAGCTATGACAGCACCTCTTCAGGGTATGGCTTGGTCTAAGTCACAATTT
TGTAGTGCACACTGTAACTTTTCTGAAATTACAGTTTTTGTCACACATTGTTATAGTAGTGGTAGCGGGTCTTG
TCCTATAACAGGCATGATTGCACGTGATCATATTCGTATTTCTGCAATGAAAAATGGTACTTTATTTTATAATTTA
ACAGTTAGCGTATCTAAATACCCTAATTTTAAATCTTTTCAATGCGTTAATAATCTCACATCTGTTTATCTAAATG
GTGATCTTGTTTTTACTTCCAACAAAACTACTGATGTTACGTCAGCAGGTGTGTATTTTAAAGCAGGTGGACC
TGTAAATTATAGTATTATGAAAGAATTTAAGGTTCTTGCTTACTTTGTTAATGGTACAGCACAAGATGTAATTTT
GTGCGACAATTCCCCCAAGGGTTTGCTAGCTTGTCAATATAACACTGGCAATTTTTCAGATGGCTTTTATCCTT
TTACTAATAGTACTTTAGTTAGGGAAAAGTTCATCGTATATCGCGAAAGTAGTGTTAATACTACTCTGGCGTTA
ACTAATTTCACTTTTACTAATGTAAGTAATGCACAGCCTAATAGTGGTGGTGTTAATACTTTTCATCTATATCAA
ACACAAACAGCTCAGAGTGGTTATTATAATTTTAATTTGTCATTTCTGAGTCAGTTTGTGTATAAGGCAAGTGA

TTTTATGTATGGGTCCTACCACCCTAGTTGTTCTTTTAGACCAGACACCATTAATAGTGGTTTGTGGTTTAATTC
TTTGTCAGTTTCTCTAGCTTACGGACCACTTCAAGGTGGGTGTAAGCAGTCAGTTTTTAGTGGTAGGGCAAC
GTGTTGCTATGCCTACTCTTACAATGGCCCGATAGCCTGTAAAGGTGTTTATTCAGGCGAATTACGGACTAAT
TTTGAATGTGGATTGCTGATTTATGTTACTAAGAGTGATGGTTCTCGTATACAGACTAGAACAGAGCCCTTAG
TATTAACGCAACACAATTATAATAATATTACTTTAGATAAGTGTGTTGACTATAATATATATGGCAGAGTAGGCC
AAGGTTTTATTACTAATGTGACTGATTCTGCTGCTAATTTTAGTTATTTAGCAGATGGTGGGTTAGCTATTTTA
GATACTTCGGGTGCCATAGATGTCTTTGTTGTACAGGGCAGCTATGGTCTTAATTATTACAAGGTCAATCCTTG
TGAAGATGTTAACAAACAGTTTGTAGTGTCTGGTGGCAATATAGTTGGCATTCTTACTTCTAGAAATGAAACA
GGTTCTGAACAGGTTGAGAACCAGTTTTATGTTAAGTTAACCAATAGCTCACATCGTCGCAGGCGT
>CK_CH_GD_YF_CF_20230307.seq
ATGTTGGGGAAGTCACTGTTTTTAGTGACCATTTTGTGTGCACTATGTAGTGCAAATTTGTTTGATCTTGCCA
ATAATTATGTGTACTACTACCAAAGTGCCTTTAGGCCTCCAAATGGATGGCATTTGCAAGGTGGTGCTTATGC
AGTAGTGAATTCTACTAATTATACTAATAATGCCGGTTCTGCAAGTGAGTGCACTGTTGGTATTATTAAGGACG
TCTATAATCAAAGTGCGGCTTCCATAGCTATGACAGCACCTTCTCAGGGTATGGCTTGGTCTAAGTCACAATT
TTGTAGTGCACACTGTAACTTTTCTGAAATTACAGTTTTCGTTACACATTGTTTTAGTAGTGGTACAGGGTCTT
GCCCTATAACAGGCATGATTGCACGTGATCATATTCGTATTTCTGCAATGAAAAATGGTTCTTTATTTTATAACT
TAACAGTTAGCGTATCTAAATACTCTAGGTTTAAGTCTTTTCAATGTGTTAACAACCTCACATCTGTTTATCTAA
ATGGTGATCTTGTTTTTACTTCCAACAAAACTACTGATGTTACGTCAGCAGGTGTGTATTTTAAAGCAGGTGG
ACCTGTAAATTATAGTGTTATGAAGGAATTTAAGGTTCTTGCTTATTTTGTTAATGGTACAGCACAAGATGTAA
TTTTGTGTGACAAGTCCCCCAAGGGTTTGCTAGCTTGTCAATATAACACTGGCAATTTTTCAGATGGCTTTTAT
CCCTTTACTAATACTACTTTAGTTAGGGAAAAGTTCATTGTATATCGTGAAAGTAGTGTTAATACTACTTTGAC
GTTAACTAATTTCACTTTTGCTAATGAAAGTAATGCACAGCCTAATAGTGGTGGTGTTAGTACTTTTCATTTATA
TCAAACACAAACAGCTCAGAGTGGTTATTATAATTTTAATTTGTCATTTCTGAGTCAGTTTGTGTATAAGGCAA
GTGATTTTATGTATGGGTCTTACCACCCTAAGTGTTCTTTTAGACCAGAAACCATTAATAGTGGTTTATGGTTT
AATTCCTTGTCAGTTTCTCTTACTTATGGACCCCTACAGGGAGGGTGTAAGCAATCTGTTTTTGGTGGTAAGG
CAACGTGTTGTTATGCCTACTCTTATAATGGCCCAAGGGCATGTAAAGGTGTTTATTCAGGTGAATTAAGCAA
GACTTTTGAATGTGGATTGCTGGTTTATGTTACTAAGAGTGATGGTTCTCGTATACAGACTAGAACAGAGCCC
TTAGTATTAACGCAACACAATTATAATAATATTACTTTAGATAAGTGTGTTGACTATAATATATATGGCAGAGTAG
GCCAAGGTTTTATTACTAATGTGACTGATTCTGCTGCTAATTTTAGTTATTTAGCAGATGGTGGGTTAGCTATT
TTAGATACTTCGGGTGCCATAGATGTCTTTGTTGTACAGGGCAGCTATGGTCTTAATTATTACAAGGTCAATCC
TTGTGAAGATGTTAACAAACAGTTTGTAGTGTCTGGTGGCAATATAGTTGGCATTCTTACTTCTACAAATGAA
ACAGGTTCTGAACAGGTTGAGAACCAGTTTTATGTTAAGTTAACCAATAGCTCACATCGTCGCAGGCGT
>CK_CH_SD_LC_YYMY-12dong_20231113.seq
ATGTTGGGGAAGTCACTGTTTTTAGTGACCATTTTGTGTGCACTATGTAGTGCAAATTTGTTTGATTTTGCCA
ATAATTATGTGTACTACTACCAAAGTGCCTTTAGGCCTCCTACTGGATGGCATTTGCAAGGGGGTGCTTATGC
AGTAGTGAATTCTACTAATTACACTAGTAATGCTGGTTCTGCAAGTGAGTGCACTGTTGGTGTTATTAAGGAC
GTTTATAATCAAAGTGCGGCTTCCATAGCTATGACAGCACCTCCTCAGGGTATGGCTTGGTCTAAGTCACAAT
TTTGTAGTGCACACTGTAACTTTTCTGAAATTACAGTTTTTGTCACACATTGTTATAGTAGTGGTACAGGGTCT
TGTCCTCTAACAGGCCTGATTGCACGTGATCATATTCGTATTTCAGCAATGAAAAACGGTTCTTTATTTTATAAT
TTAACAGTTAGCGTATCTAAATACCCTAGATTTAAGTCTTTTCAATGTGTTAACAACTTCACATCTGTTTATTTA
AATGGTGATCTTGTTTTTACTTCTAATAAAACTACTGATGTTACGTCAGCAGGTGTGTATTTTAAAGCAGGTG
GACCTGTAAACTATAGTGTTATGAAAGAATTTAAGGTTCTTGCTTACTTTCTTAATGGTACAGCACAAGATGTA
ATTTTGTGTGACAATTCCCCTAAGGGTTTGCTAGCCTGTCAATATAATACTGGCAATTTTTCAGACGGCTTTTA
TCCTTTTACTAATAGTACTTTAGTTAGGGACAAGTTCATTGTCTATCGTGAAAGTAGTGTTAATACTACTTTGAC

GTTAACTAATTTCACTTTTACTAATGTAAGTACTGCACAGCCTAATAGTGGTGGTGTTAGTACTTTTCATCTATA
TCAAACACAAACAGCTCAGAGTGGTTATTATAATTTTAATTTGTCATTTCTGAGTCAGTTTGTGTATAAGGCAA
GTGATTTTATGTATGGGTCTTATCATCCTAGGTGTTCTTTTAGACCAGAAACCATTAATAGTGGCTTATGGTTTA
ATTCTTTGTCAGTTTCTCTTACTTATGGACCCCTACAGGGAGGGTGTAAGCAATCTGTTTTTAGTGGTAGGGC
AACGTGTTGTTATGCCTACTCTTATAATGGCCCTAGGGCATGTAAAGGTGTTTATTCAGGTGAATTAAGCAAG
ACTTTTGAATGTGGATTGCTGGTTTATGTTACTAAGAGTGATGGCTCTCGTATACAGACTAGAACAGAGCCCT
TAGTACTAACGCAACACAATTATAATAATATCACTTTAGATAAGTGTGTTGACTATAATATATATGGCAGAGTAG
GCCAAGGTTTTATTACTAATGTGACTGACTCTGCTGCTAATTCTAGTTATTTAGCAGATGGTGGGTTAGCTATT
TTAGATACTTCGGGTGCCATAGATGTCTTTGTTGTACAGGGCAGCTATGGTCCTAATTATTACAAGGTCAATCC
TTGTGAAGATGTTAACCAACAGTTTGTAGTGTCTGGTGGCAATATAGTGGGCATTCTTACTTCTAGAAATGAA
ACAGGTTCTGAACAGGTTGAGAACCAGTTTTATGTTAAGTTAACCAATAGCTCACATCGTCGTAGGCGT
>CK_CH_SD_LY_LCH-2_20231010.seq
ATGTTGGGGAAGTCACTGTTTTTAGTGACCATTTTGTGTGCACTATGTAGTGCAAATTTGTTTGATTCTGCCA
ATAATTATGTGTACTACTACCAAAGTGCCTTTAGGCCTCCAGAAGGATGGCATTTGCAAGGGGGTGCTTATGC
AGTAGTGAATTCTACTAATTATACTAGTAATGCCGGTTCTGCAAGTGAGTGCACTGTTGGTATTATTAAGGACG
TCTATAATCAAAGTGCGGCTTCCATAGCTATGACAGCACCTCCTCAGGGTATGGCTTGGTCTAAGTCACAATT
TTGTAGTGCACACTGTAACTTTTCTGAAATTACAGTTTTTGTCACACATTGTTATAGTAGTGGTGCAGGGTCTT
GCCCTATAACAGGCATGATTGCACGTGACCATATTCGTATTTCTGCAATGAAAAATGGTTCTTTATTTTATAACT
TAACAGTTAGCGTATCTAAATACCCTAGATTTAAGTCTTTTCAATGTGTTAACAACTTCACATCTGTTTATTTAA
ATGGTGATCTTGTTTTTACTTCTAATAAAACTACTGATGTTACGTCAGCAGGTGTGTATTTTAAAGCAGGTGG
ACCTGTAAACTATAGTGTTATGAAAGAATTTAAGGTTCTTGCTTACTTTGTTAATGGTACAGCACAAGATGTAA
TTTTGTGTGACAATTCCCCTAAGGGTTTGCTAGCTTGTCAATATAACACTGGCAATTTTTCAGACGGCTTTTAT
CCTTTTACTAATAGTACTTTAGTTAGGGACAAGTTCATTGTCTATCGTGAAAGTAGTGTTAATACTACTTTGAC
GTTAACTAATTTCACTTTTACTAATGTAAGTACTGCACAGCCTAATAGTGGTGGTGTTAGTACTTTTCATCTATA
CCAAACACAAACAGCTCAGAGTGGTTATTATAATTTTAATTTGTCATTTCTGAGTCAGTTTGTGTATAAGGCAA
GTGATTTTATGTATGGGTCTTATCATCCTAGGTGTTCTTTTAGACCAGAAACCATTAATAGTGGTTTATGGTTTA
ATTCCTTGTCAGTTTCTCTTACTTATGGACCCCTACAGGGAGGGTGTAAGCAATCTGTTTTTAGTGGTAAGGC
AACGTGTTGTTATGCCTACTCTTATAATGGCCCTAGGGCATGTAAAGGTGTTTATTCAGGTGAATTAAGCAAG
ACTTTTGAATGTGGCTTGCTGGTTTATGTTACTAAGAGTGATGGCTCTCGTATACAGACTAGAACAGAGCCCT
TAGTATTAACGCAACACAATTATAATAATATTACTTTAGATAAGTGTGTTGACTATAATATATATGGCAGAGTAG
GCCAAGGTTTTATTACTAATGTGACTGATTCTGCTGCTAATTCTAGTTATTTAGCAGATGGTGGGTTAGCTATT
TTAGATACTTCGGGTGCCATAGATGTCTTTGTTGTACAGGGCAGCTATGGTTTTAATTATTACAAGGTCAATCC
TTGTGAAGATGTTAATCAACAGTTTGTAGTGTCTGGTGGCAATATAGTTGGCATGCTTACTTCTAGAAATGAA
ACAGGTTCTGAACAGGTTGAGAACCAGTTTTATGTTAAGTTAACCAATAGCTCACATCGTCGTAGGCGT
>CK_CH_XJ_CJ_TK-YM3C-1#2#_20230831.seq
ATGTTGGGGAAGTCACTGTTTTTAGTGACCATTTTGTGTGCACTATGTAGTGCAAATTTGTTTGATTCTGCCA
ATAATTATGTGTACTACTACCAAAGTGCCTTTAGGCCTGCACAAGGATGGCATTTGCAAGGGGGTGCTTATGC
AGTAGTGAATTCTACTAATTATACTAATAATGCCGGTTCTGCAAGTGAGTGCACTGTTGGTATTATTAAGGACG
TCTATAATCAAAGTGCGGCTTCCATAGCTATGACAGCACCTCCTCAGGGTATGGCTTGGTCTAAGTCACAATT
TTGTAGTGCACACTGTAACTTTTCTGATATTACAGTTTTTGTCACACATTGTTATAGTAGTGGTGCAGGGTCTT
GCCCTATAACAGGCATGATTGCACGTGACCATATTCGTATTTCTGCAATGAAAAATGGTTCTTTATTTTATAACT
TAACAGTTAGCGTATCTAAATACACTAGATTTAAGTCTTTTCAATGTGTTAACAACTTCACAGCTGTTTATTTAA
ATGGTGATCTTGTTTTTACTTCTAATAAAACTACTGATGTTACGTCAGCAGGTGTGTATTTTAAAGCAGGTGG
ACCTGTAAACTATAGTGTTATGAAAGAATTTAAGGTTCTTGCTTACTTTGTTAATGGTACAGCACAAGATGTAA

TTTTGTGTGACAATTCCCCTAAGGGTTTGCTAGCTTGTCAATATAACACTGGCAATTTTTCAGACGGCTTTTAT
CCTTTTACTAATAGTACTTTAGTTAGGGACAAGTTCATTGTCTATCGTGAAAGTAGTGTTAATACTACTTTGAC
GTTAACTAATTACACTTTTACTAATGTAAGTACTGCACAGCCTAATAGTGGTGATGTTAGTACTTTTCATCTATAT
CAAACACAAACAGCTCAGAGTGGTTATTATAATTTTAATTTGTCATTTCTGAGTCAGTTTGTGTATAAGGCAA
GTGATTTTATGTATGGGTCTTATCATCCTAGTTGTTCTTTTAGACCAGAAACCATTAATAGTGGTTTATGGTTTA
ATTCCTTGTCAGTTTCTCTTACTTATGGACCCCTACAGGGAGGGTGTAAGCAATCTGTTTTTAGTGGTAAGGC
AACGTGTTGTTATGCCTACTCTTATAATGGCCCTAGGGCATGTAAAGGTGTTTATTCAGGTGAATTAAGCAAG
ACTTTTGAATGTGGATTGCTGGTTTATGTAACTAAGAGTGATGGCTCTCGTATACAAACTAGAAGTGAACCAC
TGGTGTTAACTCAATATAATTATAACAACATTACTTTAAATAAGTGTGTTGATTATAATATATATGGTAGAGTTGG
TCAAGGTTTTATTACTAATGTAACTGAAGCAACTGCTAATTATAGTTATCTAGCAGATGGTGGTTTAGCTATTTT
AGATACTTCAGGAGCCATAGACATATTTGTTGTTCGAGGTGCATATGGTCTTAATTATTATAAGGTTAATCCCT
GTGAAGATGTCAACCAGCAGTTTGTAGTTTCTGGTGGTAAATTAGTAGGTATTCTTACTTCACGTAATGAGAC
TGGTTCCCAGCTTCTTGAGAATCAGTTTTACATCAAACTCACTAACGGAACACGTCGCTCTAGACGT
>CK_CH_XJ_CJ_TK-SP1-7#8#_20230831.seq
ATGTTGGGGAAGTCACTGTTTTTAGTGACCATTTTGTGTGCACTATGTAGTGCAAATTTGTTTGATTCTGCCA
ATAATTATGTGTACTACTACCAAAGTGCCCTTAGGCCTGCACAAGGATGGCATTTGCAAGGGGGTGCTTATGC
AGTAGTGAATTCTACTAATTATACTAATAATGCCGGTTCTGCAAGTGAGTGCACTGTTGGTATTATTAAGGACG
TCTATAATCAAAGTGCGGCTTCCATAGCTATGACAGCACCTCCTCAGGGTATGGCTTGGTCTAAGTCACAATT
TTGTAGTGCACACTGTAACTTTTCTGATATTACAGTTTTTGTCACACATTGTTATAGTAGTGGTGCAGGGTCTT
GCCCTATAACAGGCATGATTGCACGTGACCATATTCGTATTTCTGCAATGAAAAATGGTTCTTTATTTTATAACT
TAACAGTTAGCGTATCTAAATACACTAGATTTAAGTCTTTTCAATGTGTTAACAACTTCACAGCTGTTTATTTAA
ATGGTGATCTTGTTTTTACTTCTAATAAAACTACTGATGTTACGTCAGCAGGTGTGTATTTTAAAGCAGGTGG
ACCTGTAAACTATAGTGTTATGAAAGAATTTAAGGTTCTTGCTTACTTTGTTAATGGTACAGCACAAGATGTAA
TTTTGTGTGACAATTCCCCTAAGGGTTTGCTAGCTTGTCAATATAACACTGGCAATTTTTCAGACGGCTTTTAT
CCTTTTACTAATAGTACTTTAGTTAGGGACAAGTTCATTGTCTATCGTGAAAGTAGTGTTAATACTACTTTGAC
GTTAACTAATTACACTTTTACTAATGTAAGTACTGCACAGCCTAATAGTGGTGATGTTAGTACTTTTCATCTATAT
CAAACACAAACAGCTCAGAGTGGTTATTATAATTTTAATTTGTCATTTCTGAGTCAGTTTGTGTATAAGGCAA
GTGATTTTATGTATGGGTCTTATCATCCTAGTTGTTCTTTTAGACCAGAAACCATTAATAGTGGTTTATGGTTTA
ATTCCTTGTCAGTTTCTCTTACTTATGGACCCCTACAGGGAGGGTGTAAGCAATCTGTTTTTAGTGGTAAGGC
AACGTGTTGTTATGCCTACTCTTATAATGGCCCTAGGGCATGTAAAGGTGTTTATTCAGGTGAATTAAGCAAG
ACTTTTGAATGTGGATTGCTGGTTTATGTAACTAAGAGTGATGGCTCTCGTATACAAACTAGAAGTGAACCAC
TGGTGTTAACTCAATATAATTATAACAACATTACTTTAAATAAGTGTGTTGATTATAATATATATGGTAGAGTTGG
TCAAGGTTTTATTACTAATGTAACTGAAGCAACTGCTAATTATAGTTATCTAGCAGATGGTGGTTTAGCTATTTT
AGATACTTCAGGAGCCATAGACATATTTGTTGTTCGAGGTGCATATGGTCTTAATTATTATAAGGTTAATCCCT
GTGAAGATGTCAACCAGCAGTTTGTAGTTTCTGGTGGTAAATTAGTAGGTATTCTTACTTCACGTAATGAGAC
TGGTTCCCAGCTTCTTGAGAATCAGTTTTACATCAAACTCACTAACGGAACACGTCGCTCTAGACGT
>CK_CH_XJ_CJ_TK-SP1-5#6#_20230831.seq
ATGTTGGGGAAGTCACTGTTTTTAGTGACCATTTTGTGTGCACTATGTAGTGCAAATTTGTTTGATTCTGCCA
ATAATTATGTGTACTACTACCAAAGTGCCTTAAGGCCTGCACAAGGATGGCATTTGCAAGGGGGTGCTTATGC
AGTAGTGAATTCTACTAATTATACTAATAATGCCGGTTCTGCAAGTGAGTGCACTGTTGGTATTATTAAGGACG
TCTATAATCAAAGTGCGGCTTCCATAGCTATGACAGCACCTCCTCAGGGTATGGCTTGGTCTAAGTCACAATT
TTGTAGTGCACACTGTAACTTTTCTGATATTACAGTTTTTGTCACACATTGTTATAGTAGTGGTGCAGGGTCTT
GCCCTATAACAGGCATGATTGCACGTGACCATATTCGTATTTCTGCAATGAAAAATGGTTCTTTATTTTATAACT
TAACAGTTAGCGTATCTAAATACACTAGATTTAAGTCTTTTCAATGTGTTAACAACTTCACAGCTGTTTATTTAA

ATGGTGATCTTGTTTTTACTTCTAATAAAACTACTGATGTTACGTCAGCAGGTGTGTATTTTAAAGCAGGTGG
ACCTGTAAACTATAGTGTTATGAAAGAATTTAAGGTTCTTGCTTACTTTGTTAATGGTACAGCACAAGATGTAA
TTTTGTGTGACAATTCCCCTAAGGGTTTGCTAGCTTGTCAATATAACACTGGCAATTTTTCAGACGGCTTTTAT
CCTTTTACTAATAGTACTTTAGTTAGGGACAAGTTCATTGTCTATCGTGAAAGTAGTGTTAATACTACTTTGAC
GTTAACTAATTACACTTTTACTAATGTAAGTACTGCACAGCCTAATAGTGGTGATGTTAGTACTTTTCATCTATAT
CAAACACAAACAGCTCAGAGTGGTTATTATAATTTTAATTTGTCATTTCTGAGTCAGTTTGTGTATAAGGCAA
GTGATTTTATGTATGGGTCTTATCATCCTAGTTGTTCTTTTAGACCAGAAACCATTAATAGTGGTTTATGGTTTA
ATTCCTTGTCAGTTTCTCTTACTTATGGACCCCTACAGGGAGGGTGTAAGCAATCTGTTTTTAGTGGTAAGGC
AACGTGTTGTTATGCCTACTCTTATAATGGCCCTAGGGCATGTAAAGGTGTTTATTCAGGTGAATTAAGCAAG
ACTTTTGAATGTGGATTGCTGGTTTATGTAACTAAGAGTGATGGCTCTCGTATACAAACTAGAAGTGAACCAC
TGGTGTTAACTCAATATAATTATAACAACATTACTTTAAATAAGTGTGTTGATTATAATATATATGGTAGAGTTGG
TCAAGGTTTTATTACTAATGTAACTGAAGCAACTGCTAATTATAGTTATCTAGCAGATGGTGGTTTAGCTATTTT
AGATACTTCAGGAGCCATAGACATATTTGTTGTTCGAGGTGCATATGGTCTTAATTATTATAAGGTTAATCCCT
GTGAAGATGTCAACCAGCAGTTTGTAGTTTCTGGTGGTAAATTAGTAGGTATTCTTACTTCACGTAATGAGAC
TGGTTCCCAGCTTCTTGAGAATCAGTTTTACATCAAACTCACTAACGGAACACGTCGCTCTAGACGT
>CK_CH_XJ_CJ_TK-JMTLK-1#3#_20230831.seq
ATGTTGGGGAAGTCACTGTTTTTAGTGACCATTTTGTGTGCACTATGTAGTGCAAATTTGTTTGATTCTGCCA
ATAATTATGTGTACTACTACCAAAGTGCCTTTAGGCCTGCACAAGGATGGCATTTGCAAGGGGGTGCTTATGC
AGTAGTGAATTCTACTAATTATACTAATAATGCCGGTTCTGCAAGTGAGTGCACTGTTGGTATTATTAAGGACG
TCTATAATCAAAGTGCGGCTTCCATAGCTATGACAGCACCTCCTCAGGGTATGGCTTGGTCTAAGTCACAATT
TTGTAGTGCACACTGTAACTTTTCTGATATTACAGTTTTTGTCACACATTGTTATAGTAGTGGTGCAGGGTCTT
GCCCTATAACAGGCATGATTGCACGTGACCATATTCGTATTTCTGCAATGAAAAATGGTTCTTTATTTTATAACT
TAACAGTTAGCGTATCTAAATACACTAGATTTAAGTCTTTTCAATGTGTTAACAACTTCACAGCTGTTTATTTAA
ATGGTGGTCTTGTTTTTACTTCTAATAAAACTACTGATGTTACGTCAGCAGGTGTGTATTTTAAAGCAGGTGG
ACCTGTAAACTATAGTGTTATGAAAGAATTTAAGGTTCTTGCTTACTTTGTTAATGGTACAGCACAAGATGTAA
TTTTGTGTGACAATTCCCCTAAGGGTTTGCTAGCTTGTCAATATAACACTGGCAATTTTTCAGACGGCTTTTAT
CCTTTTACTAATAGTACTTTAGTTAGGGACAAGTTCATTGTCTATCGTGAAAGTAGTGTTAATACTACTTTGAC
GTTAACTAATTACACTTTTACTAATGTAAGTACTGCACAGCCTAATAGTGGTGATGTTAGTACTTTTCATCTATAT
CAAACACAAACAGCTCAGAGTGGTTATTATAATTTTAATTTGTCATTTCTGAGTCAGTTTGTGTATAAGGCAA
GTGATTTTATGTATGGGTCTTATCATCCTAGTTGTTCTTTTAGACCAGAAACCATTAATAGTGGTTTATGGTTTA
ATTCCTTGTCAGTTTCTCTTACTTATGGACCCCTACAGGGAGGGTGTAAGCAATCTGTTTTTAGTGGTAAGGC
AACGTGTTGTTATGCCTACTCTTATAATGGCCCTAGGGCATGTAAAGGTGTTTATTCAGGTGAATTAAGCAAG
ACTTTTGAATGTGGATTGCTGGTTTATGTAACTAAGAGTGATGGCTCTCGTATACAAACTAGAAGTGAACCAC
TGGTGTTAACTCAATATAATTATAACAACATTACTTTAAATAAGTGTGTTGATTATAATATATATGGTAGAGTTGG
TCAAGGTTTTATTACTAATGTAACTGAAGCAACTGCTAATTATAGTTATCTAGCAGATGGTGGTTTAGCTATTTT
AGATACTTCAGGAGCCATAGACATATTTGTTGTTCGAGGTGCATATGGTCTTAATTATTATAAGGTTAATCCCT
GTGAAGATGTCAACCAGCAGTTTGTAGTTTCTGGTGGTAAATTAGTAGGTATTCTTACTTCACGTAATGAGAC
TGGTTCCCAGCTTCTTGAGAATCAGTTTTACATCAAACTCACTAACGGAACACGTCGCTCTAGACGT
>CK_CH_SC_DY_DK-LFJ_20230625.seq
ATGTTGGGGAAGTCACTGTTTTTAGTGACCATTTTGTGTGCACTATGTAGTGCAAATTTGTTTGCCAATAACTA
TGTGTACTACTACCAAAGTGCCTATAGGCCTCCAAATGGATGGCATTTGCAAGGGGGTGCTTATGCAGTAGT
GAATTCTACTAATTATACTAGTAATGCCGGTCCTGCAAGTGTGTGCACTGTTGGTATTATTAAGGACGTCTATA
ATCAAAGTGCAGCTTCCATAGCTATGACAGCACCTCCTCAGGGTATGGCTTGGTCTAAGTCACAATTTTGTAG
TGCACACTGTAACTTTTCTGAAATTACAGTTTTTGTCACACATTGTTATAGTAGTGGTGCAGGTTCTTGTCCTA

TAACAGGCATGATTGCAAGTGGTCATATTCGTATTTCTGCAATGAAAAATGGTTCTTTATTTTATAATTTAACAG
TTAGCGTATCTAAATACCCTAGGTTTAAGTCTTTTCAATGTGTTAACAACTTCACATCTGTCTATCTAAATGGTG
ATCTTGTTTTTACTTCCAACAAAACTACTGATGTTACGTCAGCAGGTGTGTATTTTAAAGCAGGTGGACCTGT
AAATTATAGTGTTATGAAAGAATTTAAGGTTCTTGCTTATTTTGTTAATGGTACAGCACAAGATGTAATTTTGT
GTGACAAGTCCCCAAAGGGTTTGCTAGCTTGTCAATATAACACTGGCAATTTTTCAGATGGCTTTTATCCTTTT
ACTAATACTACTTTAGTTAGGGAAAAGTTCATCGTATATCGTGAAAGTAGTGTTAATACTACTCTGGCGTTAAC
TAATTTCACTTTTACTAATGTAAGTGATGCACAGCCTAATAGAGGTGGTGTTGATACTTTTCATTTATATCAAAC
ACAAACAGCTCAGAGTGGTTATTATAATTTTAATTTCTCATTTCTGAGTCAGTTTGTGTATAAGGCAAGTGATT
TTATGTATGGGTCTTATCATCCTAGTTGTTCTTTTAGACCAGAAACCATTAATAATGATTTGTGGTTTAACTCCT
TGTCAGTTTCTCTTACTTATGGACCCCTACAGGGAGGGTGTAAGCAATCTGTTTTTAGTGGTAAGGCAACGT
GTTGTTATGCCTACTCTTATAATGGCCCTAGAGTATGTAAAGGTGTTTATTCAGGTGAATTAAGCAAGACTTTT
GAATGTGGATTGCTGGTTTATGTTACTAAGAGTGATGGCTCTCGTATACAGACTAGAATAGAGCCCTTAGTATT
AATGCAACACAATTATAATAATATTACTTTAGATAAGTGTGTTAACTATAATATATATGGCAGAGTGGGCCAAG
GTTTTATTACTAATGTGACTGATTCCGCTGCTAATTTTAGTTATTTAGCAGATGGTGGGTTAGCTATTTTAGACA
CTTCGGGTGCCATAGATGTTTTTGTTGTACAGGGCAGCTATGGTCCTAATTATTACAAGGTCAATCCTTGTGA
AGATGTTAACCAACAGTTTGTAGTGTCTGGTGGCAATATAGTTGGCATTCTTACTTCTAGAAATGAAACAGGT
TCTGAACAGGTTGAGAACCAGTTTTATGTTAAGTTAACCAATAGCTCACATCGTCGTAGGCGC
>CK_CH__GS_SYNM-MB_XY-2#_21d_20230821.seq
ATGTTGGGGAAGTCACTGTTTTTAGTGACCATTTTGTGTGCACTATGTAGTGCAAATTTGTTTGATTTGGCCA
ATAATTATGTGTACTACTACCAAAGTGCCTTTAGGCCTCCAGATGGATGGCATTTGCAAGGGGGTGCTTATGC
AGTAGTGAATTCTACTAATTATACTAGTAATGCCGGTTCTGCAAGTGAGTGCACTGTTGGTATTATTAAGGACG
TCTATAATCAAAGTGCGGCTTCCATAGCTATGACAGCACCTTCTCAGGGTATGGCTTGGTCTAAGTCACAATT
TTGTAGTGCACACTGTAACTTTTCTGAAATTACAGTTTTCGTCACACATTGTTATAGTAGTGGTACAGGGTCTT
GCCCTATAACAGGCATGATTGCACGTGATCATATTCGTATTTCTGCAATGAAAAATGGTTCTTTATTTTATAACT
TAACAGTTAGCGTATCTAAATACTCTAGGTTTAAGTCTTTTCAATGTGTTAATAACTTCACATCTGTTTATCTAAA
TGGTGATCTTGTTTTTACTTCCAACAAAACTACTGATGTTACGTCAGCAGGTGTGTATTTTAAAGCAGGTGGA
CCTGTAAATTATAGTGTTATGAAAGAATTTAAGGTTCTTGCTTATTTTGTTAATGGTACAGCACAAGATGTAAT
TTTGTGTGACAAGTCCCCCAAGGGTTTGCTAGCTTGTCAATATAACACTGGCAATTTTTCAGATGGCTTTTAT
CCTTTTACTAATACTACTTTAGTTAGGGAAAAGTTCATTGTGTATCGTGAAAGTAGTGTTAATACTACTCTGGC
GTTAACTAATTTCACTTTTACTAATGTAAGTAATGCACAGCCTAATAGTGGTGGTGTTAATACTTTTCATTTATAT
CAAACACAAACAGCTCAGAGTGGTTATTATAATTTTAATTTGTCATTTCTGAGTCAGTTTGTGTATAAGGCAA
GTGATTTTATGTATGGGTCTTATTATCCTAGGTGTTCTTTTAGACCAGAAACCATTAATAATGGTTTGTGGTTTA
ATTCCTTGTCAGTTTCTCTTACTTATGGACCCCTACAGGGAGGGTGTAAGCAATCTGTTTTTAGTGGTAAGGC
AACGTGTTGTTATGCCTACTCTTATAATGGCCCTAGAGCATGCAAAGGTGTTTATTCAGGCGAATTAAGCAAG
ACTTTTGAATGTGGATTGCTGGTTTATGTTACTAAGAGTGATGGCTCTCGTATACAAACTAGAACGGAGCCCT
TAGTATTAACGCAACACAATTATAATAATATTACTTTAGATAAGTGTGTTAACTATAATATATATGGCAGAGTTGG
CCAAGGTTTTATTACTAATGTGACTGATTCTGCTGCTAATTTTAGTTATTTAGCAGATGGTGGGTTAGCTATTTT
AGACACTTCGGGCGCCATAGATGTTTTTGTTGTACAGGGCAGCTATGGTCCTAATTATTACAAGGTCAATCCT
TGTGAAGATGTTAACCAACAGTTTGTAGTGTCTGGTGGCAATATAGTTGGCATTCTTACTTCTAGAAATGAAA
CAGGTTCTGAACAGGTTGAGAACCAGTTTTATGTTAAGTTAACCAATAGCTCACATCGTCGTAGGCGT
>CK_CH__GS_SYNM-MB_SJ-4-7#_37d_20230821.seq
ATGTTGGGGAAGTCACTGTTTTTAGTGACCATCTTGTGTGCACTATGTAGTGCAAATTTGTTTGATTTGGCCA
ATAATTATGTGTACTACTACCAAAGTGCCTTTAGGCCTCCAGATGGATGGCATTTGCAAGGGGGTGCTTATGC
AGTGGTGAATTCTACTAATTATACTAGTAATGCCGGTTCTGCAAGTGAGTGCACTGTTGGTATTATTAAGGAC

GTCTATAATCAAAGTGCGGCTTCCATAGCTATGACAGCACCTTCTCAGGGTATGGCTTGGTCTAAGTCACAAT
TTTGTAGTGCACACTGTAACTTTTCTGAAATTACAGTTTTCGTCACACATTGTTATAGTAGTGGTACAGGGTCT
TGCCCTATAACAGGCATGATTGCACGTGATCATATTCGTATTTCTGCAATGAAAAATGGTTCTTTATTTTATAAC
TTAACAGTTAGCGTATCTAAATACTCTAGGTTTAAGTCTTTTCAATGTGTTAATAACTTCACATCTGTTTATCTAA
ATGGTGATCTTGTTTTTACTTCCAACAAAACTACTGATGTTACGTCAGCAGGTGTGTATTTTAAAGCAGGTGG
ACCTGTAAATTATAGTGTTATGAAAGAATTTAAGGTTCTTGCTTATTTTGTTAATGGTACAGCACAAGATGTAA
TTTTGTGTGACAAGTCCCCCAAGGGTTTGCTAGCTTGTCAATATAACACTGGCAATTTTTCAGATGGCTTTTAT
CCTTTTACTAATACTACTTTAGTTAGGGAAAAGTTCATTGTGTATCGTGAAAGTAGTGTTAATACTACTCTGGC
GTTAACTAATTTCACTTTTACTAATGTAAGTAATGCACAGCCTAATAGTGGTGGTGTTAATACTTTTCATTTATAT
CAAACACAAACAGCTCAGAGTGGTTATTATAATTTTAATTTGTCATTTCTGAGTCAGTTTGTGTATAAGGCAA
GTGATTTTATGTATGGGTCTTATTATCCTAGTTGTTCTTTTAGACCAGAAACCATTAATAATGGTTTGTGGTTTA
ATTCCTTGTCAGTTTCTCTTACTTATGGACCCCTACAGGGAGGGTGTAAGCAATCTGTTTTTAGTGGTAAGGC
AACGTGTTGTTATGCCTACTCTTATAATGGCCCTAGAGCATGCAAAGGTGTTTATTCAGGCGAATTAAGCAAG
ACTTTTGAATGTGGATTGCTGGTTTATGTTACTAAGAGTGATGGCTCTCGTATACAAACTAGAACGGAGCCCT
TAGTATTAACGCAACACAATTATAATAATATTACTTTAGATAAGTGTGTTAACTATAATATATATGGCAGAGTTGG
CCAAGGTTTTATTACTAATGTGACTGATTCTGCTGCTAATTTTAGTTATTTAGCAGATGGTGGGTTAGCTATTTT
AGACACTTCGGGCGCCATAGATGTTTTTGTTGTACAGGGCAGCTATGGTCCTAATTATTACAAGGTCAATCCT
TGTGAAGATGTTAACCAACAGTTTGTAGTGTCTGGTGGCAATATAGTTGGCATTCTTACTTCTAGAAATGAAA
CAGGTTCTGAACAGGTTGAGAACCAGTTTTATGTTAAGTTAACCAATAGCTCACATCGTCGTAGGCGT
>CK_CH_SD_LY_FX-XZ1C-3_20230516.seq
ATGTTGGGGAAGTCACTGTTTTTAGTGACCATCTTGTGTGCACTATGTAGTGCAAATTTGTTTGATCTGGCCA
ATAATTATGTGTACTACTACCAAAGTGCCTTTAGGCCTCCAAATGGATGGCATTTGCAAGGGGGTGCTTATGC
AGTAGTGAATTCTACTAATTATACTAGTAATGCCGGTTCTGCAAGTGAGTGCACTGTTGGTGTTATTAAGGAC
GTCTATAATCAAAGTGCGGCTTCCATAGCTATGACAGCACCTTCTCGGGGTATGGCTTGGTCTAAGTCACAAT
TTTGTAGTGCACACTGTAACTTTTCTGAAATTACAGTTTTCGTCACACATTGTTATAGTAGTGGTACAGGGTCT
TGCCCTATAACAGGCATGATTGCACGTGATCATATTCGTATTTCTGCAATGAAAAATGGTTCTTTATTTTATAAC
TTAACAGTTAGCGTATCTAAATACTCTAGGTTTAAGTCTTTTCAATGTGTTAACAACCTCACATCTGTTTATCTA
AATGGTGATCTTGTTTTTACTTCTAACAAAACTACTGATGTTACGTCAGCAGGTGTGTATTTTAAAGCAGGTG
GACCTGTAAATTATAGTGTTATGAAAGAATTTAAGGTTCTTGCTTATTTTGTTAATGGTACAGCACAAGATGTA
ATTTTGTGTGACAAGTCCCCCAAGGGTTTGCTAGCTTGTCAATATAACACTGGCAATTTTTCAGATGGCTTTT
ATCCTTTTACTAATATTACTTTAGTTAGGGAAAAGTTCATTGTATATCGTGAAAGTAGTGTTAATACTACTCTGG
CGTTAACTAATTTCACTTTTACTAATGTAAGTAATGCACAGCCTAATAGTGGTGGTGTTAATACTTTTCATTTAT
ATCAAACACAAACAGCTCAGAGTGGTTATTATAATTTTAATTTGTCATTTCTGAGTCAGTTTGTGTATAAGGCA
AGTGATTTTATGTATGGGTCTTATCATCCTAGGTGTTCTTTTAGACCAGAAACCATTAATAATGGTTTGTGGTTT
AATTCCTTGTCAGTTTCTCTTACTTATGGACCCCTACAGGGAGGGTGTAAGCAATCTGTTTTTAGTGGTAAGG
CAACGTGTTGTTATGCCTACTCTTATAATGGCCCTAGAGCATGCAAAGGTGTTTATTCAGGCGAATTAAGCAA
GACTTTTGAATGTGGATTGCTGGTTTATGTTACTAAGAGTGATGGCTCTCGTATACAAACTAGAACGGAGCCC
TTAGTATTAACGCAACACAATTATAATAATATTACTTTAGATAAGTGTGTTAACTATAATATATATGGCAGAGTTG
GCCAAGGTTTTATTACTAATGTGACTGATTCTGCTGCTAATTTTAGTTATTTAGCAGATGGTGGGTTAGCTATT
TTAGACACTTCGGGTGCCATAGATGTTTTTGTTGCACAGGGCAGCTATGGTCCTAATTATTACAAGGTCAATC
CTTGTGAAGATGTTAACCAACAGTTTGTAGTGTCTGGTGGCAATATAGTTGGTATTCTTACTTCTAGAAATGA
AACAGGTTCTGAACAGGTTGAGAACCAGTTTTATGTTAAGTTAACCAATAGCTCACATCGTCGTAGGCGC
>CK_CH_XJ_CJ_TK-4_20230111.seq
ATGTTGGGGAAGTCACTGTTCTTAGTGACCATTTTGTGTGCACTATGTAGTGCAAATTTGTTTGATTTGGCCA

ATAATTATGTGTACTACTACCAAAGTGCCTTTAGGCCTCCAAATGGATGGCATTTGCAAGGGGGTGCTTATGC
AGTAGTGAATTCTACTAATTATACTAGTAATGCCGGTTCTGCAAGTGAGTGCACTGTTGGTATTATTAAGGACG
TCTATAATCAAAGTGCGGCTTCCATAGCTATGACAGCACCTTCTCAGGGTATGGCTTGGTCTAAGTCACAATT
TTGTAGTGCACACTGTAACTTTTCTGAAATTACAGTTTTCGTCACACATTGTTATAGTAGTGGTACAGGGTCTT
GCCCTATAACAGGCATGATTGCACGTGATCATATTCGTATTTCTGCAATGAAAAATGGTTCTTTATTTTATAACT
TAACAGTTAGCGTATCTAAATACTCTAGGTTTAAGTCTTTTCAATGTGTTAACAACCTCACATCTGTTTATCTAA
ATGGTGATCTTGTTTTTACTTCCAACAAAACTACTGATGTTACGTCAGCAGGTGTGTATTTTAAAGCAGGTGG
ACCTGTAAATTATAGTGTTATGAAAGAATTTAAGGTTCTTGCTTATTTTGTTAATGGTACAGCACAAGATGTAA
TTTTGTGTGACAAGTCCCCCAAGGGTTTGCTAGCTTGTCAATATAATACTGGCAATTTTTCAGATGGCTTTTAT
CCTTTTACTAATACTACTTTAGTTAGGGAAAAGTTCATTGTATATCGTGAAAGTAGTGTTAATACTACTCTGGC
GTTAACTAATTTCACTTTTACTAATGTAAGTAATGCACAACCTAATAGTGGTGGTGTTAATACTTTTCATTTATAT
CAAACACAAACAGCTCAGAGTGGTTATTATAATTTTAATTTGTCATTTCTGAGTCAGTTTGTGTATAAGGCAA
GTGATTTTATGTATGGGTCTTATTATCCTAGGTGTTCTTTTAGACCAGAAACATTAATAATGGTTTGTGGTTTAA
TTCCTTGTCAGTTTCTCTTACTTATGGACCCCTACAGGGAGGGTGTAAGCAATCTGTTTTTAGTGGTAAGGCA
ACGTGTTGTTATGCCTACTCTTATAATGGCCCTAGAGCATGCAAAGGTGTTTATTCAGGCGAATTAAGCAAGA
CTTTTGAATGTGGATTGCTGGTTTATGTTACTAAGAGTGATGGCTCTCGTATACAAACTAGAACGGAGCCCTT
AGTATTAACGCAACACAATTATAATAATATTACTTTAGATAAGTGTGTTAACTATAATATATATGGCAGAGTTGG
CCAAGGTTTTATTACTAATGTGACTGATTCTGCTGCTAATTTTAGTTATTTAGCAGATGGTGGGTTAGCTATTTT
AGATACTTCGGGTGCCATAGATGTTTTTGTTGTACAGGGCAGCTATGGTCCTAATTATTACAAGGTCAATCCTT
GTGAAGATGTTAACCAACAGTTTGTAGTGTCTGGTGGCAATATAGTTGGCATTCTTACTTCTAGAAATGAAAC
AGGTTCTGAACAGGTTGAGAACCAGTTTTATGTTAAGTTAACCAATAGCTCACATCGTCGTAGGCGT
>CK_CH_SD_ZZ_JJW-1_20231115.seq
ATGTTGGAGAAGTCACTGTTTTTAGTGACCATTTTGTGTGCACTATGTAGTGCAAATTTGTTTGATTTTGCCAA
TAATTATGTGTACTACTACCAAAGTGCCTTTAGGCCTCCAAATGGATGGCATTTGCAAGGGGGTGCTTATGCA
GTAGTGAATTCTACTAATTATACTAGTAATGCCGGTTCTGCAAGTGGGTGCACTGTTGGTATTATTAAGGACGT
CTATAATCAAAGTGCGGCTTCCATAGCTATGACAGCACCTTCTCAGGGTATGGCTTGGTCTAAGTCACAATTT
TGTAGTGCACACTGTAACTTTTCTGAAATTACAGTTTTTGTCACACATTGTTATAGTAGTGGTACAGGGTCTTG
CCCTATAACAGGCCAGATTGCACGTGATCATATTCGTATTTCTGCAATGAAAAATGGTTCTTTATTTTATAACTT
AACAGTTAGCGTATCTAAATACTCTAGGTTTAAGTCTTTTCAATGTGTTAACAACCTCACATCTGTTTATCTAAA
TGGTGATCTTGTTTTTACTTCCAACAAAACTACTGATGTTACGTCAGCAGGTGTGTATTTTAAAGCAGGTGGA
CCTGTAAATTATAGTGTTATGAAAGAATTTAAGGTTCTTGCTTATTTTGTTAATGGTACAGTACAAGATGTAATT
TTGTGTGACAAGTCCCCCAAGGGTTTGCTAGCTTGTCAATATAACACTGGCAATTTTTCAGATGGCTTTTATC
CTTTTACTAATACTACTTTAGTTAGGGAAAAAGTCATTGTATATCGTGAAAGTAGTGTTAATACTACTCTGGCG
TTAACTAATTTCACTTTTACTAATGTAAGTAATGCACAGCCTAATAGTGGTGGTGTTAATACTTTTCATTTATATC
AAACACAAACAGCTCAGAGTGGTTATTATAATTTTAATTTGTCATTTCTGAGTCAGTTTGTGTATAAGGCAAGT
GATTTTATGTATGGTTCTTATTATCCTAGGTGTTCTTTTAGACCAGAAACCATTAATAATGGTTTGTGGTTTAAT
TCCTTGTCAGTTTCACTTACTTATGGACCCCTACAGGGAGGGTGTAAGCAATCTGTTTTTAGTGGTAAGGCAA
CGTGTTGTTATGCCTACTCTTATAATGGCCCTAGAGCATGCAAAGGTGTTTATTCAGGTGAATTAAGCAAGAC
TTTTGAATGTGGATTGCTGGTTTATGTTACTAAGAGTGATGGCTCTCGTATACAAACTAGAACGGAGCCCTTA
GTATTAACGCAACACAATTATAATAATATTACTTTAGATAAGTGTGTTAACTATAATATATATGGCAGAGTGGGC
CAAGGTTTTATTACTAATGTGACTGATTCTGCTGCTAATTTTAGTTATTTAGCAGATGGTGGGTTAGCTATTTTA
GACACTTCGGGTGCCATAGATGTTTTTGTTGTACAGGGCAGCTATGGTCCTAATTATTACAAGGTCACTCCTT
GTGAAGATGTTAATCAACAGTTTGTAGTGTCTGGTGGCAATATAGTTGGCATTCTTACTTCTAGAAATGAAAC
AGGTTCTGAACAGGTTGAGAACCAGTTTTATGTTAAGTTAACCAATAGCTCACATCGTCGTAGGCGT

>CK_CH_YN_KM_SL_DK-HYX_20230330.seq
ATGTTGGGGAAGTCACTGTTCTTAGTGACCATTTTGTGTGCACTATGTAGTGCAAATTTGTTTGATTTGGCCA
ATAATTATGTGTACTACTACCAAAGTGCCTTTAGGCCTCCAAATGGATGGCATTTGCAAGGGGGTGCTTATGC
AGTAGTGAATTCTACTAATTATACTAGTAATGCCGGTTCTGCAAGTGGGTGCACTGTTGGTATTATTAAGGAC
GTCTATAATCAAAGTGCGGCTTCCATAGCTATGACAGCACCTTCTCAGGGTATGGCTTGGTCTAAGTCACAAT
TTTGTAGTGCACACTGTAACTTTTCTGAAATTACAGTTTTCGTCACACATTGTTATAGTAGTGGTACAGGGTCT
TGCCCTATAACAGGCATGATTGCACGTGATCATATTCGTATTTCTGCAATGAAAAATGGTTCTTTATTTTATAAC
TTAACAGTTAGCGTATCTAAATACTCTAGGTTTAAGTCTTTTCAATGTGTTAACAACCTCACATCTGTTTATCTA
AATGGTGATCTTGTTTTTACTTCCAACAAAACTACTGATGTTACGTCAGCAGGTGTGTACTTTAAAGCAGGTG
GACCTGTAAATTATAGTGTTATGAAAGAATTTAAGGTTCTTGCTTATTTTGTTAATGGTACAGCACAAGATGTA
ATTTTGTGTGACAAGTCCCCCAAGGGTTTGCTAGCTTGTCAATATAACACTGGCAATTTTTCAGATGGCTTTT
ATCCTTTTACTAATACTACTTTAGTTAGGGAAAAGTTCATCGTATATCGTGAAAGTAGTGTTAATACTACTCTGG
CGTTAATTAATTTCACTTTTAAATAATGTAAGTAATGCACAGCCTAATAGTGGTGGTGTTAATACTTTTCATTTAT
ATCAAACACAAACAGCTCAGAGTGGTTATTATAATTTTAATTTCTCATTTCTGAGTCAGTTTGTGTATAAGGCA
AGTGATTTTATGTATGGGTCTTATCATCCTAGGTGTTCTTTTAGACCAGAAACCATTAATAATGATTTGTGGTTT
AATTCCTTGTCAGTTTCTCTTACTTATGGACCCCTACAGGGAGGGTGTAAGCAATCTGTTTTTAGTGGTAAGG
CAACGTGTTGTTATGCCTACTCTTATAATGGCCCTAGAGTATGTAAAGGTGTTTATTCAGGTGAATTAAGCAG
GACTTTTGAATGTGGATTGCTGGTTTATGTTACTAAGAGTGATGGCTCTCGTATACAGACTAGAACAGAGCCC
TTAGTATTAACGCAACACAATTATAATAATATTACTTTAGATAAGTGTGTTAACTATAATATATATGGCAGAGTAG
GCCAAGGTTTTATTACTAATGTGACTGATTCTGCTGCTAATTTTAGTTATTTAGCAGATGGTGGGTTAGCTATT
TTAGACACTTCGGGTGCCATAGATGTTTTTGTTGCACAGGGCAGCTATGGTCCTAATTATTACAAGGTCAATC
CTTGTGAAGATGTTAACCAACAGTTTGTAGTGTCTGGTGGCAGTATAGTTGGCATTCTTACTTCTAGAAATGA
AACAGGTTCTGAACAGGTTGAGAACCAGTTTTATGTTAAGTTAACCAATAGCTCACATCGTCGTAGGCGC
>CK_CH_SD_LY_FX-XZ1C-2_20230516.seq
ATGTTGGGGAAGTCACTGTTTTTAGTGACCATTTTGTGTGCACTATGTAGTGCAAATTTGTTTGATTTGGCCA
ATAATTATGTGTACTACTACCAAAGTGCCTTTAGGCCTCCAAATGGATGGCATTTGCAAGGGGGTGCTTATGC
AGTAGTGAATTCTACTAATTATACTAGTAATGCCGGTTCTGCAAGTGGGTGCACTGTTGGTATTATTAAGGAC
GTCTATAATCAAAGTGCGGCTTCCATAGCTATGACAGCACCTTCTCAGGGTATGACTTGGTCTACGTCACAAT
TTTGTAGTGCACACTGTAACTTTTCTGAAATTACAGTTTTCGTCACACATTGTTATAGTAGTGGTACAGGGTCT
TGCCCTATAACAGGCATGATTGCACGTGATTATATTCGTATTTCTGCAATGAAAAATGGTTCTTTATTTTATAAC
TTAACAGTTAGCGTATCTAAATACTCTAGGTTTAAGTCTTTTCAATGTGTTAACAACCTCACATCTGTTTATCTA
AATGGTGATCTTGTTTTTACTTCTAACAAAACTACTGATGTTACGTCAGCAGGTGTGTATTTTAAAGCAGGTG
GACCTGTAAATTATAGTGTTATGAAAGAATTTAAGGTTCTTGCTTATTTTGTTAATGGTACAGCACAAGATGTA
ATTTTGTGTGACAAGTCCCCCAAGGGTTTGCTAGCTTGTCAATATAACACTGGCAATTTTTCAGATGGCTTTT
ATCCTTTTATTAATACTACTTTAGTTAGGGAAAAGTTCATTGTATATCGTGAAAGTAGTGTTAATACTACTCTGG
CGTTAACTAATTTCACTTTTACTAATGTAAGTAATGCACAGCCTAATAGTGGTGGTGTTAATACTTTTCATTTAT
ATCAAACACAAACAGCTCAGAGTGGTTATTATAATTTTAATTTCTCATTTCTGAGTCAGTTTGTGTATAAGGCA
AGTGATTTTATGTATGGGTCTTATCATCCTAAGTGTTCTTTTAGACCAGAAACCATTAATAATGATTTGTGGTTT
AATTCCTTGTCAGTTTCTCTTACTTATGGACCCCTACAGGGAGGGTGTAAGCAATCTGTTTTTAGTGGTAAGG
CAACGTGTTGTTATGCCTACTCTTATAATGGCCCTAGAGTATGTAAAGGTGTTTATTCAGGTGAATTAAGCAAT
ACTTTTGAATGTGGATTGCTGGTTTATGTTACTAAGAGTGATGGCTCTCGTATACAGACTAGAACAGAGCCCT
TAGTATTAACGCAACACAATTATAATAATATTACTTTAGATAAGTGTGTTAACTATAATATATATGGCAGAGTGG
GCCAAGGTTTTATTACTAATGTGACTGATTCTGCTGCTAATTTTAGTTATTTAGCAGATGGTGGGTTAGCTATT
TTAGACACTTCGGGTGCCATAGATGTTTTTGTTGCACAGGGCAGCTATGGTCCTAATTATTACAAGGTCAATC

CTTGTGAAGATGTTAACCAACAGTTTGTAGTGTCTGGTGGCAATATAGTTGGCATTCTTACTTCTAGAAATGA
AACAGGTTCTGAACAGGTTGAGAACCAGTTTTATGTTAAGTTAACCAATAGCTCACATCGTCGTAGGCGC
>CK_CH_CQ_BS-LJT_20230116.seq
ATGTTGGGGAAGTCACTGTTTTTAGTGACCATTTTGTGTGCACTATGTAGTGCAAATTTGTTTGATTTTGCCA
ATAATTATGTGTACTACTACCAAAGTGCCTTTAGGCCTCCAAATGGATGGCATTTGCAAGGGGGTGCTTATGC
AGTAGTGAATTCTACTAATTATACTAGTAATGCCGGTTCTGCAAGTGGGTGCACTGTTGGTATTATTAAGGAC
GTCTATAATCAAAGTGCGGCTTCCATAGCTATGACAGCACCTTCTCAGGGTATGGCTTGGTCTAAGTCACAAT
TTTGTAGTGCACACTGTAACTTTTCTGAAATTACAGTTTTCGTCACACATTGTTATAGTAGTGGTACAGGGTCT
TGCCCTATAACAGGCATGATTGCACGTGATCATATTCGTATTTCTGCAATGAAAAATGGTTCTTTATTTTATAAC
TTAACAGTTAGCGTATCTAAATACTCTAAGTTTAAGTCTTTTCAATGTGTTAACAACCTCACATCTGTTTATCTA
AATGGTGATCTTGTTTTTACTTCCAACAAAACTACTGATGTTACGTCAGCAGGTGTGTATTTTAAAGCAGGTG
GACCTGTAAATTATAGTGTTATGAAAGAATTTAAGGTTCTTGCTTATTTTGTTAATGGTACAGCACAAGATGTA
ATTTTGTGTGACAAGTCCCCCAAGGGTTTGCTAGCTTGTCAATATAACACTGGCAATTTTTCAGATGGCTTTT
ATCCTTTTACTAATACTACTTTAGTTAGGGAAAAGTTCATTGTATATCGTGAAAGTAGTGTTAATACTACTCTGG
CGTTAACTAATTTCACTTTTACTAATGTAAGTAATGCACAGCCTAATAGTGGTGGTGTTAATACTTTTCATTTAT
ATCAAACACAAACAGCTCAGAGTGGTTATTATAATTTTAATTTCTCATTTCTGAGTCAGTTTGTGTATAAGGCA
AGTGATTTTATGTATGGGTCTTATCATCCTAGGTGTTCTTTTAGACCAGAAACCATTAATAATGATTTGTGGTTT
AATTCCTTGTCAGTTTCTCTTACTTATGGACCCCTACAGGGAGGGTGTAAGCAATCTGTTTTTAGTGGTAAGG
CAACGTGTTGTTATGCCTACTCTTATAATGGCCCTAGAGTATGTAAAGGTGTTTATTCAGGTGAATTAAGCAAG
ACTTTTGAATGTGGATTGCTGGTTTATGTTACTAAGAGTGATGGCTCTCGCATACAGACTAGAACAGAGCCCT
TAGTATTAACGCAACACAATTATAATAATATTACTTTAGATAAGTGTGTTAACTATAATATATATGGCAGAGTGG
GCCAAGGTTTTATTACTAATGTGACTGATTCTGCTGCTAATTTTAGTTATTTAGCAGATGGTGGATTAGCTATTT
TAGACACTTCGGGTGCCATAGATGTTTTTGTTGCACAGGGCAGCTATGGTCCTAATTATTACAAGGTCAATCC
TTGTGAAGATGTTAACCAACAGTTTGTAGTGTCTGGTGGCAGTATAGTTGGCATTCTTACTTCTAGAAATGAA
ACAGGTTCTGAACAGGTTGAGAACCAGTTTTATGTTAAGTTAACCAATAGCTCACATCGTCGTAGGCGC
>CK_CH_YN_KM_SL-DK_CLH_20230223.seq
ATGTTGGGGAAGTCACTGTTTTTAGTGACCATTTTGTGTGCACTATGTAGTGCAAATTTGTTTGATTTGGCCA
ATAATTATGTGTACTACTACCAAAGTGCCTTTAGGCCTCCAAATGGATGGCATTTGCAAGGGGGTGCTTATGC
AGTAGTGAATTCTACTAATTATACTAGTAATGCCGGTTCTGCAAGTGAGTGCACTGTTGGTATTATTAAGGACG
TCTATAATCAAAGTGCGTCTTCCATAGCTATGACAGCACCTTCTCAGGGTATGGCTTGGTCTAAGTCACAATTT
TGTAGTGCACACTGTAACTTTTCTGAAATTACAGTTTTCGTCACACATTGTTATAGTAGTGGTACAGGGTCTTG
CCCTATAACAGGCATGATTGCACGTGATCATATTCGTATTTCTGCAATGAAAAATGGTTCTTTATTTTATAACTT
AACAGTTAGCGTATCTAAATACTCTAGGTTTAAGTCTTTTCAATGTGTTAACAACCTCACATCTGTTTATCTAAA
TGGTGATCTTGTTTTTACTTCCAACAAAACTACTGATGTTACGTCAGCAGGTGTGTATTTTAAAGCAGGTGGA
CCTGTAAATTATAGTGTTATGAAAGAATTTAAGGTTCTTGCTTATTTTGTTAATGGTACAGCACAAGATGTAAT
TTTGTGTGACAAGTCCCCCAAGGGTTTGCTAGCTTGTCAATATAACACTGGCAATTTTTCAGATGGCTTTTAT
CCTTTTACTAATACTACTTTAGTTAGGGAAAAGTTCATTGTATATCGTGAAAGTAGTGTTAATACTACTCTGGC
GTTAACTAATTTCACTTTTACTAATGTAAGTAATGCACAGCCTAATAGTGGTGGTGTTAATACTTTTCATTTATAT
CAAACACAAACAGCTCAGAGTGGTTATTATAATTTTAATTTCTCATTTCTGAGTCAGTTTGTGTATAAGGCAAG
TGATTTTATGTATGGGTCTTATCATCCTAGGTGTTCTTTTAGACCAGAAACCATTAATAATGATTTGTGGTTTAA
TTCCTTGTCAGTTTCTCTTACTTATGGACCCCTACAGGGAGGGTGTAAGCAATCTGTTTTTAGTGGTAAGGCA
ACGTGTTGTTATGCCTACTCTTATAATGGCCCTAGAGTATGTAAAGGTGTTTATTCAGGTGAATTAAGCAAGAC
TTTTGAATGTGGATTGCTGGTTTATGTTACTAAGAGTGATGGCTCTCGCATACAGACTAGAACAGAGCCCTTA
GTATTAACGCAACACAATTATAATAATATTACTTTAGATAAGTGTGTTAACTATAATATATATGGCAGAGTGGGC

CAAGGTTTTATTACTAATGTGACTGATTCTGCTGCTAATTTTAGTTATTTAGCAGATGGTGGGTTAGCTATTTTA
GACACTTCGGGTGCCATAGATGTTTTTGTTGCACAGGGCAGCTATGGTCCTAATTATTACAAGGTCAATCCTT
GTGAAGATGTTAACCAACAGTTTGTAGTGTCTGGTGGCAGTATAGTTGGCATTCTTACTTCTAGAAATGAAAC
AGGTTCTGAACAGGTTGAGAGCCAGTTTTATGTTAAGTTAACCAATAGCTCACATCGTCGTAGGCGC
>CK_CH_YN_KM_DK-SYP_20230619.seq
ATGTTGGGGAAGTCACTGTTTTTAGTGACCATTTTGTGTGCACTATGTAGTGCAAATTTGTTTGATTTGGCCA
ATAATTATGTGTACTACTACCAAAGTGCCTTTAGGCCTCCAAATGGATGGCATTTGCAAGGGGGTGCTTATGC
AGTAGTGAATTCTACTAATTATACTAGTAATGCCGGTTCTGCAAGTGAGTGCACTGTTGGTATTATTAAGGACG
TCTATAATCAAAGTGCGTCTTCCATAGCTATGACAGCACCTTCTCAGGGTATGGCTTGGTCTAAGTCACAATTT
TGTAGTGCACACTGTAACTTTTCTGAAATTACAGTTTTCGTCACACATTGTTATAGTAGTGGTACAGGGTCTTG
CCCTATAACAGGCATGATTGCACGTGATCATATTCGTATTTCTGCAATGAAAAATGGTTCTTTATTTTATAACTT
AACAGTTAGCGTATCTAAATACTCTAGGTTTAAGTCTTTTCAATGTGTTAACAACCTCACATCTGTTTATCTAAA
TGGTGATCTTGTTTTTACTTCCAACAAAACTACTGATGTTACGTCAGCAGGTGTGTATTTTAAAGCAGGTGGA
CCTGTAAATTATAGTGTTATGAAAGAATTTAAGGTTCTTGCTTATTTTGTTAATGGTACAGCACAAGATGTAAT
TTTGTGTGACAAGTCCCCCAAGGGTTTGCTAGCTTGTCAATATAACACTGGCAATTTTTCAGATGGCTTTTAT
CCTTTTACTAATACTACTTTAGTTAGGGAAAAGTTCATTGTATATCGTGAAAGTAGTGTTAATACTACTCTGGC
GTTAACTAATTTCACTTTTACTAATGTAAGTAATGCACAGCCTAATAGTGGTGGTGTTAATACTTTTCATTTATAT
CAAACACAAACAGCTCAGAGTGGTTATTATAATTTTAATTTCTCATTTCTGAGTCAGTTTGTGTATAAGGCAAG
TGATTTTATGTATGGGTCTTATCATCCTAGGTGCTCTTTTAGACCAGAAACCATTAATAATGATTTGTGGTTTAA
TTCCTTGTCAGTTTCTCTTACTTATGGACCCCTACAGGGAGGGTGTAAGCAATCTGTTTTTAGTGGTAAGGCA
ACGTGTTGTTATGCCTACTCTTATAATGGCCCTAGAGTATGTAAAGGTGTTTATTCAGGTGAATTAAGCAAGAC
TTTTGAATGTGGATTGCTGGTTTATGTTACTAAGAGTGATGGCTCTCGCATACAGACTAGAACAGAGCCCTTA
GTATTAACGCAACACAATTATAATAATATTACTTTAGATAAGTGTGTTAACTATAATATATATGGCAGAGTGGGC
CAAGGTTTTATTACTAATGTGACTGATTCTGCTGCTAATTTTAGTTATTTAGCAGATGGTGGGTTAGCTATTTTA
GACACTTCGGGTGCCATAGATGTTTTTGTTGCACAGGGCAGCTATGGTCCTAATTATTACAAGGTCAATCCTT
GTGAAGATGTTAACCAACAGTTTGTAGTGTCTGGTGGCAGTATAGTTGGCATTCTTACTTCTAGAAATGAAAC
AGGTTCTGAACAGGTTGAGAACCAGTTTTATGTTAAGTTAACCAATAGCTCACATCGTCGTAGGCGC
>CK_CH_GZ_GY_DK-CXY_20231030.seq
ATGTTGGGGAAGTCACTGTTTTTAGTGACCATTTTGTGTGCACTATGTAGTGCAAATTTGTTTGATTCTGCCA
ATAATTATGTGTACTACTACCAAAGTGCCTTTAGGCCTTCAGATGGATGGCATTTGCAAGGGGGTGCTTATGC
AGTAGTGAATTCTACTAATTATACTAGTAATGCCGGTTCTGCAAGTGAGTGCACTGTTGGTATTATTAAGGACG
TCTATAATCAAAGTGCGGCTTCCATAGCTATGACAGCACCCTCTCAGGGTATGGCTTGGTCTAAGTCACAATT
TTGTAGTGCACACTGTAACTTTTCTGAAATTACAGTTTTCGTCACACATTGTTATAGTAGTGGTACAGGGTCTT
GCCCTATAACAGGCATGATTGCACGTGATCATATTCGTATTTCTGCAATGAAAAATGGTTCTTTATTTTATAACT
TAACAGTTAGCGTATCTAAATACTCTAGGTTTAAGTCTTTTCAATGTGTTAACAACTTCACATCTGTCTATCTAA
ATGGTGATCTTGTTTTTACTTCCAATAAAACTACTGATGTTATGTCAGCAGGTGTGTATTTTAAAGCAGGTGGA
CCTGTAAATTATAGTGTTATGAAAGAATTTAAGGTTCTTGCTTATTTTGTTAATGGTACAGCACAAGATGTAAT
TTTGTGTGACAAGTCCCCCAAGGGTTTGCTAGCTTGTCAATATAACACTGGCAATTTTTCAGATGGCTTTTAT
CCTTTTACTAATACTACTTTAGTTAGGGAAAAGTTCATCGTATATCGTGAAAGTAGTGTTAATACTACTCTGGT
GTTAACTAATTTCACTTTTACTAATGTAAGTAATGCACAGCCTAATAGTGGTGGTGTTAATACTTTTCATTTATAT
CAAACACAAACAGCTCAGAGTGGTTATTATAATTTTAATTTCTCATTTCTGAGTCAGTTTGTGTATAAGGCAAG
TGATTTTATGTATGGGTCTTATCATCCTAGGTGTTCTTTTAGACCAGAAACCATTAATAATGATTTGTGGTTTAA
TTCCTTGTCAGTTTCTCTTACTTATGGACCCCTACAGGGAGGGTGTAAGCAATCTGTTTTTAGTGGTAAGGCA
ACGTGTTGTTATGCCTACTCTTATAATGGCCCTAGAGTATGTAAAGGTGTTTATTCAGGTGAATTAAGCAAGAC

TTTTGAATGTGGATTGCTGGTTTATGTTACTAAGAGTGATGGCTCTCGTATACAGACTAGAACAGAGCCCTTA
GTATTAACGCAACACAATTATAATAATATTACTTTAGATAAGTGTGTTAACTATAATATATATGGCAGAGTGGGC
CAAGGTTTTATTACTAATGTGACTGATTCTGCTGCTAATTTTAGTTATTTAGCAGATGGTGGGTTAGCTATTTTA
GACACTTCGGGTGCCATAGATGTTTTTGTTGCACAGGGCAGCTATGGTCCTAATTATTACAAGGTTAATCCTT
GTGAAGATGTTAACCAACAGTTTGTAGTGTCTGGTGGCAATATAGTTGGCATTCTTACTTCTAGAAATGAAAC
AGGTTCTGAACAGGTTGAGAACCAGTTTTATGTTAAGTTAACCAATAGCTCACATCGTCGTAGGCGC
>CK_CH_GD_HY_LH-HSX_20230330.seq
ATGTTGGGGAAGTCACTGTTTTTAGTGACCATTTTGTGTGCACTATGTAGTGCAAATTTGTTTCATTTTACCAA
TAATTATGTGTACTACTACCAAAGTGCCTATAGGCCTCCAAATGGATGGCATTTGCAAGGGGGTGCTTATGCA
GTAGTGAATTCTACTAATTATACTAGTAATACCGGTTCTGCAAGTGAGTGCACTGTTGGTATTATTAAGGACGT
CTATAATCAAAGTGCGGCTTCCATAGCTATGACAGCACCCTCTCAGGGTATGGCTTGGTCTAAGTCACAATTT
TGTAGTGCACACTGTAACTTTTCTGAAATTACAGTTTTCGTCACACATTGTTATCGTAGTGGCGCAGGGTCTT
GCCCTATAACAGGCATGATTGCACGTGATTATATTCGTATTTCTGCAATGAAAAATGGTTCTTTATTTTATAACT
TAACAGTTAGCGTATCTAAATACTCTAGGTTTAAGTCTTTTCAATGTGTTAACAACTTCACATCTGTCTATCTAA
ATGGTGATCTTGTTTTTACTTCCAACAAAACTACTGATGTTACGTCAGCAGGTGTGTATTTTAAAGCAGGTGG
ACCTGTAAATTATAGTGTTATGAAAGAATTTAAGGTTCTTGCTTATTTTGTTAACGGTACAGTACAAGATGTAA
TTTTGTGTGACAAGTCCCCCAAGGGTTTGCTAGCTTGTCAATATAACACTGGCAATTTTTCAGATGGCTTTTAT
CCTTTTACTAATACTACTTTAGTTAGGGAAAAGTTCATCGTATATCGTGAAAGTAGTGTTAATACTACTCTGGT
GTTAACTAATTTCACTTTTACTAATGTAAGTAATGCACAGCCTAATAGTGGTGGTGTTGATACTTTTCATTTATA
TCAAACACAAACAGCTCAGAGTGGTTATTATAATTTTAATTTCTCATTTCTGAGTCAGTTTGTGTATAAGGCAA
GTGATTTTATGTATGGGTCTTATCATCCTAGGTGTTCTTTTAGACCAGAAACCATTAATAATGATTTGTGGTTTA
ATTCCTTGTCAGTTTCTCTTACTTATGGACCCCTACAGGGAGGGTGTAAGCAATCTGTTTTTAGTGGTAAGGC
AACGTGTTGTTATGCCTACTCTTATAATGGCCCTAGAGTATGTAAAGGTGTTTATTCAGGTGAATTAAGCAAG
ACTTTTGAATGTGGATTGCTGGTTTATGTTACTAAGAGTGATGGCTCTCGTATACAGACTAGAACAGAGCCCT
TAGTATTAACGCAACACAATTATAATAATATTACTTTAGATAAGTGTGTTAACTATAATATATATGGCAGAGTGG
GCCAAGGTTTTATTACTAATGTGACTGATTCTGCTGCTAATTTTAGTTATTTAGCAGATGGTGGGTTAGCTATT
TTAGACACTTCGGGTGCCATAGATGTTTTTGTTGCACAGGGCAGCTATGGTCCTAATTATTACAAGGTTAATC
CTTGTGAAGATGTTAACCAACAGTTTGTAGTGTCTGGTGGCAATATAGTTGGCATTCTTACTTCTAGAAATGA
AACAGGTTCTGAACAGGTTGAGAACCAGTTTTATGTTAAGTTAACCAATAGCTCACATCGTCGTAGGCGC
>CK_CH_SD_LY_YN-DZC-4#_20230404.seq
ATGTTGGGGAAGTCACTGTTGTTAGTGACCATTTTGTGTGCACTATGTAGTGCAAATTTGTTTGATTTGGCCA
ATAATTATGTGTACTACTACCAAAGTGCCTTTAGGCCTCCAAATGGATGGCATTTGCAAGGGGGTGCTTATGC
AGTAGTGAATTCTACTAATTATACTAGTAATGCCGGTTCTGCAAGTGCGTGCACTGTTGGTATTATTAAGGACG
TCTATAATCAAAGTGCGGCTTCCATAGCTATGACAGCACCTTCTCAGGGTATGGCTTGGTCTAAGTCACAATT
TTGTAGTGCACACTGTAACTTTTCTGAAATTACAGTTTTCGTCACACATTGTTATAGTAGTGGTACAGGGTCTT
GCCCTATAACAGGCATGATTGCACGTGATCATATTCGTATTTCTGCAATGAAAAATGGTTTTTTATTTTATAACT
TAACAGTTAGCGTATCTAAATACTCTAGGTTTAAGTCTTTTCAATGTGTTAACAACTTCACATCTGTCTATCTAA
ATGGTGATCTTGTTTTTACTTCCAACAAAACTACTGATGTTACGTCAGCAGGTGTGTATTTTAAAGCAGGTGG
ACCTGTAAATTATAGTGTTATGAAAGAATTTAAGGTTCTTGCTTATTTTGTTAACGGTACAGCACAAGATGTAA
TTTTGTGTGACAAGTCCCCCAAGGGTTTGCTAGCTTGTCAATATAACACTGGCAATTTTTCAGATGGCTTTTAT
CCTTTTACTAATACTACTTTAGTTAGGGAAAAGTTCATCGTATATCGTGAAAGTAGTGTTAATACTACTCTGGC
GTTAACTAATTTCACTTTTACTAATGTAAGTAATGCACAGCCTAATAGTGGTGGTGTTAATACTTTTCATTTATAT
CAAACACAAACAGCTCAGAGTGGTTATTATAATTTTAATTTCTCATTTCTGAGTCAGTTTGTGTATAAGGCAAG
TGATTTTATGTATGGGTCTTATCATCCTAGGTGTTCTTTTAGACCAGAAACCATTAATAATGATTTGTGGTTTAA

TTCCTTGTCAGTTTCTCTTACTTATGGACCCCTACAAGGAGGGTGTAAGCAATCTGTTTTTAGTGGTAAGGCA
ACGTGTTGTTATGCCTACTCTTATAATGGCCCTAGAGTATGTAAAGGTGTTTATTCAGGTGAATTAAGCAAGAC
TTTTGAATGTGGATTGCTGGTTTATGTTACTAAGAGTGATGGCTCTCGTATACAGACTAGAACAGAGCCCTTA
GTATTAACGCAACACAATTATAATAATATTACTTTAGATAAGTGTGTTAACTATAATATATATGGCAGAGTGGGC
CAAGGTTTTATTACTAATGTGACTGATTCTGCTGCTAATTTTAGTTATTTAGCAGATGGTGGGTTAGCTATTTTA
GACACTTCGGGTGCCATAGATGTTTTTGTTGCACAGGGCAGCTATGGTCCTAATTATTACAAGGTCAATCCTT
GTGAAGATGTTAACCAACAGTTTGTAGTGTCTGGTGGCAATATAGTTGGCATTCTTACTTCTAGAAATGAAAC
AGGTTCTGAACAGGTTGAGAACCAGTTTTATGTTAAGTTAACCAATAGCTCACATCGTCGTAGGCGC
>CK_CH_SD_LY_LN-ZXC-2#QHC_20231120.seq
ATGTTGGGGAAGTCACTGTTTTTAGTGACCATTTTGTGTGCACTATGTAGTGCAAATTTGTTTGATTTTGCTAA
TAATTATGTGTACTACTACCAAAGTGCCTTTAGGCCTCCAAAGGGATGGCATTTGCAAGGGGGTGCTTATGCA
GTAGTGAATTCTACTAATTATACTAGTAATGCCGGTTCTGCAAGTGAGTGCACTGTTGGTATTATTAAGGACGT
CTATAATCAAAGTGCGGCTTCCATAGCTATGACAGCACCCTCTCAGGGTATGGCTTGGTCTAAGTCACAATTT
TGTAGTGCGCACTGTAACTTTTCTGAAATTACAGTTTTCGTCACACATTGTTATAGTAGTGGTGCAGGGTCTT
GCCCTATAACAGGCATGATTGCACGTGATCATATTCGTATTTCTGCAATGAAAAATGGTTTTTTATTTTATAACT
TAACAGTTAGCGTATCTAAATACTCTAGGTTTAAGTCTTTTCAATGTGTTAACAACTTCACATCTGTCTATCTAA
ATGGTGATCTTGTTTTTACTTCCAACAAAACTACTGATGTTACGTCAGCAGGTGTGTATTTTAAAGCAGGTGG
ACCTGTAAATTATAGTGTTATGAAAGAATTTAAGGTTCTTGCTTATTTTGTTAATGGTACAGCACAAGATGTAA
TTTTGTGTGACAAGTCCCCCAAGGGTTTGCTAGCTTGTCAATATAACACTGGCAATTTTTCAGATGGCTTTTAT
CCTTTTACTAATACTACTTTAGTTAGGGAAAAGTTCATCGTATATCGTGAAAGTAGTGTTAATACTACTCTGGC
GTTAACTAATTTCACTTTTACTAATGTAAGTAATGCACAGCCTAATAGTGGTGGTGTTAGTACTTTTCATTTATA
TCAAACACAAACAGCTCAGAGTGGTTATTATAATTTTAATTTCTCATTTCTGAGTCAGTTTGTGTATAAGGCAA
GTGATTTTATGTATGGGTCTTATCATCCTAGGTGTTCTTTTAGACCAGAAACCATTAATAATGATTTGTGGTTTA
ATTCCTTGTCAGTTTCTCTTACTTATGGACCCCTACAGGGAGGGTGTAAGCAATCTGTTTTTAGTGGTAAGGC
AACTTGTTGTTATGCCTACTCTTATAATGGCCCTACAGTATGTAAAGGTGTTTATTCAGGTGAATTAAGCAAGA
CTTTTGAATGTGGATTGCTGGTTTATGTTACTAAGAGTGATGGCTCTCGTATACAGACTAGAACAGAGCCCTT
AGTATTAACGCAACACAATTATAATAATATTACTTTAGATAAGTGTGTTAACTATAATATATATGGCAGAGTGGG
CCAAGGTTTTATTACTAATGTGACTGATTCTGCTGCTAATTTTAGTTATTTAGCAGATGGTGGGTTAGCTATTTT
AGACACTTCGGGTGCCATAGATGTTTTTGTTGCACAGGGCAGCTATGGTCCTAATTATTACAAGGTCAATCCT
TGTGAAGATGTTAACCAACAGTTTGTAGTGTCTGGTGGCAATATAGTTGGCATTCTTACTTCTAGAAATGAAA
CAGGTTCTGAACAGGTTGAGAACCAGTTTTATGTTAAGTTAACCAATAGCTCACATCGTCGTAGGCGC
